# Exercise Testing Characteristics, Safety, and Quality in Patients with Cancer: A Systematic Review

**DOI:** 10.1016/j.mayocpiqo.2025.100628

**Published:** 2025-06-11

**Authors:** Elisabeth Edvardsen, Tormod Skogstad Nilsen, Robert Novo, Michael Curry, Scott C. Adams, Konstantina Matsoukas, Justin Huang, Jasme Lee, Chaya Moskowitz, Lee W. Jones, Jessica M. Scott

**Affiliations:** a Department of Pulmonary Medicine, Oslo University Hospital, Oslo, Norway; b Institute of Physical Performance, The Norwegian School of Sport Sciences, Oslo, Norway; c Memorial Sloan Kettering Cancer Center, New York, NY; d Cancer Fatigue Services, Toronto, ON, Canada; e Hunter College, New York, NY; f Weill Cornell Medical College, New York, NY

## Abstract

**Objective:**

To evaluate exercise testing (ExT) characteristics, safety, and methodological quality in oncology settings.

**Patients and Methods:**

In this systematic review, we searched electronic databases (PubMed, Embase, CINAHL, and Cochrane Library) from inception to March 2024. Studies using ExT to evaluate cardiorespiratory fitness or functional capacity in adults with a history of cancer were included. Summary data including ExT characteristics (eg, modality, cardiorespiratory fitness, and functional capacity results), safety (eg, adverse events [AE]), and methodological quality (eg, guideline adherence) was evaluated.

**Results:**

A total of 642 unique studies with 94,960 patients (58.6±9.9 years; 49% women) were included. Among the 26 ExT modalities used, the maximal cardiopulmonary exercise test (CPET) with measurement of gas exchange (n=284, 40%) and the 6-minute walk test (6MWT) (n=240, 34%) were the most frequently performed. Of the 284 studies that conducted CPET, n=204 (72%) studies reported peak oxygen consumption in mL·kg^-1^·min^-1^; average peak oxygen consumption was 21.8±5.8 mL·kg^-1^·min^-1^. Of the 237 studies that conducted 6MWT, 155 (65%) reported distance in meters (m); average distance was 445m±79 m. A total of n=36 (23%) studies reported an average distance above 500 meter. The AEs were monitored in 58 (9.0%) studies wherein a total of 120 non-serious AEs were reported among n=5699 patients (0.02%). Methodological quality and reporting varied considerably.

**Conclusion:**

The findings of this systematic review indicate that CPET with gas exchange and field-based tests such as the 6MWT are widely used; however, future research should prioritize improving safety monitoring, appropriate test selection, and methodological consistency to enhance the applicability of ExT in oncology care.

**Article Highlights**

• This systematic review provides a comprehensive overview of exercise testing (ExT) across a broad range of cancer types, capturing real-world variability in test selection, protocols, and patient populations.
• We characterized VO₂peak and 6-minute walk distance across diverse cancer sites, highlighting test-specific limitations such as the ceiling effect of the 6MWT in fitter patients.
• Major methodological gaps were identified, including inconsistent safety reporting, limited cardiopulmonary risk screening, and poor adherence to ExT protocols, underscoring the need for oncology-specific reporting standards.

Over the past 3 decades, exercise testing (ExT) has become an essential tool for evaluating cardiorespiratory fitness (CRF) and functional capacity across various clinical populations.^1^, ^2^, ^3^, ^4^, ^5^, ^6^ The ExT provides an objective assessment of the integrative capacity of the cardiopulmonary, vascular, and musculoskeletal systems under physiological stress, offering valuable insights into overall health.^7^ Accordingly, ExT is recommended in routine clinical practice to provide diagnostic and prognostic information and assess intervention efficacy, especially in patients with cardiovascular and pulmonary diseases.^8^^,^^9^ Despite its well-established role in these clinical settings, the application of ExT in oncology is not standard of care. The ExT before, during, and after cancer treatment has the potential to provide clinical physiological insights, including monitoring treatment-related decline in CRF and function, guiding individualized exercise prescriptions, and optimizing patient care.^10^ In addition, ExT can help identify individuals at risk for long-term health complications^11^ and support targeted rehabilitation efforts aimed at enhancing survivorship outcomes.^12^

In 2008, a systematic review evaluated ExT methods and provided recommendations to promote high-quality ExT in clinical oncology.^6^ However, this review was restricted to a limited range of ExT modalities (ie, maximal and submaximal cardiopulmonary exercise tests) and therefore did not evaluate other commonly used ExT, such as the 6-minute walk test (6MWT). In addition, only a small number of studies (n=90) that consisted primarily of patients with breast (38%) or lung (30%) cancers were included. Finally, ExT adverse events (n=10) were reported in less than 15% of studies; therefore, the safety of ExT in patients with cancer is unclear. Accordingly, we conducted a systematic review to update and extend previous work to provide a broad overview of ExT characteristics, safety, and methodological quality in oncology settings.

## Methods

### Search Strategy and Selection Criteria

We performed a systematic search using CINAHL (EBSCO), Cochrane Central Register of Controlled Trials (Wiley), Embase (Elsevier), SPORTDiscus, and PubMed (National Library of Medicine) from inception to March 2024. The MESH terms used were ExT (MeSH Unique ID: D005080) and neoplasms (MeSH Unique ID: D009369), with a combination of keywords (eg, peak oxygen consumption, cardiopulmonary exercise test [CPET], 6MWT, incremental shuttle walk test, and step test). The full search strategy is listed in Supplemental Appendix 1 (available online at http://www.mcpiqojournal.org). In addition, reference lists of primary studies and review articles were screened for additional studies. Studies including prospective and retrospective cohort studies, case-control studies, validation studies, cross-sectional studies, randomized and nonrandomized controlled trials, and uncontrolled trials with solid or hematologic malignancies that included ExT before, during, or after definitive adjuvant therapy were included. ExT was defined as all assessments lasting 2 minutes or longer. Exclusion criteria included preclinical studies, investigations with a participant age <18 years, not written in English, case report studies, abstracts, not published in peer-reviewed journals, review/protocol/editorial articles, pilot studies, and sample sizes less than 20 participants in total. The analysis was conducted in accordance with the Preferred Reporting Items for Systematic Reviews and Meta-analysis (PRISMA). The study was registered in the International Prospective Register of Systematic Reviews (PROSPERO), CRD42018112529.

### Systematic Review Data Extraction and Quality Assessment

Two authors (E.E. and T.S.N.) independently evaluated study eligibility by reviewing the titles and abstracts of all potential citations according to the inclusion criteria and performed data extraction using custom, standardized data abstraction forms in DistillerSR (extracted variables Supplemental Appendix 2, available online at http://www.mcpiqojournal.org). Disagreements were resolved by consensus in review with a third independent author (J.M.S.). Where multiple publications resulted from the same trial, the earliest published result for each outcome was used in the analysis. We extracted study (ie, study design, country of origin, and year of publication), patient (ie, primary cancer site, age, sex, treatment, and comorbidities), ExT characteristics (ie, type, timing, purpose, personnel, test facilities, equipment, protocol, end-criteria for maximal effort, sampling method and interval, and results), and safety (ie, report of any serious or non-serious adverse events (AEs) of any grade during or immediately after the ExT). The ExT methodological quality was evaluated based on description of test protocol and name, personnel conducting the ExT, pretest preparation and familiarization, calibration, definition of ExT end point, and adherence to available guidelines (ie, American Thoracic Society/American College of Chest Physicians statement on CPET^13^ and American Thoracic Society guidelines for the 6MWT].^14^ Quality assessment is outlined in detail in Supplemental Appendix 3 (available online at http://www.mcpiqojournal.org).

### Data Analysis

Descriptive statistics were used to summarize study characteristics, including sample size, cancer site, ExT modality, test protocols, and reported outcomes. Continuous variables were presented as means and standard deviations (SD), whereas categorical variables were reported as frequencies and percentages.

## Results

**Figure 1:**
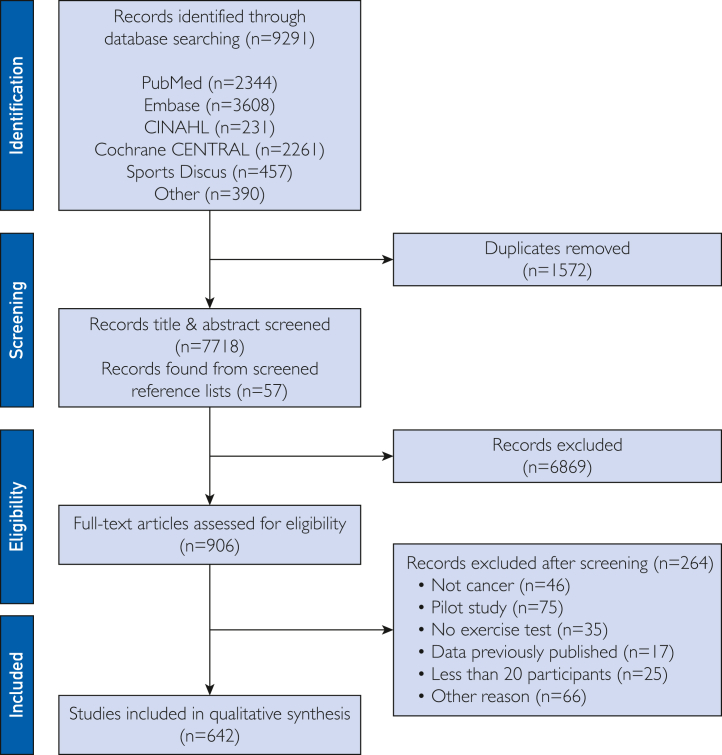
Flowchart of included studies through the review process and its reasons for exclusion according to the Preferred Reporting Items for Systematic Review and Meta-Analyses (PRISMA).

We identified a total of 9291 potential records; 1572 duplicates were removed using the EndNote citation management software program (Clarivate Analytics). A total of 7718 records remained for title or abstract screening. After abstract screening, 906 records underwent full review. Of these, 642 met eligibility criteria and were included in the systematic review (Figure 1).

### Study and Population Characteristics

**Figure 2:**
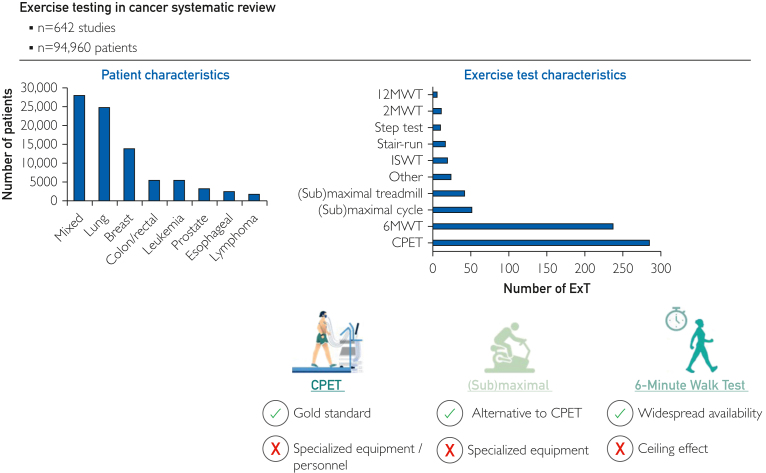
Exercise testing in cancer systematic review.

**Figure 3:**
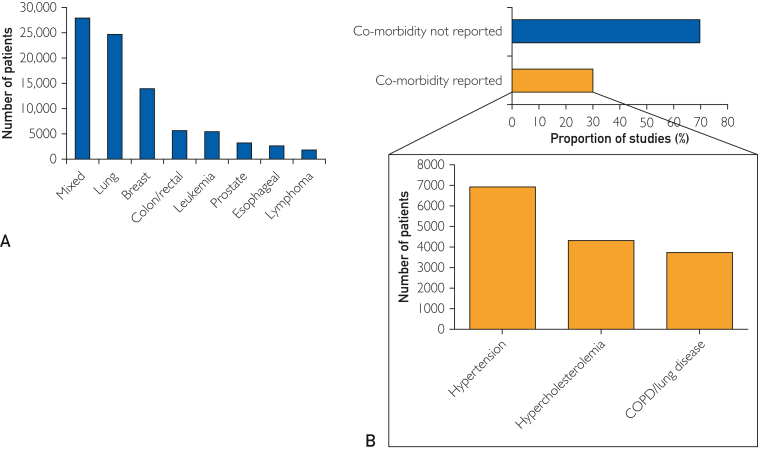
Study participant characteristics. (A) cancer types and (B) comorbidities reporting and number of patients with comorbidities.

The 642 studies included 288 (45%) cohort studies, 229 (36%) randomized controlled trials, 44 (7%) cross-sectional studies, 39 (6%) nonrandomized controlled trials, 22 (3%) one-armed intervention studies, and 20 (3%) case-control studies (Table 1; Figure 2). Sample sizes among the studies ranged from 20 patients to 1967 patients, with a total of 94,960 patients included (mean age: 58.6±9.9 years, 46,150 (49%) female). The most common patient populations were lung cancer (n=24,906, 26%), breast cancer (n=14,012, 15%), colon/rectum cancer (n=5780, 6%), and leukemia (n=5583, 6%) (Figure 3A). Information regarding comorbidities was provided in 194 (30%) studies; hypertension was the most frequently reported comorbidity (n=108 studies, n=6936 patients), followed by hypercholesterolemia (n=43 studies, n=4325 patients) and COPD/lung disease (n=89 studies, n=3755 patients) (Figure 3B). The ExT(s) were performed pretreatment, during treatment, or posttreatment in 171 (27%), 118 (18%), and 188 (29%) studies, respectively (Table 1).

**Table 1:** Study Characteristics for the Systematic Review

Variables	All studies (n=642)
No. of participants	94,960
Male, No. (%)^a^	42,018 (44)
Female, No. (%)^a^	46,173 (49)
Age, mean ± SD	58.6 ± 9.9
Sample size, No. of studies (%)	
Fewer than 50	199 (31)
Between 50 and 100	181 (28)
Between 100 and 499	230 (36)
Above 500	32 (5.0)
Publication year, No (%)	
1982-2014	244 (38)
2015-2024	398 (62)
> 2008^b^	534 (83)
Region of origin, No. (%)	
Europe	317 (49)
Americas	193 (30)
Asia	95 (15)
Oceania	35 (5.5)
Africa	2 (0.3)
Study design No. of studies (%)	
Prospective cohort study	212 (33)
Randomized controlled study	229 (36)
Retrospective cohort study	78 (12)
Non-randomized intervention study	60 (9.3)
Cross-sectional study	43 (6.7)
Case-control study	20 (3.1)
Study population by cancer site, No. of studies (No. of participants)	
Lung	194 (24,906)
Mixed	118 (28,012)
Breast	118 (14,012)
Colon/rectum	43 (5780)
Prostate	35 (3329)
Esophageal cancer	28 (2670)
Leukemia	21 (5583)
Lymphoma	14 (1890)
Other^c^	71 (8778)
Treatment, No. of studies (%)	
Surgery	361 (56)
Drug therapy	272 (33)
Radiation	178 (28)
Antihormone treatment	79 (12)
Stem cell transplant	41 (6.4)
Other treatment^d^	20 (3.1)
Settings, No. of studies (No. of participants)	
Posttreatment	188 (33,239)
Pretreatment	171 (26,531)
Pre-posttreatment	110 (14,966)
During treatment	116 (13,584)
Preduring treatment	25 (2169)
During-posttreatment	15 (2597)
Pre-during-posttreatment	11 (1331)
Other setting	6 (543)

a 4.2% of studies (n=27) did not report number of males and females.

b Number of studies published since the prior systematic review.^6^

c Other cancer sites included bladder, bone, brain, gastro, gynecologic, head/neck, liver, sarcoma, myeloma, pancreatic, testicular, and urological cancer.

d Other treatment included active surveillance (n=3), antivascular endothelial growth factor therapy (n=1), corticosteroids (n=1), EPO (n=2), erythropoietin (n=2), estrogen (n=1), herceptin (n=1), immune therapy (n=3), and estrogen (n=1).

**Figure 4:**
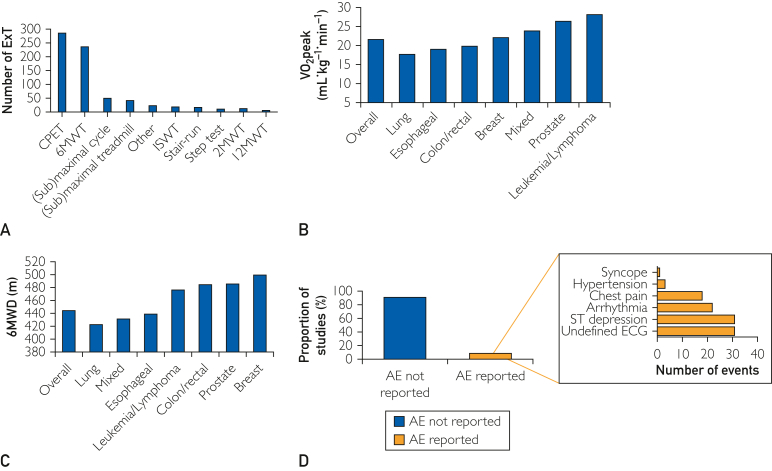
ExT characteristics. (A) exercise test modalities; (B) peak oxygen consumption; (C) 6-minute walk distance; and (D) adverse event reporting and number of adverse events. CPET, cardiopulmonary exercise test; MWT, minute walk test; ISWT, incremental shuttle walk test; ExT, exercise test.

Of the 642 studies, 58 (9.0%) included more than one ExT, resulting in a total of 705 ExTs (Figure 4A). Among the 26 unique ExT used, maximal CPET with measurement of gas exchange (n=284, 40%) and the 6MWT (n=240, 34%) were the most commonly performed (Supplemental Tables 1 and 2 [available online at http://www.mcpiqojournal.org]). Overall, 204 of 284 (72%) studies performing CPET reported peak oxygen consumption (VO_2_peak) values in mL·kg^-1^·min^-1^; among studies performing 6MWT, 155 of 240 (65%) reported 6MWD (Supplemental Table 3, [available online at http://www.mcpiqojournal.org]). Average VO_2_peak across all studies was 21.8±5.8 mL·kg^-1^·min^-1^, with a range among tumor subtypes (Figure 4B). Overall 6MWD was 445m±79 m, with a wide range across tumor subtypes (Figure 4C). Among the 155 studies reporting 6MWD, n=36 (23%) reported an average 6MWD above 500 meter. Information regarding AEs was reported in 59 studies (9%), of which 45 (76%) clearly stated that no AEs were observed (Supplemental Table 4, [available online at http://www.mcpiqojournal.org]). A total of 120 AEs were reported among n=5699 patients (∼2%) (breast: n=19 AE in n=624 patients; colon/rectal/esophageal: n=3 AE in n=514 patients; and lung: n=13 AE in 1098 patients) consisting of events such as electrocardiogram changes, dyspnea, chest pain, lack of or excessive increase in blood pressure response, post-exercise hypotension, dizziness, and muscle-related pain (Figure 4D).

A full description of ExT protocol was reported in 309 studies (48%), whereas 190 (30%) did not report any information regarding the ExT protocol (Table 2). Familiarization or pretest preparation was reported for 52 (8.1%) tests, and test personnel qualifications were reported in 118 (18%) studies. Of the 284 CPETs, calibration procedures were mentioned or reported in 52 studies (19%), and VO_2_peak criteria for maximal effort were defined in 168 studies (59%). Twenty-four (36%) of the 66 studies that indirectly estimated VO_2_peak provided the estimation equation. Of the 240 studies that performed 6MWT, 8 (3.3%) reported a 10-minute seated chair rest before the start, 25 (11%) measured resting blood pressure, and 29 (12%) recorded level of dyspnea.

**Table 2:** ExT Quality of Conduct, Results, and Safety

	CPET 284 (40)	6MWT 240 (34)	(Sub)maximal bicycle test 51 (7.2)	(Sub)maximal treadmill test 42 (6.0)	ISWT 19 (2.7)	Stair-run test 17 (2.4)	Step test 10 (1.4)	2MWT 12 (1.7)	12MWT 6 (0.9)	Other test^a^ 24 (3.4)
Conduct										
Pretest procedure	34 (12)	21 (8.8)	3 (5.9)	3 (7.1)	4 (22)	0 (0)	0 (0)	0 (0)	1 (17)	1 (4.2)
Full description of test protocol	166 (59)	82 (34)	28 (55)	14 (33)	8 (44)	10 (59)	5 (50)	3 (25)	1 (17)	7 (29)
Partial description of test protocol	47 (167)	58 (24)	5 (9.8)	16 (38)	4 (22)	3 (18)	2 (20)	4 (33)	0 (0)	6 (25)
Presence of type of ExT personnel	61 (22)	51 (21)	7 (14)	6 (14)	2 (11.1)	9 (53)	2 (20)	1 (8.3)	1 (16.7)	2 (8.3)
Equipment brand	152 (54)	19 (7.9)	17 (33)	9 (21)	1 (5.6)	1 (5.9)	N/A	N/A	N/A	3 (13)
Equipment calibration	52 (18)	N/A	2 (3.9)	1 (2.4)	N/A	N/A	N/A	N/A	N/A	1 (4.2)
Sampling-interval for VO_2_peak	113 (40)	N/A	3 (5.9)	2 (4.8)	2 (11.1)	N/A	N/A	N/A	N/A	N/A
VO_2_peak end-criteria	168 (59)	N/A	4 (7.8)	3 (7.1)	2 (11.1)	N/A	N/A	N/A	N/A	1 (4.2)
Symptom-limited endpoint	98 (35)	N/A	N/A	N/A	N/A	N/A	N/A	N/A	N/A	N/A
Results										
Primary endpoint	204 (72)	155 (79)	34 (65)	34 (81)	17 (94)	11 (65)	6 (60)	9 (75)	4 (67)	21 (88)
Safety										
Safety monitoring before, during or after the test	98 (35)	25 (10)	14 (28)	11 (26)	3 (17)	9 (53)	2 (20)	1 (8.3)	0 (0)	3 (13)
Physician present	18 (6.3)	7 (2.9)	0 (0)	2 (4.8)	N/A	8 (47)	1 (10)	N/A	N/A	N/A
AE observed	11 (3.9)	3 (2.1)	2 (3.9)	1 (2.4)	1 (5.6)	0 (0)	0 (0)	0 (0)	0 (0)	0 (0)
AE not observed	17 (6.0)	20 (8.3)	7 (14)	4 (9.5)	1 (5.6)	2 (11.8)	0 (0)	0 (0)	1 (17)	1 (4.2)
Total number of AEs	74	5	14	27	0	0	0	0	0	0
Total number of different AEs^b^	11	4	6	3	0	0	0	0	0	0

Data reported as number (%).

Abbreviations: AE, adverse events; CPET, cardiopulmonary exercise test; ISWT, incremental shuttle walk test; MWT, minute-walk-test; N/A, not applicable; VO_2_peak, peak oxygen consumption.

a Other tests included 400-meter walk test (n=6), modified 6MWT (3), Rockport one-mile fitness walking test (3), UKK walk test (3), one-mile walk test (2), Canadian aerobic fitness test (1), Cooper test (1), lactate test (1), and steep ramp test (1).

b AE included abnormalities in VO_2_ or the oxygen equivalent (n=2), arrhythmias (n=22), atrial fibrillation (n=3), chest pain/symptoms of ischemia (n=18), ECG abnormalities (n=31), drop in systolic BP (n=6), hypertension (n=3), orthopedic problems (n=NR), pain (n=3), syncope before start (n=1), severe dyspnea (n=NR), and ST depression (n=31).

## Discussion

The ExT provides an assessment of the integrative functional capacity of the cardiopulmonary, vascular, and musculoskeletal systems during stress.^7^ The centricity of ExT in non-cancer clinical populations^13^^,^^15^ for diagnosis, assessment of disease severity and progression, prognosis, and gauge for therapeutic efficacy^8^ suggests that these tests could be also employed in oncology settings to provide more objective and precise measures of CRF and functional capacity. In this systematic review, we generated a comprehensive overview of the ExT landscape in cancer. Our findings indicate that ExT is frequently used in a wide variety of oncology settings. However, variability in testing modalities and reporting highlights the need for adherence to standardized guidelines for implementation in patients with cancer.

In oncology clinical practice and research, Karnofsky (KPS) or Eastern Cooperative Oncology Group scoring scales^16^ are commonly used to evaluate functional capacity. Although entrenched into contemporary practice and research, these performance status scores were developed >60 years ago, remain subjective, and have limited reliability.^17^, ^18^, ^19^, ^20^, ^21^ In contrast, ExT provides objective assessments of CRF and functional capacity,^8^ and therefore has several advantages over alternative methods and tools that typically evaluate single systems at rest.^16^ Clearly, a variety of ExT are available to quantify CRF and functional capacity; however, given the available guidelines, existing normative values, and widespread use,^13^^,^^14^ a maximal CPET, a submaximal field test 6MWT, or a (sub)maximal test are likely the most appropriate tools to determine CRF and functional capacity across the cancer care continuum. Importantly, selection of which ExT modality to employ should be based on patient risk (eg, cardiovascular disease, and frailty), purpose (eg, prognostication and guiding exercise training), and feasibility (eg, available equipment). For instance, the gold standard assessment of CRF is CPET coupled with automated gas exchange assessment.^8^ This test is appropriate for all patients and provides multiple metrics regarding the pathogenetic/clinical mechanisms underpinning poor CRF that can be used for prognostication and to inform exercise training.^8^ The need for specialized equipment and trained personnel, however, may limit widespread clinical application.^15^ (Sub)maximal ExT may be an appropriate alternative to determine CRF when the requisite CPET facilities or qualified personnel are not available.^8^ The 6MWT is advantageous because it does not require high-tech equipment and can therefore be performed in most clinical centers. However, the 6MWT is likely only appropriate for markedly deconditioned patients due to ceiling effects that limits discrimination between individuals that can complete more than 500 meter.^6^ As a result, the 6MWT may not effectively detect significant improvements in physically fit patients who are already near the upper limit of the test (∼500 m). Despite this limitation, many studies did not acknowledge the ceiling effect, potentially impacting the accuracy of their findings. For example, we found that one in 5 studies that reported the 6MWT results had an average walking distance exceeding 500 meter, suggesting that a substantial proportion of patients may have been too fit for this test. Using the 6MWT in such populations reduces its sensitivity to detect meaningful changes, potentially underestimating cancer treatment-related toxicity and limited its ability to capture the true effects of exercise interventions.

The findings of this review are consistent with previous analyses highlighting persistent limitations in methodological quality and reporting in ExT research.^6^^,^^22^, ^23^, ^24^ For instance, in a systematic review of 60 studies that evaluated CRF in 2104 stroke survivors, van de Port et al^22^ reported that less than 50% of studies adhered to ExT recommendations. Similarly, in a 2008 systematic review of 90 studies including 5179 patients with adult-onset cancers, Jones et al^6^ found that most studies did not follow fundamental ExT principles. In the present systematic review, as much as 75% of the studies performing CPET did not report or specify end-criteria for VO_2_peak, which may compromise the accuracy of CRF assessment.^25^ Without clearly defined physiological or subjective exhaustion markers such as respiratory exchange ratio, plateau in VO_2_ despite increasing workload and ventilation, blood lactate, or achievement of age-predicted maximal heart rate, VO_2_peak values may be underestimated,^25^ leading to misclassification of CRF and potential underestimation of treatment-related decline in fitness. Nevertheless, similar to Jones et al,^6^ we found no exercise-related deaths and a low adverse event rate (∼2%). However, these safety findings should be interpreted with caution, as only ∼10% of the studies implemented safety monitoring, highlighting a potential underreporting issue. Moreover, the majority of these AEs were clinically significant, such as arrhythmias, ischemia, and blood pressure abnormalities, which reinforce the role of ExT in the comprehensive assessment of patients with cancer, particularly those undergoing treatment with potential cardiotoxic effects, where early detection of cardiovascular abnormalities is crucial for risk stratification and clinical decision-making.^26^ Finally, the absence of comorbidity risk screening reporting in as much as 70% of the studies is a significant limitation, as up to 60% of patients with cancer have pre-existing or treatment-related comorbidities^27^ that could influence safety and choice of ExT modality. Overall, despite available general ExT guidelines^13^^,^^14^ and cancer-related recommendations in 2008^6^ for reporting of ExT methods and results, there has been minimal improvement in ExT methodological quality. Moving forward, development of ExT reporting requirements akin to Consolidated Standards of Reporting Trials (CONSORT)^28^ could augment methodological rigor and reproducibility, and enhance results and safety reporting quality.

### Strengths and Limitations

A major strength of this systematic review is the comprehensive overview, which captures the real-world variability in how ExT is used across different oncology populations. Cancer diagnoses, treatments, and functional impairments vary widely, leading to diverse applications of ExT in clinical and research settings. By including multiple cancer types and all endurance ExT modalities, we provide a comprehensive overview of current ExT practices, rather than a narrow, disease-specific perspective. In addition, this broad approach allowed us to characterize VO_2_peak and 6MWD across a wide range of cancers and identify key methodological gaps in ExT, such as inconsistent safety reporting, lack of cardiopulmonary risk assessment, and variability in choice of ExT protocols and modalities.

Some limitations require consideration. First, most included studies consisted of patient cohorts with lung cancer and were conducted in Europe, where ExT is routinely performed to determine risk of surgical complications in patients with lung cancer care.^29^ In addition, ∼45% of included studies were intervention trials, where patients were likely carefully screened and excluded for severe cardiovascular disease or other risks before participation. Thus, broad generalizability of ExT to clinical management may be limited. Finally, in contrast to other chronic diseases where ExT data were captured in large cohorts (n=5000 to >700,000), only ∼10 oncology studies with relatively small sample sizes (n≤1000) were specifically designed to address maximal exercise testing-related adverse events.

## Conclusion

In summary, our findings suggest that the maximal CPET, (sub)maximal laboratory-based tests, and field-based tests such as the 6MWT are commonly used and generally safe in patients with cancer. Standardized, formal ExT guidelines, including test selection, methods, and data reporting, are needed to ensure high-quality evaluation of CRF and functional status and to strengthen the role of ExT in both clinical oncology research and clinical practice.

## Potential Competing Interests

Dr Edvardsen report personal consulting fees, honoraria, and expenses for presentations at education meetings from GlaxoSmithKline, AstraZeneca Norway, Birk NPC AS and Chiesi Pharma AB. Dr Jones has stock ownership in Pacylex, Inc and Illumisonics, Inc. Dr Adams has stock ownership in Cancer Fatigue Services Inc. All other authors declare no competing interests.

## Ethics Statement

This study is a systematic review of previously published literature and did not involve human subjects or patient data; therefore institutional review board approval was not required.
