# Supplementary material for: Exercise Testing Characteristics, Safety, and Quality in Patients with Cancer: A Systematic Review
Source: Mayo Clin Proc Innov Qual Outcomes. 2025 Jun 11;9(4):100628. doi: 10.1016/j.mayocpiqo.2025.100628 (PMC12182365; doi:10.1016/j.mayocpiqo.2025.100628)
Supplement: Supplementary Data [file mmc1.docx]

## Supplementary Online Content

Edvardsen et al., Exercise Testing, Cardiorespiratory Fitness, and All-Cause Mortality in Cancer: A Systematic Review and Meta-Analysis

**Appendix 1.** Search Strategy

**Appendix 2.** Extracted Variables

**Appendix 3.** Exercise Test Quality of Conduct, Results, and Safety

**Table S1.** Cardiopulmonary Exercise Test (CPET) Characteristics

**Table S2.** Six-minute Walk Test Characteristics

**Table S3.** Peak Oxygen Consumption and 6 Minute Walk Distance Summary Results.

**Table S4.** Exercise Test Preparation and Safety

**Supplemental References.**

## Appendix 1. Search Strategy

Comprehensive searches were conducted on 9/27/2018 in five electronic databases:

1. PubMed/Medline (National Library of Medicine)
2. EMBASE (Elsevier)
3. CINAHL (EBSCO)
4. Cochrane Central Register of Controlled Trials (Wiley)
5. SPORTDiscus^TM^

The literature search strategy was developed first in PubMed and translated to the other databases. A combination of relevant keywords and controlled vocabulary were used in the PubMed, EMBASE, and CINAHL searches and a keyword search strategy were used in Cochrane Central Register of Controlled Trials (CENTRAL).

No date restrictions were applied. Results were limited to Human clinical trials. MEDLINE records were excluded from CINAHL and EMBASE results sets.

These strategy components were combined using AND (Boolean operator):

1. Endurance functional capacity testing
2. Cancer
3. Study designs (RCTs, Prospective and retrospective cohort studies)
4. Adults
5. English
6. Human

Each of the search strategy components was first searched upon individually, combining synonyms describing that concept with the Boolean operator OR.

Available search filter used for “Cancer” concept:

The Cochrane Childhood Cancer Group (<http://childhoodcancer.cochrane.org/>) search strategy for *Cancer* was used in PubMed (and adapted it to the other databases):

(cancer OR cancers OR cancerous OR oncology OR oncolog* OR neoplasm OR neoplasms OR neoplasm* OR carcinoma OR carcinom* OR tumor OR tumour OR tumors OR tumours OR malignan* OR malignant OR "hematooncological" OR "hemato oncological" OR "hemato-oncological" OR hematologic neoplasms OR hematolo*)

Available search filters used for study designs:

<http://work.cochrane.org/pubmed>
**sensitivity- and precision-maximizing version (2008 revision); PubMed format^1^**
(randomized controlled trial[pt] OR controlled clinical trial[pt] OR randomized[tiab] OR placebo[tiab] OR clinical trials as topic[mesh:noexp] OR randomly[tiab] OR trial[ti] NOT (animals[mh] NOT humans [mh]))

<http://work.cochrane.org/embase>
**Embase search strategy for finding RCTs in Embase^1^**('crossover procedure':de OR 'double-blind procedure':de OR 'randomized controlled trial':de OR  'single-blind procedure':de OR (random* OR  factorial* OR crossover* OR cross NEXT/1 over* OR placebo* OR doubl* NEAR/1 blind* OR singl* NEAR/1 blind* OR assign* OR allocat* OR volunteer*):de,ab,ti)

1. Lefebvre C, Manheimer E, Glanville J. Chapter 6: Searching for studies. In: Higgins J, Green S (editors). Cochrane Handbook for Systematic Reviews of Interventions. Version 5.1.0 (updated March 2011). The Cochrane Collaboration, 2011. Available from [**www.cochrane-handbook.org**](http://www.cochrane-handbook.org/)

<http://libguides.sph.uth.tmc.edu/search_filters/pubmed_filters>

**Cohort studies (revised 1/10/2014)**
cohort studies[mesh:noexp] OR longitudinal studies[mesh:noexp] OR follow-up studies[mesh:noexp] OR prospective studies[mesh:noexp] OR retrospective studies[mesh:noexp] OR cohort[TIAB] OR longitudinal[TIAB] OR prospective[TIAB] OR retrospective[TIAB]

Resulting citations were managed and duplicates removed using the Endnote citation management software program (Clarivate Analytics).

|  | rategy |
| --- | --- |
| 1 | (("Walk Test"[MeSH] OR "Exercise Test"[MeSH] OR "Exercise Tolerance"[MeSH] OR "Physical Endurance"[MeSH Terms] OR "CPET" OR “MWT” OR "6MWT" OR "12MWT" OR "10MWT" OR "6MWD" OR "ISWT" OR "ETT" OR "exercising" OR "exercise-based" OR “cardiopulmonary exercise test” OR “VO2peak” OR “VO2max” OR “maximal oxygen uptake” OR "treadmill" OR "walk" OR "walking" OR "Walking"[MeSH] OR "shuttle" OR "cycle ergometer" OR "cycle ergo-meter" OR "physical endurance" OR "functional capacity" OR "Stair Climbing"[Mesh] OR "stair-climbing" OR "stair climb" OR "ramp" OR "stairs") AND ("test" OR "tests" OR "testing"))) |
| 2 | (cancer OR cancers OR cancerous OR oncology OR oncolog* OR neoplasm OR neoplasms OR neoplasm* OR carcinoma OR carcinom* OR tumor OR tumour OR tumors OR tumours OR malignan* OR malignant OR "hematooncological" OR "hemato oncological" OR "hemato-oncological" OR hematologic neoplasms OR hematolo*) |
| 3 | (randomized controlled trial[pt] OR controlled clinical trial[pt] OR randomized[tiab] OR placebo[tiab] OR clinical trials as topic[mesh:noexp] OR randomly[tiab] OR trial[ti] OR cohort studies[mesh:noexp] OR longitudinal studies[mesh:noexp] OR follow-up studies[mesh:noexp] OR prospective studies[mesh:noexp] OR retrospective studies[mesh:noexp] OR cohort[TIAB] OR longitudinal[TIAB] OR prospective[TIAB] OR retrospective[TIAB]) |
| 4 | (English[lang] AND adult[MeSH]) |
| 5 | NOT (animals[mh] NOT humans [mh]) |
| 6 | 1 AND 2 AND 3 AND 4 |
| 7 | 6 NOT 5 |

Abbreviations: **MeSH** = Medical Subject Heading, **pt** = Publication Type, **TIAB** = Title/Abstract, **ti** = Title, **mh** = MeSH Terms

## Appendix 2. Extracted Variables

The following study details were extracted: study characteristics (journal, title, first author, country, region of origin, year of publication, study design), study population (cancer population, mean age, age range, total number of: patients, males, females), treatment characteristic (cancer treatment type, time of treatment: pre, during or post), exercise tests used (type and protocol details, number and timing of tests, setting, pretest preparation, modality, end criteria used, test monitoring, who performed the test, test-qualification, maximal or submaximal, direct or indirect, type of equipment described, sampling method, calibration routine), outcome variables including measured or estimated VO_2_peak, safety (number and type of adverse events, safety monitoring, physician present).

## Appendix 3. Exercise Test Quality of Conduct, Results, and Safety

Quality of Conduct

Equipment used during CPET and submaximal tests were fully described including brand name (type, name and country). Equipment calibration, only relevant for CPET, was included if information of the calibration procedures was given according to standards given by the manufactory. Sampling-interval, only relevant for CPET, was defined if the sampling-interval for peak oxygen uptake (VO_2_peak) was reported or not. VO_2_peak end-criteria, only relevant for CPET, was included if one or more of the following criteria was used: leveling off in VO_2_, a defined cut-off value for respiratory exchange ratio, peak heart rate, blood lactate, rating of perceived exertion scale or symptom limited end-points.

Question asked for Quality of Conduct:

- CONSORT (5b) – Were details of whether and how the exercise test(s) were standardized given?
- Who performed the test?
- Is the type of equipment used described?
- Is the equipment used calibrated according to standards given by the manufactory?
- Leveling off in VO_2_?
- Definition for RER?
- Definition for percent of predicted heart rate?
- Definition for blood lactate?
- Definition for RPE (Borg scale)?
- If other end-criteria used, please list
- What sampling interval was used for the high-tech test?

Results

Question asked for results:

- What was the overall purpose of the endurance exercise test(s)?
- What was the primary endpoint?
- What was the secondary endpoint(s)?

Safety

Safety was defined as exercise-related adverse events (AE) occurring during or after the exercise test. Supervision during the test was defined as if there were any predefined safety (medical) measures before, during or after the test.

Question asked for safety:

- Who performed the test?
- Were there any predefined safety measures (e.g. medical supervision before or during the test, or post-test observation)?
- Did any testing-related AE occur?
- If so, how many?
- What kind of AE were reported?

### Table S1. Cardiopulmonary Exercise Test (CPET) Characteristics (n=284 studies)

| Study |  | Study-design | Time  Points | Overall purpose | Modality | Protocol | | | Monitoring | VO_2_peak  (mLˑkg^-1^ˑmin^-1^)  (Lˑmin^-1^)  (% of pred) |
| --- | --- | --- | --- | --- | --- | --- | --- | --- | --- | --- |
|  | Cancer Site |  |  |  |  | VO_2_ definitions | Variable reported | VO_2_peak Criteria |  |  |
| Banerjee, 2018^1^ | Bladder | Randomized controlled trial | Pre-treatment | Evaluate the effect of an intervention | CE | NR | VO_2_peak | Maximum exercise tolerance | No calibration reported  AE: NR  Predefined Safety Measures: Yes | Overall: NR  Intervention: 20.4 mLˑkg^-1^ˑmin^-1^  Control: 20.4 mLˑkg^-1^ˑmin^-1^ |
| Blackwell, 2020 | Bladder | Randomized controlled trial | Pre-treatment | To evaluate the effect of an intervention | CE | 20 sec sampling | VO₂ AT | Exhaustion Voluntary termination | Calibration: No Predefined Safety Measures: NR AE: NR | Overall: NR  Intervention: 26.4 mLˑkg^-1^ˑmin^-1^  Control: 24.8 mLˑkg^-1^ˑmin^-1^ |
| Lamb, 2016^2^ | Bladder | Prospective cohort study | Pre-treatment | Pre surgery evaluation | CE | 30 sec sampling | VO_2_peak | Voluntary termination | No calibration reported  AE: NR  Predefined Safety Measures: Yes | 15.0 mLˑkg^-1^ˑmin^-1^ |
| Longdon, 2020 ^3^ | Bladder | Prospective cohort study | Pre-treatment | Pre surgery evaluation | CE | NR sec sampling | VO₂ AT VEVCO₂ VO₂peak | NR | Calibration: Calibration procedures are mentioned, but not when and how Predefined Safety Measures: No AE: NR | 15.20 mLˑkg⁻¹ˑmin⁻¹ 81.0% pred |
| Tolchard, 2015^4^ | Bladder | Prospective cohort study | Pre-treatment | Pre surgery evaluation | CE | 10 sec period at peak exercise | VO_2_peak  Anaerobic threshold  VEVCO_2_ | Exhaustion | No calibration reported  AE: NR  Predefined Safety Measures: No | 15.2 mLˑkg^-1^ˑmin^-1^ (median) |
| An, 2020^5^ | Breast | RCT | Pre-during treatment | Evaluate the effect of an intervention | NR | NR | NR | NR | No calibration reported  Testing Related AE: NR  Predefined Safety Measures: No | NR |
| Bell, 2021 ^6^ | Breast | Randomized controlled trial | Post-treatment | To evaluate the effect of an intervention | CE | 10 sec sampling | VO₂ VE HR BP Wmax | Exhaustion | Calibration: No Predefined Safety Measures: NR AE: NR | 44% and 51% of predicted |
| Bender, 2021 ^7^ | Breast | Cross-sectional study | Pre-treatment | Pre therapy assessment | TM | NR sec sampling | HR VO₂peak | Pred HR RPE/BORG Exhaustion | Calibration: No Predefined Safety Measures: NR AE: NR | 1.38 Lˑmin⁻¹ |
| Bender, 2024 ^8^ | Breast | Randomized controlled trial | Post-treatment | To evaluate the effect of an intervention | TM | NR sec sampling | NR | Pred HR RPE/BORG | Calibration: No Predefined Safety Measures: NR AE: NR | Overall: NR  Intervention:  17.5 mLˑkg^-1^ˑmin^-1^  Control:  17.7 mLˑkg^-1^ˑmin^-1^ |
| Berling-Ernst, 2022 ^9^ | Breast | Cross-sectional study | Post-treatment | Compare cancer vs. Non cancer | CE | NR sec sampling | NR | RPE/BORG | Calibration: No Predefined Safety Measures: NR AE: NR | 26 mLˑkg^-1^ˑmin^-1^ |
| Bigaran, 2022 ^10^ | Breast | Non-randomized controlled trial | Pre-treatment; Pre-post treatment | To evaluate the effect of an intervention | NR | 30 sec sampling | ECG BP VO₂ Wmax | NR | Calibration: No Predefined Safety Measures: NR AE: NR | Overall: NR  Intervention:  27.0 mLˑkg^-1^ˑmin^-1^  Control:  22.0 mLˑkg^-1^ˑmin^-1^ |
| Bohn, 2021 ^11^ | Breast | Randomized controlled trial | Pre-treatment | To evaluate the effect of an intervention | TM | NR sec sampling | VO₂ | NR | Calibration: No Predefined Safety Measures: NR AE: NR | Overall: NR  Intervention:  30.1 mLˑkg^-1^ˑmin^-1^  Control:  28.9 mLˑkg^-1^ˑmin^-1^ |
| Bonsignore, 2017^12^ | Breast | Retrospective cohort study | Pre-post treatment | Evaluate the effect of an intervention | CE | 20 sec sampling | VO_2_peak | NR | Unclear calibration procedures  AE: NR  Predefined Safety Measures: NR | 15.0 mLˑkg^-1^ˑmin^-1^ |
| Bonsignore, 2021 ^13^ | Breast | Prospective cohort study | Post-treatment | Evaluate late toxiciy > 1 year post-therapy | CE | 15 to 20 sec interval sec sampling | AT/VT HR O₂-pulse VE VEVCO₂ VO_2_peak | Pred HR RPE/BORG Exhaustion | Calibration: No Predefined Safety Measures: NR AE: NR | 19.1 mLˑkg⁻¹ˑmin⁻¹ 84.2% pred |
| Burnett, 2013^14^ | Breast | Cross-sectional study | Post treatment | Evaluate validity (e.g. VO_2_peak vs ISWT) | TM | 15 sec sampling | VO_2_peak  Anaerobic threshold  HR Peak  O_2_ pulse | Leveling off  RER>1.1  >90% of age pred HR  Borg>17  Exhaustion | Yes calibration reported  AE: No  Predefined Safety Measures: Yes | 25.4 mLˑkg^-1^ˑmin^-1^  74% pred |
| Cheema, 2006 ^15^ | Breast | Prospective cohort study | Post treatment | To evaluate the effect of an intervention | CE | NR sec sampling | VO₂ HR RER VO₂peak | Exhaustion | Calibration: Yes Predefined Safety Measures: No AE: NR | 24.30 mLˑkg⁻¹ˑmin⁻¹ 1.7 Lˑmin⁻¹ |
| Chung, 2022 ^16^ | Breast | Randomized controlled trial | During-post treatment | To evaluate the effect of an intervention; Evaluate acute toxicity < 1 year post-therapy | CE | NR sec sampling | HR VEVCO₂ VEVO₂ RER PETCO₂ PETO₂ Wmax METs | NR | Calibration: No Predefined Safety Measures: NR AE: NR | Overall: NR  Intervention:  16.8 mLˑkg^-1^ˑmin^-1^  Control:  14.6 mLˑkg^-1^ˑmin^-1^ |
| Cornette, 2016^17^ | Breast | Randomized controlled trial | During treatment | Evaluate the effect of an intervention | CE | The highest oxygen uptake during exercise | VO_2_peak | NR | No calibration reported  AE: NR  Predefined Safety Measures: Yes | Overall: NR  Intervention:  22.5 mLˑkg^-1^ˑmin^-1^  Control:  23.4 mLˑkg^-1^ˑmin^-1^ |
| Courneya, 2003^18^ | Breast | Randomized controlled trial | Post treatment | Evaluate the effect of an intervention | CE | 15 sec sampling | VO_2_peak  HR Peak  RER | Exhaustion  Leveling off in VO2  RER ≥ 1.0  RPE > 19 | Calibration procedures described in detail  Testing-Related AE: No  Predefined Safety Measures: NR | Overall: NR  Intervention:  18.6 mLˑkg^-1^ˑmin^-1^  Control:  18.8 mLˑkg^-1^ˑmin^-1^ |
| Courneya, 2007^19^ | Breast | Randomized control trial | During treatment | Evaluate the effect of an intervention | TM | NR | VO_2_peak  Anaerobic threshold | NR | No calibration reported  AE: NR  Predefined Safety Measures: No | Overall: NR  RET: 25.5  AET: 24.8  UC: 1.76 L/min  RET: 1.73 L/min  AT: 1.72 L/min |
| Courneya, 2008^20^ | Breast | Randomized controlled trial | During treatment | Evaluate the effect of an intervention | NR | NR | VO_2_peak | NR | No calibration reported  AE: NR  Predefined Safety Measures: NR | 27.2 mLˑkg^-1^ˑmin^-1^  NR |
| Courneya, 2014^21^ | Breast | Randomized controlled trial | Pre-treatment | Evaluate the effect of an intervention | NR | NR | NR | NR | No calibration reported  AE: NR  Predefined Safety Measures: NR | NR |
| Diaz-Balboa, 2022 ^22^ | Breast | Cross-sectional study | During treatment | Method study | CE | NR sec sampling | ECG BP SpO₂ Borg VO₂ VO_2_peak | Exhaustion Voluntary termination | Calibration: No Predefined Safety Measures: Yes AE: NR | 20.83 mLˑkg⁻¹ˑmin⁻¹ 1.416 Lˑmin⁻¹ |
| Dolan, 2010 ^23^ | Breast | Randomized controlled trial | Pre-post treatment | To evaluate the effect of an intervention | TM | NR sec sampling | Hb VO₂ VO_2_peak | NR | Calibration: No Predefined Safety Measures: No AE: NR | 25.20 mLˑkg⁻¹ˑmin⁻¹ |
| Foulkes, 2023 ^24^ | Breast | Randomized controlled trial | Pre-post treatment | To evaluate the effect of an intervention | CE | 30 sec sampling | NR | NR | Calibration: No Predefined Safety Measures: NR AE: NR | Overall: NR  Intervention:  23.6 mLˑkg^-1^ˑmin^-1^  Control:  22.8 mLˑkg^-1^ˑmin^-1^ |
| Friedenreich, 2022 ^25^ | Breast | Prospective cohort study | Post-treatment | Evaluate late toxicity > 1 year post-therapy | TM | NR sec sampling | AT/VT HR VO_2_peak | NR | Calibration: No Predefined Safety Measures: NR AE: NR | 26.6 mLˑkg⁻¹ˑmin⁻¹ |
| Giallauria, 2014^26^ | Breast | Randomized controlled trial | During treatment | Evaluate the effect of an intervention | CE | 20 sec sampling | VO_2_peak | Exhaustion | No calibration reported  AE: NR  Predefined Safety Measures: NR | Overall: NR  Intervention:  12.6 mLˑkg^-1^ˑmin^-1^  Control:  12.8 mLˑkg^-1^ˑmin^-1^ |
| Hokken, 2009^27^ | Breast | Prospective cohort study | Post treatment | Evaluate the effect of an intervention | CE | NR | VO_2_peak | NR | No calibration reported  AE: NR  Predefined Safety Measures: NR | 17.8 mLˑkg^-1^ˑmin^-1^ |
| Hughes, 2015^28^ | Breast | Randomized controlled trial | Post treatment | Evaluate the effect of an intervention | CE | 15 sec sampling | NR | NR | No calibration reported  AE: NR  Predefined Safety Measures: NR | 19.8 mLˑkg^-1^ˑmin^-1^ |
| Isanejad, 2023 ^29^ | Breast | Randomized controlled trial | During treatment | To evaluate the effect of an intervention | TM | 45 sec sampling | HR O₂-pulse VE VEVCO₂ VEVO₂ VCO₂ ECG BP | Leveling off in VO₂ RER Pred HR RPE/BORG | Calibration: No Predefined Safety Measures: Yes AE: No | NR |
| Jacquinot, 2022 ^30^ | Breast | Randomized controlled trial | Pre-during treatment | To evaluate the effect of an intervention | CE | 30 sec sampling | AT/VT HR O₂-pulse VE VEVCO₂ RER BP ECG Wmax | Judgment by the technician Exhaustion | Predefined Safety Measures: Yes AE: NR | Overall: NR  Intervention:  25.7 mLˑkg^-1^ˑmin^-1^  Control:  24.3 mLˑkg^-1^ˑmin^-1^ |
| Jones, 2012^31^ | Breast | Retrospective cohort study | Pre-during-post treatment | 1) Pre-post treatment evaluation other than surgery  2) Evaluate acute toxicity  3) Evaluate late toxicity > 1-year post-therapy | CE | 30 sec sampling | VO_2_peak  HR Peak  O_2_ pulse  RER | RER ≥ 1.10  Exhaustion | No calibration reported  AE: NR  Predefined Safety Measures: NR | 17.8 mLˑkg^-1^ˑmin^-1^  73% pred |
| Khouri, 2014^32^ | Breast | Case-control study | Post treatment | Evaluate validity (e.g. VO_2_peak vs ISWT) | TM | NR | VO_2_peak  HR Peak  RER | RER ≥ 1.10  % of HR: NR  Exhaustion | No calibration reported  AE: NR  Predefined Safety Measures: NR | 24.5 mLˑkg^-1^ˑmin^-1^ |
| Kiecolt-Glaser, 2022 ^33^ | Breast | Non-randomized intervention study | Pre-treatment | Evaluated breast cancer survivors and inflammatory responses to a typhoid vaccine | CE | NR sec sampling | NR | NR | Predefined Safety Measures: NR AE: NR | 22 mLˑkg⁻¹ˑmin⁻¹ |
| Kim, 2006 ^34^ | Breast | Randomized controlled trial | During treatment | To evaluate the effect of an intervention | TM | NR sec sampling | HR VO₂ BP ECG | NR | Calibration: No Predefined Safety Measures: Yes AE: NR | Overall: NR  Intervention: 1672 mLˑˑmin^-1^  Control: 1597 mLˑmin^-1^ |
| Kirkham, 2020^35^ | Breast | Randomized controlled trial | During treatment | To evaluate the effect of an intervention | CE | NR | NR | NR | No calibration reported  AE: NR  Predefined Safety Measures: NR | NR |
| Klassen, 2014^36^ | Breast | Cross-sectional study | Pre-during-post treatment | 1) Pre-post treatment evaluation other than surgery  2) Evaluate acute toxicity | CE | 30 sec sampling | VO_2_peak  Anaerobic threshold  HR Peak  RER | RER > 1.1  % of pred HR+/- 10%  Exhaustion | Unclear calibration procedures  Testing-Related AE: Yes  Predefined Safety Measures: Yes | 16.8 mLˑkg^-1^ˑmin^-1^  85% pred |
| Koevoets, 2022 ^37^ | Breast | Randomized controlled trial | Post-treatment | To evaluate the effect of an intervention | CE | NR sec sampling | ECG VO₂ | NR | Calibration: No Predefined Safety Measures: NR AE: NR | NR |
| Koevoets, 2023 ^38^ | Breast | Randomized controlled trial | Pre-post treatment | To evaluate the effect of an intervention | NR | 30 sec sampling | HR ECG | NR | Calibration: No Predefined Safety Measures: NR AE: NR | NR |
| Lahart, 2018^39^ | Breast | Randomized controlled trial | Post treatment | Evaluate the effect of an intervention | TM | 20 sec sampling | VO_2_peak  HR Peak | NR | Calibration procedures described in detail  AE: NR  Predefined Safety Measures: NR | Overall: NR  Intervention:  Intervention: 24.1 mLˑkg^-1^ˑmin^-1^  Control: 26.6 mLˑkg^-1^ˑmin^-1^ |
| MacVicar, 1989^40^ | Breast | Randomized controlled trial | During treatment | Evaluate the effect of an intervention | CE | NR | VO_2_peak | Exhaustion | Unclear calibration procedures  AE: NR  Predefined Safety Measures: NR | Intervention: 1.02 L·min^-1^ |
| Madison, 2023 ^41^ | Breast | Prospective cohort study | During treatment; Post surgery, pre adjuvant the | Evaluate late toxicity > 1 year post-therapy | CE | NA sec sampling | NR | NR | Calibration: NA Predefined Safety Measures: Yes AE: NR | NR |
| Mehnerta, 2011^42^ | Breast | Randomized controlled trial | Post treatment | Evaluate the effect of an intervention | CE | NR | VO_2_peak | NR | No calibration reported  AE: NR  Predefined Safety Measures: NR | 26.5 mLˑkg^-1^ˑmin^-1^ |
| Moghadam, 2021 ^43^ | Breast | Randomized controlled trial | Post-treatment | To evaluate the effect of an intervention | CE | NR sec sampling | HR | NR | Calibration: No Predefined Safety Measures: Yes AE: NR | NR |
| Nuri, 2012^44^ | Breast | Randomized controlled trial | Post treatment | Evaluate the effect of an intervention | TM | NR | NR | NR | No calibration reported  AE: NR  Predefined Safety Measures: NR | Overall: NR  Intervention: 17.2 mLˑkg^-1^ˑmin^-1^  Control 14.5 mLˑkg^-1^ˑmin^-1^ |
| Ochi, 2022 ^45^ | Breast | Randomized controlled trial | Post-treatment | To evaluate the effect of an intervention | CE | NR sec sampling | VO₂ | Exhaustion | Calibration: No Predefined Safety Measures: NR AE: NR | Overall: NR  Intervention: 25.0 mLˑkg^-1^ˑmin^-1^  Control: 24.9 mLˑkg^-1^ˑmin^-1^ |
| Pagola, 2020 ^46^ | Breast | Randomized controlled trial | Post-treatment | To evaluate the effect of an intervention | CE | 20 sec sampling | AT HR VO₂ | Exhaustion | Calibration: No Predefined Safety Measures: NR AE: NR | NR |
| Peck, 2022 ^47^ | Breast | Prospective cohort study | Pre-during-post treatment | Evaluate acute toxicity < 1 year post-therapy | CE | 20 sec sampling | ECG VO₂ | Judgment by the technician Exhaustion | Calibration: No Predefined Safety Measures: NR AE: NR | Inactive: 16.2 mLˑkg^-1^ˑmin^-1^  Somewhat Active: 19.1 mLˑkg^-1^ˑmin^-1^  Highly Active: 23.6 mLˑkg^-1^ˑmin^-1^ |
| Samhan, 2021 | Breast | Randomized controlled trial | Post-treatment | To evaluate the effect of an intervention | TM | 30 sec sampling | HR BP VO₂ | Exhaustion | Calibration: Yes Predefined Safety Measures: NR AE: NR | Overall: NR  Intervention: 26.3 mLˑkg^-1^ˑmin^-1^  Control: 27.3 mLˑkg^-1^ˑmin^-1^ |
| Scharhag-Rosenberger, 2015^48^ | Breast | Prospective cohort study | Post treatment | Evaluate validity (e.g. VO_2_peak vs ISWT) | CE | 30 sec sampling | VO_2_peak  HR Peak  RER | Exhaustion | Calibration procedures described in detail  AE: NR  Predefined Safety Measures: NR | 22.6 mLˑkg^-1^ˑmin^-1^ |
| Scott, 2018^49^ | Breast | RCT | During treatment | To evaluate the effect of an intervention | TM | NR | VO_2_peak | Symptom limited | No calibration reported  AE: Yes (1, ECG abnormalities)  Predefined Safety Measures: No | 22.5 mLˑkg^-1^ˑmin^-1^  90% pred |
| Scott, 2023 ^50^ | Breast | Randomized controlled trial | During-post treatment | To evaluate the effect of an intervention | TM | NR sec sampling | HR ECG BP VO₂ VO_2_peak | NR | Calibration: No Predefined Safety Measures: Yes AE: NR | 24.9 mLˑkg⁻¹ˑmin⁻¹ |
| Siripanya, 2023 ^51^ | Breast | Randomized controlled trial | During treatment | To evaluate the effect of an intervention | TM | 30 sec sampling | HR RER VO₂ | RER Pred HR Exhaustion | Calibration: No Predefined Safety Measures: NR AE: NR | NR |
| Smoot, 2014^52^ | Breast | Cross-sectional study | During-post treatment | 1) Evaluate the effect of an intervention  2) To see if CPET affected arm volume | TM | NR | VO_2_peak  HR Peak  RPE | Voluntary termination | Unclear calibration procedures  AE: NR  Predefined Safety Measures: NR | 25.6 mLˑkg^-1^ˑmin^-1^ |
| Stalsberg, 2022 | Breast | Randomized controlled trial | Pre-treatment | Evaluate adherence to training | TM | 30 sec sampling | VO₂, VO_2_peak | NR | Calibration: No Predefined Safety Measures: NR AE: NR | 28.4 mLˑkg⁻¹ˑmin⁻¹ |
| Suesada, 2018^53^ | Breast | Prospective cohort study | Pre-post treatment | Evaluate accute toxiciy < 1-year post-therapy | CE | NR | VO_2_peak^1^ | NR | No calibration reported  AE: NR  Predefined Safety Measures: NR | 16.8 mLˑkg^-1^ˑmin^-1^ |
| Swisher, 2015^54^ | Breast | Randomized control trial | Post treatment | Evaluate the effect of an intervention | TM | NR | VO_2_peak | No | No calibration reported  AE: NR  Predefined Safety Measures: No | Overall: NR  Intervention: 19.4 mLˑkg^-1^ˑmin^-1^  Control 20.2 mLˑkg^-1^ˑmin^-1^ |
| Travier, 2015^55^ | Breast | Randomized controlled trial | During treatment | Evaluate the effect of an intervention | CE | NR | VO_2_peak  Anaerobic threshold  HR Peak | NR | No calibration reported  AE: NR  Predefined Safety Measures: NR | Overall: NR  Intervention: 23.9 mLˑkg^-1^ˑmin^-1^  Control: 23.8 mLˑkg^-1^ˑmin^-1^ |
| Tubiana-Mathieu, 2021 ^56^ | Breast | Prospective cohort study | During treatment | Method study (e.g. VO_2_peak vs ISWT) | CE | NR sec sampling | AT HR VO₂ | NR | Calibration: No Predefined Safety Measures: Yes AE: NR | NR |
| Uth, 2020 ^57^ | Breast | Randomized controlled trial | Post-treatment | To evaluate the effect of an intervention | CE | 30 sec sampling | HR RER VO₂ Wmax | Leveling off in VO₂ RER | Calibration: No Predefined Safety Measures: Yes AE: No | Overall: NR  Intervention: 28.5 mLˑkg^-1^ˑmin^-1^  Control 25.6 mLˑkg^-1^ˑmin^-1^  Intervention: 2.0 Lˑmin^-1^  Control 1.9 Lˑmin^-1^ |
| Vincent, 2020 ^58^ | Breast | Randomized controlled trial | During-post treatment | To evaluate the effect of an intervention | CE | NR sec sampling | HR VE; RER VO₂ | NR | Calibration: No Predefined Safety Measures: NR AE: NR | Overall: NR  Intervention: 20.8 mLˑkg^-1^ˑmin^-1^  Control 20.9 mLˑkg^-1^ˑmin^-1^ |
| Zvinovski, 2021 ^59^ | Breast | Single arm intervention study | Post-treatment | To evaluate the effect of an intervention | TM | NR sec sampling | HR RPE VO₂ | Leveling off in VO₂ | Calibration: NR Predefined Safety Measures: Yes AE: No | NR |
| Leensen, 2017^60^ | Breast and colon | Prospective cohort study | During-post treatment | Evaluate the effect of an intervention | CE | NR | VO_2_peak | NR | No calibration reported  AE: NR  Predefined Safety Measures: NR | 28.0 mLˑkg^-1^ˑmin^-1^ |
| Martin, 2015^61^ | Breast and prostate | Randomized controlled trial | Post treatment | To evaluate the effect of an intervention | CE | NR | NR | NR | No calibration reported  AE: NR  Predefined Safety Measures: NR | NR |
| Schneider, 2020^62^ | Breast and prostate | Prospective cohort study | Post treatment | Method study (e.g. VO2peak vs ISWT) | CE | NR | VO_2_peak | NR | Calibration procedures described in detail  AE: NR  Predefined Safety Measures: NR | 19.7 mLˑkg^-1^ˑmin^-1^ |
| Berkel, 2022 ^63^ | Colon/rectum | Randomized controlled trial | Pre-treatment | Pre surgery evaluation | CE | NR sec sampling | AT | NR | Calibration: Calibration procedures are mentioned, but not when and how Predefined Safety Measures: NR AE: NR | NR |
| Bolshinsky, 2022 ^64^ | Colon/rectum | Retrospective cohort study | Pre-treatment | Pre surgery evaluation | CE | NR sec sampling | AT HR VE VEVCO₂ RER ECG BP SpO₂ Wmax | Judgment by the technician Exhaustion | Calibration: No Predefined Safety Measures: Yes AE: NR | NR |
| Challand, 2012^65^ | Colon/rectum | Randomized controlled trial | Pre-treatment | 1) Pre surgery evaluation  2) Evaluate the effect of an intervention | CE | NR | Anaerobic threshold | NR | No calibration reported  AE: NR  Predefined Safety Measures: NR | NR |
| Chan, 2015 ^66^ | Colon/rectum | Retrospective cohort study | Pre-treatment | Pre surgery evaluation | CE | NR | VO_2_peak  Anaerobic threshold | NR | No calibration reported  AE: NR  Predefined Safety Measures: NR | Overall: NR  Planned CCU:  12.6 mLˑkg^-1^ˑmin^-1^  No planned CCU:  15.3 mLˑkg^-1^ˑmin^-1^ |
| Christensen, 2019^67^ | Colon/rectum | Randomized controlled trial | Post treatment | To evaluate the effect of an intervention | CE | NR | NR | NR | No calibration reported  AE: NR  Predefined Safety Measures: NR | Overall: 25.2 mLˑkg^-1^ˑmin^-1^ |
| Cramer, 2014^68^ | Colon/rectum | Prospective cohort study | Pre-during treatment | Evaluate acute toxicity < 1-year post-therapy | TM | NR | NR | NR | No calibration reported  AE: NR  Predefined Safety Measures: NR | Overall: NR  Treatment Naiive: 23.4 mLˑkg^-1^ˑmin^-1^  Chemotherapy: 20.4 mLˑkg^-1^ˑmin^-1^ |
| Devin, 2016^69^ | Colon/rectum | Randomized controlled trial | Post treatment | Evaluate the effect of an intervention | CE | 15 sec sampling | VO_2_peak | NR | Calibration procedures described in detail  AE: Yes  Predefined Safety Measures: Yes | Overall: NR  Intervention: 22.8 mLˑkg^-1^ˑmin^-1^  Control: 21.5 mLˑkg^-1^ˑmin^-1^ |
| Exarchou, 2020 ^70^ | Colon/rectum | Retrospective cohort study | Pre-treatment | Pre surgery evaluation | CE | 30 sec sampling | AT HR O₂-pulse VE VEVCO₂ RER ECG BP SpO₂ Wmax | Voluntary termination | Calibration: No Predefined Safety Measures: NR AE: NR | Peak: NR  AT Open: 9.0 mLˑkg⁻¹ˑmin⁻¹  AT Laproscopic: 9.0 mLˑkg⁻¹ˑmin⁻¹ |
| Franssen, 2023 ^71^ | Colon/rectum | Prospective cohort study | Pre-treatment | Pre surgery evaluation | CE | 10 sec sampling | AT/VT HR O₂-pulse VE VEVCO₂ RER | Voluntary termination | Calibration: Calibration procedures are mentioned, but not when and how Predefined Safety Measures: Yes AE: NR | 18.4 mLˑkg⁻¹ˑmin⁻¹ |
| Heitkamp, 2023 ^72^ | Colon/rectum | Non-randomized intervention study | Post-treatment | To evaluate the effect of an intervention | CE | NR sec sampling | HR RER Wmax VO₂peak | RER Exhaustion | Calibration: No Predefined Safety Measures: NR AE: NR | 22 mLˑkg⁻¹ˑmin⁻¹ |
| Hossain, 2020^73^ | Colon/rectum | Randomized controlled trial | Pre-post treatment | To evaluate the effect of an intervention | CE | NR | NR | NR | No calibration reported  AE: NR  Predefined Safety Measures: NR | Peak: NR  AT EPA: 16.7 mLˑkg⁻¹ˑmin⁻¹  AT Placebo: 15.1 mLˑkg⁻¹ˑmin⁻¹ |
| Lee, 2013^74^ | Colon/rectum | Retrospective cohort study | Pre-treatment | Pre surgery evaluation | CE | 20 sec sampling | NR | NR | No calibration reported  AE: NR  Predefined Safety Measures: NR | NR |
| Loughney, 2021 ^75^ | Colon/rectum | Randomized controlled trial | Pre-during treatment | To evaluate the effect of an intervention | CE | NR sec sampling | AT/VT | NR | Calibration: No Predefined Safety Measures: NR AE: NR | Peak: NR  AT intervention: 11.6 mLˑkg⁻¹ˑmin⁻¹  AT control: 10.8 mLˑkg⁻¹ˑmin⁻¹ |
| Minnella, 2020 ^76^ | Colon/rectum | Randomized controlled trial | Pre-treatment | To evaluate the effect of an intervention | CE | 20 sec sampling | AT/VT HR VE VEVCO₂ RER ECG BP SpO₂ Wmax VO₂ | RER | Calibration: Yes Predefined Safety Measures: NR AE: NR | Peak: NR  AT intervention: 12.3 mLˑkg⁻¹ˑmin⁻¹  AT control: 13.8 mLˑkg⁻¹ˑmin⁻¹ |
| Morielli, 2021 ^77^ | Colon/rectum | Randomized controlled trial | During treatment | To evaluate the effect of an intervention | TM | NR sec sampling | VE | NR | Calibration: No Predefined Safety Measures: NR AE: NR | Overall: NR  Intervention: 27.3 mLˑkg^-1^ˑmin^-1^  Control: 29.6 mLˑkg^-1^ˑmin^-1^ |
| Pinto, 2013^78^ | Colon/rectum | Randomized controlled trial | Post treatment | Evaluate the effect of an intervention | TM | NR | VO_2_peak | NR | Calibration procedures not available  AE: NR  Predefined Safety Measures: No | Overall: NR  Intervention: 21.9 mLˑkg^-1^ˑmin^-1^  Control: 23.8 mLˑkg^-1^ˑmin^-1^ |
| Rose, 2018^79^ | Colon/rectum | Retrospective cohort study | Pre-treatment | Pre surgery evaluation | CE | NR | VO_2_peak  Anaerobic threshold  HR Peak  O_2_ pulse  VEVCO_2_  RER | Voluntary termination | Calibration procedures described in detail  AE: NR  Predefined Safety Measures: NR | 16.3 mLˑkg^-1^ˑmin^-1^ |
| Sellar, 2014^80^ | Colon/rectum | Prospective cohort study | Post treatment | To evaluate the effect of an intervention | CE | NR | VO_2_peak | NR | No calibration reported  AE: No  Predefined Safety Measures: NR | 22.5 mLˑkg-1ˑmin-1  1.86 L·min-1 |
| West, 2014^81^ | Colon/rectum | Prospective cohort study | Pre-treatment | Pre surgery evaluation | CE | 30 sec sampling | VO_2_peak  Anaerobic threshold  VEVCO_2_ | Voluntary termination | No calibration reported  Testing-Related AE: No  Predefined Safety Measures: NR | 15.8 mLˑkg^-1^ˑmin^-1^  NR |
| West, 2014^82^ | Colon/rectum | Prospective cohort study | Pre-post treatment | Pre-post surgery evaluation | CE | NR | VO_2_peak  Anaerobic threshold  HR Peak  O_2_ pulse  VEVCO_2_ | Voluntary termination | No calibration reported  AE: NR  Predefined Safety Measures: NR | 18.1 mLˑkg^-1^ˑmin^-1^ |
| West, 2014^83^ | Colon/rectum | Prospective cohort study | Pre-treatment | Pre surgery evaluation | CE | NR | VO_2_peak  O_2_ pulse  VEVCO_2_ | NR | No calibration reported  AE: NR  Predefined Safety Measures: Yes | 18.9 mLˑkg^-1^ˑmin^-1^ |
| Wilson, 2019^84^ | Colon/rectum | Retrospective cohort study | Pre-treatment | Pre surgery evaluation | CE | NR | Anaerobic threshold  VEVCO_2_ | NA | No calibration reported  AE: NR  Predefined Safety Measures: No | AT: 11.2 mLˑkg^-1^ˑmin^-1^  VEVCO_2_: 33 |
| Tufo, 2018^85^ | Colon/rectum and liver | Prospective cohort study | Pre-post treatment | Pre surgery evaluation | NR | NR | VO_2_peak  Anaerobic threshold | NR | No calibration reported  AE: NR  Predefined Safety Measures: NR | Overall: NR  AT: 70-79 years:  17.5 mLˑkg^-1^ˑmin^-1^  AT: >80 years:  15.8 mLˑkg^-1^ˑmin^-1^ |
| Astrup Søndergaard, 2022 ^86^ | Esophageal | Prospective cohort study | Pre-post treatment | Evaluate acute toxicity < 1 year post-therapy | CE | NR sec sampling | HR RER ECG BP SpO₂ Wmax VO₂peak | RER Exhaustion | Calibration: No Predefined Safety Measures: NR AE: NR | 21.2 mLˑkg⁻¹ˑmin⁻¹ 87.5% pred |
| Benington, 2019^87^ | Esophageal | Retrospective cohort study | Pre-treatment | Pre surgery evaluation | CE | NR | VO_2_peak  Anaerobic threshold  VEVCO_2_ | RER>1.15  >80% of age pred HR | No calibration reported  AE: NR  Predefined Safety Measures: NR | 19.1 mLˑkg^-1^ˑmin^-1^ |
| Chang, 2020^88^ | Esophageal | Randomized controlled trial | Post treatment | To evaluate the effect of an intervention | CE | NR | VO_2_peak | NR | No calibration reported  AE: NR  Predefined Safety Measures: NR | 15.41 mLˑkg^-1^ˑmin^-1^ |
| Drummond, 2018^89^ | Esophageal | Prospective cohort study | Pre-post treatment | Pre-post treatment evaluation other than surgery | CE | NR | VO_2_peak | Exhaustion | No calibration reported  AE: NR  Predefined Safety Measures: Yes | 17.1 mLˑkg^-1^ˑmin^-1^ |
| Forshaw, 2008^90^ | Esophageal | Prospective cohort study | Pre-treatment | Pre surgery evaluation | CE | NR | VO_2_peak  Anaerobic threshold | NR | No calibration reported  AE: NR  Predefined Safety Measures: Yes | 20.5 mLˑkg^-1^ˑmin^-1^  83% pred |
| Jack, 2014 ^91^ | Esophageal | Prospective cohort study | Pre-during treatment | Pre-post treatment evaluation other than surgery (e.g. effect of drugs/radiation) | CE | NR sec sampling | VO₂ HR ECG BP SpO₂ Wmax | Voluntary termination | Calibration: No Predefined Safety Measures: Yes AE: NR | 20.8 mLˑkg⁻¹ˑmin⁻¹ 1.61 Lˑmin⁻¹ |
| Lam, 2019^92^ | Esophageal | Retrospective cohort study | Pre-treatment | Pre surgery evaluation | CE | 30 sec sampling | VO_2_peak  Anaerobic threshold | Voluntary termination | No calibration reported  AE: NR  Predefined Safety Measures: Yes | 21.1 mLˑkg^-1^ˑmin^-1^ |
| Moyes, 2013^93^ | Esophageal | Retrospective cohort study | Pre-treatment | Pre surgery evaluation | CE | NR | VO_2_peak  Anaerobic threshold | Development of symptoms  Exhaustion | No calibration reported  AE: NR  Predefined Safety Measures: Yes | 15.2 mLˑkg^-1^ˑmin^-1^ |
| Navidi, 2018 ^94^ | Esophageal | Prospective cohort study | Pre-post treatment | Evaluate acute toxicity < 1 year post-therapy | CE | 30 sec sampling | AT HR VEVCO₂ RER ECG BP SpO₂, VO₂peak | Judgment by the technician Exhaustion Voluntary termination | Calibration: Yes Predefined Safety Measures: Yes AE: NR | 21.7 mLˑkg⁻¹ˑmin⁻¹ |
| O'Neill, 2018^95^ | Esophageal | Randomized controlled trial | Post treatment | To evaluate the effect of an intervention | CE | NR | NR | NR | No calibration reported  AE: Yes (1 AE, atrial fibrillation)  Predefined Safety Measures: NR | Overall: NR  Intervention: 18.7 mLˑkg^-1^ˑmin^-1^  Control: 21.8 mLˑkg^-1^ˑmin |
| Ozova, 2022 ^96^ | Esophageal | Prospective cohort study | Pre-treatment | Pre surgery evaluation | TM | NR sec sampling | AT O₂-pulse VE VEVCO₂ VCO₂ VO₂ | NR | Calibration: No Predefined Safety Measures: Yes AE: NR | Overall: NR  Intervention: 15.8 mLˑkg^-1^ˑmin^-1^  Control: 18.5 mLˑkg^-1^ˑmin  % predicted: 79%  % predicted: 82 |
| Patel, 2019^97^ | Esophageal | Prospective cohort study | Pre-treatment | Pre surgery evaluation | CE | NR | VO_2_peak | NR | Calibration procedures described in detail  AE: NR  Predefined Safety Measures: NR | 18.2 mLˑkg^-1^ˑmin^-1^ (median) |
| Sinclair, 2017^98^ | Esophageal | Prospective cohort study | Pre-treatment | Pre surgery evaluation | CE | NR | VO_2_peak  VEVCO_2_ | Symptom-limited | No calibration reported  AE: NR  Predefined Safety Measures: NR | 18.8 mLˑkg^-1^ˑmin^-1^ |
| Thomson, 2018^99^ | Esophageal | Prospective cohort study | Pre-treatment | Pre-post treatment evaluation other than surgery | CE | NR | VO_2_peak  Anaerobic threshold  VEVCO_2_ | Exhaustion | No calibration reported  AE: NR  Predefined Safety Measures: NR | 16.6 mLˑkg^-1^ˑmin^-1^ |
| van Vulpen, 2021 ^100^ | Esophageal | Randomized controlled trial | Post-treatment | To evaluate the effect of an intervention | CE | 30 sec sampling | AT/VT HR Wmax RCP | Exhaustion | Calibration: No Predefined Safety Measures: Yes AE: NR | Overall: NR  Intervention: 22.6mLˑkg^-1^ˑmin^-1^  Control: 22.4 mLˑkg^-1^ˑmin |
| West, 2021 ^101^ | Esophago-gastric | Prospective cohort study | Pre-post treatment | Pre-post treatment evaluation other than surgery (e.g. effect of drugs/radiation) | NR | NR sec sampling | AT/VT VO₂ | NR | Calibration: No Predefined Safety Measures: NR AE: NR | Overall: NR  Male:22.7 mLˑkg^-1^ˑmin^-1^  Female: 16.8 mLˑkg^-1^ˑmin |
| Hyltander, 2005^102^ | Esophagus, stomach and pancreas | Randomized controlled trial | Pre-post treatment | Evaluate the effect of an intervention | TM | NR | VO_2_peak | NR | No calibration reported  AE: NR  Predefined Safety Measures: NR | Overall: NR  Intervention 1: 1.88 L·min^-1^  Intervention 2: 1.79 L·min^-1^  Control: 2.01 L·min^-1^ |
| Cho, 2018^103^ | Gastric | Prospective cohort study | Pre-treatment | To evaluate the effect of an intervention | TM | NR | VO_2_peak | NR | No calibration reported  AE: No  Predefined Safety Measures: NR | 35.1 mLˑkg^-1^ˑmin |
| Sinclair, 2016^104^ | Gastric | Retrospective cohort study | Pre-post treatment | Pre-post treatment evaluation other than surgery | CE | NR | VO_2_peak  Anaerobic threshold  VEVCO_2_ | Symptom limited | No calibration reported  AE: NR  Predefined Safety Measures: NR | 16.8 mLˑkg^-1^ˑmin^-1^  93% pred |
| Lindman, 2021 ^105^ | Hematological | One-armed intervention study | Pre-during treatment | To evaluate the effect of an intervention | CE | 15 sec sampling | RER VO₂ ECG BP, | RER | Calibration: Yes Predefined Safety Measures: Yes AE: NR | Overall: NR  Male: 1.5 L·min^-1^  Female: 1.09 L·min^-1^ |
| van Wijk, 2021 ^106^ | Hepatobiliary | Prospective cohort study | Pre-treatment | To evaluate the effect of an intervention | NR | NR sec sampling | AT/VT | NR | Calibration: NR Predefined Safety Measures: NR AE: NR | NR |
| Caru, 2019^107^ | Leukemia | Case-control study | Post treatment | Evaluate late toxicity > 1-year post-therapy | CE | NR | VO_2_peak | NR | Unclear calibration procedures  AE: NR  Predefined safety measures: NR | 32.4 mLˑkg^-1^ˑmin^-1^  77% pred |
| Jarvela, 2010^108^ | Leukemia | Case-control study | Post treatment | 1) Evaluate late toxicity > 1-year post-therapy  2) To evaluate physical fitness | CE | 60 sec sampling | VO_2_peak  HR Peak  RER | RER > 1.10  Exhaustion | No calibration reported  AE: NR  Predefined Safety Measures: NR | 34.8 mLˑkg^-1^ˑmin^-1^ |
| Labonte, 2020^109^ | Leukemia | Cross-sectional study | Post treatment | Method study (e.g. VO2peak vs ISWT) | CE | NR | VO_2_peak | NR | No calibration reported  AE: NR  Predefined Safety Measures: NR | 32.1 mLˑkg^-1^ˑmin^-1^ |
| Lemay, 2020^110^ | Leukemia | Prospective cohort study | Post treatment | Evaluate late toxicity > 1-year post-therapy | CE | NR | VO_2_peak | NR | Unclear calibration procedures  AE: NR  Predefined Safety Measures: NR | 30.7 mLˑkg^-1^ˑmin^-1^  84% pred |
| Long, 2020^111^ | Leukemia | Case-control study | Post treatment | Evaluate late toxicity > 1-year post-therapy | TM | NR | VO_2_peak | NR | Unclear calibration procedures  AE: NR  Predefined Safety Measures: NR | 36.78 mLˑkg^-1^ˑmin^-1^  2.64 L/min |
| Myrdal, 2018^112^ | Leukemia | Cross-sectional study | Post treatment | Evaluate late toxicity > 1-year post-therapy | CE | NR | VO_2_peak  HR Peak  RER | RER > 1.10  Exhaustion | Unclear calibration procedures  AE: NR  Predefined Safety Measures: NR | 35.0 mLˑkg^-1^ˑmin^-1^  85% pred |
| Myrdal, 2020^113^ | Leukemia | Cross-sectional study | Post treatment | Evaluate late toxicity > 1-year post-therapy | TM | NR | VO_2_peak | NR | Calibration procedures described in detail  AE: NR  Predefined Safety Measures: NR | 36.4 mLˑkg^-1^ˑmin^-1^ (median)  89% pred |
| Phillips, 2020^114^ | Leukemia | Case-control study | Post treatment | Evaluate late toxicity > 1-year post-therapy | TM | NR | NR | NR | No calibration reported  AE: NR  Predefined Safety Measures: NR | 85% predicted HR: 23.5 mLˑkg^-1^ˑmin^-1^ |
| Tonorezos, 2013^115^ | Leukemia | Case-control study | Post treatment | Evaluate late toxicity > 1-year post-therapy | TM | NR | VO_2_peak | Exhaustion  Volitional fatigue | No calibration reported  AE: NR  Predefined Safety Measures: NR | 30.7 mLˑkg^-1^ˑmin^-1^ |
| Yeon, 2022 ^116^ | Leukemia | Retrospective cohort study | Pre-treatment | Survival | TM | 15 sec sampling | HR RER ECG VO₂ AT | RER | Calibration: No Predefined Safety Measures: Yes AE: NR | NR |
| Armenian, 2017^117^ | Leukemia and lymphoma | Retrospective cohort study | Post treatment | 1) Diagnostic  2) To evaluate CPET feasibility | CE | 30 sec sampling | VO_2_peak  HR Peak | Leveling off in VO_2_  RER ≥ 1.10  Voluntary termination | No calibration reported  AE: No  Predefined Safety Measures: Yes | 19.5 mLˑkg^-1^ˑmin^-1^  78% pred |
| Bayram, 2024^118^ | Leukemia and lymphoma | Randomized controlled trial | Pre-post treatment | To evaluate the effect of an intervention | TM | NR sec sampling | HR VEVCO₂ RER ECG BP SpO₂ | RER Pred HR Exhaustion | Calibration: No Predefined Safety Measures: NR AE: NR | Overall: NR  Intervention: 22.9 mLˑkg^-1^ˑmin^-1^  Control: 22.8 mLˑkg^-1^ˑmin^-1^  Intervention: 79% pred  Control: 88% pred |
| Ishikawa, 2019 ^119^ | Leukemia and lymphoma | Retrospective cohort study | Pre-post treatment | Evaluate acute toxicity < 1 year post-therapy | CE | NR sec sampling | NR | Leveling off in VO₂ % of pred heart rate Exhaustion | Calibration: No Predefined Safety Measures: NR AE: NR | 22.5 mLˑkg⁻¹ˑmin⁻¹ |
| Kirsten, 2021 ^120^ | Leukemia and lymphoma | Prospective cohort study | Pre-treatment | Survival | CE | 30 sec sampling | HR RER ECG Wmax/kg | RER Exhaustion | Calibration: No Predefined Safety Measures: NR AE: NR | Overall: 17.6 mLˑkg^-1^ˑmin^-1^  Overall: 72% |
| Dunne, 2016^121^ | Liver | Randomized controlled trial | Pre-treatment | Evaluate the effect of an intervention | CE | 30 sec sampling | VO_2_peak | NR | No calibration reported  AE: NR  Predefined Safety Measures: Yes | Overall: NR  Intervention: 16.1 mLˑkg^-1^ˑmin^-1^  Control: 15.7 mLˑkg^-1^ˑmin^-1^ |
| Kaibori, 2013^122^ | Liver | Prospective cohort study | Pre-treatment | Pre surgery evaluation | CE | Highest value | NR | Fatigue, pain or headache, or failure to maintain a speed greater than 40 rpm for more than 30 seconds | No calibration reported  AE: NR  Predefined Safety Measures: No | NR |
| Kaibori, 2019^123^ | Liver | Retrospective cohort study | Pre-post treatment | Pre-post surgery evaluation (effect of surgery) | CE | NR | NR | NR | No calibration reported  AE: NR  Predefined Safety Measures: NR | peak: NR  Intervention AT: 12.0 mLˑkg^-1^ˑmin^-1^  Control AT: 11.9 mLˑkg^-1^ˑmin^-1^ |
| Batchelor, 2015^124^ | Lung | Retrospective cohort study | Pre-treatment | 2) Evaluate VO_2_peak Thoracoscore vs outcome after surgery | CE | NR | VO_2_peak | Maximum symptom limited | No calibration reported  AE: NR  Predefined Safety Measures: NR | Overall: NR  Surgery:  14.2 mLˑkg^-1^ˑmin^-1^  Not surgery:  10.1 mLˑkg^-1^ˑmin^-1^ |
| Beccaria, 2001^125^ | Lung | Prospective cohort study | Pre-post treatment | Pre-post surgery evaluation (effect of surgery) | CE | 10 sec sampling | NR | NR | No calibration reported  AE: NR  Predefined Safety Measures: NR | NR |
| Bechard, 1987^126^ | Lung | Prospective cohort study | Pre-treatment | Evaluate acute toxicity < 1-year post-therapy | CE | NR | NR | NR | Calibration procedures described in detail  AE: NR  Predefined Safety Measures: Yes | Overall: NR  Individual patient VO_2_peak reported |
| Begum, 2016^127^ | Lung | Prospective cohort study | Pre-post treatment | Pre surgery evaluation | NR | NR | VO_2_peak | NR | No calibration reported  AE: NR  Predefined Safety Measures: NR | 17.4 mLˑkg^-1^ˑmin^-1^ |
| Bhatia, 2019^128^ | Lung | Randomized controlled trial | Pre-treatment | To evaluate the effect of an intervention | CE | NR | NR | NR | No calibration reported  Testing Related AE: No  Predefined Safety Measures: Yes | Overall: NR  Intervention: 19.9 mLˑkg^-1^ˑmin^-1^  Control: 20.4 mLˑkg^-1^ˑmin^-1^ |
| Bobbio, 2009^129^ | Lung | Prospective cohort study | Pre-post treatment | Pre surgery evaluation | CE | NR | VO_2_peak  HR Peak  O_2_ pulse  VEVCO2 | NR | No calibration reported  AE: NR  Predefined Safety Measures: NR | 18.7 mLˑkg^-1^ˑmin^-1^  69% pred |
| Bolliger, 1995^130^ | Lung | Prospective cohort study | Pre-post treatment | Pre-post surgery evaluation | CE | NR | VO_2_peak | Leveling off in VO2  Exhaustion | No calibration reported  AE: NR  Predefined Safety Measures: NR | Overall: NR  Complications:  15.2 mLˑkg^-1^ˑmin^-1^  No complications:  18.6 mLˑkg^-1^ˑmin^-1^  NR  85% pred |
| Boujibar, 2018^131^ | Lung | Retrospective cohort study | Pre-treatment | 1) Pre surgery evaluation  2) Evaluate the effect of an intervention | CE | NR | VO_2_peak | Exhaustion | No calibration reported  AE: NR  Predefined Safety Measures: NR | 15.4 mLˑkg^-1^ˑmin^-1^ |
| Brunelli, 2009^132^ | Lung | Prospective cohort study | Pre-treatment | Pre surgery evaluation | CE | 30 sec sampling | NR | Symptom limited | No calibration reported  AE: NR  Predefined Safety Measures: NR | Overall: NR  Major resection: 15.9 mLˑkg^-1^ˑmin^-1^  Minor resection: 14.4 mLˑkg^-1^ˑmin^-1^  Major resection: 62% pred  Minor resection: 57% pred |
| Brunelli, 2014^133^ | Lung | Prospective cohort study | Pre-treatment | Pre surgery evaluation | CE | 30 sec sampling | VO_2_peak | NR | No calibration reported  AE: NR  Predefined Safety Measures: NR | 16.1 mLˑkg^-1^ˑmin^-1^ |
| Brutsche, 2000^134^ | Lung | Prospective cohort study | Pre-treatment | Pre surgery evaluation | CE | NR | VO_2_peak Ventilation | Exhaustion | No calibration reported  AE: NR  Predefined Safety Measures: NR | 21.1 mLˑkg^-1^ˑmin^-1^  94% pred |
| Campione, 2010^135^ | Lung | Retrospective cohort study | Pre-treatment | Pre surgery evaluation | CE | NR | VO_2_peak  HR Peak  O_2_ pulse  Ventilation  VEVCO_2_ | RER > 1.00  Exhaustion | Calibration procedures described in detail  AE: NR  Predefined Safety Measures: NR | 11.1 mLˑkg^-1^ˑmin^-1^  NR |
| Cavalheri, 2016^136^ | Lung | Cross-sectional | Post-treatment | Evaluate validity (e.g. VO_2_peak vs ISWT) | CE | 20 sec sampling | VO_2_peak mLˑkg^-1^ˑmin^-1^ VO_2_peak L·min^-1^  VO_2_peak in % of pred HR Peak  O_2_ pulse | RPE | No calibration reported  AE: NR  Predefined Safety Measures: Yes | 15.0 mLˑkg^-1^ˑmin^-1^  1.03 L·min^-1^  63% pred |
| Chao, 2022 ^16^ | Lung | Retrospective cohort study | Pre-treatment | Pre surgery evaluation | CE | NR sec sampling | AT/VT VEVCO₂ VO₂ BP | NR | Calibration: No Predefined Safety Measures: Yes AE: No | NR |
| Chouinard, 2022 ^137^ | Lung | Retrospective cohort study | Pre-treatment | Pre surgery evaluation | CE | NR sec sampling | HR Ventilation Work rate SpO₂ BR VO₂peak | RER Pred HR RPE/BORG | Calibration: No Predefined Safety Measures: Yes AE: NR | 18.8 mLˑkg⁻¹ˑmin⁻¹ 100.6% pred |
| Colman, 1982^138^ | Lung | Prospective cohort study | Pre-during treatment | Pre surgery evaluation | CE | NR | NR | NR | No calibration reported  AE: NR  Predefined Safety Measures: NR | NR |
| Dun, 2023 ^139^ | Lung | Prospective cohort study | Pre-treatment | Pre surgery evaluation; Survival | CE | NR sec sampling | HR ECG BP VO₂peak | Leveling off in VO₂ RER Pred HR Exhaustion NR | Calibration: No Predefined Safety Measures: Yes AE: Yes | 22.9 mLˑkg⁻¹ˑmin⁻¹ |
| Edvardsen, 2015^140^ | Lung | Prospective cohort study | Pre-post treatment | Pre-post surgery evaluation (effect of surgery) | TM | NR | VO_2_peak  RER | RER  Blood lactate  Exhaustion | No calibration reported  AE: NR  Predefined Safety Measures: NR | 23.9 mLˑkg^-1^ˑmin^-1^  NR  81% pred |
| Edvardsen, 2015^141^ | Lung | Randomized controlled trial | Pre-post treatment | Evaluate the effect of an intervention | TM | NR | VO_2_peak | Exhaustion | No calibration reported  AE: NR  Predefined Safety Measures: NR | Overall: NR  Intervention: 19.2 mLˑkg^-1^ˑmin^-1^  Control: 18.1 mLˑkg^-1^ˑmin^-1^ |
| Epstein, 1995^142^ | Lung | Prospective cohort study | Pre-treatment | Pre surgery evaluation | CE | NR | NR | symptom-limited | No calibration reported  AE: NR  Predefined Safety Measures: NR | NR |
| Fang, 2014^143^ | Lung | Prospective cohort study | Pre-treatment | Pre surgery evaluation | CE | NR | VO_2_peak | NR | No calibration reported  AE: NR  Predefined Safety Measures: Yes | 23.1 mLˑkg^-1^ˑmin^-1^  ---  75% pred |
| Fernandez-Rodrıguez, 2018^144^ | Lung | Prospective cohort study | Pre-post treatment | Pre-post surgery evaluation (effect of surgery) | CE | NR | VO_2_peak | NR | No calibration reported  AE: NR  Predefined Safety Measures: NR | 18.8 mLˑkg^-1^ˑmin^-1^  80% pred |
| Fresard, 2016^145^ | Lung | Prospective cohort study | Pre-treatment | Evaluate the effect of an intervention | CE | NR | VO_2_peak  Anaerobic threshold  HR Peak  O_2_ pulse  Ventilation  VEVCO_2_ | RER > 1.2  % of pred HR > 80% | Calibration procedures described in detail  AE: NR  Predefined Safety Measures: NR | Overall: NR  NAC+: 16.4 mLˑkg^-1^ˑmin^-1^  NAC-: 20.5 mLˑkg^-1^ˑmin^-1^ |
| Gao, 2024 ^146^ | Lung | Randomized controlled trial | Post-treatment | To evaluate the effect of an intervention | NR | NR sec sampling | NR | NR | Calibration: No Predefined Safety Measures: NR AE: NR | Overall: NR  Intervention: 11.6 mLˑkg^-1^ˑmin^-1^  Control: 11.2 mLˑkg^-1^ˑmin^-1^ |
| Granger, 2015^147^ | Lung | Prospective cohort study | Pre-post treatment | Evaluate validity (e.g. VO_2_peak vs ISWT) | CE | 30 sec sampling | VO_2_peak  HR Peak  Ventilation | % of pred HR: NR  Breathlessness | No calibration reported  AE: NR  Predefined Safety Measures: NR | 17.8 mLˑkg^-1^ˑmin^-1^  76% pred |
| Gravier, 2020 ^148^ | Lung | Retrospective cohort study | Pre-treatment | Pre surgery evaluation | CE | Average of 3-5 breath sec sampling | AT/VT HR VE VEVCO₂ RER ECG Wmax | Leveling off in VO₂ RER Pred HR RPE/BORG | Calibration: No Predefined Safety Measures: Yes AE: Yes | 14.6 mLˑkg^-1^ˑmin^-1^  63% pred |
| Gravier, 2022 ^149^ | Lung | Randomized controlled trial | Pre-treatment | To evaluate the effect of an intervention | CE | NR sec sampling | AT/VT HR VEVCO₂ ECG Wmax VO₂peak | NR | Calibration: No Predefined Safety Measures: NR AE: NR | 64.0% pred |
| Hwang, 2012^150^ | Lung | Randomized controlled trial | During treatment | Evaluate the effect of an intervention | CE | 30 sec sampling | VO_2_peak  HR Peak  RER | NR | No calibration reported  AE: NR  Predefined Safety Measures: NR | Intervention:15.1 mLˑkg^-1^ˑmin^-1^  Control:16.7 mLˑkg^-1^ˑmin^-1^  49% pred |
| Janssen, 2017^151^ | Lung | One-armed intervention study | Post treatment | Evaluate the effect of an intervention | CE | NR | VO_2_peak | NR | No calibration reported  AE: NR  Predefined Safety Measures: NR | 1.28 L·min^-1^  72% pred |
| Jones, 2007^152^ | Lung | Single arm intervention | Pre-treatment | Evaluate the effect of an intervention | CE | 30 sec sampling | VO_2_peak  Ventilation  RER | Symptom limited | No calibration reported  Testing Related AE: No  Predefined Safety Measures: yes | 15.7 mLˑkg^-1^ˑmin^-1^  NR  70% pred |
| Jones, 2010^153^ | Lung | Prospective cohort study | Pre-treatment | Pre surgery evaluation | CE | 30 sec sampling | VO_2_peak  HR peak  Ventilation | NR | No calibration reported  AE: NR  Predefined Safety Measures: NR | 15.8 mLˑkg^-1^ˑmin^-1^  1.18 L·min^-1^  64% pred |
| Kasikcioglu, 2009^154^ | Lung | Prospective cohort study | Pre-treatment | Pre surgery evaluation | TM | 10 sec sampling | VO_2_peak | RER > 1.1  Voluntary termination | No calibration reported  AE: Yes  Predefined Safety Measures: NR | 21.4 mLˑkg^-1^ˑmin^-1^  ---  --- |
| Kristenson, 2024 ^155^ | Lung | Retrospective cohort study | Pre-treatment | Pre surgery evaluation | CE | 10 sec sampling | HR VE; VEVCO₂ RER ECG BP Borg VO₂peak | RPE/BORG Judgment by the technician Exhaustion | Calibration: Yes Predefined Safety Measures: NR AE: NR | 18 mLˑkg⁻¹ˑmin⁻¹ |
| Kushibe, 2008^156^ | Lung | Retrospective cohort study | Pre-post treatment | Pre-post surgery evaluation | CE | 15 sec sampling | VO_2_peak | Until dyspnea or leg discomfort | No calibration reported  AE: NR  Predefined Safety Measures: Yes | 19.3 mLˑkg^-1^ˑmin^-1^ |
| Kushibe, 2008^157^ | Lung | Retrospective cohort study | Pre-post treatment | Pre-post surgery evaluation | CE | 15 sec sampling | VO_2_peak | Until dyspnea or leg discomfort | No calibration reported  AE: NR  Predefined Safety Measures: NR | Right upper lobe:19.3 mLˑkg^-1^ˑmin^-1^  Left upper lobe: 20.2 mLˑkg^-1^ˑmin^-1^  Right lower lobe: 20.1 mLˑkg^-1^ˑmin^-1^  Left lower lobe: 17.6 mLˑkg^-1^ˑmin^-1^ |
| Larsen, 1997^158^ | Lung | Prospective cohort study | Pre-post treatment | Pre-post surgery evaluation | CE | Average of eight breaths | VO_2_peak  Ventilation | RER > 1.09  Exhaustion | Calibration procedures described in detail  AE: NR  Predefined Safety Measures: NR | Pneumonectomy: 80% pred  Lobectomy: 88% pred |
| Larsen, 1997^159^ | Lung | Prospective cohort study | Pre-treatment | Pre surgery evaluation | CE | Average of eight breaths | VO_2_peak | Leveling off in VO_2_  RER > 1.09 | Calibration procedures described in detail  AE: NR  Predefined Safety Measures: NR | NR  1.38 L·min^-1^  83% pred |
| Licker, 2011^160^ | Lung | Prospective cohort study | Pre-treatment | Pre surgery evaluation | TM | NR | NR | Exhaustion | No calibration reported  AE: NR  Predefined Safety Measures: NR | NR |
| Lindenmann, 2020^161^ | Lung | Retrospective cohort study | Pre-treatment | Pre surgery evaluation | CE | NR | VO_2_peak | NR | No calibration reported  AE: NR  Predefined Safety Measures: NR | 18.3 mLˑkg^-1^ˑmin^-1^  65% pred |
| Loewen, 2007^162^ | Lung | Prospective cohort study | Pre-treatment | Pre surgery evaluation | CE | NR | VO_2_peak | Symptom-limited | No calibration reported  AE: NR  Predefined Safety Measures: NR | Overall: NR  Low Risk: 17.0 mLˑkg^-1^ˑmin^-1^  76% pred  High Risk: 17.9 mLˑkg^-1^ˑmin^-1^  73% pred  Very High Risk:  12.0 mLˑkg^-1^ˑmin^-1^  57% pred  No surgery: 14.4 mLˑkg^-1^ˑmin^-1^  60% pred |
| Marhic, 2019^163^ | Lung | Non-randomized intervention study | Pre-post treatment | To evaluate the effect of an intervention | NR | NR | NR | NR | No calibration reported  AE: NR  Predefined Safety Measures: NR | 42% pred |
| Markos, 1989^164^ | Lung | Prospective cohort study | Pre-treatment | Pre-post surgery evaluation | CE | NR | VO_2_peak | NR | No calibration reported  AE: NR  Predefined Safety Measures: NR | Overall: NR  No complications:  17.5 mLˑkg^-1^ˑmin^-1^  70% pred  Complications:  16.8 mLˑkg^-1^ˑmin^-1^  75% pred |
| Marulli, 2010^165^ | Lung | Prospective cohort study | Pre-post treatment | Pre-post treatment evaluation other than surgery | CE | NR | NR | Exhaustion | No calibration reported  AE: NR  Predefined Safety Measures: NR | 1.76 Lˑmin^-1^ |
| Matzi, 2007^166^ | Lung | Randomized controlled trial | Pre-treatment | Evaluate the effect of an intervention | NR | NR | NR | NR | No calibration reported  AE: NR  Predefined Safety Measures: NR | NR |
| Minnella, 2021 ^167^ | Lung | Retrospective cohort study | Pre-treatment | Pre surgery evaluation | CE | NR sec sampling | AT/VT VO₂peak | NR | Calibration: No Predefined Safety Measures: NR AE: NR; Other | 16.8 mLˑkg⁻¹ˑmin⁻¹ |
| Miyazaki, 2018^168^ | Lung | Retrospective cohort study | Pre-post treatment | Pre surgery evaluation | CE | NR | NR | NR | No calibration reported  AE: NR  Predefined Safety Measures: NR | NR |
| Nagamatsu  , 1996^169^ | Lung | Prospective cohort study | Pre-treatment | Evaluate validity (e.g. VO_2_peak vs ISWT) | CE | 20 sec sampling | NR | NR | No calibration reported  AE: NR  Predefined Safety Measures: NR | NR |
| Nagamatsu, 2004^170^ | Lung | Prospective cohort study | Pre-treatment | Pre surgery evaluation | CE | 20 sec sampling | NR | Borg > 16 | No calibration reported  AE: NR  Predefined Safety Measures: NR | NR |
| Nagamatsu, 2011^171^ | Lung | Prospective cohort study | Pre-post treatment | Pre-post surgery evaluation | CE | 20 sec sampling | NR | RPE (Borg scale) > 16 | No calibration reported  AE: NR  Predefined Safety Measures: NR | NR |
| Nezu, 1998^172^ | Lung | Prospective cohort study | Pre-post treatment | Pre-post surgery evaluation (effect of surgery) | CE | NR | VO_2_peak  HR Peak  O_2_ pulse | RPE | No calibration reported  AE: NR  Predefined Safety Measures: No | 19.2 mLˑkg^-1^ˑmin^-1^  NR |
| Novoa, 2011^173^ | Lung | Prospective cohort study | Pre-treatment | Evaluate validity (e.g. VO_2_peak vs ISWT) | CE | NR | VO_2_peak | NR | Calibration procedures described in detail  AE: NR  Predefined Safety Measures: NR | 20.3 mLˑkg^-1^ˑmin^-1^  NR  84% pred |
| Nugent, 1999^174^ | Lung | Prospective cohort study | Pre-post treatment | Pre-post surgery evaluation (effect of surgery) | NR | 15 sec sampling | NR | RPE | Yes, calibration reported  AE: NR  Predefined Safety Measures: NR | NR |
| Op den Kamp, 2012^175^ | Lung | Case-control study | Post treatment | Evaluate acute toxicity one-year post-therapy | CE | NR | VO_2_peak | NR | No calibration reported  AE: NR  Predefined Safety Measures: NR | NR |
| Perrotta, 2019^176^ | Lung | Retrospective cohort study | Pre-treatment | Pre surgery evaluation | CE | NR | VO_2_peak | symptom-limited, peak HR | No calibration reported  AE: NR  Predefined Safety Measures: Yes | 17.7 mLˑkg^-1^ˑmin^-1^  NR  64% pred |
| Pierce, 1994^177^ | Lung | Prospective cohort study | Pre-post treatment | Pre-post surgery evaluation (effect of surgery) | CE | NR | VO_2_peak HR Peak | RPE | No calibration reported  AE: NR  Predefined Safety Measures: Yes | 18.4 mLˑkg^-1^ˑmin^-1^  NR |
| Quist, 2018^178^ | Lung | Randomized control trial | Post treatment | To evaluate the effect of an intervention | CE | NR | VO_2_peak | NR | No calibration reported  AE: NR  Predefined Safety Measures: NR | 1.59 L·min^-1^ |
| Quist, 2020^179^ | Lung | Randomized control trial | During treatment | To evaluate the effect of an intervention | CE | NR | VO_2_peak | NR | No calibration reported  AE: NR  Predefined Safety Measures: NR | Intervention group: 1.47 L·min^-1^  Control group: 1.59 L·min^-1^ |
| Ribas, 2001^180^ | Lung | Prospective cohort study | Pre-treatment | Pre surgery evaluation | CE | NR | VO_2_peak | NR | No calibration reported  AE: NR  Predefined Safety Measures: Yes | 16.6 mLˑkg^-1^ˑmin^-1^  NR  80% pred |
| Rocco, 2013^181^ | Lung | Prospective cohort study | Pre-treatment | Pre surgery evaluation | CE | NR | VO_2_peak | NR | No calibration reported  AE: NR  Predefined Safety Measures: NR | 15.3 mLˑkg^-1^ˑmin^-1^ |
| Rodrigues, 2016^182^ | Lung | Retrospective cohort study | Pre-treatment | Pre surgery evaluation | CE | NR | VO_2_peak | symptom-limited | No calibration reported  AE: NR  Predefined Safety Measures: NR | 16.5 mLˑkg^-1^ˑmin^-1^  NR  65% pred |
| Rushwan, 2024^183^ | Lung | Prospective cohort study | Pre-treatment | Pre surgery evaluation | CE | NR sec sampling | AT HR VEVCO₂ VO₂ Wmax VO₂peak | Judgment by the technician Exhaustion | Calibration: No Predefined Safety Measures: Yes AE: NR | 16.4 mLˑkg⁻¹ˑmin⁻¹ |
| Salhi, 2015^184^ | Lung | Randomized controlled trial | Pre-post treatment | 1) Pre-post surgery evaluation  2) Evaluate the effect of an intervention | CE | NR | VO_2_peak | NR | No calibration reported  AE: NR  Predefined Safety Measures: NR | Intervention:  1.34 L·min^-1^  Control:1.35 L·min^-1^ |
| Shafiek, 2016^185^ | Lung | Cross-sectional study | Pre-treatment | Pre surgery evaluation | CE | NR | VO_2_peak  HR Peak  O_2_ pulse  Ventilations  VEVCO_2_  RER | NR | No calibration procedures mentioned  AE: NR  Predefined Safety Measures: NR | Complications:  16.7 mLˑkg^-1^ˑmin^-1^  75% pred  No complications:  16.5 mLˑkg^-1^ˑmin^-1^  75% pred |
| Stanzani, 2014^186^ | Lung | Retrospective cohort study | Pre-post treatment | Pre surgery evaluation | CE | 15 sec sampling | NR | NR | No calibration reported  AE: NR  Predefined Safety Measures: NR | NR |
| Stefanelli, 2013^187^ | Lung | Non-randomized intervention study | Pre-treatment | Evaluate the effect of an intervention | CE | NR | VO_2_peak | % of pred HR: NR  the occurrence of other kinds of limitations | No calibration reported  AE: NR  Predefined Safety Measures: NR | Overall: NR  Intervention:  14.9 mLˑkg^-1^ˑmin^-1^  65% pred  Control:  14.8 mLˑkg^-1^ˑmin^-1^  61% pred |
| Torchio, 2010^188^ | Lung | Retrospective cohort study | Pre-treatment | Pre surgery evaluation | TM | NR | VO_2_peak  VEVCO_2_ | NR | No calibration reported  AE: NR  Predefined Safety Measures: NR | 19.0 mLˑkg^-1^ˑmin^-1^  1.39 L·min^-1^  65% pred |
| Torchio, 2017^189^ | Lung | Retrospective cohort study | Pre-treatment | Pre surgery evaluation | TM | 20 sec sampling | VO_2_peak  VEVCO_2_ | NR | No calibration reported  AE: NR  Predefined Safety Measures: NR | 19.0 mLˑkg^-1^ˑmin^-1^ |
| Umezu, 2012^190^ | Lung | Prospective cohort study | Pre-treatment | Pre surgery evaluation | CE | 60 sec sampling | NR | Symptom limited | No calibration reported  AE: NR  Predefined Safety Measures: NR | NR |
| Vargas Fajardo, 2014^191^ | Lung | Prospective cohort study | Pre-during post treatment | Pre surgery evaluation | CE | NR | VO_2_peak | Symptom limited | No calibration reported  AE: NR  Predefined Safety Measures: NR | 18.8 mLˑkg^-1^ˑmin^-1^  78% of pred |
| Villani, 2004^192^ | Lung | Prospective cohort study | Pre-treatment | Pre surgery evaluation | CE | NR | VO_2_peak mLˑkg^-1^ˑmin^-1^ VO_2_peak L·min^-1^  VO_2_peak in % of pred HR Peak  O_2_ pulse | > 100% of age pred  Exhaustion | No calibration reported  AE: NR  Predefined Safety Measures: Yes | 21.5 mLˑkg^-1^ˑmin^-1^  1.5 L·min^-1^  76% pred |
| Walsh, 1994^193^ | Lung | Prospective cohort study | Pre-treatment | Pre surgery evaluation | CE | NR | VO_2_peak | Exhaustion  dyspnea | No calibration reported  AE: NR  Predefined Safety Measures: NR | Overall: NR  Surgical:  15.4 mLˑkg^-1^ˑmin^-1^  Medical:  12.5 mLˑkg^-1^ˑmin^-1^ |
| Wang, 1999^194^ | Lung | Prospective cohort study | Pre-treatment | Pre surgery evaluation | CE | NR | VO_2_peak | NR | No calibration reported  Testing related adverse event: NR  Predefined Safety Measures: NR | 17.9 mLˑkg^-1^ˑmin^-1^ |
| Wang, 2000^195^ | Lung | Prospective cohort study | Pre-treatment | Pre surgery evaluation | CE | NR | VO_2_peak mLˑkg^-1^ˑmin^-1^  VO_2_peak in % of pred  O_2_ pulse | % of pred HR ≥90 | Unclear calibration procedures  AE: NR  Predefined Safety Measures: NR | 18.0 mLˑkg^-1^ˑmin^-1^  74% pred |
| Wang, 2006^196^ | Lung | Prospective cohort study | Pre-post treatment | Pre-post surgery evaluation | CE | NR | VO_2_peak | NR | Calibration procedures described in detail  AE: NR  Predefined Safety Measures: NR | 18.5 mLˑkg^-1^ˑmin^-1^  78% pred |
| Wang, 2011 ^197^ | Lung | Retrospective cohort study | Pre-treatment | Pre surgery evaluation | NR | NR | VO_2_peak | NR | No calibration reported  AE: NR  Predefined Safety Measures: NR | 15.0 mLˑkg^-1^ˑmin^-1^ |
| Win, 2005^198^ | Lung | Prospective cohort study | Pre-treatment | Pre surgery evaluation | TM | 30 sec sampling | VO_2_peak | Exhaustion | No calibration reported  AE: NR  Predefined Safety Measures: NR | 18.8 mLˑkg^-1^ˑmin^-1^  88% pred |
| Win, 2006^199^ | Lung | Prospective cohort study | Pre- treatment | Evaluate validity (e.g. VO_2_peak vs ISWT) | TM | 30 sec sampling | VO_2_peak | Judgment by the technician  Voluntary termination | No calibration reported  Testing Related AE: Yes  Predefined Safety Measures: Yes | 18.3 mLˑkg^-1^ˑmin^-1^  84% pred |
| Win, 2008^200^ | Lung | Prospective cohort study | Pre-treatment | Pre surgery evaluation | CE | 15 sec sampling | VO_2_peak | NR | No calibration reported  AE: NR  Predefined Safety Measures: NR | 18.8 mLˑkg^-1^ˑmin^-1^  88% pred |
| Puente-Maestú, 2011^201^ | Lung and sarcoma | Prospective cohort study | Pre-treatment | Pre surgery evaluation | CE | 20 sec sampling | VO_2_peak | NR | No calibration reported  AE: NR  Predefined Safety Measures: NR | 18.1 mLˑkg^-1^ˑmin^-1^ |
| Courneya, 2009 ^202^ | Lymphoma | Randomized controlled trial | During treatment | To evaluate the effect of an intervention | NR | 15 sec sampling | ECG AT VO₂ | NR | Calibration: No Predefined Safety Measures: Yes AE: NR | Overall: NR  Intervention: 24.7 mLˑkg^-1^ˑmin^-1^  Control: 25.4 mLˑkg^-1^ˑmin^-1^ |
| Elbl, 2006^203^ | Lymphoma | Prospective cohort study | Post treatment | Evaluate late toxicity > 1 year post-therapy | NR | NR sec sampling | VO₂ VCO₂ | Exhaustion | Calibration: No Predefined Safety Measures: NR AE: NR | NR |
| Elbl, 2006^204^ | Lymphoma | Prospective cohort study | Pre-post treatment | Pre-post treatment evaluation other than surgery (e.g. effect of drugs/radiation) | CE | NR sec sampling | ECG BP VO₂ VO₂peak | Exhaustion | Calibration: No Predefined Safety Measures: Yes AE: NR | 28.4 mLˑkg⁻¹ˑmin⁻¹ |
| Jain, 1996 ^205^ | Lymphoma | Retrospective cohort study | Pre-treatment | Pre therapy assessment | TM | NR sec sampling | VO₂ BR VeVCO₂ BP VO₂peak | RER % of pred heart rate Exhaustion | Calibration: No Predefined Safety Measures: NR AE: NR | 75.0% pred |
| Murbraech, 2016 ^206^ | Lymphoma | Cross-sectional study | Post treatment | Evaluate late toxicity > 1 year post-therapy | CE | 20 sec sampling | VO₂ RER HR BP Wmax VO₂peak | NR Symptom-limited | Calibration: No Predefined Safety Measures: NR AE: NR | 27.1 mLˑkg⁻¹ˑmin⁻¹ 102.0% pred |
| Rizwan, 2021 ^207^ | Lymphoma | Prospective cohort study | Post-treatment | Evaluate late toxicity > 1 year post-therapy | CE | NR sec sampling | AT HR O₂-pulse VEVCO₂ RER | Leveling off in VO₂ RER | Calibration: No Predefined Safety Measures: Yes AE: No | 26 mLˑkg⁻¹ˑmin⁻¹ 85% pred |
| Vermaete, 2014 ^208^ | Lymphoma | Prospective cohort study | Pre-during-post treatment | Evaluate acute toxicity < 1 year post-therapy | CE | NR sec sampling | ECG BP VO₂ VCO₂ VE SpO₂  Wmax VO₂peak | NR | Calibration: No Predefined Safety Measures: Yes AE: NR | 2.42 Lˑmin⁻¹ 103.0% pred |
| Persoon, 2017 ^209^ | Lymphoma and melanoma | Cross-sectional study | Post treatment | Evaluate acute toxicity < 1 year post-therapy | CE | 15 sec sampling | VO₂ HR RER ECG SpO₂ BP Wmax VO₂peak | NR | Calibration: No Predefined Safety Measures: Yes AE: Yes | 21.7 mLˑkg⁻¹ˑmin⁻¹ 1.7 Lˑmin⁻¹ 74.0% pred |
| Persoon, 2017 ^209^ | Lymphoma and multiple myeloma | Randomized controlled trial | Post treatment | To evaluate the effect of an intervention | CE | 15 sec sampling | VO₂ | NR | Calibration: No Predefined Safety Measures: NR AE: NR | 21.7 mLˑkg⁻¹ˑmin⁻¹ 1.7 Lˑmin⁻¹  74% pred |
| Armand, 2023 ^210^ | Mixed | Cross-sectional study | Post-treatment | Evaluate late toxicity > 1 year post-therapy | CE | NR sec sampling | HR RER Wmax VO₂peak | RER Pred HR Voluntary termination | Calibration: No Predefined Safety Measures: NR AE: NR | 42.2 mLˑkg⁻¹ˑmin⁻¹ 3.034 Lˑmin⁻¹ |
| Atkinson, 2021 ^211^ | Mixed | Randomized controlled trial | Post-treatment | To evaluate the effect of an intervention | CE | NR sec sampling | HR ER Borg ECG BP SpO₂ Wmax VO₂peak | NR | Calibration: Yes Predefined Safety Measures: Yes AE: NR | 26.5 mLˑkg⁻¹ˑmin⁻¹ 69.0% pred |
| Bertheussen, 2013^212^ | Mixed | Cross-sectional study | Post treatment | Evaluate validity (e.g. VO_2_peak vs ISWT) | TM | NR | VO_2_peak | Leveling off in VO_2_  RER >1.05  Exhaustion | No calibration reported  AE: NR  Predefined Safety Measures: NR | 31.8 mLˑkg^-1^ˑmin^-1^ |
| Bjorke, 2020^213^ | Mixed | Cross-sectional study | Pre-treatment | Method study (e.g. VO2peak vs IS) | TM | NR | VO_2_peak | NR | No calibration reported  AE: NR  Predefined Safety Measures: NR | 29.8 mLˑkg^-1^ˑmin^-1^ |
| Bjørke, 2020 ^214^ | Mixed | Prospective cohort study | Pre-treatment | Method study (e.g. VO₂peak vs ISWT) | TM | 60 sec sampling | HR VE RER Borg ECG Wmax Borg VO₂peak | Leveling off in VO₂ RER Pred HR RPE/BORG Exhaustion | Calibration: Yes Predefined Safety Measures: NR AE: NR | 29.8 mLˑkg⁻¹ˑmin⁻¹ |
| Carannante, 2023 ^215^ | Mixed | Prospective cohort study | Pre-during-post treatment | Evaluate late toxicity > 1 year post-therapy; Evaluate acute toxicity < 1 year post-therapy | CE | NR sec sampling | AT HR O₂-pulse VEVCO₂ slope  Wmax VO₂/w slope VO₂peak | RER | Calibration: No Predefined Safety Measures: NR AE: NR | 18.2 mLˑkg⁻¹ˑmin⁻¹ 70.3% pred |
| Courneya, 2008^216^ | Mixed | Randomized controlled trial | Post treatment | Evaluate the effect of an intervention | CE | 30 sec sampling | VO_2_peak  Anaerobic threshold | NR | Calibration procedures described in detail  AE: NR  Predefined Safety Measures: Yes | Overall: NR  Intervention:15.6 mLˑkg^-1^ˑmin^-1^  Control: 16.1 mLˑkg^-1^ˑmin^-1^ |
| Daneryd, 1998^217^ | Mixed | Randomized controlled trial | Post treatment | Evaluate the effect of an intervention | TM | 60 sec sampling | VO_2_peak  HR Peak  Ventilation | NR | Calibration procedures described in detail  AE: NR  Predefined Safety Measures: NR | Overall: NR  Post intervention  Control: 1.15 L·min^-1^  EPO: 1.29 L·min^-1^ |
| De Backer, 2007^218^ | Mixed | Prospective cohort study | During treatment | Evaluate validity (e.g. VO_2_peak vs ISWT) | CE | 30 sec sampling | VO_2_peak  Anaerobic threshold  HR Peak  RER | Exhaustion | No calibration reported  AE: No  Predefined Safety Measures: Yes | 27.6 mLˑkg^-1^ˑmin^-1^ |
| De Backer, 2007^219^ | Mixed | One-armed intervention study | Post treatment | 1) Pre-post treatment evaluation other than surgery  2) Evaluate the effect of an intervention | CE | NR | VO_2_peak  Anaerobic threshold  HR Peak  RER | NR | No calibration reported  AE: NR  Predefined Safety Measures: Yes | Overall: NR  Men: 30.7 mLˑkg^-1^ˑmin^-1^  Women: 24.2 mLˑkg^-1^ˑmin^-1^ |
| De Backer, 2008^220^ | Mixed | Prospective cohort study | Pre-treatment | Evaluate the effect of an intervention | CE | NR | VO_2_peak  Anaerobic threshold  HR Peak | NR | No calibration reported  AE: NR  Predefined Safety Measures: NR | 25.7 mLˑkg^-1^ˑmin^-1^ |
| Demmelmaier, 2021 ^221^ | Mixed | Randomized controlled trial | During treatment | To evaluate the effect of an intervention | TM | NR sec sampling | VO₂ RER Borg | RER RPE/BORG Judgment by the technician | Calibration: No Predefined Safety Measures: NR AE: NR | Overall: NR  Intervention: 30.5 mLˑkg^-1^ˑmin^-1^  Intervention: 30.9 mLˑkg^-1^ˑmin^-1^  Intervention: 31.2 mLˑkg^-1^ˑmin^-1^  Control: 29.1 mLˑkg^-1^ˑmin^-1^ |
| Goodenough, 2022 ^222^ | Mixed | Prospective cohort study | Post-treatment | Evaluate late toxicity > 1 year post-therapy | TM | NR sec sampling | HR VE RER ECG BP | RPE/BORG Judgment by the technician Exhaustion | Calibration: No Predefined Safety Measures: NR AE: NR | Overall: NR  Females: 22.5 mLˑkg^-1^ˑmin^-1^  Males: 28.8 mLˑkg^-1^ˑmin^-1^ |
| Griffith, 2009^223^ | Mixed | Randomized controlled trial | During treatment | Evaluate the effect of an intervention | TM | NR | VO_2_peak | RPE ≥ 18  RER ≥ 1.10 | No calibration reported  AE: NR  Predefined Safety Measures: NR | Overall: NR  Intervention: 13.9 mLˑkg^-1^ˑmin^-1^  Control: 13.2 mLˑkg^-1^ˑmin^-1^ |
| Henriksson, 2023 ^224^ | Mixed | Randomized controlled trial | During treatment | To evaluate the effect of an intervention | TM | NR sec sampling | NR | RER RPE/BORG Judgment by the technician | Calibration: No Predefined Safety Measures: Yes AE: NR | Overall: NR  BC low: 29.9 mLˑkg^-1^ˑmin^-1^  BC high: 31.3 mLˑkg^-1^ˑmin^-1^  PC low: 29.9 mLˑkg^-1^ˑmin^-1^  PC high: 30.0 mLˑkg^-1^ˑmin^-1^ |
| Huang, 2016^225^ | Mixed | Retrospective cohort study | Pre-treatment | 1) Pre surgery evaluation  2) Evaluate the effect of an intervention | CE | NR | VO_2_peak  AT | NR | No calibration reported  AE: NR  Predefined Safety Measures: Yes | 16.0 mLˑkg^-1^ˑmin^-1^ |
| Jones, 2014 ^226^ | Mixed | Randomized controlled trial | Post treatment | Evaluate the effect of an intervention | NR | NR | VO_2_peak  Anaerobic threshold  RER | NR | No calibration reported  AE: NR  Predefined Safety Measures: Yes | Overall: NR  Intervention: 14.5 mLˑkg^-1^ˑmin^-1^  Control: 14.3 mLˑkg^-1^ˑmin^-1^  Intervention: 1.2 L·min^-1^  Control: 1.2 L·min^-1^ |
| Kampshoff, 2015^227^ | Mixed | Randomized controlled trial | Post treatment | Evaluate the effect of an intervention | CE | 15 sec sampling | VO_2_peak  Anaerobic threshold | NR | No calibration reported  AE: NR  Predefined Safety Measures: NR | Overall: NR  High intensity training:  21.9 mLˑkg^-1^ˑmin^-1^  Low-to-moderate intensity:  22.3 mLˑkg^-1^ˑmin^-1^  Control: 21.5 mLˑkg^-1^ˑmin^-1^ |
| Kampshoff, 2018^228^ | Mixed | Randomized controlled trial | Post treatment | Evaluate the effect of an intervention | CE | 60 sec sampling | VO_2_peak | NR | No calibration reported  AE: NR  Predefined Safety Measures: NR | Overall: NR  Intervention: 22.1 mLˑkg^-1^ˑmin^-1^ |
| Kenjale, 2014^229^ | Mixed | Retrospective cohort study | Pre-treatment | Pre therapy assessment | TM | 30 sec sampling | VO_2_peak  HR Peak  O_2_ pulse | symptom-limited  RER: NR | No calibration reported  AE: Yes  Predefined Safety Measures: NR | 21.7 mLˑkg^-1^ˑmin^-1^  81% pred |
| Kerrigan, 2023 ^230^ | Mixed | Randomized controlled trial | subclinical cardiotoxicity | To evaluate the effect of an intervention | TM | 30 sec sampling | HR | Judgment by the technician Exhaustion | Calibration: No Predefined Safety Measures: NR AE: NR | Overall: NR  Intervention: 16.9 mLˑkg^-1^ˑmin^-1^  Usual Care: 17.9 mLˑkg^-1^ˑmin^-1^ |
| Klika, 2011^231^ | Mixed | Case-control study | Post treatment | To compare the physiological responses and lactate kinetics during CPET | CE | NR | VO_2_peak  Anaerobic threshold  HR Peak | Exhaustion | Calibration procedures described in detail  AE: NR  Predefined Safety Measures: Yes | Overall: NR  Women: 34.8 mLˑkg^-1^ˑmin^-1^  Men: 45.0 mLˑkg^-1^ˑmin^-1^ |
| Li, 2018^232^ | Mixed | Retrospective cohort study | Pre-treatment | Evaluate validity (e.g. VO_2_peak vs ISWT) | CE | NR | VO_2_peak  Anaerobic threshold | Voluntary termination  Termination judged by the physician | No calibration reported  AE: NR  Predefined Safety Measures: Yes | Overall: NR  Measured: 17.6 mLˑkg^-1^ˑmin^-1^  Estimated: 37.5 mLˑkg^-1^ˑmin^-1^ |
| Lundholm, 2004^233^ | Mixed | Randomized controlled trial | During treatment | Evaluate the effect of an intervention | TM | 60 sec sampling | NR | Exhaustion | Unclear calibration procedures  AE: NR  Predefined Safety Measures: NR | Overall: NR  Intervention: 1268 mLˑmin^-1^  Usual Care: 1259 mLˑkg^-1^ˑmin^-1^ |
| May, 2008^234^ | Mixed | Randomized controlled trial | Post treatment | Evaluate the effect of an intervention | CE | 30 sec sampling | VO_2_peak | clinical symptoms, when the cycling rate was lower than 60 rpm, or by the physician’s intervention. | Unclear calibration procedures  AE: NR  Predefined Safety Measures: Yes | Intervention: 23.9 mLˑkg^-1^ˑmin^-1^  Control: 23.3 mLˑkg^-1^ˑmin^-1^  84% pred |
| May, 2010^235^ | Mixed | Prospective cohort study | Post treatment | 1) Evaluate the effect of an intervention  2) Evaluate validity (e.g. VO_2_peak vs ISWT) | CE | 30 sec sampling | VO_2_peak | Leveling off in VO_2_  RER > 1.1  % of pred HR: NR  Exhaustion | Unclear calibration procedures  AE: NR  Predefined Safety Measures: NR | 23.7 mLˑkg^-1^ˑmin^-1^ |
| Midtgaard, 2013^236^ | Mixed | Randomized controlled trial | Post treatment | To evaluate the effect of an intervention | CE | NR | VO_2_peak | NR | No calibration reported  AE: NR  Predefined Safety Measures: Yes | Intervention: 1.97  Control: 1.99 |
| Nadruz, 2018^237^ | Mixed | Case-control study | Post treatment | Evaluate late toxicity > 1-year post-therapy | NR | 10 sec | VO_2_peak  HR Peak  VEVCO_2_  RER | RER > 1.1  Symptom limited | No calibration reported  AE: NR  Predefined Safety Measures: Yes | 16.0 mLˑkg^-1^ˑmin^-1^  62% pred |
| Nawoor-Quinn, 2022 ^238^ | Mixed | Prospective cohort study | Pre-treatment | Pre surgery evaluation | CE | NR sec sampling | VE Vt VO₂ VCO₂ RER VEVCO₂ VEVO₂ HR SpO₂ Wmax | NR | Predefined Safety Measures: NR AE: NR | 17.1 mLˑkg^-1^ˑmin^-1^ |
| Ness, 2020^239^ | Mixed | Prospective cohort study | Post treatment | Evaluate late toxicity > 1-year post-therapy | TM | NR | VO_2_peak | NR | No calibration reported  AE: NR  Predefined Safety Measures: NR | Anthracyclines/chest radiot: 25.7 mLˑkg^-1^ˑmin^-1^  Surv without: 26.82 mLˑkg^-1^ˑmin^-1^  Healthy: 32.69 mLˑkg^-1^ˑmin^-1^  Anthracyclines/chest radiot: 78% pred  Surv without: 82% pred  Healthy: 98% pred |
| O'Mahony, 2021 ^240^ | Mixed | Prospective cohort study | Pre-treatment | Pre surgery evaluation | CE | NR sec sampling | AT VO₂ VEVCO₂ Wmax VO₂peak | NR | Calibration: No Predefined Safety Measures: NR AE: NR | 17.15 mLˑkg⁻¹ˑmin⁻¹ |
| Pahl, 2020^241^ | Mixed | Randomized controlled trial | Pre-post treatment | To evaluate the effect of an intervention | CE | NR | NR | NR | No calibration reported  AE: NR  Predefined Safety Measures: Yes | Overall: NR  Median:  Intervention: 19.5 mLˑkg^-1^ˑmin^-1^  Control: 25.7 mLˑkg^-1^ˑmin^-1^ |
| Pein, 2004^242^ | Mixed | Cross-sectional study | Post treatment | Evaluate late toxicity > 1-year post-therapy | NR | NR | NR | NR | No calibration reported  AE: NR  Predefined Safety Measures: NR | NR |
| Pillinger, 2022 ^243^ | Mixed | Prospective cohort study | Pre-treatment | Pre surgery evaluation | CE | 30 sec sampling | AT HR VE; VEVCO₂ ECG BP Borg SpO₂ VO₂peak | RPE/BORG Exhaustion | Calibration: Yes Predefined Safety Measures: Yes AE: No | 21.1 mLˑkg⁻¹ˑmin⁻¹ |
| Prinsen, 2013^244^ | Mixed | Randomized controlled trial | Post treatment | Evaluate the effect of an intervention | CE | NR | VO_2_peak  HR Peak  Ventilation  RER | Leveling off in VO_2_  RER ≥ 1.0  % of pred HR: <10 beats below pred maximal  Blood lactate | No calibration reported  AE: NR  Predefined Safety Measures: NR | Overall: NR  Intervention: 27.0 mLˑkg^-1^ˑmin^-1^  Control: 28.0 mLˑkg^-1^ˑmin^-1^ |
| Santa Mina, 2020 ^245^ | Mixed | Cross-sectional study | During-post treatment | Method study | TM | NR sec sampling | AT HR RER Borg VO₂ ECG BP SpO₂ VO₂peak | Leveling off in VO₂ RER Pred HR RPE/BORG Judgment by the technician Exhaustion Voluntary termination | Calibration: Yes Predefined Safety Measures: Yes AE: NR | 31.3 mLˑkg⁻¹ˑmin⁻¹ |
| Schmidt, 2013^246^ | Mixed | Prospective cohort study | During treatment | Evaluate validity (e.g. VO_2_peak vs ISWT) |  | CE | VO_2_peak  HR Peak  O_2_ Pulse  RER  RPE | Maintain a cadence above 60 rpe | Calibration procedures described in detail  AE: NR  Predefined Safety Measures: Yes | 21.2 mLˑkg^-1^ˑmin^-1^  69% pred |
| Schmitt, 2016^247^ | Mixed | Randomized controlled trial | Post treatment | To evaluate the effect of an intervention | TM | NR | NR | NR | No calibration reported  AE: NR  Predefined Safety Measures: Yes | Overall: NR  Intervention: 27.1 mLˑkg^-1^ˑmin^-1^  Control: 23.8 mLˑkg^-1^ˑmin^-^ |
| Schneider, 2023 ^248^ | Mixed | Randomized controlled trial | Pre-during-post treatment | To evaluate the effect of an intervention | CE | 30 sec sampling | RER Wmax ECG VO₂ VO₂peak | RER Exhaustion | Calibration: No Predefined Safety Measures: Yes AE: NR | 88.0% pred |
| Stuiver, 2017^249^ | Mixed | Cross-sectional study | Pre-post treatment | Evaluate validity (e.g. VO_2_peak vs ISWT) | CE | NR | VO_2_peak | Maximum performance | No calibration reported  AE: NR  Predefined Safety Measures: NR | 21.7 mLˑkg^-1^ˑmin^-1^ |
| van der Schoot, 2022 ^250^ | Mixed | Randomized controlled trial | Pre-treatment | To evaluate the effect of an intervention | CE | NR sec sampling | NR | NR | Calibration: No Predefined Safety Measures: Yes AE: NR | Overall: NR  Intervention: 30.4 mLˑkg^-1^ˑmin^-1^  Control: 29.7 mLˑkg^-1^ˑmin^-^ |
| Viamonte, 2023 ^251^ | Mixed | Randomized controlled trial | Post-treatment | To evaluate the effect of an intervention | TM | NR sec sampling | NR | NR | Calibration: No Predefined Safety Measures: NR AE: NR | Overall: NR  Intervention: 24.4 mLˑkg^-1^ˑmin^-1^  Control: 23.8 mLˑkg^-1^ˑmin^-^ |
| Waterland, 2022 ^252^ | Mixed | Prospective cohort study | Pre-treatment | Pre surgery evaluation | CE | NR sec sampling | AT/VT  PetO₂ VO₂ VO₂peak | NR | Calibration: No Predefined Safety Measures: NR AE: NR | 14 mLˑkg⁻¹ˑmin⁻¹ |
| Weemaes, 2021 ^253^ | Mixed | Prospective cohort study | Post-treatment | Method study | CE | NR sec sampling | NR | Exhaustion | Calibration: Calibration procedures are mentioned, but not when and how Predefined Safety Measures: NR AE: NR | 19.5 mLˑkg⁻¹ˑmin⁻¹ |
| Weert, 2004^254^ | Mixed | One-armed intervention study | Post treatment | Evaluate the effect of an intervention | CE | NR | VO_2_peak  O_2_ pulse  RER | symptom-limited | No calibration reported  AE: NR  Predefined Safety Measures: NR | 1.39 L·min^-1^ |
| Wiestad, 2020 ^255^ | Mixed | Prospective cohort study | Pre-post treatment | Evaluate late toxicity > 1 year post-therapy | TM | 30 sec sampling | HR VE; RER Borg | RER RPE/BORG Judgment by the technician Exhaustion | Calibration: Yes Predefined Safety Measures: Yes AE: NR | Chemotherapy: 31 mLˑkg^-1^ˑmin^-1^  Radiation: 29.4 mLˑkg^-1^ˑmin^-1^  Endocrine: 32 mLˑkg^-1^ˑmin^-^ |
| Koutoukidis, 2020^256^ | Multiple myeloma | Randomized controlled trial | During-post treatment | To evaluate the effect of an intervention | CE | NR | NR | NR | No calibration reported  AE: NR  Predefined Safety Measures: NR | Overall: NR  Intervention: 18.1 mLˑkg^-1^ˑmin^-1^  Control: 19.3 mLˑkg^-1^ˑmin^-^ |
| Van Dongen, 2019^257^ | Multiple myeloma | Randomized controlled trial | Post treatment | To evaluate the effect of an intervention | CE | NR | VO_2_peak | NR | No calibration reported  AE: NR  Predefined Safety Measures: NR | 22 mLˑkg^-1^ˑmin^-1^ |
| Lee, 2022 ^258^ | Ovarian | Randomized controlled trial | Post-treatment | To evaluate the effect of an intervention | TM | NR sec sampling | ECG BP  RPE | RPE/BORG | Calibration: No Predefined Safety Measures: Yes AE: NR | Overall: NR  Intervention: 33.5 mLˑkg^-1^ˑmin^-1^  Control: 35.5 mLˑkg^-1^ˑmin^-^ |
| Pinelli, 2021 ^259^ | Ovarian | Prospective cohort study | During treatment | Pre surgery evaluation | NR | NR sec sampling | NR | NR | Calibration: No Predefined Safety Measures: NR AE: NR | NR |
| Smits, 2023 ^260^ | Ovarian | Prospective cohort study | Pre-treatment | Pre surgery evaluation | CE | NR sec sampling | AT/VT VEVCO₂ VO₂ | NR | Calibration: No Predefined Safety Measures: Yes AE: NR | NR |
| Ausania, 2012^261^ | Pancreatic | Prospective cohort study | Pre-treatment | Pre surgery evaluation | NR | NR | Anaerobic threshold | NR | No calibration reported  AE: NR  Predefined Safety Measures: ECG and BP | No complication:  14.1 mLˑkg^-1^ˑmin^-1^  Complication:  11.3 mLˑkg^-1^ˑmin^-1^ |
| Chandrabalan, 2013^262^ | Pancreatic | Retrospective cohort study | Pre-treatment | Pre surgery evaluation | CE | NR | Anaerobic threshold | Symptom limited | No calibration reported  AE: NR  Predefined Safety Measures: NR | Peak: NR  Median AT: 10.3 mLˑkg^-1^ˑmin^-1^ |
| Junejo, 2014^263^ | Pancreatic | Prospective cohort study | Pre-treatment | Pre surgery evaluation | CE | By 5 rolling breath-by-breath measures | NR | NR | Calibration procedures described in detail  AE: NR  Predefined Safety Measures: Yes | NR |
| Alberga, 2012^264^ | Prostate | Randomized controlled trial | Pre-during treatment | Evaluate the effect of an intervention | TM | 30 sec sampling | VO_2_peak | Voluntary termination | No calibration reported  AE: NR  Predefined Safety Measures: NR | 28.8 mLˑkg^-1^ˑmin^-1^ |
| Bonsignore, 2018^265^ | Prostate | Retrospective cohort study | Post treatment | Evaluate the effect of an intervention | TM | 20 sec sampling | VO_2_peak  HR Peak | NR | Calibration procedures described in detail  AE: NR  Predefined Safety Measures: NR | 16.9 mLˑkg^-1^ˑmin^-1^ |
| Djurhuus, 2023 ^266^ | Prostate | Randomized controlled trial | Pre-treatment | To evaluate the effect of an intervention | CE | 30 sec sampling | HR VEVCO₂ RER Wmax | Leveling off in VO₂ RER RPE/BORG | Calibration: No Predefined Safety Measures: NR AE: NR | Overall: NR  Intervention: 31.4 mLˑkg^-1^ˑmin^-1^  Control: 33.6 mLˑkg^-1^ˑmin^-1^ |
| Eriksen, 2017^267^ | Prostate | Randomized controlled trial | Active surveillance | Evaluate the effect of an intervention | CE | NR | VO_2_peak | NR | No calibration reported  AE: NR  Predefined Safety Measures: NR | Overall: NR  Intervention: 29.0 mLˑkg^-1^ˑmin^-1^  Control: 26.0 mLˑkg^-1^ˑmin^-1^ |
| Harrison, 2022 ^268^ | Prostate | Randomized controlled trial | Pre-during treatment | To evaluate the effect of an intervention | TM | NR sec sampling | AT/VT HR O₂-pulse VE VEVCO₂ RER ECG BP SpO₂ VO₂peak | Leveling off in VO₂ RER RPE/BORG Exhaustion NR | Calibration: No Predefined Safety Measures: Yes AE: No | 28.5 mLˑkg⁻¹ˑmin⁻¹ |
| Hojan, 2017 ^269^ | Prostate | Randomized controlled trial | Pre-during treatment | To evaluate the effect of an intervention | NR | NR sec sampling | METs | NR | Calibration: NA Predefined Safety Measures: NR AE: NR | NR |
| Houben, 2023 ^270^ | Prostate | Randomized controlled trial | During treatment | To evaluate the effect of an intervention | CE | 30 sec sampling | RER ECG Wmax | NR | Calibration: No Predefined Safety Measures: Yes AE: NR | Intervention 1: 21.5 mLˑkg^-1^ˑmin^-^  Intervention 2: 19.5 mLˑkg^-1^ˑmin^-1^  Control: 23.1 mLˑkg^-1^ˑmin^-1^ |
| Jones, 2014 ^271^ | Prostate | Randomized controlled trial | Post treatment | To evaluate the effect of an intervention | TM | NR sec sampling | HR VEVCO₂ RER | NR | Calibration: No Predefined Safety Measures: No AE: No | Overall: NR  Intervention: 27.7 mLˑkg^-1^ˑmin^-1^  Control: 29.2 mLˑkg^-1^ˑmin^-1^ |
| Kang, 2021 ^272^ | Prostate | Randomized controlled trial | Pre-treatment | To evaluate the effect of an intervention | TM | 15 sec sampling | HR RER | RER Pred HR RPE/BORG Exhaustion | Calibration: No Predefined Safety Measures: NR AE: NR | Overall: NR  Intervention: 29.6 mLˑkg^-1^ˑmin^-1^  Control: 28.4 mLˑkg^-1^ˑmin^-1^ |
| Ndjavera, 2020^273^ | Prostate | Randomized controlled trial | Pre-during treatment | To evaluate the effect of an intervention | CE | NR | NR | NR | No calibration reported  AE: NR  Predefined Safety Measures: NR | Overall: NR  Intervention: 23.5 mLˑkg^-1^ˑmin^-1^  Control: 22.4 mLˑkg^-1^ˑmin^-1^ |
| Scott, 2015^274^ | Prostate | Randomized controlled trial | During treatment | Evaluate validity (e.g. VO_2_peak vs ISWT) | TM | 30 sec sampling | VO_2_peak  Anaerobic threshold  HR Peak  VEVCO_2_  RER | Leveling off in VO2  RER > 1.10  % of pred HR > 85% | Calibration procedures described in detail  AE: Yes  Predefined Safety Measures: NR | 27.0 mLˑkg^-1^ˑmin^-1^ |
| Segal, 2009^275^ | Prostate | Randomized controlled trial | Post treatment | Evaluate the effect of an intervention | TM | 15 sec sampling | VO_2_peak | NR | No calibration reported  AE: Yes  Predefined Safety Measures: NR | Overall: NR  Intervention: 29.4 mLˑkg^-1^ˑmin^-1^  Control: 28.8 mLˑkg^-1^ˑmin^-1^ |
| Uth, 2014^276^ | Prostate | Randomized controlled trial | During treatment | Evaluate the effect of an intervention | CE | 30 sec sampling | VO_2_peak | Leveling off in VO_2_  RER ≥ 1.05  Exhaustion | No calibration reported  AE: NR  Predefined Safety Measures: NR | Overall: NR  Intervention: 27.2 mLˑkg^-1^ˑmin^-1^  Control: 26.4 mLˑkg^-1^ˑmin^-1^ |
| Uth, 2018^277^ | Prostate | Randomized controlled trial | During-post treatment | Evaluate the effect of an intervention | CE | 30 sec sampling | VO_2_  RER | Leveling off in VO_2_  RER | No calibration reported  AE: NR  Predefined Safety Measures: No | Overall: NR  Intervention: 27.4 mLˑkg^-1^ˑmin^-1^  Control: 27.6 mLˑkg^-1^ˑmin^-1^ |
| Van Blarigan, 2023 ^278^ | Prostate | Randomized controlled trial | Pre-treatment | To evaluate the effect of an intervention | NR | NR sec sampling | NR | NR | Calibration: No Predefined Safety Measures: NR AE: NR | Overall: NR  Intervention: 28.0 mLˑkg^-1^ˑmin^-1^  Control: 27.8 mLˑkg^-1^ˑmin^-1^ |
| Adams, 2017^279^ | Testicular | Randomized controlled trial | Post treatment | Evaluate the effect of an intervention | TM | NR | VO_2_peak | Exhaustion | No calibration reported  AE: NR  Predefined Safety Measures: NR | Overall: NR  Intervention:  37.0 mLˑkg^-1^ˑmin^-1^  Control:  37.1 mLˑkg^-1^ˑmin^-1^ |
| Hyltander, 1991^280^ | Testicular | Randomized controlled trial | During treatment | Evaluate the effect of an intervention | CE | NR | NR | NR | No calibration reported  AE: NR  Predefined Safety Measures: Yes | NR |

Cardiorespiratory fitness assessment tests characterized according to the American Thoracic Society criteria for cardiopulmonary exercise tests.

**Abbreviations:** AE, Testing related Adverse events, VO_2_peak, peak aerobic capacity; NA, not applicable; O_2_, oxygen; HR, HR; bpm, beats per minute; BP, blood pressure; NR, not reported; TM, TM; min, minute; CE,CE; sec, second; RER, respiratory exchange ratio; RPE, rate of perceived exertion; ECG, electrocardiogram; HR Peak, peak HR; HR_max_, maximum HR; SpO_2_, arterial oxygen saturation

### Table S2. Six-minute-walking test Characteristics (n=240 studies)

| Study | Cancer site | Study design | Time  Points | Overall purpose | Familiarization/  performed two tests  (yes/no) | Level of Standardization  (yes/unclear/no) | Monitoring  (10 min rest, BP, SpO_2_, RPE) | 6MWD  (meter)  (% of pred) |
| --- | --- | --- | --- | --- | --- | --- | --- | --- |
| Longdon, 2020 ^3^ | Bladder | Prospective cohort study | Pre-treatment | Pre surgery evaluation | NR | No | NR | NR |
| Minnella, 2019^281^ | Bladder | Randomized controlled trial | Pre-treatment | To evaluate the effect of an intervention | NR | Unclear | NR | NR |
| Pieczynska, 2022 ^282^ | Brain | Prospective cohort study | Pre-during treatment | Pre therapy assessment | NR | Yes | Rest: Yes BP: Yes  RPE: Yes | 405 m |
| Pieczynska, 2023 ^283^ | Brain | Randomized controlled trial | Pre-during treatment | To evaluate the effect of an intervention | NR | Yes | Rest: Yes | Intervention: 483.59 m Control: 382.21 m |
| Ruden, 2011^284^ | Brain | Prospective cohort study | During treatment | Survival | NR | Yes | NR | 448m |
| Ariza-Garcia, 2019^285^ | Breast | Randomized controlled trial | During treatment | To evaluate the effect of an intervention | Pre-test familiarization | Yes | NR | NR |
| Bellissimo, 2023 ^286^ | Breast | Prospective cohort study | Post-treatment | Evaluate acute toxicity < 1-year post-therapy | NR | Yes | NR | 473m |
| Brahmbhatt, 2020 ^287^ | Breast | Non-randomized intervention study | Pre-post treatment | To evaluate the effect of an intervention | NR | No | NR | 474 m |
| Buchan, 2016^288^ | Breast | Randomized controlled trial | Post treatment | Evaluate the effect of an intervention | NR | Yes | NR | Overall: NR  Intervention: 554m  Control: 528.3m |
| CeSEiko, 2020^289^ | Breast | Randomized controlled trial | During treatment | To evaluate the effect of an intervention | Pre-test familiarization | Yes | NR | NR |
| Cornette, 2016^17^ | Breast | Randomized controlled trial | During treatment | Evaluate the effect of an intervention | Other | Yes | NR | NR |
| Diaz-Balboa, 2022 ^22^ | Breast | Cross-sectional study | During treatment | Method study | NR | Yes | NR | 606.1 m |
| Hojan, 2020^290^ | Breast | Randomized controlled trial | Post treatment | To evaluate the effect of an intervention | NR | Unclear | NR | NR |
| Kaltsatou, 2011^291^ | Breast | Randomized controlled trial | Post treatment | Evaluate the effect of an intervention | NR | No | NR | Overall: NR  Intervention: 483.3m  Control: 403.1m |
| Kim, 2019^292^ | Breast | Retrospective cohort study | During treatment | Method study | NR | Unclear | NR | 563.8m |
| Kokkonen, 2017^293^ | Breast | Prospective cohort study | During treatment | Evaluate acute toxicity | NR | No | NR | 426m |
| Kulkarni, 2013^294^ | Breast | Randomized controlled trial | During treatment | Evaluate the effect of an intervention | NR | Unclear | NR | NR |
| Mustian, 2008^295^ | Breast | Randomized controlled trial | Post treatment | Evaluate the effect of an intervention | NR | No | NR | Overall: NR  Intervention: 609.1m  Control: 611.6m |
| Ochi, 2022 ^45^ | Breast | Randomized controlled trial | Post-treatment | To evaluate the effect of an intervention | NR | Yes | NR | Intervention: 586 m Control: 603 m |
| Ortiz, 2018^296^ | Breast | Prospective cohort study | Post treatment | Method study | NR | Yes | NR | 436.4m |
| Ortiz, 2021 ^297^ | Breast | Randomized controlled trial | Post-treatment | To evaluate the effect of an intervention | No | Unclear | NR | Intervention: 430.39 m Control: 443.97 m |
| Prieto-Gόmez, 2022 ^298^ | Breast | Randomized controlled trial | Post-treatment | To evaluate the effect of an intervention | NR | Yes | NR | 327 m |
| Reis, 2013^299^ | Breast | Randomized controlled trial | During treatment | Evaluate the effect of an intervention | NR | Yes | NR | 396m |
| Santagnello, 2020^300^ | Breast | Randomized controlled trial | Post treatment | To evaluate the effect of an intervention | 6MWT were performed two times | Unclear | NR | NR |
| Scott, 2018 ^49^ | Breast | Randomized controlled trial | During treatment | To evaluate the effect of an intervention | NR | Unclear | NR | NR |
| Tubiana-Mathieu, 2021 ^56^ | Breast | Prospective cohort study | During treatment | Method study (e.g. VO₂peak vs ISWT) | NR | Unclear | NR | NR |
| Vardar Yagli, 2015 ^301^ | Breast | Randomized controlled trial | Post treatment | To evaluate the effect of an intervention | NR | Yes | BP: Yes  RPE: Yes | NR |
| Vardar Yagli, 2015^301^ | Breast | Cross-sectional study | Pre-post treatment | Evaluate late toxicity | NR | Yes | NR | 511m |
| Vincent, 2020 ^58^ | Breast | Randomized controlled trial | During-post treatment | To evaluate the effect of an intervention | NR | Unclear | NR | Intervention: 520.3 m Control: 522.9 m |
| Wang, 2011 ^302^ | Breast | Randomized controlled trial | Pre-during treatment | To evaluate the effect of an intervention | NR | No | RPE: Yes | 488.1 m NR Intervention: 503.1 m Control: 473.9 m |
| Winters-Stone, 2022 ^303^ | Breast | Randomized controlled trial | Post-treatment | To evaluate the effect of an intervention | NR | No | NR | NR |
| Bousquet- Dion, 2018^304^ | Colon/Rectum | Randomized controlled trial | Pre-post treatment | Evaluate the effect of an intervention | YES | Yes | NR | NR |
| Chen, 2017^305^ | Colon/Rectum | Randomized controlled trial | Pre-treatment | Evaluate the effect of an intervention | NR | Unclear | NR | NR |
| Chen, 2020 ^306^ | Colon/rectum | Non-randomized intervention study | During treatment | To evaluate the effect of an intervention | NR | No | NR | 435.57 m Intervention: 435.57 m Other: 435.57 m Other: 463.26 m |
| Courneya, 2016^307^ | Colon/Rectum | Randomized controlled trial | Post treatment | Evaluate the effect of an intervention | NR | No | NR | NR |
| Gillis, 2014^308^ | Colon/Rectum | Randomized controlled trial | Pre-post treatment | Evaluate the effect of an intervention | NR | Yes | NR | NR |
| Lee, 2013^74^ | Colon/Rectum | Retrospective cohort study | Pre-treatment | Pre surgery evaluation | NR | Yes | NR | NR |
| Minnella, 2020 ^76^ | Colon/rectum | Randomized controlled trial | Pre-treatment | To evaluate the effect of an intervention | NR | Yes | NR | Intervention: 487.8 m Control: 540.3 m |
| Morielli, 2021 ^77^ | Colon/rectum | Randomized controlled trial | During treatment | To evaluate the effect of an intervention | NR | Yes | NR | Intervention: 530 m Control: 548 m |
| Northgraves, 2020^309^ | Colon/Rectum | Randomized controlled trial | Pre-treatment | To evaluate the effect of an intervention | Familiarization not given | Yes | NR | NR |
| Pecorelli, 2016^310^ | Colon/Rectum | Randomized controlled trial | Pre-post treatment | Post-surgery evaluation | NR | Yes | NR | NR |
| Soares-Miranda, 2021 ^311^ | Colon/rectum | Cross-sectional study | Post-treatment | Post surgery evaluation; Association between PF and QoL | No | Yes | NR | 427 m |
| Wang, 2023 ^312^ | Colon/rectum | Retrospective cohort study | Pre-treatment | To evaluate the effect of an intervention | NR | Unclear | NR | Intervention: 389.98 m Control: 388.92 m |
| Zimmer, 2018^313^ | Colon/Rectum | Randomized controlled trial | During treatment | To evaluate the effect of an intervention | NR | No | NR | NR |
| Li, 2022 ^314^ | Colon/rectum and Gastric | Non-randomized intervention study | Pre-post treatment | To evaluate the effect of an intervention | NR | No | NR | Intervention: 548 m Control: 550 m Other: 564 m Other: 552 m |
| Chang, 2020 ^88^ | Esophageal cancer | Randomized controlled trial | Post treatment | To evaluate the effect of an intervention | NR | No | NR | 367.51 m NR Intervention: 392.54 m Control: 351.73 m |
| Eriksson, 2021 ^315^ | Esophageal cancer | Prospective cohort study | Pre-treatment | Pre surgery evaluation | NR | Yes | NR | Overall: NR  Median:  Males: 492m (89% pred)  Females: 510m (101%pred) |
| Guinan, 2018^316^ | Esophageal | Prospective cohort study | Pre-during treatment | Evaluate acute toxicity | NR | Yes | NR | 529.4m |
| Guinan, 2019^317^ | Esophageal | Prospective cohort study | Pre-post treatment | Pre-post surgery evaluation (effect of surgery) | NR | Yes | NR | 502.6m |
| Inoue, 2016 ^318^ | Esophageal cancer | Retrospective cohort study | Pre-post treatment | Pre-post surgery evaluation (effect of surgery) | NR | Yes | RPE: Yes | 494 m |
| Minnella, 2018 ^319^ | Esophageal cancer | Randomized controlled trial | Pre-treatment | To evaluate the effect of an intervention | NR | Yes | NR | Intervention: 452.1 m Control: 449.2 m |
| Otani, 2020^320^ | Esophageal | Prospective cohort study | Pre-post treatment | Pre-post surgery evaluation (effect of surgery) | NR | Unclear | NR | NR |
| Sugimura, 2022 ^321^ | Esophageal cancer | Cross-sectional study | Pre-treatment | Survival | NR | Yes | NR | 470.8m |
| Tan, 2006^322^ | Esophageal | Prospective cohort study | Pre-post treatment | Pre-post surgery evaluation (effect of surgery) | NR | No | BP | 286.2m |
| Tang, 2021 ^323^ | Esophageal cancer | Prospective cohort study | Pre-treatment | Pre surgery evaluation | NR | Yes | NR | 407 m |
| Eden, 2018^324^ | Head-neck/CNS | Cross-sectional study | During treatment | Method study | YES | Yes | NR | 542.15m |
| Samuel, 2013^325^ | Head-neck/CNS | Randomized controlled trial | During treatment | Evaluate the effect of an intervention | NR | Unclear | NR | Overall: NR  Intervention: 408m  Control: 396m |
| Samuel, 2019^326^ | Head-neck / CNS | Randomized controlled trial | During treatment | To evaluate the effect of an intervention | NR | No | NR | NR |
| Su, 2017^327^ | Head-neck/CNS | Randomized controlled trial | During treatment | Evaluate the effect of an intervention | NR | No | NR | NR |
| Yen, 2019 ^328^ | Head-neck / CNS | Randomized controlled trial | During treatment | To evaluate the effect of an intervention | NR | Yes | Rest: Yes BP: Yes RPE: Yes | Intervention: 410.1 m Control: 392.2 m |
| van Wijk, 2021 ^106^ | Hepatobillary | Prospective cohort study | Pre-treatment | To evaluate the effect of an intervention | NR | No | NR | NR |
| Hayashi, 2017^329^ | Hepato-pancreato-biliary and liver | Prospective cohort study | Pre-treatment | Pre surgery evaluation | NR | Yes | NR | NR |
| Keerthana, 2024 ^330^ | Leukemia | Randomized controlled trial | During treatment | To evaluate the effect of an intervention | NR | No | NR | Intervention: 336.45 m Control: 315.9 m |
| Labonte, 2020 ^109^ | Leukemia | Cross-sectional study | Post treatment | Method study (e.g. VO₂peak vs ISWT) | Yes | Yes | Rest: Yes BP: Yes RPE: Yes | 611.2 m |
| Labonté, 2020 ^331^ | Leukemia | Prospective cohort study | Post-treatment | Method study (e.g. VO₂peak vs ISWT) | NR | Yes | Rest: Yes BP: Yes  RPE: Yes | 611.2 m |
| Morris, 2011^332^ | Leukemia | Retrospective cohort study | Post treatment | Evaluate acute toxicity  Evaluate effect on physical performance after methylprednisolone | NR | Unclear | NR | 384m |
| Ness, 2012^333^ | Leukemia | Prospective cohort study | Post treatment | Evaluate late toxicity | NR | Unclear | NR | NR |
| Timilshina, 2019^334^ | Leukemia | Prospective cohort study | Pre-post treatment | Evaluate late toxicity > 1-year post-therapy | NR | No | NR | 481.6m |
| Wogksch, 2022 ^335^ | Leukemia | Case-control study | Post-treatment | Examine associations | NR | Unclear | NR | NR |
| Armenian, 2017^117^ | Leukemia  Lymphoma | Retrospective cohort study | Post treatment | Diagnostic (e.g. hypoxemia, ischemia) | NR | Yes | NR | Overall: NR  474m (244 to 640) Median (range) |
| Bewarder, 2019 ^336^ | Leukemia and lymphoma | Non-randomized intervention study | During treatment | To evaluate the effect of an intervention | NR | Unclear | NR | 447 m |
| DeFeo, 2020^337^ | Leukemia Lymphoma | Case-control study | Post treatment | Evaluate late toxicity > 1-year post-therapy | NR | Yes | RPE  Dyspnea | Osteonecrosis: 523.5m  No Osteonecrosis: 572.1m |
| Jones, 2015^338^ | Leukemia  Lymphoma | Retrospective cohort study | Pre-post treatment | Pre therapy assessment  Pre-post treatment evaluation other than surgery | NR | Yes | NR | 404m (median) |
| Morais, 2023 ^339^ | Leukemia  Lymphoma | Non-randomized intervention study | Pre-post treatment | To evaluate the effect of an intervention | NR | No | NR | 386.2 m 64.0 % |
| Morishita, 2013^340^ | Leukemia  Lymphoma | Prospective cohort study | Pre-post treatment | Evaluate acute toxicity | NR | Yes | NR | 502.9m |
| Morishita, 2015^341^ | Leukemia  Lymphoma | Prospective cohort study | Pre-post treatment | Pre-post treatment evaluation other than surgery | NR | Yes | NR | 478.7m |
| Morishita, 2017^342^ | Leukemia  Lymphoma | Prospective cohort study | Pre-post treatment | Pre-post treatment evaluation other than surgery  Evaluate acute toxicity | NR | Yes | NR | 492.5m |
| Kawaguchi, 2020^343^ | Liver | Retrospective cohort study | During treatment | Other: impact of serum decorin levels on physical function and prognosis | NR | No | NR | 379m |
| Ambrogi, 2012^344^ | Lung | Prospective cohort study | Post treatment | Pre-post surgery evaluation | NR | Unclear | NR | 78% pred |
| An, 2023 ^345^ | Lung | Cross-sectional study | Pre-treatment | Pre surgery evaluation | NR | Unclear | NR | Other: 517.39 m Other: 521.33 m |
| Andreetti, 2014^346^ | Lung | Prospective cohort study | Pre-post treatment | Pre-post surgery evaluation | NR | No | NR | 257.4m |
| Araujo, 2016^347^ | Lung | Prospective cohort study | Pre-post treatment | Pre-post surgery evaluation | Yes | Unclear | NR | 418m |
| Ayub, 2023 ^348^ | Lung | Cross-sectional study | Pre-treatment | Pre surgery evaluation | No | Yes | Rest: Yes BP: Yes  RPE: Yes | Other: 314.1 m Other: 423.4 m |
| Bhatia, 2019 ^128^ | Lung | Randomized controlled trial | Pre-treatment | To evaluate the effect of an intervention | NR | No | NR | Intervention: 368 m Control: 398 m |
| Bradley, 2013^349^ | Lung | Prospective cohort study | Pre-post treatment | Evaluate the effect of an intervention | NR | No | NR | 454.5m |
| Brocki, 2015^350^ | Lung | Prospective cohort study | Post treatment | Post-surgery evaluation | NR | Yes | NR | NR |
| Brocki, 2016^351^ | Lung | Prospective cohort study | Pre-post treatment | Evaluate the effect of an intervention | Yes | Yes | NR | Overall: NR  VATS: 429.6m  Mini open: 424.8m |
| Bruera, 2003^352^ | Lung | Prospective cohort study | During treatment | Evaluate the effect of an intervention | NR | Unclear | NR | 508m |
| Cavalheri, 2016 ^136^ | Lung | Cross-sectional | Post treatment | Method study (e.g. VO₂peak vs ISWT) | Yes | Yes | RPE: Yes | 503 m 81 % |
| Chang, 2014^353^ | Lung | Non-randomized intervention study | Post treatment | Evaluate the effect of an intervention | NR | Unclear | NR | NR |
| Coats, 2016^354^ | Lung | Randomized controlled trial | Post treatment | Evaluate the effect of an intervention | NR | No | NR | 509m |
| D’Andrilli, 2016^355^ | Lung | Non-randomized intervention study | Pre-post treatment | Pre-post surgery evaluation  Method study | NR | No | NR | NR |
| Denehy, 2013^356^ | Lung | Prospective cohort study | During treatment | To evaluate the prognostic utility of the exercise capacity | NR | Unclear | NR | NR |
| Deslauriers, 2011^357^ | Lung | Prospective cohort study | Post treatment | Evaluate late toxicity  Evaluate late effects after surgery | NR | No | NR | 421m |
| Dhillion, 2017^358^ | Lung | Prospective cohort study | During treatment | Evaluate the effect of an intervention | No | No | NR | Overall: NR  Intervention: 251m  Control: 235m |
| Doğan, 2020^359^ | Lung | Randomized controlled trial | During treatment | To evaluate the effect of an intervention | NR | No | NR | NR |
| Edbrooke, 2019^360^ multi | Lung | Randomized controlled trial | During treatment | To evaluate the effect of an intervention | NR | Unclear | NR | NR |
| Edbrooke, 2019^361^ | Lung | Randomized controlled trial | During treatment | Method study | NR | No | NR | NR |
| Gao, 2015^363^ | Lung | Non-randomized intervention study | Pre-treatment | Pre surgery evaluation | NR | Unclear | NR | NR |
| Gao, 2024 ^146^ | Lung | Randomized controlled trial | Post-treatment | To evaluate the effect of an intervention | NR | No | NR | NR |
| Glattki, 2012^364^ | Lung | Prospective cohort study | Post treatment | Evaluate the effect of an intervention | NR | Yes | NR | NR |
| Goldsmith, 2021 ^365^ | Lung | Prospective cohort study | Pre-treatment | To evaluate the effect of an intervention | NR | Unclear |  | 306.6 m Intervention: 306.6 m |
| Granger, 2014^366^ | Lung | Prospective cohort study | Pre-post treatment | Pre-post surgery evaluation | Yes | Yes | NR | 407m |
|  |  |  |  |  |  |  |  |  |
| Granger, 2015^367^ | Lung | Prospective cohort study | Pre-treatment | Method study | Other | Yes | NR | 421m |
| Granger, 2015^147^ | Lung | Prospective cohort study | During-post treatment | Method study | Yes | Yes | NR | 487m  499m |
| Granger, 2015^368^ | Lung | Prospective cohort study | Pre-treatment | Method study | NR | No | NR | 415m |
| Granger, 2016^369^ | Lung | Prospective cohort study | Pre-post treatment | Pre-post surgery evaluation | Yes | Yes | NR | 441m |
| Sebio Garcia, 2017^370^ | Lung | Randomized controlled trial | Pre-treatment | To evaluate the effect of an intervention | NR | Unclear | BP: Yes  RPE: Yes | Intervention: 420 m Control: 507.7 m |
| Ha, 2015^371^ | Lung | Retrospective cohort study | Pre-treatment | Pre surgery evaluation | NR | Yes | SpO_2_=94.5 | 395m |
| Ha, 2020^372^ | Lung | Prospective cohort study | Pre-treatment | Pre surgery evaluation | NR | Unclear | NR | 369.7 m 69.4 % |
| Hamadaa, 2019^373^ | Lung | Prospective cohort study | Pre-treatment | Survival | State that familiarization was not given | Yes | NR | 461m |
| Hattori, 2018^374^ | Lung | Retrospective cohort study | Pre-treatment | Pre surgery evaluation | NR | Yes | NR | All: 500m  With pneumonia: 425m  Without: 500m |
| Henke, 2014^375^ | Lung | Randomized controlled trial | During treatment | Evaluate the effect of an intervention | NR | Unclear | NR | NR |
| Huang, 2017^376^ | Lung | Randomized controlled trial | Pre-treatment | Evaluate the effect of an intervention | NR | No | NR | NR |
| Illini, 2022 ^377^ | Lung | One-armed intervention study | Post-treatment | To evaluate the effect of an intervention | NR | Unclear | NR | 472.8 m Other: 522.9 m |
| Irie, 2016^378^ | Lung | Prospective cohort study | Pre-treatment | Pre surgery evaluation | NR | Unclear | NR | NR |
| Jastrzebski, 2015^379^ | Lung | Prospective cohort study | During treatment | Evaluate the effect of an intervention | Yes | Yes | NR | 360.3m |
| Ji, 2019^380^ | Lung | Randomized controlled trial | During treatment | To evaluate the effect of an intervention | NR | No | NR | 433.43m |
| Jones, 2007^152^ | Lung | Other | Pre-treatment | Evaluate the effect of an intervention | NR | Yes | NR | 438m |
| Jones, 2012^381^ | Lung | Prospective cohort study | During treatment | Investigate the prognostic importance of functional capacity | NR | Yes | NR | 424m |
| Jonsson, 2019^382^ | Lung | Randomized controlled trial | Pre-post treatment | To evaluate the effect of an intervention | NR | Yes | NR | NR |
| Kasymjanova, 2009^383^ | Lung | Prospective cohort study | Pre-post treatment | Evaluate acute toxicity < 1-year post-treatment | Yes | Yes | RPE | 462m |
| Katakami, 2018 ^384^ | Lung | Randomized controlled trial | Pre-during treatment | To evaluate the effect of an intervention | NR | No | NR | 376 m |
| Katakami, 2018^384^ | Lung | Randomized controlled trial | During treatment | Evaluate the effect of an intervention | NR | No | NR | Overall: NR  Intervention: 380m  Control: 376m |
| Kong, 2020 ^385^ | Lung | Prospective cohort study | Pre-treatment | Pre surgery evaluation | Yes | Yes | NR | Other: 519.5 m Other: 487.9 m |
| Lai, 2017^386^ | Lung | Randomized controlled trial | Pre-treatment | Evaluate the effect of an intervention | NR | Unclear | NR | 477m |
| Lai, 2019^387^ |  |  |  |  |  |  |  |  |
| Li, 2019^388^ | Lung | Randomized controlled trial | Post treatment | To evaluate the effect of an intervention | NR | No | NR | NR |
| Li, 2024 ^389^ | Lung | Randomized controlled trial | Pre-post treatment | To evaluate the effect of an intervention | NR | No |  | Intervention: 453.28 m Control: 457.89 m |
| Liu, 2020^390^ | Lung | Randomized controlled trial | Pre-treatment | To evaluate the effect of an intervention | NR | Unclear | NR | NR |
| Marjanski, 2015^391^ | Lung | Prospective cohort study | Pre-treatment | Pre surgery evaluation | NR | Yes | NR | 489m |
| Maruyama, 2011^392^ | Lung | Prospective cohort study | Pre-during-post treatment | Method study | NR | No | NR | 521m |
| Menna, 2020^393^ | Lung | Randomized controlled trial | Pre-post treatment | Pre-post surgery evaluation (effect of surgery) | NR | No | NR | NR |
| Miller, 2005^394^ | Lung | Prospective cohort study | Pre-post treatment | Pre-post treatment evaluation other than surgery | NR | Unclear | NR | NR |
| Minnella, 2021 ^167^ | Lung | Retrospective cohort study | Pre-treatment | Pre surgery evaluation | NR | Yes | NR | 468 m |
| Mohan, 2008^395^ | Lung | Prospective cohort study | Pre-post treatment | Pre-post treatment evaluation other than surgery | NR | Unclear | NR | 331m |
| Mohan, 2017^396^ | Lung | Prospective cohort study | Pre-post treatment | Pre-post treatment evaluation other than surgery | NR | Unclear | NR | 503m |
| Morano, 2014^397^ | Lung | Randomized controlled trial | Pre-treatment | Evaluate the effect of an intervention | NR | No | NR | NR |
| Mujovic, 2014^398^ | Lung | Prospective cohort study | pre-during-post treatment | Evaluate the effect of an intervention | NR | Unclear | NR | Overall: NR  All: 500m  With pneumonia: 425m  without: 500m |
| Mujovic, 2015^399^ | Lung | Prospective cohort study | Pre-post treatment | Evaluate the effect of an intervention | NR | Unclear | NR | 322m |
| Nakagawa, 2018^400^ | Lung | Retrospective cohort study | Pre-treatment | Pre surgery evaluation | NR | Unclear | SpO_2_=89.5 | 430m |
| Nakagawa, 2018 ^400^ | Lung | Prospective cohort study | Pre-treatment | Post-surgery evaluation | NR | No | NR |  |
| Nomori, 2001^401^ | Lung | Retrospective cohort study | Pre-post treatment | Pre-post surgery evaluation | NR | Yes | NR | 499m |
| Nomori, 2003^402^ | Lung | Retrospective cohort study | Pre-post treatment | Pre-post surgery evaluation | NR | Yes | NR | Overall: NR  Non-surgery: 400m  Surgery: 411m |
| Parsons, 2003^403^ | Lung | Prospective cohort study | Pre-treatment | Pre surgery evaluation | Yes | No | NR | NR |
| Quist, 2015^404^ | Lung | Prospective cohort study | During treatment | Evaluate the effect of an intervention | NR | Yes | NR | 527.4m |
| Quist, 2018 ^178^ | Lung | Randomized controlled trial | Post treatment | To evaluate the effect of an intervention | NR | No | NR | NR |
| Quist, 2020 ^179^ | Lung | Randomized controlled trial | During treatment | To evaluate the effect of an intervention | NR | No | NR | Intervention: 475 m Control: 443 m |
| Rehman, 2023 ^405^ | Lung | Randomized controlled trial | During treatment | To evaluate the effect of an intervention | NR | No | NR | Intervention: 468.31 m Control: 476.4 m |
| Rick, 2014^406^ | Lung | Retrospective cohort, study | Post treatment | Evaluate the effect of an intervention  Method study | NR | Yes | NR | Overall: NR  Sleeve lobectomy: 482m  Standard lobectomy: 490m |
| Riesenberg, 2010^407^ | Lung | Prospective cohort study | Post treatment | Evaluate the effect of an intervention | NR | Yes | NR | 396m |
| Rosero, 2020^408^ | Lung | Non-randomized controlled study | During treatment | To evaluate the effect of an intervention | No | Unclear | NR | Intervention: 414.7m  Control: 425.7m |
| Rutkowska, 2019^409^ | Lung | Randomized controlled trail | During treatment | To evaluate the effect of an intervention | NR | No | NR | NR |
| Saad, 2006^410^ | Lung | Prospective cohort study | Pre-post treatment | Pre-post surgery evaluation | NR | No | NR | NR |
| Saad, 2007^411^ | Lung | Prospective cohort study | Pre-post treatment | Pre-post surgery evaluation | NR | No | NR | 426.7m |
| Saito, 2020^412^ | Lung | Retrospective cohort study | Pre-post treatment | Pre-post surgery evaluation (effect of surgery) | NR | No | NR | 309m |
| Salhi, 2015^184^ | Lung | Randomized controlled trial | Pre-post treatment | Pre-post surgery evaluation  Evaluate the effect of an intervention | NR | No | NR | NR |
| Shallwani, 2016^413^ | Lung | Prospective cohort study | Pre-post treatment | Pre-post treatment evaluation other than surgery | NR | Unclear | NR | NR |
| Stefanelli, 2013^187^ | Lung | Other | Pre-treatment | Evaluate the effect of an intervention | NR | Yes | NR | 527m |
| Sterzi, 2013^414^ | Lung | Prospective cohort study | Pre-treatment | Evaluate the effect of an intervention | NR | No | NR | 509m |
| Stigt, 2013^415^ | Lung | Prospective cohort study | Pre-post treatment | Evaluate the effect of an intervention | NR | No | NR | NR |
| Suzuki, 2010^416^ | Lung | Randomized controlled trial | Post treatment | Evaluate the effect of an intervention | NR | No | NR | NR |
| Takemura, 2021 ^417^ | Lung | Randomized controlled trial | During treatment | Diagnostic (e.g. hypoxemia, ischemia) | NR | Yes | NR | 403.18 m |
| Tao, 2023 ^418^ | Lung | Randomized controlled trial | Post-treatment | To evaluate the effect of an intervention | NR | Unclear | NR | Intervention: 380.18 m Control: 366.75 m |
| Timmerman, 2018^419^ | Lung | Prospective cohort study | Pre-post treatment | Pre-post surgery evaluation | NR | No | NR | NR |
| Titz, 2018^420^ | Lung | Cross-sectional study | During treatment | To evaluate physical fitness during advanced treatment compared to healthy | NR | Yes | NR | NR |
| Torre-Bouschoulet, 2018^421^ | Lung | Prospective cohort study | Pre-during treatment | Evaluate late toxicity | NR | No | NR | NR |
| Torre-Bouscoulet, 2018^422^ | Lung | Prospective cohort study | Pre-post treatment | Pre-post treatment evaluation other than surgery | NR | Unclear | NR | NR |
| Ueda, 2019^423^ | Lung | Retrospective cohort study | Pre-post treatment | Pre-post surgery evaluation (effect of surgery) | NR | No | NR | 410m |
| Ugalde, 2008^424^ | Lung | Prospective cohort study | Post treatment | Evaluate late toxicity  Evaluate physical function between normal diaphragmatic motion and abnormal motion 5-year post surgery | NR | Unclear | NR | 447m |
| Vagvolgyi, 2018^425^ | Lung | Randomized controlled trial | Pre-during-post treatment | Evaluate the effect of an intervention | NR | Unclear | NR | 393m |
| van der Weijst, 2023 ^426^ | Lung | Prospective cohort study | Pre-post treatment | Evaluate late toxicity > 1-year post-therapy | NR | Unclear |  | 351.1 m |
| Wang, 2013^427^ | Lung | Prospective cohort study | During treatment | Evaluate acute toxicity  Evaluate late toxicity | NR | Unclear | NR | 355m |
| Yi-Jun, 2009^428^ | Lung | Prospective cohort study | Pre-treatment | Pre-post surgery evaluation | NR | No | NR | 274m |
| Yang, 2018^429^ | Lung | quasi-experimental trial with a pre-post-test design | Pre-treatment | Evaluate the effect of an intervention | NR | Yes | NR | NR |
| Zarogoulidis, 2012^430^ | Lung | Prospective cohort study | Pre-during treatment | Pre-post surgery evaluation  Pre-post treatment evaluation other than surgery | NR | Unclear | RPE | 224m |
| Zhang, 2020^431^ | Lung | Randomized controlled trial | During-post treatment | To evaluate the effect of an intervention | NR | No | NR | NR |
| Vermaete, 2014^208^ | Lymphoma | Prospective cohort study | Pre-during-post treatment | Evaluate acute toxicity | NR | Yes | NR | 613m |
| Armstrong, 2013^432^ | Mixed | Prospective cohort study | Post treatment | Evaluate late toxicity | NR | No | NR | NR |
| Bolli, 2020 ^433^ | Mixed | Randomized controlled trial | Pre-post treatment | To evaluate the effect of an intervention | No | No | NR | Intervention: 384.38 m |
| Brown, 2018^434^ | Mixed | Randomized controlled trial | Post treatment | To evaluate the effect of an intervention | NR | Unclear | NR | NR |
| Chemaitilly, 2015^435^ | Mixed | Retrospectove cohort study | Post treatment | Evaluate late toxicity > 1-year post-therapy | NR | Yes | NR | NR |
| Devenney, 2023 ^436^ | Mixed | One-armed intervention study | During treatment | To evaluate the effect of an intervention | NR | No | NR | 474 m Other: 545 m |
| Gagnon, 2013^437^ | Mixed | Non-randomized intervention study | NR | Evaluate the effect of an intervention | NR | Yes | NR | 395m |
| Glare, 2011^438^ | Mixed | Prospective cohort study | During treatment | Evaluate the effect of an intervention | NR | No | NR | 441.5m |
| Green, 2016^439^ | Mixed | Prospective cohort study | Post treatment | Evaluate late toxicity and method study | NR | Yes | NR | NR |
| Hadzibegovic, 2023 ^440^ | Mixed | Prospective cohort study | During treatment | Evaluate acute toxicity < 1-year post-therapy | NR | No | NR | 440 m |
| Hayakawa, 2019^441^ | Mixed | Retrospective cohort study | Pre-post treatment | Evaluate acute toxicity < 1-year post-therapy | NR | No | NR | NR |
| Hui, 2014^442^ | Mixed | Randomized controlled trial | During treatment | Evaluate the effect of an intervention | NR | Yes | NR | Overall: NR  Intervention: 397.7m  Control: 399m |
| Hutchison, 2019^443^ | Mixed | Non-randomized controlled study | During-post treatment | To evaluate the effect of an intervention | NR | No | BP | 399m |
| Irwin, 2017^444^ | Mixed | Randomized controlled trial | Post treatment | Evaluate the effect of an intervention | 6MWT were performed two times | Yes | NR | Overall: NR  Intervention: 485m  Control: 481m |
| Jones, 2014 ^226^ | Mixed | Randomized controlled trial | Post treatment | To evaluate the effect of an intervention | NR | No | NR | Intervention: 358 m Control: 356 m |
| Kimberg, 2015^445^ | Mixed | Cross-sectional study | Post treatment | Evaluate late toxicity | NR | No | NR | NR |
| Kirkham, 2016^446^ | Mixed | Retrospective cohort study | Post treatment | Evaluate the effect of an intervention | NR | Yes | NR | 527.6m |
| Leach, 2018^447^ | Mixed | Non-randomized intervention study | During-post treatment | Other: To design a rehab program (only if no follow-up) | NR | Unclear | BP | 409m |
| Lezin, 2019^448^ | Mixed | Non-randomized intervention study | Two groups: one during treatment and one post treatment | To evaluate the effect of an intervention | NR | Unclear | Resting before start | No treatment group: 266m  Treatment group: 323m |
| Al-Mozaini, 2022 ^449^ | Mixed | Cross-sectional study | Pre-treatment | Covid-19 | NR | Unclear | NR | 491 m |
| Madeddu, 2012^450^ | Mixed | Randomized controlled trial | During treatment | Evaluate the effect of an intervention | NR | Yes | NR | Overall: NR  Intervention: 429m  Control: 411m  NR |
| Mangia, 2017^451^ | Mixed | Prospective cohort study | During treatment | Method study | NR | Unclear | NR | 438m |
| Marker, 2024 ^452^ | Mixed | One-armed intervention study | Post-treatment | To evaluate the effect of an intervention | NR | Unclear | NR | 583.4 m Other: 642.8 m |
| McCrary, 2019^453^ | Mixed | Prospective cohort study | Post treatment | Evaluate late toxicity > 1-year post-therapy | NR | Unclear | NR | 425.5m |
| Mikkelsen, 2022 ^454^ | Mixed | Randomized controlled trial | During treatment | To evaluate the effect of an intervention | NR | No | NR | Intervention: 463.7 m Control: 434.4 m |
| Morishita, 2013^455^ | Mixed | Non-randomized intervention study | During treatment | Evaluate the effect of an intervention | NR | Yes | NR | Overall: NR  High-frequency physio: 484.5m  Low-frequency physio:519.4m |
| Morishita, 2015^341^ | Mixed | Prospective cohort study | Pre-post treatment | Pre-post treatment evaluation other than surgery | NR | Yes | NR | 494,7m |
| Morris, 2009^456^ | Mixed | One-armed intervention study | Post treatment | Evaluate the effect of an intervention | NR | Yes | NR | 360.1m |
| Mulrooney, 2016^457^ | Mixed | Cross-sectional study | Pre-post treatment | Evaluate late toxicity | NR | No | NR | NR |
| Ngo-Huang, 2021 ^458^ | Mixed | One-armed intervention study | During treatment | Evaluate acute toxicity < 1-year post-therapy | NR | No | NR | 354 m |
| Noh, 2023 ^459^ | Mixed | Prospective cohort study | Post-treatment | Covid19 | NR | No | NR | 373 m |
| Oviatt, 2011^460^ | Mixed | Prospective cohort study | During treatment | To evaluate the effect of a procedure (pulmonary stent) | NR | No | NR | 195.7m |
| Parmar, 2017^461^ | Mixed | Retrospective cohort study | During treatment | Evaluate the effect of an intervention | NR | No | NR | 352m |
| Potiaumpai, 2021 ^462^ | Mixed | Randomized controlled trial | Pre-post treatment | To evaluate the effect of an intervention | NR | No | NR | NR |
| Rossi, 2016 ^463^ | Mixed | Randomized controlled trial | Post treatment | To evaluate the effect of an intervention | NR | Unclear | RPE: Yes | Intervention: 431 m Control: 427 m |
| Santa Mina, 2017^464^ | Mixed | Prospective cohort study | Post treatment | Evaluate the effect of an intervention | NR | Yes | NR | 439.7m |
| Schmidt, 2013 ^246^ | Mixed | Prospective cohort study | During treatment | Method study (e.g. VO₂peak vs ISWT) | NR | Yes |  | 594 m |
| Schuler, 2017^465^ | Mixed | Randomized controlled trial | During treatment | Evaluate the effect of an intervention | NR | No | NR | Overall: NR  Intervention: 517.8m  Control: 539.5m |
| Schumacher, 2019 ^466^ | Mixed | Cross-sectional study | Some during treatment and some post | Method study (e.g. VO₂peak vs ISWT) | NR | Yes | BP: Yes RPE: Yes | 485 m |
| Slater, 2015^467^ | Mixed | Case-control study | Post treatment | Evaluate late toxicity | NR | Unclear | NR | Overall: NR  Survivors: 583.1m  Controls: 591.9m |
| Smith, 2014^468^ | Mixed | Prospective cohort study | Post treatment | Method study | NR | Yes | NR | NR |
| Sturm, 2014^469^ | Mixed | Non-randomized controlled trial | During treatment | Evaluate the effect of an intervention | NR | No | NR | NR |
| Swenson, 2014^470^ | Mixed | Non-randomized intervention study | Pre-during-post treatment | Evaluate the effect of an intervention | NR | Yes | NR | 418m |
| Tran, 2016^471^ | Mixed | Prospective cohort study | Post treatment | Evaluate the effect of an intervention | NR | Yes | NR | 371.4m |
| Uster, 2018^472^ | Mixed | Randomized controlled trial | During treatment | Evaluate the effect of an intervention | NR | Yes | NR | NR |
| Waterland, 2022 ^252^ | Mixed | Prospective cohort study | Pre-treatment | Pre surgery evaluation | NR | Unclear | NR | NR |
| Coleman, 2008^473^ | Multiple myeloma | Randomized controlled trial | During treatment | Evaluate the effect of an intervention | NR | No | NR | NR |
| Larsen, 2020 ^474^ | Multiple myeloma | Cross-sectional study | Pre-treatment | Pre therapy assessment | NR | No | NR | NR |
| An, 2019^475^ | Pancreatic | Non-randomized intervention study | Pre-during treatment | To evaluate the effect of an intervention | NR | No | NR | 462.5m |
| Stene, 2019^476^ | Pancreatic cancer | Randomized controlled trial | During treatment | Method study (e.g. VO2peak vs ISWT) | NR | Unclear | NR | 481.6m |
| Wiedenmann, 2008^477^ | Pancreatic | Randomized controlled trial | During treatment | Evaluate the effect of an intervention | NR | No | NR | NR |
| Alibhai, 2010^478^ | Prostate | Prospective cohort study | Pre-during treatment | Evaluate acute toxicity | NR | Unclear | NR | 471m |
| Alibhai, 2015^479^ | Prostate | Matched prospective cohort study | Pre-during treatment | Evaluate acute toxicity  Evaluate late toxicity | NR | Unclear | NR | NR |
| Au, 2019^480^ | Prostate | Randomized controlled trial | Pre-post treatment | To evaluate the effect of an intervention | NR | No | NR | NR |
| Harrison, 2022 ^268^ | Prostate | Randomized controlled trial | Pre-during treatment | To evaluate the effect of an intervention | NR | No | NR | NR |
| Hojan, 2017^269^ | Prostate | Randomized controlled trial | Pre-during treatment | Evaluate the effect of an intervention | NR | Unclear | NR | NR |
| Mareschal, 2017^481^ | Prostate | Non-randomized intervention study | Pre-during treatment | Evaluate the effect of an intervention | NR | Yes | NR | 487.0m |
| O’Neill, 2015^482^ | Prostate | Randomized controlled trial | During treatment | Evaluate the effect of an intervention | NR | No | NR | Overall: NR  Intervention: 515.5m  Control: 470.1m |
| Santa Mina, 2018^483^ | Prostate | Randomized controlled trial | Pre-treatment | To evaluate the effect of an intervention | NR | No | NR | NR |
| Villumsen, 2019^484^ | Prostate | Randomized controlled trial | During treatment | To evaluate the effect of an intervention | NR | No | NR | NR |
| Bishop, 2020^485^ | Sarcoma | Case-control study | Post treatment | Evaluate late toxicity > 1-year post-therapy | NR | No | NR | NR |
| Morri, 2018 ^486^ | Sarcoma | Prospective cohort study | During-post treatment | To describe the rehabilitative pathway, ref values of the functional results | NR | Unclear | NR | 450 m |
| Van Egmond-van Dam, 2017^487^ | Sarcoma | Prospective cohort study | Post treatment | Evaluate late toxicity | NR | No | NR | 513.5m |

**Table S3.** Peak Oxygen Consumption and Six-Minute Walk Distance Summary Results.

|  | CPET | | | |  | 6MWT | | | |
| --- | --- | --- | --- | --- | --- | --- | --- | --- | --- |
|  | Number of Studies | % | VO_2_peak  (mLˑkg^-1^ˑmin^-1^) | SD |  | Number of Studies | % | 6MWD (m) | SD |
| Overall | 204 | 100 | 21.8 | 5.8 |  | 152 | 100 | 445 | 79 |
| Bladder | 4 | 2.0 | 19.1 | 5 |  | 0 | 0 | N/A | N/A |
| Breast | 41 | 20.1 | 22.3 | 4.2 |  | 18 | 11.8 | 500 | 79 |
| Colon/rectum | 12 | 5.9 | 19.9 | 3.4 |  | 5 | 3.3 | 485 | 68 |
| Esophageal/Gastric | 16 | 7.8 | 20 | 4.7 |  | 9 | 5.9 | 440 | 76 |
| Leukemia/ Lymphoma/Multiple Myeloma | 17 | 8.3 | 27.1 | 6.1 |  | 12 | 7.9 | 477 | 98 |
| Lung | 53 | 26.0 | 17.7 | 2.6 |  | 61 | 40.1 | 424 | 73 |
| Mixed | 43 | 21.1 | 23.9 | 6.7 |  | 33 | 21.7 | 432 | 81 |
| Prostate | 14 | 6.9 | 26.7 | 3.9 |  | 3 | 2.0 | 483 | 21 |
| Other | 4* | 2.0 | 25 | 12.5 |  | 11** | 7.2 | 440 | 52 |

**Abbreviation:** CPET; cardiopulmonary exercise test; 6MWT, Six-minute walk test; VO_2_peak; peak oxygen consumption; MWD, minute walk distance; SD, standard deviation.

* <4 studies by cancer type: Liver, Ovarian, Testicular, Pancreatic

**<4 studies by cancer type: Brain, Head/neck, Liver, Pancreatic, Sarcoma

### Table S4. Exercise Test Preparation and Safety Characteristics

| Study | Cancer site | Participants  (n) | Comorbidities reported | Effort during test | Pre-test preparation | Who performed  the test? | Safety Monitoring  (HR, ECG, BP, SpO_2_) | Presence of test-related AE | Number of AE reported (n) | Type of AE |
| --- | --- | --- | --- | --- | --- | --- | --- | --- | --- | --- |
| Banerjee, 2018^1^ | Bladder | 60 | Yes | Maximal | NR | NR | Yes | NR | NR | NR |
| Blackwell, 2020^488^ | Bladder | 40 | No | Maximal | NR | NR | NR | NR | NR | NR |
| Lamb, 2016^2^ | Bladder | 82 | No | Maximal | NR | NR | Yes | NR | NR | NR |
| Longdon, 2020^3^ | Bladder | 141 | Yes | Maximal | NR | Physician | No | NR | NR | NR |
| Minnella, 2019^281^ | Bladder | 70 | Yes | Submaximal | NR | NR | NR | NR | NR | NR |
| Tolchard, 2015^4^ | Bladder | 105 | No | Maximal | NR | Technician and anesthetist | Yes | NR | NR | NR |
| van Egmond-van Dam, 2017^487^ | Sarcoma | 20 | No | Submaximal | NR | NR | NR | NR | NR | NR |
| Morri, 2018^486^ | Sarcoma | 30 | No | Submaximal | NR | NR | NR | NR | NR | NR |
| Pieczynska, 2022^282^ | Brain | 29 | No | Submaximal | NR | NR | Yes | NR | NR | NR |
| Pieczynska, 2023^283^ | Brain | 33 | No | Submaximal | NR | Physiotherapist | NA | NR | NR | NR |
| Ruden, 2011^489^ | Brain | 243 | No | Submaximal | NR | Exercise physiologist/sport scientists | NR | No | 0 | NA |
| Alizadeh, 2019^490^ | Breast | 52 | No | Submaximal | NR | NR | NA | NR | NR | NR |
| An, 2020^5^ | Breast | 264 | No | Maximal | NR | NR | NA | NR | NR | NR |
| Antunes, 2023^491^ | Breast | 93 | Yes | Maximal | NR | NR | NA | NR | NR | NR |
| Ariza-Garcia, 2019^285^ | Breast | 68 | No | Submaximal | Yes | NR | NA | NR | NR | NR |
| Bell, 2021^6^ | Breast | 20 | No | Maximal | NR | NR | Yes | NR | NR | NR |
| Bellissimo,, 2023^286^ | Breast | 223 | Yes | Submaximal | NR | Trained professionals | NA | NR | NR | NR |
| Bender, 2021^7^ | Breast | 73 | No | Submaximal | NR | NR | No | NR | NR | NR |
| Bender, 2024^8^ | Breast | 153 | No | Submaximal | NR | NR | No | NR | NR | NR |
| Berling-Ernst, 2022^9^ | Breast | 46 | No | Maximal | NR | NR | No | NR | NR | NR |
| Bigaran, 2022^10^ | Breast | 20 | No | Maximal | NR | NR | Yes | NR | NR | NR |
| Bohn, 2021^11^ | Breast | 55 | No | Maximal | NR | NR | No | NR | NR | NR |
| Bonsignore, 2017^12^ | Breast | 29 | Yes | Maximal | NR | Test staff | Yes | NR | NR | NR |
| Bonsignore,, 2021^13^ | Breast | 147 | Yes | Maximal | NR | Exercise physiologist | Yes | NR | NR | NR |
| Brahmbhatt, 2020^287^ | Breast | 22 | No | Submaximal | NR | NR | NA | NR | NR | NR |
| Brown,, 2023^492^ | Breast | 320 | No | Submaximal | No | NR | NA | NR | NR | NR |
| BUCHAN, 2016^288^ | Breast | 42 | No | Submaximal | NR | NR | NA | NR | NR | NR |
| Burnett, 2013^14^ | Breast | 30 | Yes | Both maximal and submaximal | Yes | Exercise physiologist | Yes | No | 0 | NA |
| Casla, 2015^493^ | Breast | 94 | No | Submaximal | NR | NR | NA | NR | NR | NR |
| CEŠEIKO, 2020^289^ | Breast | 55 | No | Both maximal and submaximal | Yes | NR | No | NR | NR | NR |
| Chap, 1997^494^ | Breast | 64 | No | Submaximal | NR | NR | NA | NR | NR | NR |
| Cheema, 2006^15^ | Breast | 27 | No | Maximal | NR | NR | NA | NR | NR | NR |
| Chung, 2022^495^ | Breast | 32 | Yes | Maximal | NR | NR | No | NR | NR | NR |
| Cornette, 2016^17^ | Breast | 42 | No | Both maximal and submaximal | Performed two 6MWT | Respiratory physiologist | Yes | NR | NR | NR |
| Courneya, 2003^18^ | Breast | 52 | No | Maximal | NR | NR | Yes | No | 0 | NA |
| Courneya, 2007^19^ | Breast | 242 | Yes | Maximal | NR | NR | NA | NR | NR | NR |
| Courneya, 2008 ^20^ | Breast | 242 | No | Maximal | NR | NR | NA | NR | NR | NR |
| Courneya, 2014^21^ | Breast | 301 | No | Maximal | NR | NR | NA | NR | NR | NR |
| Delrieu, 2020^496^ | Breast | 51 | No | Submaximal | NR | NR | NA | NR | NR | NR |
| Diaz-Balboa, 2022^22^ | Breast | 120 | Yes | Maximal | NR | Physiotherapist and cardiologist | Yes | NR | NR | NR |
| Dieli-Conwright, 2018^497^ | Breast | 100 | No | Submaximal | NR | NR | NA | NR | NR | NR |
| Do, 2015^498^ | Breast | 62 | No | Submaximal | NR | NR | NA | NR | NR | NR |
| Dolan, 2010^23^ | Breast | 242 | No | Maximal | NR | NR | NA | NR | NR | NR |
| Elme, 2013^499^ | Breast | 494 | Yes | Submaximal | NR | NR | NA | NR | NR | NR |
| Farajivaf, 2023^500^ | Breast | 89 | No | Submaximal | NR | NR | NA | NR | NR | NR |
| Foulkes, 2023^24^ | Breast | 104 | Yes | Maximal | NR | NR | No | NR | NR | NR |
| Franca-Lara, 2023^501^ | Breast | 107 | No | Submaximal | NR | NR | NA | NR | NR | NR |
| Friedenreich, 2022^25^ | Breast | 1528 | No | Maximal | NR | Exercise physiologist | Yes | NR | NR | NR |
| Giallauria, 2015^502^ | Breast | 51 | No | Maximal | NR | NR | Yes | NR | NR | NR |
| Groarke, 2019^503^ | Breast | 896 | Yes | Maximal | NR | NR | Yes | NR | NR | NR |
| Gustavsson, 1999^504^ | Breast | 90 | Yes | Maximal | NR | NR | Yes | NR | NR | NR |
| Gyenes, 1994^505^ | Breast | 37 | No | Maximal | NR | NR | Yes | NR | NR | NR |
| Heim, 2007^506^ | Breast | 63 | No | Submaximal | NR | NR | NA | NR | NR | NR |
| Hojan, 2020^290^ | Breast | 47 | Yes | Submaximal | NR | NR | NA | NR | NR | NR |
| Hokken, 2009^27^ | Breast | 75 | No | Maximal | NR | Exercise physiologist/sport scientists | NA | NR | NR | NR |
| Hughes, 2015^28^ | Breast | 94 | No | Submaximal | NR | Exercise physiologist/sport scientists | Yes | NR | NR | NR |
| Isanejad, 2023^29^ | Breast | 30 | No | Maximal | NR | NR | Yes | No | 0 |  |
| Jacquinot, 2022^30^ | Breast | 89 | No | Maximal | NR | Trained professionals | Yes | NR | NR | NR |
| Joaquim, 2023^507^ | Breast | 37 | Yes | Maximal | NR | NR | NA | NR | NR | NR |
| Jones, 2012 ^31^ | Breast | 248 | No | Maximal | NR | NR | Yes | NR | NR | NR |
| Jones, 2020^508^ | Breast | 51 | No | Submaximal | NR | NR | NA | NR | NR | NR |
| Kaltsatou, 2011^291^ | Breast | 27 | No | Submaximal | NR | NR | Yes | NR | NR | NR |
| Khan, 2020 | Breast | 60 | No | NR | NR | NR | NA | NR | NR | NR |
| Khouri, 2014^32^ | Breast | 77 | Yes | Maximal | NR | NR | Yes | NR | NR | NR |
| Kiecolt-Glaser, 2022^33^ | Breast | 158 | Yes | Maximal | NR | NR | No | NR | NR | NR |
| Kim, 2006^34^ | Breast | 41 | No | Maximal | NR | Exercise physiologist/sport scientists/physician | Yes | NR | NR | NR |
| Kim, 2019^292^ | Breast | 94 | No | Submaximal | NR | NR | NA | NR | NR | NR |
| Kirkham, 2020^35^ | Breast | 78 | No | Submaximal | NR | NR | NA | NR | NR | NR |
| Klassen, 2014^36^ | Breast | 222 | No | Maximal | NR | NR | Yes | Yes | 18 | Excessive increase in BP, ECG abnormalities, severe dyspnea, orthopedic problems |
| Koevoets, 2022^37^ | Breast | 181 | Yes | Maximal | NR | NR | Yes | NR | NR | NR |
| Koevoets, 2023^38^ | Breast | 104 | No | Maximal | NR | NR | Yes | NR | NR | NR |
| Kokkonen, 2017^293^ | Breast | 128 | No | Submaximal | NR | NR | NA | NR | NR | NR |
| Kulkarni, 2013^294^ | Breast | 54 | No | Submaximal | NR | NR | NA | NR | NR | NR |
| Lahart, 2017^39^ | Breast | 32 | Yes | Maximal | NR | NR | Yes | NR | NR | NR |
| Lakoski, 2013^509^ | Breast | 180 | No | Maximal | NR | Physician | NA | NR | NR | NR |
| Ligibel, 2016^510^ | Breast | 98 | No | Maximal | NR | NR | NA | NR | NR | NR |
| MacVicar, 1989^40^ | Breast | 45 | No | Maximal | NR | NR | Yes | NR | NR | NR |
| Madison, 2023^41^ | Breast | 80 | No | Maximal | NR | Exercise physiologist | NA | NR | NR | NR |
| Mehnert, 2011^42^ | Breast | 58 | No | Maximal | NR | NR | NA | NR | NR | NR |
| Mijwel, 2018 ^511^ | Breast | 240 | No | Submaximal | NR | NR | NA | NR | NR | NR |
| Mijwel, 2019^512^ | Breast | 206 | No | Submaximal | NR | NR | NA | NR | NR | NR |
| Moghadam, 2021 ^43^ | Breast | 45 | No | Maximal | Yes | NR | No | NR | NR | NR |
| Murtezani, 2014^513^ | Breast | 36 | No | Submaximal | NR | NR | NA | NR | NR | NR |
| Musanti, 2012^514^ | Breast | 55 | No | Submaximal | NR | Research assistant | NA | NR | NR | NR |
| Mustian, 2008^295^ | Breast | 21 | No | Submaximal | NR | ACSM certified fitness instructor | NA | NR | NR | NR |
| Mutrie, 2007^515^ | Breast | 92 | No | Submaximal | NR | NR | NA | NR | NR | NR |
| Natalucci, 2021^516^ | Breast | 30 | No | Submaximal | NR | NR | NA | NR | NR | NR |
| Navarro-Sanz, 2018^517^ | Breast | 53 | No | Maximal | NR | NR | Yes | No | 0 | NA |
| Nuri, 2012^44^ | Breast | 29 | No | Maximal | NR | NR | Yes | NR | NR | NR |
| Ochi, 2022^45^ | Breast | 50 | No | Maximal | NR | NR | No | NR | NR | NR |
| Okumatsu, 2019^518^ | Breast | 32 | Yes | Maximal | NR | NR | NA | NR | NR | NR |
| Ortiz, 2018^296^ | Breast | 89 | No | Submaximal | NR | NR | NA | NR | NR | NR |
| Ortiz, 2021^297^ | Breast | 89 | No | Submaximal | No | NR | NA | NR | NR | NR |
| Pagola, 2020^46^ | Breast | 23 | No | Maximal | Yes | NR | Yes | NR | NR | NR |
| Peck, 2022^47^ | Breast | 136 | Yes | Maximal | NR | Exercise physiologist | Yes | NR | NR | NR |
| Penttinen, 2011^519^ | Breast | 537 | Yes | Submaximal | NR | NR | NA | NR | NR | NR |
| Prieto-Gόmez, 2022^298^ | Breast | 80 | No | Submaximal | NR | NR | NA | No | 0 | NA |
| Rabin, 2006 | Breast | 86 | No | Submaximal | NR | NR | NA | NR | NR | NR |
| Reis, 2013^299^ | Breast | 41 | No | Submaximal | NR | NR | NA | NR | NR | NR |
| Reis, 2021^520^ | Breast | 23 | No | Submaximal | NR | NR | NA | NR | NR | NR |
| Reis, 2023^521^ | Breast | 26 | No | Submaximal | NR | NR | NA | NR | NR | NR |
| Rogers, 2015^522^ | Breast | 222 | No | Submaximal | NR | NR | NA | NR | NR | NR |
| Romero-Elias, 2023^523^ | Breast | 84 | No | Submaximal | NR | NR | NA | NR | NR | NR |
| Saarto, 2012^524^ | Breast | 500 | No | Submaximal | NR | NR | NA | NR | NR | NR |
| Samhan, 2021 ^525^ | Breast | 60 | No | Maximal | NR | NR | Yes | NR | NR | NR |
| Santagnello, 2020^300^ | Breast | 20 | Yes | Submaximal | Performed two 6MWT | NR | NA | NR | NR | NR |
| Scharhag-Rosenberger, 2015^48^ | Breast | 52 | No | Maximal | Yes | NR | Yes | NR | NR | NR |
| Schmidt, 2015^526^ | Breast | 26 | No | Maximal | NR | NR | Yes | NR | NR | NR |
| Schwartz, 2000^527^ | Breast | 31 | No | Submaximal | NR | NR | NA | NR | NR | NR |
| Scott, 2013^528^ | Breast | 90 | No | Submaximal | NR | NR | NA | NR | NR | NR |
| Scott, 2018^49^ | Breast | 65 | No | Both maximal and submaximal | NR | Dedicated study-personnel with degree | Yes | Yes | 1 | ECG abnormalities |
| Scott, 2023^50^ | Breast | 158 | Yes | Maximal | NR | NR | Yes | NR | NR | NR |
| Segal, 2001^529^ | Breast | 123 | No | NR | NR | NR | NA | NR | NR | NR |
| Siripanya, 2023^51^ | Breast | 22 | No | Maximal | NR | NR | No | NR | NR | NR |
| Smoot, 2014^52^ | Breast | 133 | No | Maximal | NR | NR | Yes | NR | NR | NR |
| Soriano-Maldonado, 2023^530^ | Breast | 60 | No | Submaximal | NR | NR | NA | NR | NR | NR |
| Stalsberg, 2022^531^ | Breast | 47 | No | Maximal | NR | NR | No | NR | NR | NR |
| Suesada, 2018^53^ | Breast | 37 | No | Maximal | NR | NR | Yes | NR | NR | NR |
| Swisher, 2015^54^ | Breast | 28 | No | Maximal | NR | NR | NA | NR | NR | NR |
| Travier, 2015^55^ | Breast | 204 | No | Maximal | NR | NR | NA | NR | NR | NR |
| Tubiana-Mathieu, 2021^56^ | Breast | 138 | Yes | Maximal | NR | Respiratory physiologist | Yes | NR | NR | NR |
| Uth, 2020^57^ | Breast | 68 | No | Maximal | NR | NR | No | No | 0 | NA |
| van Waart, 2015^532^ | Breast | 230 | No | NR | NR | NR | NA | NR | NR | NR |
| Vardar Yagli, 2015 ^533^ | Breast | 21 | No | Submaximal | NR | NR | Yes | NR | NR | NR |
| Vardar-Yagli, 2015 ^301^ | Breast | 40 | No | Submaximal | NR | NR | NA | NR | NR | NR |
| Vincent, 2020^58^ | Breast | 94 | Yes | Maximal | NR | NR | Yes | NR | NR | NR |
| Wang, 2011^302^ | Breast | 72 | No | Submaximal | NR | NR | NA | NR | NR | NR |
| Winters-Stone, 2022^303^ | Breast | 114 | No | Submaximal | NR | NR | NA | NR | NR | NR |
| Zvinovski, 2021^59^ | Breast | 24 | No | Maximal | Yes | Trained professionals | Yes | No | 0 | NA |
| Leensen, 2017^60^ | Breast and Colon | 93 | No | Maximal | NR | Physician | NR | NR | NR | NR |
| Schwartz, 2009^534^ | Breast and Colon | 101 | No | Submaximal | NR | NR | NR | No | 0 | NA |
| Martin, 2015^61^ | Breast and prostate | 159 | No | Maximal | NR | NR | No | NR | NR | NR |
| Schneider, 2020^535^ | Breast and prostate | 40 | No | Maximal | Yes | Exercise physiologist | Yes | NR | NR | NR |
| Schneider, 2020^62^ | Breast and prostate | 40 | No | Maximal | Verification test performed | NR | NR | NR | NR | NR |
| Alexander, 2017^536^ | Colon/rectum | 181 | No | NR | NR | NR | NA | NR | NR | NR |
| Berkel, 2022^63^ | Colon/rectum | 57 | Yes | Maximal | NR | NR | Yes | NR | NR | NR |
| Bolshinsky, 2022^64^ | Colon/rectum | 82 | No | Maximal | Yes | Trained professionals | Yes | NR | NR | NR |
| Bousquet-Dion, 2018^304^ | Colon/rectum | 63 | Yes | Submaximal | Yes | NR | NA | NR | NR | NR |
| Challand, 2012^65^ | Colon/rectum | 179 | No | Submaximal | NR | NR | NA | NR | NR | NR |
| Chan, 2015 ^66^ | Colon/rectum | 48 | No | Maximal | NR | NR | Yes | NR | NR | NR |
| Chen, 2017^305^ | Colon/rectum | 116 | No | Submaximal | NR | NR | NA | NR | NR | NR |
| Chen, 2020^306^ | Colon/rectum | 42 | No | Submaximal | NR | NR | NA | NR | NR | NR |
| Christensen, 2019^67^ | Colon/rectum | 39 | No | Maximal | NR | NR | NA | NR | NR | NR |
| Courneya, 2016^307^ | Colon/rectum | 211 | No | Submaximal | NR | NR | NA | NR | NR | NR |
| Cramer, 2014^68^ | Colon/rectum | 50 | No | Maximal | NR | NR | NA | NR | NR | NR |
| Cuijpers, 2022^537^ | Colon/rectum | 256 | No | Maximal | NR | NR | NA | NR | NR | NR |
| Devin, 2016^69^ | Colon/rectum | 47 | No | Maximal | Yes | NR | Yes | Yes | 1 | Post-exercise hypotension |
| Exarchou, 2020^70^ | Colon/rectum | 146 | No | Maximal | Yes | NR | Yes | NR | NR | NR |
| Franssen, 2024^71^ | Colon/rectum | 102 | No | Maximal | Yes | Exercise physiologist | Yes | NR | NR | NR |
| Gillis, 2014^308^ | Colon/rectum | 77 | Yes | Submaximal | NR | NR | NA | NR | NR | NR |
| Heitkamp, 2023^72^ | Colon/rectum | 50 | No | Maximal | NR | NR | No | NR | NR | NR |
| Heldens, 2017^538^ | Colon/rectum | 75 | No | Maximal | NR | Physiotherapist | NA | NR | NR | NR |
| Heldens, 2021^539^ | Colon/rectum | 25 | Yes | Maximal | NR | NR | NA | NR | NR | NR |
| Hossain, 2020^73^ | Colon/rectum | 61 | No | Maximal | NR | NR | NA | NR | NR | NR |
| Kindred, 2018^540^ | Colon/rectum | 46 | No | Submaximal | NR | NR | NA | NR | NR | NR |
| Lee, 2013 ^74^ | Colon/rectum | 112 | Yes | Both maximal and submaximal | NR | NR |  | NR | NR | NR |
| Lee, 2013 ^541^ | Colon/rectum | 191 | No | Submaximal | NR | NR | NA | NR | NR | NR |
| Liedman, 1995^542^ | Colon/rectum | 213 | Yes | Maximal | NR | A staff member | Yes | Yes | Unclear | Unclear |
| Loughney, 2021^75^ | Colon/rectum | 33 | Yes | Submaximal | NR | NR | No | NR | NR | NR |
| Minnella, 2020^76^ | Colon/rectum | 42 | Yes | Maximal | NR | NR | Yes | NR | NR | NR |
| Morielli, 2021^77^ | Colon/rectum | 36 | Yes | Maximal | NR | NR | No | NR | NR | NR |
| Nakamura, 2019^543^ | Colon/rectum | 119 | No | Submaximal | NR | Physician and trained assistance | NA | NR | NR | NR |
| Norager, 2006^544^ | Colon/rectum | 193 | No | Maximal | NR | NR | No | NR | NR | NR |
| Northgraves, 2020^309^ | Colon/rectum | 21 | No | Submaximal | No | NR | NA | NR | NR | NR |
| Nuri, 2016^545^ | Colon/rectum | 30 | No | Submaximal | NR | NR | NA | NR | NR | NR |
| Pecorelli, 2015^310^ | Colon/rectum | 174 | No | Submaximal | NR | NR | NA | NR | NR | NR |
| Pinto, 2013^78^ | Colon/rectum | 46 | No | Submaximal | NR | NR | NA | NR | NR | NR |
| Rose, 2018^79^ | Colon/rectum | 213 | Yes | Maximal | CPET performed 3 times in healthy group | NR | Yes | NR | NR | NR |
| Sellar, 2014^80^ | Colon/rectum | 29 | No | Maximal | NR | NR | Yes | No | 0 | NA |
| Soares-Miranda, 2021^311^ | Colon/rectum | 71 | No | Submaximal | No | NR | NA | NR | NR | NR |
| Soumya, 2022^546^ | Colon/rectum | 46 | Yes | Maximal | NR | Advanced cardiac life support trained person | NA | No | 0 | NA |
| Wang, 2023^312^ | Colon/rectum | 587 | No | Submaximal | NR | NR | NA | NR | NR | NR |
| West, 2014 ^81^ | Colon/rectum | 136 | Yes | Maximal | NR | An assessor | Yes | Yes | 1 | Supraventricular tachycardia at peak exercise |
| West, 2014 ^82^ | Colon/rectum | 25 | No | Maximal | NR | NR | Yes | NR | NR | NR |
| West, 2014 ^83^ | Colon/rectum | 105 | Yes | Maximal | NR | NR | Yes | NR | NR | NR |
| Wilson, 2019^84^ | Colon/rectum | 1375 | Yes | Submaximal | NR | NR | NA | NR | NR | NR |
| Zimmer, 2018^313^ | Colon/rectum | 30 | No | Submaximal | NR | Exercise physiologist/sport scientists | NA | NR | NR | NR |
| Li, 2022^314^ | Colon/rectum and Gastric | 878 | Yes | Submaximal | NR | NR | NA | NR | NR | NR |
| Tufo, 2018^85^ | Colon/rectum and liver | 161 | Yes | Maximal | NR | NR | NR | NR | NR | NR |
| Astrup Søndergaard, 2022^86^ | Esophageal cancer | 47 | Yes | Maximal | NR | NR | Yes | NR | NR | NR |
| Benington, 2019^87^ | Esophageal cancer | 200 | Yes | Maximal | NR | NR | Yes | NR | NR | NR |
| Chang, 2020 ^547^ | Esophageal cancer | 88 | No | Both maximal and submaximal | NR | NR | Yes | NR | NR | NR |
| Doganay, 2020^548^ | Esophageal cancer | 39 | No | Submaximal | NR | NR | NA | NR | NR | NR |
| Drummond, 2018^89^ | Esophageal cancer | 42 | No | Maximal | NR | Physician | Yes | NR | NR | NR |
| Eriksson, 2021^315^ | Esophageal cancer | 109 | No | Submaximal | NR | Physiotherapist | NA | NR | NR | NR |
| Forshaw, 2008^90^ | Esophageal cancer | 78 | No | Maximal | NR | Physician | Yes | NR | NR | NR |
| Gannon, 2017^549^ | Esophageal cancer | 50 | No | Maximal | NR | NR | NA | NR | NR | NR |
| Guinan, 2018^316^ | Esophageal cancer | 36 | No | Submaximal | NR | NR | NA | NR | NR | NR |
| Guinan, 2019^317^ | Esophageal cancer | 52 | No | Submaximal | NR | NR | NA | NR | NR | NR |
| Inoue, 2016^318^ | Esophageal cancer | 34 | No | Submaximal | NR | NR | NA | NR | NR | NR |
| Jack, 2014^91^ | Esophageal cancer | 89 | No | Maximal | NR | NR | Yes | NR | NR | NR |
| Lam, 2019^92^ | Esophageal cancer | 206 | No | Maximal | NR | NR | NA | NR | NR | NR |
| Liedman, 2001^550^ | Esophageal cancer | 39 | Yes | Maximal | NR | NR | Yes | NR | NR | NR |
| Minnella, 2018^319^ | Esophageal cancer | 51 | Yes | Submaximal | NR | NR | NA | NR | NR | NR |
| Moyes, 2013^93^ | Esophageal cancer | 108 | No | Maximal | NR | NR | Yes | NR | NR | NR |
| Navidi, 2018^94^ | Esophageal cancer | 31 | No | Maximal | Yes | Exercise physiologist | Yes | NR | NR | NR |
| O'Neill, 2018^95^ | Esophageal cancer | 43 | No | Maximal | NR | Physiotherapist and physician | Yes | Yes | 1 | Atrial fibrillation |
| Otani, 2020^320^ | Esophageal cancer | 52 | Yes | Submaximal | NR | NR | NA | NR | NR | NR |
| Ozova, 2022^96^ | Esophageal cancer | 200 | Yes | Maximal | No | NR | No | NR | NR | NR |
| Patel, 2019^97^ | Esophageal cancer | 120 | No | Maximal | NR | Physiologists | Yes | NR | NR | NR |
| Sinclair, 2017^98^ | Esophageal cancer | 240 | Yes | Maximal | NR | NR | Yes | NR | NR | NR |
| Sugimura, 2022^321^ | Esophageal cancer | 363 | Yes | Submaximal | NR | Physical therapists | NA | NR | NR | NR |
| Tan, 2006^322^ | Esophageal cancer | 21 | No | Submaximal | NR | NR | Yes | NR | NR | NR |
| Tang, 2021^323^ | Esophageal cancer | 77 | Yes | Submaximal | NR | NR | NA | NR | NR | NR |
| Thomson, 2018^99^ | Esophageal cancer | 38 | No | Maximal | NR | NR | Yes | NR | NR | NR |
| van Vulpen, 2021^100^ | Esophageal cancer | 120 | No | Maximal | Yes | Sports med physician | No | NR | NR | NR |
| Von Dobeln, 2016^551^ | Esophageal cancer | 97 | Yes | Maximal | NR | NR | Yes | NR | NR | NR |
| West, 2021 ^101^ | Esophago-gastric | 136 | No | Maximal | NR | NR | No | NR | NR | NR |
| Cho, 2014^552^ | Gastro | 20 | No | Maximal | NR | NR | NR | No | 0 | NA |
| Cho, 2018^103^ | Gastro | 48 | No | Maximal | NR | NR | NR | No | NR | NR |
| Hyltander, 2005^102^ | Gastro | 31 | No | Maximal | NR | NR | NR | NR | NR | NR |
| Nowak, 2023^553^ | Glioblastoma | 30 | No | Submaximal | NR | NR | NA | NR | NR | NR |
| Sinclair, 2016^104^ | Gastro | 89 | Yes | Maximal | NR | NR | Yes | NR | NR | NR |
| Peel, 2015^554^ | Gynecologic | 37 | No | Submaximal | NR | NR | NR | NR | NR | NR |
| Eden, 2018^324^ | Head-neck / CNS | 72 | No | Submaximal | NR | NR | NR | NR | NR | NR |
| Samuel, 2013^325^ | Head-neck / CNS | 66 | No | Submaximal | Pretest familiarization | NR | NR | No | NR | NR |
| Samuel, 2019^326^ | Head-neck / CNS | 81 | Yes | Submaximal | NR | Physiotherapist | NR | NR | NR | NR |
| Su, 2017^327^ | Head-neck / CNS | 148 | No | Submaximal | NR | NR | NR | NR | NR | NR |
| Yen, 2019^328^ | Head-neck / CNS | 35 | No | Submaximal | NR | NR | NR | NR | NR | NR |
| Lindman, 2021^105^ | Hematological cancers | 30 | No | Maximal | NR | NR | Yes | NR | NR | NR |
| van Wijk, 2021^106^ | Hepatobillary | 100 | No | Submaximal | NR | NR | No | NR | NR | NR |
| Hayashi 2017^329^ | Hepato-pancreato-biliary cancer and liver | 75 | No | Submaximal | NR | NR | NR | NR | NR | NR |
| Alibhai, 2015^555^ | Leukemia | 237 | No | Submaximal | NR | NR | NA | NR | NR | NR |
| Caru, 2019^107^ | Leukemia | 1046 | No | Both maximal and submaximal | NR | Exercise physiologist/sport scientists | Yes | NR | NR | NR |
| Fateen, 2019^556^ | Leukemia | 50 | No | Submaximal | NR | NR | NA | NR | NR | NR |
| Jarfelt, 2007^557^ | Leukemia | 35 | No | Maximal | NR | NR | Yes | NR | NR | NR |
| Jarvela, 2010^108^ | Leukemia | 42 | No | Maximal | NR | Experienced tester | Yes | NR | NR | NR |
| Keerthana, 2024^330^ | Leukemia | 30 | No | Submaximal | NR | NR | NA | NR | NR | NR |
| Labonte, 2020 | Leukemia | 168 | No | Both maximal and submaximal | Yes | Exercise physiologist/sport scientists and physician | Yes | NR | NR | NR |
| Lemay, 2020^110^ | Leukemia | 224 | No | Maximal | NR | NR | NA | NR | NR | NR |
| Long, 2020^111^ | Leukemia | 36 | No | Maximal | NR | NR | Yes | NR | NR | NR |
| Morris, 2011^332^ | Leukemia | 59 | No | Submaximal | NR | Physiotherapist | NA | NR | NR | NR |
| Myrdal, 2018^112^ | Leukemia | 116 | No | Maximal | NR | NR | Yes | NR | NR | NR |
| Myrdal, 2020^113^ | Leukemia | 90 | Yes | Maximal | NR | NR | Yes | NR | NR | NR |
| Ness, 2007 ^558^ | Leukemia | 75 | No | Submaximal | NR | NR | NA | NR | NR | NR |
| Ness, 2012^333^ | Leukemia | 415 | Yes | Submaximal | NR | NR | NA | NR | NR | NR |
| Phillips, 2020^114^ | Leukemia | 629 | No | Submaximal | NR | NR | Yes | NR | NR | NR |
| Suominen, 2022 ^559^ | Leukemia | 98 | No | Submaximal | NR | NR | NA | NR | NR | NR |
| Timilshina, 2014^560^ | Leukemia | 239 | No | Submaximal | NR | NR | NA | NR | NR | NR |
| Timilshina, 2019^334^ | Leukemia | 71 | Yes | Submaximal | NR | NR | NA | NR | NR | NR |
| Tonorezos, 2013^115^ | Leukemia | 685 | No | Both maximal and submaximal | NR | NR | Yes | NR | NR | NR |
| Wogksch, 2022^335^ | Leukemia | 1166 | No | Submaximal | NR | NR | NA | NR | NR | NR |
| Yeon, 2022 ^116^ | Leukemia | 72 | No | Maximal | NR | Specialist nurse | Yes | NR | NR | NR |
| Armenian, 2017^117^ | Leukemia and lymphoma | 20 | Yes | Maximal | NR | Physician and technician | Yes | No | 0 | NA |
| Bayram, 2024^118^ | Leukemia and lymphoma | 30 | No | Maximal | NR | NR | Yes | NR | NR | NR |
| Bewarder, 2019^336^ | Leukemia and lymphoma | 45 | No | Submaximal | NR | NR | NA | No | 0 | NA |
| de Almeida, 2019^561^ | Leukemia and lymphoma | 27 | No | Submaximal | No | NR | NA | NR | NR | NR |
| DeFeo, 2020^337^ | Leukemia and lymphoma | 1967 | No | Submaximal | NR | NR | NA | NR | NR | NR |
| Groβek, 2021^562^ | Leukemia and lymphoma | 51 | No | Maximal | NR | Exercise physiologist | NA | NR | NR | NR |
| Ishikawa, 2019^119^ | Leukemia and lymphoma | 88 | No | Maximal | NR | NR | NA | NR | NR | NR |
| Jones, 2015^338^ | Leukemia and lymphoma | 407 | No | Submaximal | NR | Physiotherapist | NA | NR | NR | NR |
| Kirsten, 2021^120^ | Leukemia and lymphoma | 178 | No | Maximal | NR | NR | Yes | NR | NR | NR |
| Kramer, 2013^563^ | Leukemia and lymphoma | 40 | No | Submaximal | NR | NR | NA | NR | NR | NR |
| Morais, 2023^339^ | Leukemia and lymphoma | 29 | No | Submaximal | NR | NR | NA | NR | NR | NR |
| Morishita, 2013^455^ | Leukemia and lymphoma | 113 | No | Submaximal | NR | NR | NA | NR | NR | NR |
| Morishita, 2015 ^341^ | Leukemia and lymphoma | 30 | No | Submaximal | NR | NR | NA | NR | NR | NR |
| Morishita, 2017^342^ | Leukemia and lymphoma | 30 | No | Submaximal | NR | NR | NA | NR | NR | NR |
| Morishita, 2018^564^ | Leukemia and lymphoma | 28 | No | Maximal | NR | NR | Yes | NR | NR | NR |
| Dunne, 2016^121^ | Liver | 37 | Yes | Maximal | NR | Clinical physiologists | Yes | NR | NR | NR |
| Kaibori, 2013^122^ | Liver | 61 | Yes | Maximal | NR | NR | No | NR | NR | NR |
| Kaibori, 2019^123^ | Liver | 106 | Yes | Maximal | NR | NR | NR | NR | NR | NR |
| Kawaguchi, 2020^343^ | Liver | 65 | No | Submaximal | NR | Physiotherapist | NR | NR | NR | NR |
| Ambrogi, 2012^344^ | Lung | 29 | No | Submaximal | NR | NR | NA | NR | NR | NR |
| An, 2023^345^ | Lung | 104 | No | Submaximal | NR | NR | NA | NR | NR | NR |
| Andersen, 2013^565^ | Lung | 51 | No | Maximal | NR | NR | NA | NR | NR | NR |
| Andreetti, 2014^346^ | Lung | 145 | Yes | Submaximal | NR | NR | NA | NR | NR | NR |
| Araujo, 2016^347^ | Lung | 48 | Yes | Submaximal | Yes | NR | NA | NR | NR | NR |
| Arbane, 2014^566^ | Lung | 131 | No | Maximal | Two tests performed | NR | NA | NR | NR | NR |
| Ayub, 2023^348^ | Lung | 75 | No | Submaximal | No | NR | Yes | NR | NR | NR |
| Bagg, 1984^567^ | Lung | 30 | No | Submaximal | NR | NR | NA | NR | NR | NR |
| Batchelor, 2015^124^ | Lung | 22 | No | Maximal | NR | NR | NA | NR | NR | NR |
| Beccaria, 2001^125^ | Lung | 62 | Yes | Maximal | NR | NR | Yes | NR | NR | NR |
| Bechard, 1987^126^ | Lung | 50 | No | Maximal | NR | NR | Yes | NR | NR | NR |
| Begum, 2016^127^ | Lung | 1684 | No | Maximal | NR | NR | NA | NR | NR | NR |
| Benattia, 2016^568^ | Lung | 106 | Yes | Submaximal | NR | NR | NA | NR | NR | NR |
| Bhatia, 2019^128^ | Lung | 151 | No | Both maximal and submaximal | NR | NR |  | No | 0 | NA |
| Bobbio, 2009^129^ | Lung | 73 | No | Maximal | NR | NR | NA | NR | NR | NR |
| Bolliger, 1995^130^ | Lung | 25 | No | Maximal | NR | NR | Yes | NR | NR | NR |
| Boujibar, 2018^131^ | Lung | 34 | Yes | Maximal | NR | NR | NA | NR | NR | NR |
| Boujibar, 2022^569^ | Lung | 118 | No | Submaximal | NR | Exercise physiologist | NA | NR | NR | NR |
| Bradley, 2013^349^ | Lung | 363 | Yes | Submaximal | NR | NR | NA | NR | NR | NR |
| Brocki, 2015^350^ | Lung | 78 | Yes | Submaximal | NR | NR | NA | NR | NR | NR |
| Brocki, 2016^351^ | Lung | 68 | Yes | Submaximal | Yes | NR | NA | NR | NR | NR |
| Bruera, 2003^352^ | Lung | 33 | No | Submaximal | NR | NR | NA | NR | NR | NR |
| Brunelli, 2002^570^ | Lung | 160 | No | Maximal | NR | Physician | NA | NR | NR | NR |
| Brunelli, 2003 ^571^ | Lung | 229 | No | Maximal | NR | NR | NA | NR | NR | NR |
| Brunelli, 2003 ^572^ | Lung | 227 | No | Maximal | NR | Physician | NA | NR | NR | NR |
| Brunelli, 2004^573^ | Lung | 109 | No | Maximal | NR | Physician | NA | NR | NR | NR |
| Brunelli, 2007 ^574^ | Lung | 200 | Yes | Maximal | NR | NR | NA | NR | NR | NR |
| Brunelli, 2007 ^575^ | Lung | 156 | Yes | Maximal | NR | NR | NA | NR | NR | NR |
| Brunelli, 2008^576^ | Lung | 640 | Yes | Maximal | NR | Physician | NA | No | 0 | NA |
| Brunelli, 2009^132^ | Lung | 263 | No | Maximal | Yes | NR | Yes | NR | NR | NR |
| Brunelli, 2012^577^ | Lung | 282 | Yes | Maximal | NR | Physician | NA | NR | NR | NR |
| Brunelli, 2014^133^ | Lung | 157 | No | Maximal | NR | NR | Yes | NR | NR | NR |
| Brutsche, 2000^134^ | Lung | 125 | No | Maximal | NR | NR | Yes | NR | NR | NR |
| Campione, 2010^135^ | Lung | 99 | No | Maximal | NR | NR | Yes | NR | NR | NR |
| Cavalheri, 2016^136^ | Lung | 20 | No | Both maximal and submaximal | Yes | NR | Yes | NR | NR | NR |
| Chang, 2014^353^ | Lung | 65 | No | Submaximal | NR | NR | NA | NR | NR | NR |
| Chao, 2022^16^ | Lung | 125 | No | Maximal | NR | NR | Yes | No | 0 | NA |
| Chouinard, 2022^137^ | Lung | 593 | Yes | Maximal | No | NR | Yes | NR | NR | NR |
| Coats, 2016 ^354^ | Lung | 87 | No | Submaximal | NR | NR | NA | NR | NR | NR |
| Colman, 1982^138^ | Lung | 49 | No | Maximal | NR | NR | Yes | NR | NR | NR |
| D'Andrilli, 2016^355^ | Lung | 88 | Yes | Submaximal | NR | NR | Yes | NR | NR | NR |
| Denehy, 2013^356^ | Lung | 100 | Yes | Submaximal | NR | Clinical staff | NA | No | 0 | NA |
| Deslauriers, 2011^357^ | Lung | 100 | No | Submaximal | NR | NR | NA | NR | NR | NR |
| Dhillon, 2017^358^ | Lung | 111 | No | Submaximal | NR | NR | NA | NR | NR | NR |
| Doğan, 2020^359^ | Lung | 60 | Yes | Submaximal | NR | NR | NA | NR | NR | NR |
| Dong, 2017^578^ | Lung | 171 | No | Maximal | NR | Physician | NA | NR | NR | NR |
| Dun, 2023^139^ | Lung | 895 | No | Maximal | NR | NR | Yes | NR | NR | NR |
| Edbrooke, 2019 ^361^ | Lung | 80 | No | Submaximal | NR | NR | NA | NR | NR | NR |
| Edbrooke, 2019 ^360^ | Lung | 92 | Yes | Submaximal | NR | NR | NA | NR | NR | NR |
| Edvardsen, 2015 ^579^ | Lung | 61 | Yes | Maximal | NR | Exercise physiologist/sport scientists | NA | NR | NR | NR |
| Edvardsen, 2015 ^140^ | Lung | 70 | Yes | Maximal | Yes | NR | Yes | NR | NR | NR |
| Epstein, 1995^142^ | Lung | 74 | Yes | Maximal | NR | NR | Yes | NR | NR | NR |
| Erdogan, 2013 ^362^ | Lung | 24 | Yes | Maximal | NR | NR | NA | NR | NR | NR |
| Fang, 2013^143^ | Lung | 107 | Yes | Maximal | NR | NR | Yes | NR | NR | NR |
| Fennelly, 2017^580^ | Lung | 101 | No | Maximal | NR | NR | Yes | NR | NR | NR |
| Fernandez-Rodrıguez, 2018^144^ | Lung | 114 | Yes | Maximal | NR | NR | NA | NR | NR | NR |
| Fresard, 2016^145^ | Lung | 34 | Yes | Maximal | NR | NR | Yes | NR | NR | NR |
| Gao, 2015^363^ | Lung | 342 | No | Submaximal | NR | NR | NA | NR | NR | NR |
| Gao, 2024^146^ | Lung | 79 | No | Maximal | NR | NR | No | NR | NR | NR |
| Glattki, 2012^364^ | Lung | 47 | No | Submaximal | NR | NR | NA | NR | NR | NR |
| Goldsmith, 2021^365^ | Lung | 216 | No | Submaximal | NR | NR | NA | NR | NR | NR |
| Granger, 2014^366^ | Lung | 50 | Yes | Submaximal | Yes | NR | NA | NR | NR | NR |
| Granger, 2015 ^367^ | Lung | 56 | No | Submaximal | Performed two 6MWT | NR | NA | NR | NR | NR |
| Granger, 2015 ^147^ | Lung | 20 | Yes | Both maximal and submaximal | Yes | NR | Yes | NR | NR | NR |
| Granger, 2015 ^581^ | Lung | 69 | No | Submaximal | NR | NR | NA | NR | NR | NR |
| Granger, 2016^369^ | Lung | 69 | No | Submaximal | Yes | NR | NA | NR | NR | NR |
| Gravier, 2020^148^ | Lung | 203 | Yes | Maximal | Yes | NR | Yes | NR | NR | NR |
| Gravier, 2022^149^ | Lung | 36 | Yes | Maximal | NR | NR | Yes | NR | NR | NR |
| Ha, 2015^371^ | Lung | 96 | Yes | Submaximal | NR | Physician | NA | NR | NR | NR |
| Ha, 2020^372^ | Lung | 35 | Yes | Submaximal | NR | NR | NA | NR | NR | NR |
| Hamada, 2019^373^ | Lung | 224 | No | Submaximal | No | Physiotherapist | NA | NR | NR | NR |
| Hattori, 2018^374^ | Lung | 321 | Yes | Submaximal | NR | Physiotherapist | NA | NR | NR | NR |
| Helminen, 2021^582^ | Lung | 283 | No | Submaximal | NR | NR | NA | NR | NR | NR |
| Henke, 2014^375^ | Lung | 46 | No | Submaximal | NR | NR | NA | NR | NR | NR |
| Huang, 2017^376^ | Lung | 90 | Yes | Submaximal | NR | NR | NA | NR | NR | NR |
| Hwang, 2012^150^ | Lung | 24 | Yes | Maximal | NR | NR | Yes | NR | NR | NR |
| Illini, 2022^377^ | Lung | 52 | No | Submaximal | NR | NR | NA | No | 0 | NA |
| Irie, 2016^378^ | Lung | 188 | Yes | Submaximal | NR | Physiotherapist | NA | NR | NR | NR |
| Ito, 2015^583^ | Lung | 65 | Yes | Both maximal and submaximal | NR | NR | NA | NR | NR | NR |
| Janssen, 2017^151^ | Lung | 50 | Yes | Maximal | NR | NR | NA | NR | NR | NR |
| Jastrzebski, 2015^379^ | Lung | 20 | No | Submaximal | Yes | NR | NA | NR | NR | NR |
| Ji, 2019 ^380^ | Lung | 64 | No | Submaximal | NR | NR | NA | NR | NR | NR |
| Jones, 2007^152^ | Lung | 20 | No | Both maximal and submaximal | 6MWT: two tests performed | Exercise physiologist/sport scientists | Yes | No | 0 | NA |
| Jones, 2010^153^ | Lung | 398 | No | Maximal | NR | NR | Yes | NR | NR | NR |
| Jones, 2012 ^381^ | Lung | 118 | No | Submaximal | NR | Trained clinical research assistant | NA | NR | NR | NR |
| Jonsson, 2019^382^ | Lung | 94 | No | Submaximal | NR | Physiotherapist | NA | NR | NR | NR |
| Kadiri, 2019^584^ | Lung | 31 | Yes | Maximal | NR | NR | NA | NR | NR | NR |
| Kasikcioglu, 2009^154^ | Lung | 49 | Yes | Maximal | NR | NR | Yes | Yes | 7 | Unifocal ventricular arrhythmias=3, temporarily atrial fibrillation=2, drop in systolic blood pressure=2 |
| Kasymjanova, 2009^383^ | Lung | 64 | No | Submaximal | Yes | NR | NA | NR | NR | NR |
| Katakami, 2018 ^384^ | Lung | 174 | No | Submaximal | NR | NR | NA | NR | NR | NR |
| Keenan, 2004^585^ | Lung | 201 | No | NR | NR | NR | NA | NR | NR | NR |
| Kong, 2020^385^ | Lung | 614 | Yes | Submaximal | Yes | NR | NA | NR | NR | NR |
| Kristenson, 2024^155^ | Lung | 158 | Yes | Maximal | NR | NR | Yes | NR | NR | NR |
| Kushibe, 2008 ^586^ | Lung | 106 | Yes | Maximal | NR | NR | Yes | NR | NR | NR |
| Kushibe, 2008 ^157^ | Lung | 100 | Yes | Maximal | NR | NR | Yes | NR | NR | NR |
| Lai, 2017^386^ | Lung | 101 | No | Submaximal | NR | NR | Yes | NR | NR | NR |
| Lai, 2019^387^ | Lung | 68 | Yes | Submaximal | NR | NR | NA | NR | NR | NR |
| Larsen, 1997 ^158^ | Lung | 57 | No | Maximal | NR | NR | Yes | NR | NR | NR |
| Larsen, 1997 | Lung | 97 | No | Maximal | NR | NR | Yes | NR | NR | NR |
| Li, 2019^388^ | Lung | 80 | No | Submaximal | NR | NR | NA | NR | NR | NR |
| Li, 2024^389^ | Lung | 169 | No | Submaximal | NR | NR | NA | NR | NR | NR |
| Licker, 2011^160^ | Lung | 210 | Yes | Maximal | NR | NR | Yes | NR | NR | NR |
| Lindenmann, 2020^161^ | Lung | 342 | Yes | Maximal | NR | NR | Yes | NR | NR | NR |
| Liu, 2020 ^587^ | Lung | 73 | Yes | Submaximal | NR | Physician | NA | NR | NR | NR |
| Loewen, 2007^162^ | Lung | 403 | No | Maximal | NR | NR | Yes | NR | NR | NR |
| Marhic, 2019^163^ | Lung | 20 | Yes | Maximal | NR | NR | NA | NR | NR | NR |
| Marjanski, 2015^391^ | Lung | 253 | No | Submaximal | NR | Physiotherapist | Yes | NR | NR | NR |
| Markos, 1989^164^ | Lung | 55 | No | Both maximal and submaximal | Yes | NR | Yes | NR | NR | NR |
| Marulli, 2010^165^ | Lung | 36 | No | Maximal | NR | NR | Yes | NR | NR | NR |
| Maruyama, 2011^392^ | Lung | 100 | No | Submaximal | NR | NR | NA | NR | NR | NR |
| Matzi, 2007^166^ | Lung | 32 | Yes | Maximal | NR | NR | NA | NR | NR | NR |
| Menna, 2020^393^ | Lung | 44 | Yes | Submaximal | NR | NR | NA | NR | NR | NR |
| Miller, 2005^394^ | Lung | 31 | Yes | Submaximal | NR | Respiratory therapist | NA | NR | NR | NR |
| Minnella, 2021^167^ | Lung | 81 | Yes | Maximal | NR | Exercise physiologist | No | NR | NR | NR |
| Miyazaki, 2018^168^ | Lung | 209 | Yes | Maximal | NR | NR | Yes | NR | NR | NR |
| Mohan, 2008^395^ | Lung | 44 | No | Submaximal | NR | NR | NA | NR | NR | NR |
| Mohan, 2017^396^ | Lung | 32 | No | Submaximal | NR | NR | NA | NR | NR | NR |
| Morano, 2014^397^ | Lung | 24 | Yes | Submaximal | NR | NR | NA | NR | NR | NR |
| Mujovic, 2014^398^ | Lung | 83 | Yes | Submaximal | NR | NR | NA | NR | NR | NR |
| Mujovic, 2015^399^ | Lung | 103 | Yes | Submaximal | NR | NR | NA | NR | NR | NR |
| Nagamatsu, 1996^169^ | Lung | 64 | No | Submaximal | NR | NR | NA | NR | NR | NR |
| Nagamatsu, 2004^170^ | Lung | 211 | No | Maximal | NR | NR | NA | NR | NR | NR |
| Nagamatsu, 2011^171^ | Lung | 164 | Yes | Maximal | NR | NR | Yes | NR | NR | NR |
| Nakagawa, 2014 ^588^ | Lung | 121 | No | Submaximal | NR | NR | NA | NR | NR | NR |
| Nakagawa, 2018 ^400^ | Lung | 121 | No | Submaximal | NR | NR | NA | NR | NR | NR |
| Nezu, 1998^172^ | Lung | 82 | No | Maximal | NR | NR | Yes | NR | NR | NR |
| Nikolic, 2008^589^ | Lung | 101 | Yes | Maximal | NR | Physician | NA | NR | NR | NR |
| Ninan, 1997^590^ | Lung | 47 | No | Submaximal | NR | Respiratory technician | NA | NR | NR | NR |
| Nomori, 2001^401^ | Lung | 44 | No | Submaximal | NR | Physiotherapist | NA | NR | NR | NR |
| Nomori, 2003^402^ | Lung | 27 | No | Submaximal | NR | Physiotherapist | NA | NR | NR | NR |
| Novoa, 2011^173^ | Lung | 38 | Yes | Maximal | NR | NR | Yes | NR | NR | NR |
| Nugent, 1999^174^ | Lung | 77 | No | Maximal | NR | NR | Yes | NR | NR | NR |
| Op den Kamp, 2012^175^ | Lung | 26 | No | Maximal | NR | NR | NA | NR | NR | NR |
| Parsons, 2003^403^ | Lung | 70 | No | Submaximal | Yes | NR | NA | NR | NR | NR |
| Perrotta, 2019^176^ | Lung | 25 | Yes | Maximal | NR | NR | Yes | NR | NR | NR |
| Pierce, 1994^177^ | Lung | 54 | No | Both maximal and submaximal | NR | NR | Yes | NR | NR | NR |
| Quist, 2015^404^ | Lung | 114 | No | Both maximal and submaximal | NR | NR | Yes | No | 0 | NA |
| Quist, 2018^178^ | Lung | 211 | Yes | Both maximal and submaximal | NR | NR |  | NR | NR | NR |
| Quist, 2020^179^ | Lung | 218 | No | Both maximal and submaximal | NR | Physiotherapist |  | NR | NR | NR |
| Refai, 2014 ^591^ | Lung | 283 | No | Submaximal | NR | Physician | NA | No | 0 | NA |
| Rehman, 2023^405^ | Lung | 40 | No | Submaximal | NR | The principal investigator | NA | NR | NR | NR |
| Ribas, 2001^180^ | Lung | 40 | Yes | Maximal | NR | NR | Yes | NR | NR | NR |
| Rick, 2014^406^ | Lung | 227 | Yes | Submaximal | NR | NR | Yes | NR | NR | NR |
| Riesenberg, 2010^407^ | Lung | 45 | No | Both maximal and submaximal | NR | NR | No | NR | NR | NR |
| Rocco, 2013^181^ | Lung | 119 | Yes | Maximal | NR | NR | NA | NR | NR | NR |
| Rodrigues, 2016^182^ | Lung | 50 | Yes | Maximal | NR | NR | Yes | NR | NR | NR |
| Rosero, 2020^408^ | Lung | 26 | No | Submaximal | NR | NR | NA | No | 0 | NA |
| Rushwan, 2023^183^ | Lung | 552 | Yes | Maximal | NR | NR | Yes | NR | NR | NR |
| Rutkowska, 2019^409^ | Lung | 30 | Yes | Submaximal | NR | Physiotherapist | NA | NR | NR | NR |
| Saad, 2006^410^ | Lung | 36 | No | Submaximal | NR | NR | NA | NR | NR | NR |
| Saad, 2007^411^ | Lung | 36 | No | Submaximal | NR | NR | NA | NR | NR | NR |
| Saito, 2020^412^ | Lung | 311 | No | Submaximal | NR | NR | NA | NR | NR | NR |
| Salhi, 2015^184^ | Lung | 86 | Yes | Both maximal and submaximal | NR | NR | Yes | NR | NR | NR |
| Sebio Garcia, 2017  ^370^ | Lung | 22 | Yes | Both maximal and submaximal | NR | NR | Yes | NR | NR | NR |
| Shafiek, 2016^185^ | Lung | 51 | Yes | Maximal | NR | NR | Yes | NR | NR | NR |
| Shallwani, 2016^413^ | Lung | 47 | No | Submaximal | NR | NR | NA | NR | NR | NR |
| Stanzani, 2014^186^ | Lung | 239 | Yes | Maximal | NR | NR | NA | NR | NR | NR |
| Stefanelli, 2013^187^ | Lung | 40 | Yes | Both maximal and submaximal | NR | NR | Yes | NR | NR | NR |
| Sterzi, 2013^414^ | Lung | 110 | No | Submaximal | NR | NR | NA | NR | NR | NR |
| Stigt, 2013^415^ | Lung | 49 | No | Submaximal | NR | NR | NA | NR | NR | NR |
| Surg, 1984 | Lung | 82 | No | Maximal | NR | NR | No | NR | NR | NR |
| Suzuki, 2010^416^ | Lung | 20 | Yes | Submaximal | NR | NR | NA | NR | NR | NR |
| Takemura, 2021^417^ | Lung | 180 | No | Submaximal | NR | NR | NA | NR | NR | NR |
| Tang, 2009 ^428^ | Lung | 45 | No | Submaximal | NR | NR | Yes | NR | NR | NR |
| Tao, 2023^418^ | Lung | 88 | Yes | Submaximal | NR | Rehabilitation therapists | NA | NR | NR | NR |
| Timmerman, 2018^419^ | Lung | 23 | No | Submaximal | NR | NR | NA | NR | NR | NR |
| Titz, 2018^420^ | Lung | 227 | No | Submaximal | NR | Exercise physiologist/sport scientists | NA | Yes | 4 | dyspnea n=2  pain n=2 |
| Torchio, 2010^188^ | Lung | 145 | Yes | Maximal | NR | NR | Yes | NR | NR | NR |
| Torchio, 2017^189^ | Lung | 263 | No | Maximal | NR | NR | Yes | NR | NR | NR |
| Torre-Bouscoulet, 2018 ^422^ | Lung | 23 | No | Submaximal | NR | NR | NA | NR | NR | NR |
| Torre-Bouscoulet, 2018 ^421^ | Lung | 52 | No | Submaximal | NR | NR | Yes | NR | NR | NR |
| Ueda, 2019^423^ | Lung | 116 | No | Submaximal | NR | NR | NA | NR | NR | NR |
| Ugalde, 2008^424^ | Lung | 88 | No | Submaximal | NR | NR | Yes | NR | NR | NR |
| Umezu, 2012^190^ | Lung | 45 | Yes | Maximal | NR | NR | Yes | NR | NR | NR |
| Vagvolgyi, 2018^425^ | Lung | 238 | Yes | Both maximal and submaximal | NR | NR | Yes | NR | NR | NR |
| van der Weijst, 2023^426^ | Lung | 51 | Yes | Submaximal | NR | NR | NA | No | 0 | NA |
| Varela, 2001^592^ | Lung | 81 | Yes | Maximal | NR | NR | NA | NR | NR | NR |
| Vargas Fajardo, 2014^191^ | Lung | 26 | Yes | Maximal | NR | NR | NA | NR | NR | NR |
| Villani, 2004^192^ | Lung | 150 | Yes | Maximal | NR | NR | Yes | NR | NR | NR |
| Walsh, 1994^193^ | Lung | 66 | No | Maximal | NR | NR | Yes | NR | NR | NR |
| Wang, 1999^194^ | Lung | 40 | No | Maximal | NR | Physician | Yes | NR | NR | NR |
| Wang, 2000^195^ | Lung | 57 | Yes | Maximal | NR | NR | Yes | NR | NR | NR |
| Wang, 2006^196^ | Lung | 28 | No | Maximal | NR | NR | Yes | NR | NR | NR |
| Wang, 2011 ^197^ | Lung | 57 | No | Maximal | NR | NR | NA | NR | NR | NR |
| Wang, 2013^427^ | Lung | 105 | No | Submaximal | NR | NR | NA | NR | NR | NR |
| Weinstein, 2007^593^ | Lung | 191 | Yes | Maximal | NR | Physician | Yes | NR | NR | NR |
| Win, 2004^594^ | Lung | 111 | No | Maximal | Yes | NR | NA | NR | NR | NR |
| Win, 2005 ^595^ | Lung | 99 | No | Maximal | NR | NR | Yes | NR | NR | NR |
| Win, 2006^199^ | Lung | 125 | No | Maximal | Yes | NR | Yes | Yes | 2 | Significant ST changes without symptoms. Coronary angio confirmed signifcant coronary artery disease |
| Win, 2007^596^ | Lung | 110 | No | Maximal | NR | NR | NA | NR | NR | NR |
| Win, 2008^200^ | Lung | 110 | Yes | Maximal | NR | NR | NA | NR | NR | NR |
| Yang, 2018^429^ | Lung | 162 | Yes | Submaximal | NR | An assessor | NA | No | 0 | NA |
| Zarogoulidis, 2012^430^ | Lung | 241 | No | Submaximal | NR | NR | NA | NR | NR | NR |
| Zhang, 2020^431^ | Lung | 240 | No | Submaximal | NR | Respiratory therapist | NA | NR | NR | NR |
| Puente-Maestú, 2011^201^ | Lung and sarcoma | 126 | No | Maximal | NR | NR | NR | NR | NR | NR |
| Adams, 2015^597^ | Lymphoma | 21 | No | NR | NR | NR | NA | NR | NR | NR |
| Bargi, 2020^598^ | Lymphoma | 47 | No | Maximal | NR | NR | NA | NR | NR | NR |
| Courneya, 2009^202^ | Lymphoma | 122 | Yes | Maximal | NR | Exercise physiologist | Yes | NR | NR | NR |
| Cox, 2021^599^ | Lymphoma | 30 | No | Maximal | NR | NR | NA | NR | NR | NR |
| Elbl, 2006 ^203^ | Lymphoma | 47 | Yes | Maximal | NR | NR | NA | NR | NR | NR |
| Elbl, 2006 ^204^ | Lymphoma | 106 | Yes | Maximal | NR | NR | Yes | NR | NR | NR |
| Groarke, 2015^600^ | Lymphoma | 789 | Yes | Maximal | NR | NR | Yes | No | 0 | NA |
| Gustavsson, 1990^601^ | Lymphoma | 26 | No | Maximal | NR | NR | NA | NR | NR | NR |
| Jain, 1996^205^ | Lymphoma | 191 | No | Maximal | NR | NR | Yes | NR | NR | NR |
| Lund, 1996 ^602^ | Lymphoma | 116 | No | Maximal | NR | NR | Yes | NR | NR | NR |
| Murbraech, 2016^206^ | Lymphoma | 274 | No | Maximal | NR | NR | Yes | NR | NR | NR |
| Pohjola-Sintonen, 1987^603^ | Lymphoma | 28 | No | Maximal | NR | NR | Yes | Yes | 13 | local chest pain n=1, tachycardia during exercise n=2, myocardial ischemia n=2, unable to raise BP during exercise test n=3 |
| Rizwan, 2021^207^ | Lymphoma | 64 | Yes | Maximal | Yes | Exercise physiologist | Yes | No | 0 | NA |
| Vermaete, 2014^208^ | Lymphoma | 29 | No | Both maximal and submaximal | 6MWT: Two tests performed | NR | Yes | NR | NR | NR |
| Persoon, 2017^604^ | Lymphoma and melanoma | 109 | Yes | Maximal | NR | Physician | Yes | Yes | 8 | ECG abnormalities n=5, abnormalities in oxygen uptake or the oxygen equivalent n=2, lack of systolic BP response n=1 |
| Persoon, 2017^209^ | Lymphoma and multiple myeloma | 109 | Yes | Maximal | NR | NR | NR | NR | NR | NR |
| Adamsen, 2006^605^ | Mixed | 82 | No | Maximal | NR | NR | No | NR | NR | NR |
| Adamsen, 2009^606^ | Mixed | 269 | No | Maximal | NR | NR | No | NR | NR | NR |
| Al-Mozaini, 2022 ^449^ | Mixed | 37 | No | Submaximal | NR | NR | NA | NR | NR | NR |
| Armand, 2023^210^ | Mixed | 20 | No | Submaximal | NR | NR | No | NR | NR | NR |
| Armstrong, 2013^432^ | Mixed | 498 | Yes | Submaximal | NR | NR | NA | NR | NR | NR |
| Atkinson, 2021^211^ | Mixed | 43 | No | Maximal | NR | NR | Yes | NR | NR | NR |
| Baumann, 2011^607^ | Mixed | 33 | No | Submaximal | NR | NR | NA | No | 0 | NA |
| Bertheussen, 2013^212^ | Mixed | 163 | No | Maximal | NR | NR | NA | NR | NR | NR |
| Bird, 2010^608^ | Mixed | 58 | No | Maximal | NR | NR | NA | NR | NR | NR |
| Bjorke, 2020^213^ | Mixed | 615 | No | Maximal | NR | The test leaders | Yes | NR | NR | NR |
| Bjørke, 2020 ^214^ | Mixed | 535 | Yes | Maximal | Yes | Trained professionals | Yes | NR | NR | NR |
| Bolli, 2020^433^ | Mixed | 31 | Yes | Submaximal | No | NR | NA | NR | NR | NR |
| Broderick, 2013^609^ | Mixed | 43 | No | Submaximal | NR | NR | NA | No | 0 | NA |
| Brown, 2018^434^ | Mixed | 60 | No | Submaximal | NR | NR | NA | No | 0 | NA |
| Cantwell, 2024^610^ | Mixed | 191 | Yes | Maximal | NR | NR | NA | NR | NR | NR |
| Carannante, 2023^215^ | Mixed | 77 | Yes | Maximal | NR | NR | Yes | NR | NR | NR |
| Cella, 2008 ^611^ | Mixed | 401 | No | NR | NR | NR | NA | NR | NR | NR |
| Chemaitilly, 2015^435^ | Mixed | 748 | No | Submaximal | NR | NR | NA | NR | NR | NR |
| Coletta, 2021 ^612^ | Mixed | 849 | No | Maximal | NR | NR | NA | NR | NR | NR |
| Coletta, 2021 ^613^ | Mixed | 233 | No | Maximal | NR | NR | NA | NR | NR | NR |
| Courneya, 2008^216^ | Mixed | 55 | Yes | Maximal | NR | Physician supervised | Yes | NR | NR | NR |
| Daneryd, 1998^217^ | Mixed | 108 | No | Maximal | NR | NR | NA | NR | NR | NR |
| De Backer, 2007 ^218^ | Mixed | 37 | No | Both maximal and submaximal | NR | Exercise physiologist/sport scientists | Yes | No | 0 | NA |
| De Backer, 2007 ^614^ | Mixed | 57 | No | Maximal | NR | NR | Yes | NR | NR | NR |
| De Backer, 2008^220^ | Mixed | 71 | No | Maximal | NR | NR | Yes | NR | NR | NR |
| Demmelmaier, 2021^221^ | Mixed | 577 | No | Maximal | NR | NR | No | NR | NR | NR |
| Devenney, 2023^436^ | Mixed | 37 | No | Submaximal | NR | NR | NA | NR | NR | NR |
| Dimeo, 1997^615^ | Mixed | 70 | No | Maximal | NR | NR | Yes | NR | NR | NR |
| Dimeo, 2004^616^ | Mixed | 35 | No | Maximal | NR | NR | Yes | NR | NR | NR |
| Drake, 2004^617^ | Mixed | 45 | No | Both maximal and submaximal | NR | NR | Yes | NR | NR | NR |
| Fioritto, 2021^618^ | Mixed | 26 | Yes | Maximal | NR | NR | Yes | NR | NR | NR |
| Gagnon, 2013^437^ | Mixed | 131 | No | Submaximal | NR | NR | NA | NR | NR | NR |
| George, 2014^619^ | Mixed | 54 | No | Maximal | NR | NR | NA | NR | NR | NR |
| Glare, 2011^438^ | Mixed | 54 | No | Submaximal | NR | NR | NA | NR | NR | NR |
| Goodenough, 2022^222^ | Mixed | 185 | No | Maximal | NR | NR | Yes | NR | NR | NR |
| Green, 2016^439^ | Mixed | 606 | No | Submaximal | NR | NR | NA | NR | NR | NR |
| Gresham, 2018^620^ | Mixed | 248 | Yes | Submaximal | NR | NR | NA | NR | NR | NR |
| Griffith, 2009^223^ | Mixed | 126 | No | Maximal | NR | NR | Yes | NR | NR | NR |
| Groarke, 2020 ^621^ | Mixed | 1632 | Yes | Maximal | NR | NR | NA | NR | NR | NR |
| Hadzibegovic, 2023^440^ | Mixed | 333 | Yes | Submaximal | NR | NR | NA | NR | NR | NR |
| Hayakawa, 2019^441^ | Mixed | 93 | No | Submaximal | NR | NR | NA | NR | NR | NR |
| Henriksson, 2023^224^ | Mixed | 351 | Yes | Maximal | NR | Trained professionals | No | NR | NR | NR |
| Huang, 2016^225^ | Mixed | 26 | Yes | Maximal | NR | Physician | Yes | NR | NR | NR |
| Hui, 2014^442^ | Mixed | 20 | Yes | Submaximal | NR | NR | Yes | NR | NR | NR |
| Hutchison, 2019^443^ | Mixed | 103 | No | Submaximal | NR | Physiotherapist | Yes | NR | NR | NR |
| Irwin, 2017^444^ | Mixed | 186 | No | Submaximal | 6MWT: two tests performed | NR | NA | No | 0 | NA |
| Jones, 2014^226^ | Mixed | 90 | Yes | Both maximal and submaximal | NR | NR | Yes | NR | NR | NR |
| Kampshoff, 2015^227^ | Mixed | 277 | No | Maximal | NR | NR | NA | NR | NR | NR |
| Kampshoff, 2018^228^ | Mixed | 277 | No | Maximal | NR | NR | NA | NR | NR | NR |
| Kenjale, 2014^229^ | Mixed | 413 | Yes | Maximal | NR | Exercise physiologist/sport scientists | Yes | Yes | 31 | ST depression n=27, bundle branch block n=4 |
| Kerrigan, 2023^230^ | Mixed | 22 | Yes | Maximal | NR | NR | Yes | NR | NR | NR |
| Kimberg, 2015^445^ | Mixed | 1304 | No | Submaximal | NR | NR | NA | NR | NR | NR |
| Kirkham, 2016^446^ | Mixed | 132 | No | Submaximal | NR | Exercise physiologist/sport scientists | NA | NR | NR | NR |
| Klika, 2011^231^ | Mixed | 268 | No | Both maximal and submaximal | NR | NR | Yes | NR | NR | NR |
| Knobf, 2017^622^ | Mixed | 154 | No | Maximal | NR | NR | Yes | NR | NR | NR |
| Knutsen, 2006^623^ | Mixed | 100 | No | Maximal | Pre-test familization | Nurse and physiotherapist | NA | No | 0 | NA |
| Leach, 2018^447^ | Mixed | 547 | No | Submaximal | NR | Physiotherapist | Yes | NR | NR | NR |
| Lestuzzi, 2022^624^ | Mixed | 192 | Yes | Maximal | NR | NR | NA | Yes | 27 | ECG and symptoms of ischemia n=8,  silent ischemia n=8,  arrhythmia n=16 |
| Lezin, 2019 ^448^ | Mixed | 208 | Yes | Submaximal | NR | NR | NA | NR | NR | NR |
| Li, 2018 ^232^ | Mixed | 43 | No | Maximal | NR | Physician | Yes | NR | NR | NR |
| Lundholm, 2004 ^233^ | Mixed | 304 | No | Maximal | NR | NR | Yes | NR | NR | NR |
| Madeddu, 2012 ^450^ | Mixed | 60 | No | Submaximal | NR | NR | NA | NR | NR | NR |
| Mäkinen, 1990 ^625^ | Mixed | 41 | Yes | Maximal | NR | NR | Yes | NR | NR | NR |
| Mangia, 2017 ^451^ | Mixed | 121 | No | Submaximal | NR | NR | NA | NR | NR | NR |
| Marker, 2024 ^452^ | Mixed | 420 | No | Submaximal | NR | NR | NA | NR | NR | NR |
| May, 2008 ^234^ | Mixed | 147 | No | Maximal | NR | Physician present | NA | NR | NR | NR |
| May, 2010 ^235^ | Mixed | 147 | No | Both maximal and submaximal | Pre-test familization | An assessor | Yes | NR | NR | NR |
| McCrary, 2019 ^453^ | Mixed | 100 | No | Submaximal | NR | NR | NA | No | 0 | NA |
| Midtgaard, 2005 ^626^ | Mixed | 91 | No | Maximal | NR | NR | NA | NR | NR | NR |
| Midtgaard, 2013 ^236^ | Mixed | 214 | No | Maximal | NR | NR | NA | NR | NR | NR |
| Mikkelsen, 2022 | Mixed | 84 | No | Submaximal | NR | NR | NA | No | 0 | NA |
| Morishita, 2013 ^340^ | Mixed | 321 | No | Submaximal | NR | NR | NA | NR | NR | NR |
| Morishita, 2015 ^627^ | Mixed | 126 | No | Submaximal | NR | NR | NA | NR | NR | NR |
| Morris, 2009 ^456^ | Mixed | 30 | Yes | Submaximal | NR | Physiotherapist | Yes | NR | NR | NR |
| Mulrooney, 2016 ^457^ | Mixed | 1853 | Yes | Submaximal | NR | NR | NA | NR | NR | NR |
| Nadruz, 2018 ^237^ | Mixed | 75 | Yes | Maximal | NR | NR | Yes | NR | NR | NR |
| Nawoor-Quinn, 2022 ^238^ | Mixed | 1398 | Yes | Maximal | NR | Exercise physiologist | Yes | NR | NR | NR |
| Ness, 2020 ^239^ | Mixed | 1326 | No | Maximal | NR | NR | Yes | NR | NR | NR |
| Ngo-Huang, 2021 ^458^ | Mixed | 23 | No | Submaximal | NR | NR | NA | NR | NR | NR |
| Noh, 2023 ^459^ | Mixed | 94 | No | Submaximal | NR | NR | NA | NR | NR | NR |
| Oldervoll, 2011 ^628^ | Mixed | 231 | Yes | Maximal | NR | NR | NA | NR | NR | NR |
| O'Mahony, 2021 ^240^ | Mixed | 317 | No | Maximal | NR | Exercise physiologist | No | NR | NR | NR |
| Oviatt, 2011 ^460^ | Mixed | 37 | No | Submaximal | NR | NR | NA | NR | NR | NR |
| Pahl, 2020 ^241^ | Mixed | 44 | No | Maximal | NR | NR | NA | NR | NR | NR |
| Parmar, 2017 ^461^ | Mixed | 374 | No | Submaximal | NR | Physiotherapist | NA | NR | NR | NR |
| Pein, 2004 ^242^ | Mixed | 447 | No | Maximal | NR | NR | NA | NR | NR | NR |
| Pillinger, 2022 ^243^ | Mixed | 129 | No | Maximal | Yes | NR | Yes | No | 0 | NA |
| Potiaumpai, 2021 ^462^ | Mixed | 35 | No | Submaximal | NR | NR | NA | NR | NR | NR |
| Prinsen, 2013 ^244^ | Mixed | 37 | No | Maximal | NR | NR | NA | NR | NR | NR |
| Quinn, 2020 ^629^ | Mixed | 103 | No | Submaximal | NR | NR | NA | NR | NR | NR |
| Rabin, 2016 ^630^ | Mixed | 35 | No | Submaximal | NR | NR | NA | NR | NR | NR |
| Repka, 2014 ^631^ | Mixed | 319 | No | NR | NR | NR | NA | NR | NR | NR |
| Rossi, 2016 ^463^ | Mixed | 28 | No | Submaximal | NR | NR | NA | NR | NR | NR |
| Ruiz-Casado, 2014 ^632^ | Mixed | 319 | No | NR | NR | NR | NA | NR | NR | NR |
| Santa Mina, 2017 ^464^ | Mixed | 224 | No | Submaximal | NR | NR | Yes | NR | NR | NR |
| Santa Mina, 2020 ^245^ | Mixed | 44 | Yes | Maximal | Yes | Exercise physiologist | Yes | NR | NR | NR |
| Schmidt, 2013 ^246^ | Mixed | 50 | No | Both maximal and submaximal | NR | Physician | Yes | NR | NR | NR |
| Schmitt, 2016 ^247^ | Mixed | 26 | No | Maximal | NR | NR | NA | NR | NR | NR |
| Schneider, 2023 ^248^ | Mixed | 57 | Yes | Maximal | Yes | Exercise physiologist | Yes | NR | NR | NR |
| Schuler, 2017 ^465^ | Mixed | 70 | No | Submaximal | NR | NR | NA | NR | NR | NR |
| Schumacher, 2019 ^466^ | Mixed | 187 | No | Both maximal and submaximal | NR | Clinical Cancer Exercise Specialists | Yes | No | 0 | NA |
| Slater, 2015 ^467^ | Mixed | 185 | No | Submaximal | NR | NR | Yes | NR | NR | NR |
| Smith, 2014 ^468^ | Mixed | 1778 | No | Submaximal | NR | NR | NA | NR | NR | NR |
| Stuiver, 2017 ^249^ | Mixed | 283 | No | Maximal | NR | NR | Yes | NR | NR | NR |
| Sturm, 2014 ^469^ | Mixed | 40 | No | Submaximal | NR | NR | NA | NR | NR | NR |
| Swenson, 2014 ^470^ | Mixed | 115 | No | Submaximal | NR | Physiotherapist | Yes | NR | NR | NR |
| Thorsen, 2005 ^633^ | Mixed | 111 | No | Submaximal | NR | NR | NA | NR | NR | NR |
| Thorsen, 2006 ^634^ | Mixed | 90 | No | Submaximal | NR | NR | NA | NR | NR | NR |
| Tran, 2016 ^471^ | Mixed | 188 | No | Submaximal | NR | NR | NA | NR | NR | NR |
| Uster, 2018 ^472^ | Mixed | 58 | No | Submaximal | NR | Physiotherapist | NA | NR | NR | NR |
| van der Schoot, 2022 ^250^ | Mixed | 266 | No | Maximal | NR | Trained professionals | Yes | NR | NR | NR |
| van Haren, 2018 ^635^ | Mixed | 29 | No | Submaximal | NR | Physiotherapist | NA | No | 0 | NA |
| Van Weert, 2004 ^254^ | Mixed | 37 | No | Maximal | NR | NR | Yes | NR | NR | NR |
| Velensek, 2008 ^636^ | Mixed | 179 | No | Submaximal | NR | NR | NA | NR | NR | NR |
| Viamonte, 2023 ^251^ | Mixed | 75 | Yes | Maximal | NR | NR | No | NR | NR | NR |
| Waterland, 2022 ^252^ | Mixed | 50 | No | Maximal | NR | NR | No | NR | NR | NR |
| Weemaes, 2021 ^253^ | Mixed | 106 | No | Maximal | Yes | NR | No | NR | NR | NR |
| Wiestad, 2020 ^255^ | Mixed | 55 | No | Maximal | Yes | Trained professionals | Yes | NR | NR | NR |
| Coleman, 2008^473^ | Multiple Myeloma | 120 | No | Submaximal | NR | NR | NR | NR | NR | NR |
| Koutoukidis, 2020^256^ | Multiple Myeloma | 131 | No | Maximal | NR | NR | NR | NR | NR | NR |
| Larsen, 2020^474^ | Multiple myeloma | 100 | Yes | Submaximal | NR | NR | NA | NR | NR | NR |
| Van Dongen, 2019^257^ | Multiple Myeloma | 109 | No | Maximal | NR | NR | NR | NR | NR | NR |
| Lee, 2022 ^258^ | Ovarian cancer | 27 | No | Maximal | NR | NR | Yes | NR | NR | NR |
| Pinelli, 2021 ^259^ | Ovarian Cancer | 114 | No | NR | NR | NR | No | NR | NR | NR |
| Smits, 2023 ^260^ | Ovarian cancer | 142 | No | Maximal | NR | NR | No | NR | NR | NR |
| An, 2019^475^ | Pancreatic cancer | 50 | No | Submaximal | NR | NR | NR | NR | NR | NR |
| Ausania, 2012^261^ | Pancreatic cancer | 50 | Yes | Submaximal | NR | NR | NR | NR | NR | NR |
| Chandrabalan, 2013^262^ | Pancreatic cancer | 100 | No | Maximal | NR | NR | NR | NR | NR | NR |
| Junejo, 2014^263^ | Pancreatic cancer | 64 | No | Maximal | NR | Physician and clinical scientist | Yes | NR | NR | NR |
| Stene, 2019^476^ | Pancreatic cancer | 46 | Yes | Submaximal | NR | NR | NR | NR | NR | NR |
| Wiedenmann, 2008^477^ | Pancreatic cancer | 89 | No | Submaximal | NR | NR | NR | NR | NR | NR |
| Alberga, 2012 ^264^ | Prostate | 25 | No | Maximal | NR | NR | NA | NR | NR | NR |
| Alibhai, 2010 ^478^ | Prostate | 259 | No | Submaximal | NR | NR | NA | NR | NR | NR |
| Alibhai, 2015 ^479^ | Prostate | 259 | No | Submaximal | NR | NR | NA | NR | NR | NR |
| Au, 2019 ^480^ | Prostate | 38 | No | Submaximal | NR | NR | NA | NR | NR | NR |
| Beydoun, 2014 ^637^ | Prostate | 379 | No | Submaximal | NR | Exercise physiologist/sport scientists | NA | NR | NR | NR |
| Bonsignore, 2018 ^265^ | Prostate | 27 | Yes | Maximal | NR | NR | Yes | NR | NR | NR |
| Capela, 2023 ^638^ | Prostate | 31 | Yes | Maximal | NR | NR | NA | NR | NR | NR |
| Cormie, 2015 ^639^ | Prostate | 64 | No | NR | NR | Exercise physiologist/sport scientists | NA | NR | NR | NR |
| Djurhuus, 2023 ^266^ | Prostate | 30 | No | Maximal | NR | NR | No | NR | NR | NR |
| Eriksen, 2017 ^267^ | Prostate | 26 | No | Maximal | NR | NR | NA | NR | NR | NR |
| Galvao, 2010 ^640^ | Prostate | 28 | No | Submaximal | NR | NR | NA | NR | NR | NR |
| Galvão, 2014 ^641^ | Prostate | 100 | No | Submaximal | NR | NR | NA | No | 0 | NA |
| Gong, 2020 ^642^ | Prostate | 616 | Yes | Maximal | NR | NR | NA | NR | NR | NR |
| Harrison, 2022 ^268^ | Prostate | 26 | No | Maximal | NR | Trained professionals | Yes | No | 0 | NA |
| Henriksson, 1987 ^643^ | Prostate | 100 | Yes | Maximal | NR | NR | Yes | Yes | 1 | Angina pectoris, central chest pain, dizziness |
| Henriksson, 1989 ^644^ | Prostate | 100 | No | Maximal | NR | NR | Yes | NR | NR | NR |
| Hojan, 2017 ^269^ | Prostate | 72 | No | Both maximal and submaximal | NR | NR | No | NR | NR | NR |
| Houben, 2023 | Prostate | 96 | Yes | Maximal | NR | NR | Yes | NR | NR | NR |
| Jones, 2014 ^32^ | Prostate | 50 | No | Maximal | NR | NR | NA | No | 0 | NA |
| Kang, 2021 ^272^ | Prostate | 52 | Yes | Maximal | NR | NR | No | NR | NR | NR |
| Lam, 2020 ^645^ | Prostate | 25 | No | Submaximal | NR | NR | NA | NR | NR | NR |
| Lauwick, 2009 ^646^ | Prostate | 40 | No | Submaximal | NR | NR | NA | NR | NR | NR |
| Mareschal, 2017 ^481^ | Prostate | 24 | No | Submaximal | NR | Trained professionals | NA | NR | NR | NR |
| Ndjavera, 2020 ^273^ | Prostate | 50 | No | Maximal | NR | NR | NA | NR | NR | NR |
| Nilsen, 2015 ^647^ | Prostate | 58 | No | Maximal | NR | NR | NA | NR | NR | NR |
| O´Neill, 2015 ^648^ | Prostate | 94 | No | Submaximal | NR | NR | NA | NR | NR | NR |
| Park, 2021 ^649^ | Prostate | 172 | Yes | Submaximal | NR | Instructor | NA | NR | NR | NR |
| Santa Mina, 2018 ^483^ | Prostate | 86 | No | Submaximal | NR | NR | NA | Yes | 1 | leg pain during 6MWT n=1 |
| Scott, 2015 ^274^ | Prostate | 40 | Yes | Maximal | Yes | Exercise physiologist/sport scientists | Yes | Yes | 3 | Ischemic ECG changes |
| Segal, 2009 ^275^ | Prostate | 121 | No | Maximal | NR | NR | NA | Yes | 1 | Syncope before treadmill test |
| Uth, 2014 ^276^ | Prostate | 57 | No | Both maximal and submaximal | Yes | NR | NA | NR | NR | NR |
| Uth, 2018 ^277^ | Prostate | 22 | No | Maximal | NR | NR | NA | NR | NR | NR |
| Van Blarigan, 2023^278^ | Prostate | 51 | No | Maximal | NR | NR | No | NR | NR | NR |
| Villumsen, 2019 ^484^ | Prostate | 46 | Yes | Submaximal | NR | NR | NA | NR | NR | NR |
| Windsor, 2004 ^650^ | Prostate | 65 | No | Maximal | NR | NR | NA | NR | NR | NR |
| Bishop, 2020^485^ | Sarcoma | 479 | No | Submaximal | NR | Exercise physiologist/sport scientists | NR | NR | NR | NR |
| Adams, 2017^279^ | Testicular | 63 | No | Maximal | NR | NR | NR | NR | NR | NR |
| Bloomquista, 2023 | Testicular | 40 | Yes | Maximal | NR | NR | NA | NR | NR | NR |
| Hyltander, 1991^280^ | Testicular | 33 | No | Maximal | NR | NR | NR | NR | NR | NR |

**Abbreviation**: 6MWT, six-minute walk test, AE, adverse event, HR, heart rate, NA, not applicable; NR, not reported

## REFERENCES

1. Banerjee S, Manley K, Shaw B, et al. Vigorous intensity aerobic interval exercise in bladder cancer patients prior to radical cystectomy: a feasibility randomised controlled trial. *Supportive Care in Cancer* 2018; **26**(5): 1515-23.

2. Lamb BW, Tan WS, Eneje P, et al. Benefits of robotic cystectomy with intracorporeal diversion for patients with low cardiorespiratory fitness: A prospective cohort study. *Urologic oncology* 2016; **34**(9): 417.e17-23.

3. Longdon E, Mistry H, Pratt O, et al. Variables associated with survival in patients with invasive bladder cancer with and without surgery. *Anaesthesia* 2020; **75**(7): 887-95.

4. Tolchard S, Angell J, Pyke M, et al. Cardiopulmonary reserve as determined by cardiopulmonary exercise testing correlates with length of stay and predicts complications after radical cystectomy. *BJU international* 2015; **115**(4): 554-61.

5. An KY, Kang DW, Morielli AR, et al. Patterns and predictors of exercise behavior during 24 months of follow-up after a supervised exercise program during breast cancer chemotherapy. 2020; **17**(1).

6. Bell RA, Baldi JC, Jones LM. Additional cardiovascular fitness when progressing from moderate- to high-intensity exercise training in previously trained breast cancer survivors. *Supportive care in cancer* 2021.

7. Bender CM, Sereika SM, Gentry AL, et al. Physical activity, cardiorespiratory fitness, and cognitive function in postmenopausal women with breast cancer. *Support Care Cancer* 2021; **29**(7): 3743-52.

8. Bender CM, Sereika SM, Gentry AL, et al. Effects of aerobic exercise on neurocognitive function in postmenopausal women receiving endocrine therapy for breast cancer: The Exercise Program in Cancer and Cognition randomized controlled trial. *Psycho-Oncology* 2024; **33**(2).

9. Berling-Ernst A, Yahiaoui-Doktor M, Kiechle M, et al. Predictors of cardiopulmonary fitness in cancer-affected and -unaffected women with a pathogenic germline variant in the genes BRCA1/2 (LIBRE-1). *Sci Rep* 2022; **12**(1): 2907.

10. Bigaran A, Howden EJ, Foulkes S, et al. Prescribing Exercise in Early-Stage Breast Cancer During Chemotherapy: A Simple Periodized Approach to Align With the Cyclic Phases of Chemotherapy. *Journal of Strength & Conditioning Research* 2022; **36**(10): 2934-41.

11. Bohn SK, Thune I, Flote VG, et al. Effects of a 1-Year Physical Activity Intervention on Markers of Hemostasis among Breast Cancer Survivors: A Randomized Controlled Trial. *TH Open* 2021; **5**(1): E14-E23.

12. Bonsignore A, Marzolini S, Oh P. Cardiac rehabilitation for women with breast cancer and treatment-related heart failure compared with coronary artery disease: A retrospective study. *Journal of rehabilitation medicine* 2017; **49**(3): 277-81.

13. Bonsignore A, Marwick TH, Adams SC, et al. Clinical, Echocardiographic, and Biomarker Associations With Impaired Cardiorespiratory Fitness Early After HER2-Targeted Breast Cancer Therapy. *JACC: CardioOncology* 2021; **3**(5): 678-91.

14. Burnett D, Kluding P, Porter C, Fabian C, Klemp J. Cardiorespiratory fitness in breast cancer survivors. *Springerplus* 2013; **2**(1): 68.

15. Cheema BS, Gaul CA. Full-body exercise training improves fitness and quality of life in survivors of breast cancer. 2006; **20**(1): 14.

16. Chao WH, Tuan SH, Tang EK, Tsai YJ, Chung JH, Chen GB, Lin KL. Effectiveness of Perioperative Cardiopulmonary Rehabilitation in Patients With Lung Cancer Undergoing Video-Assisted Thoracic Surgery. *Frontiers in Medicine* 2022; **9**((Chao W.-H.) Department of Medical Education and Research, Kaohsiung Veterans General Hospital, Kaohsiung City, Taiwan(Chao W.-H.; Tsai Y.-J.) Department of Medical Education and Research, Zuoying Branch of Kaohsiung Armed Forces General Hospital, Kaohsiu).

17. Cornette T, Vincent F, Mandigout S, et al. Effects of home-based exercise training on VO2 in breast cancer patients under adjuvant or neoadjuvant chemotherapy (SAPA): a randomized controlled trial. *European journal of physical and rehabilitation medicine* 2016; **52**(2): 223-32.

18. Courneya KS, Mackey JR, Bell GJ, Jones LW, Field CJ, Fairey AS. Randomized controlled trial of exercise training in postmenopausal breast cancer survivors: cardiopulmonary and quality of life outcomes. *Journal of clinical oncology* 2003; **21**(9): 1660‐8.

19. Courneya KS, Segal RJ, Mackey JR, et al. Effects of aerobic and resistance exercise in breast cancer patients receiving adjuvant chemotherapy: a multicenter randomized controlled trial. *J Clin Oncol* 2007; **25**(28): 4396-404.

20. Courneya KS, McKenzie DC, Mackey JR, et al. Moderators of the effects of exercise training in breast cancer patients receiving chemotherapy: a randomized controlled trial. *Cancer* 2008; **112**(8): 1845-53.

21. Courneya KS, Segal RJ, Gelmon K, et al. Predictors of adherence to different types and doses of supervised exercise during breast cancer chemotherapy. *Int J Behav Nutr Phys Act* 2014; **11**: 85.

22. Diaz-Balboa E, Gonzalez-Salvado V, Rodriguez-Romero B, et al. Thirty-second sit-to-stand test as an alternative for estimating peak oxygen uptake and 6-min walking distance in women with breast cancer: a cross-sectional study. 2022; **30**(10): 8251‐60.

23. Dolan LB, Gelmon K, Courneya KS, et al. Hemoglobin and aerobic fitness changes with supervised exercise training in breast cancer patients receiving chemotherapy. 2010; **19**(11): 2826.

24. Foulkes SJ, Howden EJ, Haykowsky MJ, et al. Exercise for the Prevention of Anthracycline-Induced Functional Disability and Cardiac Dysfunction: the BREXIT Study. 2023; **147**(7): 532‐45.

25. Friedenreich CM, Vallance JK, McNeely ML, et al. The Alberta moving beyond breast cancer (AMBER) cohort study: baseline description of the full cohort. *Cancer Causes Control* 2022; **33**(3): 441-53.

26. Giallauria F, Gentile M, Chiodini P, et al. Exercise training reduces high mobility group box-1 protein levels in women with breast cancer: findings from the DIANA-5 study. *Monaldi archives for chest disease = Archivio Monaldi per le malattie del torace* 2014; **82**(2): 61-7.

27. Hokken JW, van der Cruijsen-Raaijmakers M, Schep G, Vreugdenhil G. Impact of anthracycline dose on quality of life and rehabilitation in breast cancer treatment. *Neth J Med* 2009; **67**(6): 220-5.

28. Hughes DC, Darby N, Gonzalez K, Boggess T, Morris RM, Ramirez AG. Effect of a six-month yoga exercise intervention on fitness outcomes for breast cancer survivors. *Physiotherapy theory and practice* 2015; **31**(7): 451-60.

29. Isanejad A, Nazari S, Gharib B, Motlagh AG. Comparison of the effects of high-intensity interval and moderate-intensity continuous training on inflammatory markers, cardiorespiratory fitness, and quality of life in breast cancer patients. *Journal of Sport & Health Science* 2023; **12**(6): 674-89.

30. Jacquinot Q, Meneveau N, Falcoz A, et al. Cardiotoxicity is mitigated after a supervised exercise program in HER2-positive breast cancer undergoing adjuvant trastuzumab. *Frontiers in Cardiovascular Medicine* 2022; **9**((Jacquinot Q., qjacquinot@irfc-fc.fr) Regional Federative Cancer Institute of Franche-Comté, Besançon, France(Jacquinot Q., qjacquinot@irfc-fc.fr; Bouhaddi M.; Mougin F.) Research Unit EA3920, University of Franche-Comté, Besançon, France(Meneveau N.; Cur).

31. Jones LW, Courneya KS, Mackey JR, et al. Cardiopulmonary function and age-related decline across the breast cancer survivorship continuum. *J Clin Oncol* 2012; **30**(20): 2530-7.

32. Khouri MG, Hornsby WE, Risum N, et al. Utility of 3-dimensional echocardiography, global longitudinal strain, and exercise stress echocardiography to detect cardiac dysfunction in breast cancer patients treated with doxorubicin-containing adjuvant therapy. *Breast cancer research and treatment* 2014; **143**(3): 531-9.

33. Kiecolt-Glaser JK, Renna M, Peng J, et al. Breast cancer survivors' typhoid vaccine responses: chemotherapy, obesity, and fitness make a difference. 2022; **103**: 1‐9.

34. Kim CJ, Kang DH, Smith BA, Landers KA. Cardiopulmonary responses and adherence to exercise in women newly diagnosed with breast cancer undergoing adjuvant therapy. 2006; **29**(2): 156.

35. Kirkham A, Bland K, Zucker D, et al. "Chemotherapy-periodized" Exercise to Accommodate for Cyclical Variation in Fatigue. 2020; **52**(2): 278‐86.

36. Klassen O, Schmidt ME, Scharhag-Rosenberger F, et al. Cardiorespiratory fitness in breast cancer patients undergoing adjuvant therapy. *Acta oncologica (Stockholm, Sweden)* 2014; **53**(10): 1356-65.

37. Koevoets EW, Schagen SB, de Ruiter MB, et al. Effect of physical exercise on cognitive function after chemotherapy in patients with breast cancer: a randomized controlled trial (PAM study). 2022; **24**(1): 36.

38. Koevoets EW, Petr J, Monninkhof EM, et al. Effect of Physical Exercise on MRI-Assessed Brain Perfusion in Chemotherapy-Treated Breast Cancer Patients: A Randomized Controlled Trial. *Journal of Magnetic Resonance Imaging* 2023; ((Koevoets E.W.; Monninkhof E.M., e.monninkhof@umcutrecht.nl; Witlox L.; May A.M.) Julius Center for Health Sciences and Primary Care, University Medical Center Utrecht, Utrecht University, Utrecht, Netherlands(Koevoets E.W.; Stuiver M.M.; de Ruiter M.B.;).

39. Lahart IM, Carmichael AR, Nevill AM, Kitas GD, Metsios GS. The effects of a home-based physical activity intervention on cardiorespiratory fitness in breast cancer survivors; a randomised controlled trial. *J Sports Sci* 2018; **36**(10): 1077-86.

40. MacVicar MG, Winningham ML, Nickel JL. Effects of aerobic interval training on cancer patients' functional capacity. *Nursing research* 1989; **38**(6): 348-51.

41. Madison AA, Filatov M, Andridge R, et al. A troubled heart: Mood disorder history longitudinally predicts faster cardiopulmonary aging in breast cancer survivorship. *PLoS One* 2023; **18**(3): e0283849.

42. Mehnert A, Veers S, Howaldt D, Braumann KM, Koch U, Schulz KH. Effects of a physical exercise rehabilitation group program on anxiety, depression, body image, and health-related quality of life among breast cancer patients. *Onkologie* 2011; **34**(5): 248-53.

43. Hooshmand Moghadam B, Golestani F, Bagheri R, et al. The effects of high-intensity interval training vs. Moderate-intensity continuous training on inflammatory markers, body composition, and physical fitness in overweight/obese survivors of breast cancer: A randomized controlled clinical trial. *Cancers* 2021; **13**(17).

44. Nuri R, Kordi MR, Moghaddasi M, Rahnama N, Damirchi A, Rahmani-Nia F, Emami H. Effect of combination exercise training on metabolic syndrome parameters in postmenopausal women with breast cancer. *Journal of cancer research and therapeutics* 2012; **8**(2): 238-42.

45. Ochi E, Tsuji K, Narisawa T, et al. Cardiorespiratory fitness in breast cancer survivors: a randomised controlled trial of home-based smartphone supported high intensity interval training. *BMJ Support Palliat Care* 2022; **12**(1): 33-7.

46. Pagola I, Morales JS, Alejo LB, et al. Concurrent Exercise Interventions in Breast Cancer Survivors with Cancer-related Fatigue. *Int J Sports Med* 2020; **41**(11): 790-7.

47. Peck SS, Esmaeilzadeh M, Rankin K, et al. Self-Reported Physical Activity, QoL, Cardiac Function, and Cardiorespiratory Fitness in Women With HER2+ Breast Cancer. *JACC: CardioOncology* 2022; **4**(3): 387-400.

48. Scharhag-Rosenberger F, Kuehl R, Klassen O, et al. Exercise training intensity prescription in breast cancer survivors: validity of current practice and specific recommendations. *Journal of cancer survivorship : research and practice* 2015; **9**(4): 612-9.

49. Scott JM, Iyengar NM, Nilsen TS, et al. Feasibility, safety, and efficacy of aerobic training in pretreated patients with metastatic breast cancer: A randomized controlled trial. *Cancer* 2018; **124**(12): 2552-60.

50. Scott JM, Lee J, Herndon JE, et al. Timing of exercise therapy when initiating adjuvant chemotherapy for breast cancer: a randomized trial. 2023; **44**(46): 4878‐89.

51. Siripanya S, Parinyanitikul N, Tanaka H, Suksom D. Home-Based Buddhist Walking Meditation Mitigates Cardiotoxicity of Anthracycline Chemotherapy in Breast Cancer Patients: A Randomized Controlled Trial. *Journal of Integrative & Complementary Medicine* 2023; **29**(9): 562-73.

52. Smoot B, Zerzan S, Krasnoff J, Wong J, Cho M, Dodd M. Upper extremity bioimpedance before and after treadmill testing in women post breast cancer treatment. *Breast cancer research and treatment* 2014; **148**(2): 445-53.

53. Suesada MM, Carvalho HA, Albuquerque ALP, Salge JM, Stuart SR, Takagaki TY. Impact of thoracic radiotherapy on respiratory function and exercise capacity in patients with breast cancer. 2018; **44**(6): 469.

54. Swisher AK, Abraham J, Bonner D, et al. Exercise and dietary advice intervention for survivors of triple-negative breast cancer: effects on body fat, physical function, quality of life, and adipokine profile. *Support Care Cancer* 2015; **23**(10): 2995-3003.

55. Travier N, Velthuis MJ, Steins Bisschop CN, et al. Effects of an 18-week exercise programme started early during breast cancer treatment: a randomised controlled trial. *BMC Med* 2015; **13**: 121.

56. Tubiana-Mathieu N, Cornette T, Mandigout S, Leobon S, Vincent F, Venat L, Deluche E. Can the six-minute walk test be used to individualize physical activity intensity in patients with breast cancer? *Cancers* 2021; **13**(22).

57. Uth J, Fristrup B, Sørensen V, et al. Exercise intensity and cardiovascular health outcomes after 12 months of football fitness training in women treated for stage I-III breast cancer: Results from the football fitness After Breast Cancer (ABC) randomized controlled trial. *Prog Cardiovasc Dis* 2020; **63**(6): 792-9.

58. Vincent F, Deluche E, Bonis J, et al. Home-Based Physical Activity in Patients With Breast Cancer: during and/or After Chemotherapy? Impact on Cardiorespiratory Fitness. A 3-Arm Randomized Controlled Trial (APAC). *Integrative cancer therapies* 2020; **19**.

59. Zvinovski F, Stephens JA, Ramaswamy B, et al. A Cardiac Rehabilitation Program for Breast Cancer Survivors: A Feasibility Study. *Journal of Oncology* 2021; **2021**((Zvinovski F., filadelfiya.zvinovski@osumc.edu; Ramaswamy B., bhuvaneswari.ramaswamy@osumc.edu; Reinbolt R.E., raquel.reinbolt@osumc.edu; Noonan A.M., anne.noonan@osumc.edu; Vandeusen J.B., jeffrey.vandeusen@osumc.edu; Wesolowski R., robert.wesolowski@osu).

60. Leensen MCJ, Groeneveld IF, van der Heide I, et al. Return to work of cancer patients after a multidisciplinary intervention including occupational counselling and physical exercise in cancer patients: a prospective study in the Netherlands. *BMJ Open* 2017; **7**(6): e014746.

61. Martin EA, Battaglini CL, Hands B, Naumann F. Higher-Intensity Exercise Results in More Sustainable Improvements for VO2peak for Breast and Prostate Cancer Survivors. 2015; **42**(3): 241.

62. Schneider J, Schluter K, Sprave T, Wiskemann J, Rosenberger F. Exercise intensity prescription in cancer survivors: ventilatory and lactate thresholds are useful submaximal alternatives to VO2peak. 2020.

63. Berkel AEM, Bongers BC, Kotte H, et al. Effects of Community-based Exercise Prehabilitation for Patients Scheduled for Colorectal Surgery With High Risk for Postoperative Complications: Results of a Randomized Clinical Trial. *Ann Surg* 2022; **275**(2): e299-e306.

64. Bolshinsky V, Ismail H, Li M, et al. Clinical covariates that improve surgical risk prediction and guide targeted prehabilitation: an exploratory, retrospective cohort study of major colorectal cancer surgery patients evaluated with preoperative cardiopulmonary exercise testing. *Perioperative Medicine* 2022; **11**(1).

65. Challand C, Struthers R, Sneyd JR, Erasmus PD, Mellor N, Hosie KB, Minto G. Randomized controlled trial of intraoperative goal-directed fluid therapy in aerobically fit and unfit patients having major colorectal surgery. *British journal of anaesthesia* 2012; **108**(1): 53-62.

66. Chan KE, Pathak S, Smart NJ. The impact of cardiopulmonary exercise testing on patients over the age of 80 undergoing elective colorectal cancer surgery. 2016; **18**(6): 578-85.

67. Christensen JF, Simonsen C, Banck-Petersen A, et al. Safety and feasibility of preoperative exercise training during neoadjuvant treatment before surgery for adenocarcinoma of the gastro-oesophageal junction. *BJS Open* 2019; **3**(1): 74-84.

68. Cramer L, Hildebrandt B, Kung T, et al. Cardiovascular function and predictors of exercise capacity in patients with colorectal cancer. 2014; **64**(13): 1310.

69. Devin JL, Sax AT, Hughes GI, et al. The influence of high-intensity compared with moderate-intensity exercise training on cardiorespiratory fitness and body composition in colorectal cancer survivors: a randomised controlled trial. *J Cancer Surviv* 2016; **10**(3): 467-79.

70. Exarchou K, Patel S, Barrow H, Lunevicius R, Arthur JD. Laparoscopic Surgery Is Safe and Beneficial in True Functional High-Risk Patients with Colorectal Cancer: Utilization of Cardiopulmonary Exercise Test. *J Laparoendosc Adv Surg Tech A* 2020; **30**(11): 1194-203.

71. Franssen RFW, Berkel AEM, Ten Cate DWG, et al. A retrospective analysis of the association of effort-independent cardiopulmonary exercise test variables with postoperative complications in patients who underwent elective colorectal surgery. *Langenbecks Arch Surg* 2023; **409**(1): 7.

72. Heitkamp M, Spanier B, Von Korn P, Knapp S, Groß C, Haller B, Halle M. Feasibility of a 12-Month Exercise Intervention in Postsurgical Colorectal Cancer Patients. *Translational Sports Medicine* 2023; **2023**((Heitkamp M., melanie.heitkamp@mri.tum.de; Spanier B., biancaspanier.bs@gmail.com; Von Korn P., pia.vonkorn@mri.tum.de; Knapp S., knappsebastian@gmx.net; Groß C., claudia.gross@mri.tum.de; Halle M., martin.halle@mri.tum.de) Department of Prevention and Sp).

73. Hossain T, Phillips BE, Doleman B, Lund JN, Williams JP. A double-blind randomized controlled trial of the effects of eicosapentaenoic acid supplementation on muscle inflammation and physical function in patients undergoing colorectal cancer resection. 2020; **39**(7): 2055.

74. Lee L, Schwartzman K, Carli F, et al. The association of the distance walked in 6 min with pre-operative peak oxygen consumption and complications 1 month after colorectal resection. *Anaesthesia* 2013; **68**(8): 811-6.

75. Loughney L, West MA, Moyses H, et al. The effects of neoadjuvant chemoradiotherapy and an in-hospital exercise training programme on physical fitness and quality of life in locally advanced rectal cancer patients: a randomised controlled trial (The EMPOWER Trial). *Perioperative Medicine* 2021; **10**(1).

76. Minnella EM, Ferreira V, Awasthi R, et al. Effect of two different pre-operative exercise training regimens before colorectal surgery on functional capacity: A randomised controlled trial. *Eur J Anaesthesiol* 2020; **37**(11): 969-78.

77. Morielli AR, Usmani N, Boule NG, et al. Feasibility, Safety, and Preliminary Efficacy of Exercise During and After Neoadjuvant Rectal Cancer Treatment: a Phase II Randomized Controlled Trial. *Clinical colorectal cancer* 2021.

78. Pinto BM, Papandonatos GD, Goldstein MG, Marcus BH, Farrell N. Home-based physical activity intervention for colorectal cancer survivors. *Psychooncology* 2013; **22**(1): 54-64.

79. Rose GA, Davies RG, Davison GW, et al. The cardiopulmonary exercise test grey zone; optimising fitness stratification by application of critical difference. *British Journal of Anaesthesia* 2018; **120**(6): 1187-94.

80. Sellar CM, Bell GJ, Haennel RG, Au HJ, Chua N, Courneya KS. Feasibility and efficacy of a 12-week supervised exercise intervention for colorectal cancer survivors. 2014; **39**(6): 715.

81. West MA, Lythgoe D, Barben CP, Noble L, Kemp GJ, Jack S, Grocott MP. Cardiopulmonary exercise variables are associated with postoperative morbidity after major colonic surgery: a prospective blinded observational study. *British journal of anaesthesia* 2014; **112**(4): 665-71.

82. West MA, Loughney L, Barben CP, Sripadam R, Kemp GJ, Grocott MP, Jack S. The effects of neoadjuvant chemoradiotherapy on physical fitness and morbidity in rectal cancer surgery patients. *European journal of surgical oncology : the journal of the European Society of Surgical Oncology and the British Association of Surgical Oncology* 2014; **40**(11): 1421-8.

83. West MA, Parry MG, Lythgoe D, Barben CP, Kemp GJ, Grocott MP, Jack S. Cardiopulmonary exercise testing for the prediction of morbidity risk after rectal cancer surgery. *Br J Surg* 2014; **101**(9): 1166-72.

84. Wilson RJT, Yates DRA, Walkington JP, Davies SJ. Ventilatory inefficiency adversely affects outcomes and longer-term survival after planned colorectal cancer surgery. 2019; **123**(2): 238.

85. Tufo A, Dunne DFJ, Manu N, et al. Hepatectomy for octogenarians with colorectal liver metastasis in the era of enhanced recovery. *European journal of surgical oncology : the journal of the European Society of Surgical Oncology and the British Association of Surgical Oncology* 2018; **44**(7): 1040-7.

86. Astrup Søndergaard MM, Nordsmark M, Sloth Møller D, Melgaard Nielsen K, Poulsen SH. Reduction in myocardial function and oxygen consumption after chemoradiotherapy in patients with esophageal cancer. *Acta Oncol* 2022; **61**(5): 566-74.

87. Benington S, Bryan A, Milne O, Alkhaffaf B. CPET and cardioesophagectomy: A single centre 10-year experience. *European journal of surgical oncology : the journal of the European Society of Surgical Oncology and the British Association of Surgical Oncology* 2019; **45**(12): 2451-6.

88. Chang Y, Tsai Y, Hsu C, Chao Y, Lin K. The effectiveness of a nurse-led exercise and health education informatics program on exercise capacity and quality of life among cancer survivors after esophagectomy: a randomized controlled trial. 2020; **101**: N.PAG.

89. Drummond RJ, Vass D, Wadhawan H, Craig CF, MacKay CK, Fullarton GM, Forshaw MJ. Routine pre- and post-neoadjuvant chemotherapy fitness testing is not indicated for oesophagogastric cancer surgery. *Annals of the Royal College of Surgeons of England* 2018; **100**(7): 515-9.

90. Forshaw MJ, Strauss DC, Davies AR, et al. Is cardiopulmonary exercise testing a useful test before esophagectomy? *The Annals of thoracic surgery* 2008; **85**(1): 294-9.

91. Jack S, West MA, Raw D, et al. The effect of neoadjuvant chemotherapy on physical fitness and survival in patients undergoing oesophagogastric cancer surgery. *European journal of surgical oncology : the journal of the European Society of Surgical Oncology and the British Association of Surgical Oncology* 2014; **40**(10): 1313-20.

92. Lam S, Alexandre L, Hardwick G, Hart AR. The association between preoperative cardiopulmonary exercise-test variables and short-term morbidity after esophagectomy: A hospital-based cohort study. *Surgery* 2019; **166**(1): 28-33.

93. Moyes LH, McCaffer CJ, Carter RC, Fullarton GM, Mackay CK, Forshaw MJ. Cardiopulmonary exercise testing as a predictor of complications in oesophagogastric cancer surgery. *Annals of the Royal College of Surgeons of England* 2013; **95**(2): 125-30.

94. Navidi M, Phillips AW, Griffin SM, Duffield KE, Greystoke A, Sumpter K, Sinclair RCF. Cardiopulmonary fitness before and after neoadjuvant chemotherapy in patients with oesophagogastric cancer. *British Journal of Surgery* 2018; **105**(7): 900-6.

95. O'Neill LM, Guinan E, Doyle SL, et al. The RESTORE Randomized Controlled Trial: Impact of a Multidisciplinary Rehabilitative Program on Cardiorespiratory Fitness in Esophagogastric cancer Survivorship. 2018; **268**(5): 747.

96. Ozova M, Chomakhidze P, Poltavskaya M, Vychuzhanin D, Kopylov P, Saner H, Andreev D. Cardiopulmonary exercise testing for cardiovascular risk assessment in patients undergoing gastric and oesophageal cancer surgery: results from a prospective interventional cohort study. *Open Heart* 2022; **9**(2).

97. Patel N, Powell AG, Wheat JR, et al. Cardiopulmonary fitness predicts postoperative major morbidity after esophagectomy for patients with cancer. 2019; **7**(14): e14174.

98. Sinclair RCF, Phillips AW, Navidi M, Griffin SM, Snowden CP. Pre-operative variables including fitness associated with complications after oesophagectomy. *Anaesthesia* 2017; **72**(12): 1501-7.

99. Thomson IG, Wallen MP, Hall A, et al. Neoadjuvant therapy reduces cardiopulmunary function in patients undegoing oesophagectomy. *International journal of surgery (London, England)* 2018; **53**: 86-92.

100. van Vulpen JK, Hiensch AE, van Hillegersberg R, et al. Supervised exercise after oesophageal cancer surgery: the PERFECT multicentre randomized clinical trial. *British journal of surgery* 2021.

101. West MA, Baker WC, Rahman S, et al. Cardiopulmonary exercise testing has greater prognostic value than sarcopenia in oesophago-gastric cancer patients undergoing neoadjuvant therapy and surgical resection. *J Surg Oncol* 2021; **124**(8): 1306-16.

102. Hyltander A, Bosaeus I, Svedlund J, et al. Supportive nutrition on recovery of metabolism, nutritional state, health-related quality of life, and exercise capacity after major surgery: a randomized study. *Clinical gastroenterology and hepatology : the official clinical practice journal of the American Gastroenterological Association* 2005; **3**(5): 466-74.

103. Cho I, Son Y, Song S, et al. Feasibility and effects of a postoperative recovery exercise program developed specifically for gastric cancer patients (PREP-GC) undergoing minimally invasive gastrectomy. 2018; **18**(2): 118.

104. Sinclair R, Navidi M, Griffin SM, Sumpter K. The impact of neoadjuvant chemotherapy on cardiopulmonary physical fitness in gastro-oesophageal adenocarcinoma. *Annals of the Royal College of Surgeons of England* 2016; **98**(6): 396-400.

105. Lindman A, Handberg C, Olesen G, Duijts S. Health‐related quality of life and physical functioning in patients participating in a rehabilitation programme, undergoing non‐myeloablative allogeneic haematopoietic stem cell transplantation: Outcomes from a single arm longitudinal study. *European Journal of Cancer Care* 2021; **30**(6): 1-17.

106. van Wijk L, van der Snee L, Buis CI, Hentzen JEKR, Haveman ME, Klaase JM. A prospective cohort study evaluating screening and assessment of six modifiable risk factors in HPB cancer patients and compliance to recommended prehabilitation interventions. *Perioperative Medicine* 2021; **10**(1).

107. Caru M, Samoilenko M, Drouin S, et al. Childhood Acute Lymphoblastic Leukemia Survivors Have a Substantially Lower Cardiorespiratory Fitness Level Than Healthy Canadians Despite a Clinically Equivalent Level of Physical Activity. 2019; **8**(6): 674.

108. Jarvela LS, Niinikoski H, Lahteenmaki PM, Heinonen OJ, Kapanen J, Arola M, Kemppainen J. Physical activity and fitness in adolescent and young adult long-term survivors of childhood acute lymphoblastic leukaemia. *Journal of cancer survivorship : research and practice* 2010; **4**(4): 339-45.

109. Labonte J, Caru M, Lemay V, et al. Developing and validating equations to predict [Formula: see text]O2 peak from the 6MWT in Childhood ALL Survivors. 2020: 1‐8.

110. Lemay V, Caru M, Samoilenko M, et al. Physical Activity and Sedentary Behaviors in Childhood Acute Lymphoblastic Leukemia Survivors. 2020; **42**(1): 53.

111. Long TM, Lee F, Lam K, Wallman KE, Walwyn TS, Choong CS, Naylor LH. Cardiovascular Testing Detects Underlying Dysfunction in Childhood Leukemia Survivors. 2020; **52**(3): 525.

112. Myrdal OH, Kongerud J, Sikkeland LI, et al. Risk factors for impaired pulmonary function and cardiorespiratory fitness in very long-term adult survivors of childhood acute lymphoblastic leukemia after treatment with chemotherapy only. *Acta Oncologica* 2018; **57**(5): 658-64.

113. Myrdal OH, Diep PP, Ruud E, et al. Determinants of cardiorespiratory fitness in very long-term survivors of allogeneic hematopoietic stem cell transplantation: a national cohort study. 2020.

114. Phillips NS, Howell CR, Lanctot JQ, et al. Physical fitness and neurocognitive outcomes in adult survivors of childhood acute lymphoblastic leukemia: A report from the St. Jude Lifetime cohort. 2020; **126**(3): 640.

115. Tonorezos ES, Snell PG, Moskowitz CS, et al. Reduced cardiorespiratory fitness in adult survivors of childhood acute lymphoblastic leukemia. *Pediatric blood & cancer* 2013; **60**(8): 1358-64.

116. Yeon SH, Lee MW, Duong TT, et al. Cardiopulmonary Exercise Test With Comorbidity Index Before Allogeneic Hematopoietic Stem Cell Transplantation. *Integr Cancer Ther* 2022; **21**: 15347354221134249.

117. Armenian SH, Horak D, Scott JM, et al. Cardiovascular Function in Long-Term Hematopoietic Cell Transplantation Survivors. *Biology of blood and marrow transplantation : journal of the American Society for Blood and Marrow Transplantation* 2017; **23**(4): 700-5.

118. Bayram S, Bargi G, Celik Z, Bosnak Guclu M. Effects of pulmonary rehabilitation in hematopoietic stem cell transplantation recipients: a randomized controlled study. 2023; **32**(1): 72.

119. Ishikawa A, Otaka Y, Kamisako M, et al. Factors affecting lower limb muscle strength and cardiopulmonary fitness after allogeneic hematopoietic stem cell transplantation. 2019; **27**(5): 1793.

120. Kirsten J, Wais V, Schulz SVW, Sala E, Treff G, Bunjes D, Steinacker JM. Sarcopenia screening allows identifying high-risk patients for allogenic stem cell transplantation. *Cancers* 2021; **13**(8).

121. Dunne DF, Jack S, Jones RP, et al. Randomized clinical trial of prehabilitation before planned liver resection. *Br J Surg* 2016; **103**(5): 504-12.

122. Kaibori M, Ishizaki M, Matsui K, et al. Assessment of preoperative exercise capacity in hepatocellular carcinoma patients with chronic liver injury undergoing hepatectomy. *BMC Gastroenterol* 2013; **13**: 119.

123. Kaibori M, Matsui K, Yoshii K, Ishizaki M, Iwasaka J, Miyauchi T, Kimura Y. Perioperative exercise capacity in chronic liver injury patients with hepatocellular carcinoma undergoing hepatectomy. 2019; **14**(8): e0221079.

124. Batchelor N, Daniels IR, Thangakunam B, Samuel J, Ibrahim B, Aitchison D. Lack of Utility of Thoracoscore in Evaluating Fitness for Surgery in Lung Cancer. *Colorectal disease : the official journal of the Association of Coloproctology of Great Britain and Ireland The Indian journal of chest diseases & allied sciences* 2015; **57**(1): 13-5.

125. Beccaria M, Corsico A, Fulgoni P, Zoia MC, Casali L, Orlandoni G, Cerveri I. Lung cancer resection: the prediction of postsurgical outcomes should include long-term functional results. *Chest* 2001; **120**(1): 37-42.

126. Bechard D, Wetstein L. Assessment of exercise oxygen consumption as preoperative criterion for lung resection. 1987; **44**(4): 344.

127. Begum SS, Papagiannopoulos K, Falcoz PE, Decaluwe H, Salati M, Brunelli A. Outcome after video-assisted thoracoscopic surgery and open pulmonary lobectomy in patients with low VO2 max: a case-matched analysis from the ESTS databasedagger. *European journal of cardio-thoracic surgery : official journal of the European Association for Cardio-thoracic Surgery* 2016; **49**(4): 1054-8; discussion 8.

128. Bhatia C, Kayser B. Preoperative high-intensity interval training is effective and safe in deconditioned patients with lung cancer: A randomized clinical trial. 2019; **51**(9): 712.

129. Bobbio A, Chetta A, Internullo E, et al. Exercise capacity assessment in patients undergoing lung resection. *European journal of cardio-thoracic surgery : official journal of the European Association for Cardio-thoracic Surgery* 2009; **35**(3): 419-22.

130. Bolliger CT, Wyser C, Roser H, Soler M, Perruchoud AP. Lung scanning and exercise testing for the prediction of postoperative performance in lung resection candidates at increased risk for complications. *Chest* 1995; **108**(2): 341-8.

131. Boujibar F, Bonnevie T, Debeaumont D, et al. Impact of prehabilitation on morbidity and mortality after pulmonary lobectomy by minimally invasive surgery: A cohort study. *Journal of Thoracic Disease* 2018; **10**(4): 2240-8.

132. Brunelli A, Belardinelli R, Refai M, Salati M, Socci L, Pompili C, Sabbatini A. Peak oxygen consumption during cardiopulmonary exercise test improves risk stratification in candidates to major lung resection. *Chest* 2009; **135**(5): 1260-7.

133. Brunelli A, Pompili C, Salati M, Refai M, Berardi R, Mazzanti P, Tiberi M. Preoperative maximum oxygen consumption is associated with prognosis after pulmonary resection in stage I non-small cell lung cancer. *Ann Thorac Surg* 2014; **98**(1): 238-42.

134. Brutsche MH, Spiliopoulos A, Bolliger CT, Licker M, Frey JG, Tschopp JM. Exercise capacity and extent of resection as predictors of surgical risk in lung cancer. *Eur Respir J* 2000; **15**(5): 828-32.

135. Campione A, Terzi A, Bobbio M, Rosso GL, Scardovi AB, Feola M. Oxygen pulse as a predictor of cardiopulmonary events in lung resection. *Asian Cardiovasc Thorac Ann* 2010; **18**(2): 147-52.

136. Cavalheri V, Jenkins S, Cecins N, Gain K, Hill K. Comparison of the six-minute walk test with a cycle-based cardiopulmonary exercise test in people following curative intent treatment for non-small cell lung cancer. *Chronic respiratory disease* 2016; **13**(2): 118-27.

137. Chouinard G, Roy P, Blais MC, et al. Exercise testing and postoperative complications after minimally invasive lung resection: A cohort study. *Frontiers in Physiology* 2022; **13**((Chouinard G.; Roy P.; Blais M.-C.; Lippens A.; Pelletier É.; Roy E.; Marcoux M.; Ugalde P.A.; Rheault J.; Pigeon M.-A.; Nicodème F.; Lacasse Y.; Maltais F., francois.maltais@med.ulaval.ca) Institut universitaire de cardiologie et de pneumologie de Québec).

138. Colman NC, Schraufnagel DE, Rivington RN, Pardy RL. Exercise testing in evaluation of patients for lung resection. 1982; **125**(5): 604.

139. Dun Y, Cui N, Wu S, et al. Cardiorespiratory fitness and morbidity and mortality in patients with non-small cell lung cancer: a prospective study with propensity score weighting. *Ann Med* 2023; **55**(2): 2295981.

140. Edvardsen E, Anderssen SA, Borchsenius F, Skjonsberg OH. Reduction in cardiorespiratory fitness after lung resection is not related to the number of lung segments removed. *BMJ Open Sport Exerc Med* 2015; **1**(1): e000032.

141. Edvardsen E, Skjonsberg OH, Holme I, Nordsletten L, Borchsenius F, Anderssen SA. High-intensity training following lung cancer surgery: a randomised controlled trial. *Thorax* 2015; **70**(3): 244-50.

142. Epstein SK, Faling LJ, Daly BD, Celli BR. Inability to perform bicycle ergometry predicts increased morbidity and mortality after lung resection. *Chest* 1995; **107**(2): 311-6.

143. Fang Y, Ma G, Lou N, Liao W, Wang D. Preoperative Maximal Oxygen Uptake and Exercise-induced Changes in Pulse Oximetry Predict Early Postoperative Respiratory Complications in Lung Cancer Patients. *Scandinavian journal of surgery : SJS : official organ for the Finnish Surgical Society and the Scandinavian Surgical Society* 2014; **103**(3): 201-8.

144. Fernandez-Rodriguez L, Torres I, Romera D, et al. Prediction of postoperative lung function after major lung resection for lung cancer using volumetric computed tomography. *The Journal of thoracic and cardiovascular surgery* 2018; **156**(6): 2297-308 e5.

145. Fresard I, Licker M, Adler D, Lovis A, Robert J, Karenovics W, Diaper J. Preoperative Peak Oxygen Uptake in Lung Cancer Subjects With Neoadjuvant Chemotherapy: A Cross-Sectional Study. *Respiratory Care* 2016; **61**(8): 1059-66.

146. Gao T, Luo JL, Guo P, Hu XW, Wei XY, Hu Y. Impact of continuous care on cardiac function in patients with lung cancer complicated by coronary heart disease. *World Journal of Clinical Cases* 2024; **12**(2): 314-21.

147. Granger CL, Denehy L, Parry SM, Martin J, Dimitriadis T, Sorohan M, Irving L. Which field walking test should be used to assess functional exercise capacity in lung cancer? An observational study. *BMC pulmonary medicine* 2015; **15**: 89.

148. Gravier FE, Bonnevie T, Boujibar F, et al. Cardiopulmonary exercise testing in patients with non-small cell lung cancer: Trust the VO2peak? *Journal of Thoracic Disease* 2020; **12**(10): 5313-23.

149. Gravier F-E, Smondack P, Boujibar F, et al. Prehabilitation sessions can be provided more frequently in a shortened regimen with similar or better efficacy in people with non-small cell lung cancer: a randomised trial. *Journal of Physiotherapy (Elsevier)* 2022; **68**(1): 43-50.

150. Hwang CL, Yu CJ, Shih JY, Yang PC, Wu YT. Effects of exercise training on exercise capacity in patients with non-small cell lung cancer receiving targeted therapy. *Supportive care in cancer : official journal of the Multinational Association of Supportive Care in Cancer* 2012; **20**(12): 3169-77.

151. Janssen SM, Abbink JJ, Lindeboom R, Vliet Vlieland TP. Outcomes of Pulmonary Rehabilitation After Treatment for Non-Small Cell Lung Cancer Stages I to IIIa: AN OBSERVATIONAL STUDY. *Journal of cardiopulmonary rehabilitation and prevention* 2017; **37**(1): 65-71.

152. Jones LW, Peddle CJ, Eves ND, et al. Effects of presurgical exercise training on cardiorespiratory fitness among patients undergoing thoracic surgery for malignant lung lesions. *Cancer* 2007; **110**(3): 590-8.

153. Jones LW, Watson D, Herndon JE, Eves ND, Haithcock BE, Loewen G, Kohman L. Peak oxygen consumption and long-term all-cause mortality in nonsmall cell lung cancer. *Cancer* 2010; **116**(20): 4825-32.

154. Kasikcioglu E, Toker A, Tanju S, Arzuman P, Kayserilioglu A, Dilege S, Kalayci G. Oxygen uptake kinetics during cardiopulmonary exercise testing and postoperative complications in patients with lung cancer. *Lung Cancer* 2009; **66**(1): 85-8.

155. Kristenson K, Hylander J, Boros M, Hedman K. VE/VCO2 slope threshold optimization for preoperative evaluation in lung cancer surgery: identifying true high- and low-risk groups. *Journal of Thoracic Disease* 2024; **16**(1): 123-32.

156. Kushibe K, Kawaguchi T, Kimura M, Takahama M, Tojo T, Taniguchi S. Changes in ventilatory capacity, exercise capacity, and pulmonary blood flow after lobectomy in patients with lung cancer--which lobectomy has the most loss in exercise capacity? *Interactive cardiovascular and thoracic surgery* 2008; **7**(6): 1011-4.

157. Kushibe K, Kawaguchi T, Kimura M, Takahama M, Tojo T, Taniguchi S. Exercise capacity after lobectomy in patients with chronic obstructive pulmonary disease. *Interactive cardiovascular and thoracic surgery* 2008; **7**(3): 398-401.

158. Larsen KR, Svendsen UG, Milman N, Brenoe J, Petersen BN. Cardiopulmonary function at rest and during exercise after resection for bronchial carcinoma. *The Annals of thoracic surgery* 1997; **64**(4): 960-4.

159. Larsen KR, Lund JO, Svendsen UG, Milman N, Petersen BN. Prediction of post-operative cardiopulmonary function using perfusion scintigraphy in patients with bronchogenic carcinoma. *Clin Physiol* 1997; **17**(3): 257-67.

160. Licker M, Schnyder JM, Frey JG, et al. Impact of aerobic exercise capacity and procedure-related factors in lung cancer surgery. *The European respiratory journal* 2011; **37**(5): 1189-98.

161. Lindenmann J, Fink-Neuboeck N, Fediuk M, et al. Preoperative Peak Oxygen Consumption: A Predictor of Survival in Resected Lung Cancer. *Cancers (Basel)* 2020; **12**(4).

162. Loewen GM, Watson D, Kohman L, et al. Preoperative exercise Vo2 measurement for lung resection candidates: results of Cancer and Leukemia Group B Protocol 9238. *Journal of thoracic oncology : official publication of the International Association for the Study of Lung Cancer* 2007; **2**(7): 619-25.

163. Marhic A, Dakhil B, Plantefeve G, Zaimi R, Oltean V, Bagan P. Long-term survival following lung surgery for cancer in high-risk patients after perioperative pulmonary rehabilitation. 2019; **28**(2): 235.

164. Markos J, Mullan BP, Hillman DR, et al. Preoperative assessment as a predictor of mortality and morbidity after lung resection. *The American review of respiratory disease* 1989; **139**(4): 902-10.

165. Marulli G, Rea F, Nicotra S, et al. Effect of induction chemotherapy on lung function and exercise capacity in patients affected by malignant pleural mesothelioma. *European journal of cardio-thoracic surgery : official journal of the European Association for Cardio-thoracic Surgery* 2010; **37**(6): 1464-9.

166. Matzi V, Lindenmann J, Muench A, et al. The impact of preoperative micronutrient supplementation in lung surgery. A prospective randomized trial of oral supplementation of combined alpha-ketoglutaric acid and 5-hydroxymethylfurfural. *European journal of cardio-thoracic surgery : official journal of the European Association for Cardio-thoracic Surgery* 2007; **32**(5): 776-82.

167. Minnella EM, Baldini G, Quang ATL, Bessissow A, Spicer J, Carli F. Prehabilitation in Thoracic Cancer Surgery: From Research to Standard of Care. *J Cardiothorac Vasc Anesth* 2021; **35**(11): 3255-64.

168. Miyazaki T, Callister MEJ, Franks K, Dinesh P, Nagayasu T, Brunelli A. Minute ventilation-to-carbon dioxide slope is associated with postoperative survival after anatomical lung resection. *Lung Cancer* 2018; **125**: 218-22.

169. Nagamatsu Y, Takamori S, Hayashida R, Yamana H, Shirouzu K. Pulmonary capacity in lung cancer patients prior to lung resection--comparison of the unilateral pulmonary artery occlusion test with expired gas analysis during exercise testing. *The Kurume medical journal* 1996; **43**(4): 273-7.

170. Nagamatsu Y, Shima I, Hayashi A, Yamana H, Shirouzu K, Ishitake T. Preoperative spirometry versus expired gas analysis during exercise testing as predictors of cardiopulmonary complications after lung resection. *Surgery today* 2004; **34**(2): 107-10.

171. Nagamatsu Y, Iwasaki Y, Hayashida R, et al. Factors related to an early restoration of exercise capacity after major lung resection. *Surgery today* 2011; **41**(9): 1228-33.

172. Nezu K, Kushibe K, Tojo T, Takahama M, Kitamura S. Recovery and limitation of exercise capacity after lung resection for lung cancer. *Chest* 1998; **113**(6): 1511-6.

173. Novoa NM, Varela G, Jimenez MF, Ramos J. Value of the average basal daily walked distance measured using a pedometer to predict maximum oxygen consumption per minute in patients undergoing lung resection. *European journal of cardio-thoracic surgery : official journal of the European Association for Cardio-thoracic Surgery* 2011; **39**(5): 756-62.

174. Nugent AM, Steele IC, Carragher AM, et al. Effect of thoracotomy and lung resection on exercise capacity in patients with lung cancer. *Thorax* 1999; **54**(4): 334-8.

175. Op den Kamp CM, Langen RC, Minnaard R, et al. Pre-cachexia in patients with stages I-III non-small cell lung cancer: systemic inflammation and functional impairment without activation of skeletal muscle ubiquitin proteasome system. *Lung Cancer* 2012; **76**(1): 112-7.

176. Perrotta F, Cennamo A, Cerqua FS, et al. Effects of a high-intensity pulmonary rehabilitation program on the minute ventilation/carbon dioxide output slope during exercise in a cohort of patients with COPD undergoing lung resection for non-small cell lung cancer. 2019; **45**(6): e20180132.

177. Pierce RJ, Copland JM, Sharpe K, Barter CE. Preoperative risk evaluation for lung cancer resection: predicted postoperative product as a predictor of surgical mortality. *American journal of respiratory and critical care medicine* 1994; **150**(4): 947-55.

178. Quist M, Sommer MS, Vibe-Petersen J, et al. Early initiated postoperative rehabilitation reduces fatigue in patients with operable lung cancer: A randomized trial. 2018; **126**: 125.

179. Quist M, Langer SW, Lillelund C, et al. Effects of an exercise intervention for patients with advanced inoperable lung cancer undergoing chemotherapy: a randomized clinical trial. 2020; **145**: 76‐82.

180. Ribas J, Jimenez MJ, Barbera JA, Roca J, Gomar C, Canalis E, Rodriguez-Roisin R. Gas exchange and pulmonary hemodynamics during lung resection in patients at increased risk: relationship with preoperative exercise testing. *Chest* 2001; **120**(3): 852-9.

181. Rocco G, Gatani T, Di Maio M, et al. The impact of decreasing cutoff values for maximal oxygen consumption (VO(2)max) in the decision-making process for candidates to lung cancer surgery. *J Thorac Dis* 2013; **5**(1): 12-8.

182. Rodrigues F, Grafino M, Faria I, Pontes da Mata J, Papoila AL, Felix F. Surgical risk evaluation of lung cancer in COPD patients - A cohort observational study. *Revista portuguesa de pneumologia* 2016; **22**(5): 266-72.

183. Rushwan A, Stefanou D, Tariq J, et al. Increased minute ventilation-to-carbon dioxide slope during cardiopulmonary exercise test is associated with poor postoperative outcome following lung cancer resection. *Eur J Cardiothorac Surg* 2024; **65**(3).

184. Salhi B, Haenebalcke C, Perez-Bogerd S, et al. Rehabilitation in patients with radically treated respiratory cancer: A randomised controlled trial comparing two training modalities. *Lung Cancer* 2015; **89**(2): 167-74.

185. Shafiek H, Valera JL, Togores B, Torrecilla JA, Sauleda J, Cosio BG. Risk of postoperative complications in chronic obstructive lung diseases patients considered fit for lung cancer surgery: beyond oxygen consumption. *European journal of cardio-thoracic surgery : official journal of the European Association for Cardio-thoracic Surgery* 2016; **50**(4): 772-9.

186. Stanzani F, Paisani Dde M, Oliveira A, Souza RC, Perfeito JA, Faresin SM. Morbidity, mortality, and categorization of the risk of perioperative complications in lung cancer patients. *Jornal brasileiro de pneumologia : publicacao oficial da Sociedade Brasileira de Pneumologia e Tisilogia* 2014; **40**(1): 21-9.

187. Stefanelli F, Meoli I, Cobuccio R, et al. High-intensity training and cardiopulmonary exercise testing in patients with chronic obstructive pulmonary disease and non-small-cell lung cancer undergoing lobectomy. *Eur J Cardiothorac Surg* 2013; **44**(4): e260-5.

188. Torchio R, Guglielmo M, Giardino R, et al. Exercise ventilatory inefficiency and mortality in patients with chronic obstructive pulmonary disease undergoing surgery for non-small-cell lung cancer. *Eur J Cardiothorac Surg* 2010; **38**(1): 14-9.

189. Torchio R, Mazzucco A, Guglielmo M, Giardino R, Ciacco C, Ardissone F. Minute ventilation to carbon dioxide output (V'E/V'CO2 slope) is the strongest death predictor before larger lung resections. *Monaldi archives for chest disease = Archivio Monaldi per le malattie del torace* 2017; **87**(3): 817.

190. Umezu H, Miyoshi S, Araki O, et al. Efficacy of functional operability algorithm for octogenarians with primary lung cancer. *General thoracic and cardiovascular surgery* 2012; **60**(1): 36-42.

191. Vargas Fajardo Mdel C, Novoa Valentin NM, Jimenez Lopez MF, Ramos Gonzalez J, Varela Simo G. An alternative method for predicting the risk of postoperative complications in lung resection. *Arch Bronconeumol* 2014; **50**(3): 87-92.

192. Villani F, Busia A. Preoperative evaluation of patients submitted to pneumonectomy for lung carcinoma: role of exercise testing. *Tumori* 2004; **90**(4): 405-9.

193. Walsh GL, Morice RC, Putnam JB, et al. Resection of lung cancer is justified in high-risk patients selected by exercise oxygen consumption. *The Annals of thoracic surgery* 1994; **58**(3): 704-10; discussion 11.

194. Wang J, Olak J, Ultmann RE, Ferguson MK. Assessment of pulmonary complications after lung resection. *The Annals of thoracic surgery* 1999; **67**(5): 1444-7.

195. Wang JS, Abboud RT, Evans KG, Finley RJ, Graham BL. Role of CO diffusing capacity during exercise in the preoperative evaluation for lung resection. *American journal of respiratory and critical care medicine* 2000; **162**(4 Pt 1): 1435-44.

196. Wang JS, Abboud RT, Wang LM. Effect of lung resection on exercise capacity and on carbon monoxide diffusing capacity during exercise. *Chest* 2006; **129**(4): 863-72.

197. Wang JS, Abboud RT, Graham BL. Predicted postoperative product and diffusion heterogeneity index in the evaluation of candidates for lung resection. 2011; **56**(4): 449.

198. Win T, Jackson A, Sharples L, Groves AM, Wells FC, Ritchie AJ, Laroche CM. Cardiopulmonary exercise tests and lung cancer surgical outcome. *Chest* 2005; **127**(4): 1159-65.

199. Win T, Jackson A, Groves AM, Sharples LD, Charman SC, Laroche CM. Comparison of shuttle walk with measured peak oxygen consumption in patients with operable lung cancer. *Thorax* 2006; **61**(1): 57-60.

200. Win T, Sharples L, Groves AM, Ritchie AJ, Wells FC, Laroche CM. Predicting survival in potentially curable lung cancer patients. *Lung* 2008; **186**(2): 97-102.

201. Puente-Maestu L, Villar F, Gonzalez-Casurran G, et al. Early and long-term validation of an algorithm assessing fitness for surgery in patients with postoperative FEV(1) and diffusing capacity of the lung for carbon monoxide < 40%. *Chest* 2011; **139**(6): 1430-8.

202. Courneya KS, Sellar CM, Stevinson C, et al. Randomized controlled trial of the effects of aerobic exercise on physical functioning and quality of life in lymphoma patients. *Journal of clinical oncology : official journal of the American Society of Clinical Oncology* 2009; **27**(27): 4605-12.

203. Elbl L, Vasova I, Tomaskova I, Jedlicka F, Navratil M, Pospisil Z, Vorlicek J. Cardiac function and cardiopulmonary performance in patients after treatment for non-Hodgkin's lymphoma. *Neoplasma* 2006; **53**(2): 174-81.

204. Elbl L, Vasova I, Tomaskova I, et al. Cardiopulmonary exercise testing in the evaluation of functional capacity after treatment of lymphomas in adults. *Leukemia & lymphoma* 2006; **47**(5): 843-51.

205. Jain B, Floreani AA, Anderson JR, Vose JM, Robbins RA, Rennard SI, Sisson JH. Cardiopulmonary function and autologous bone marrow transplantation: results and predictive value for respiratory failure and mortality. The University of Nebraska Medical Center Bone Marrow Transplantation Pulmonary Study Group. *Bone marrow transplantation* 1996; **17**(4): 561-8.

206. Murbraech K, Holte E, Broch K, et al. Impaired Right Ventricular Function in Long-Term Lymphoma Survivors. *Journal of the American Society of Echocardiography : official publication of the American Society of Echocardiography* 2016; **29**(6): 528-36.

207. Rizwan R, Gauvreau K, Vinograd C, et al. VO2peak in Adult Survivors of Hodgkin Lymphoma: Rate of Decline, Sex Differences, and Cardiovascular Events. *JACC: CardioOncology* 2021; **3**(2): 263-73.

208. Vermaete N, Wolter P, Verhoef G, Gosselink R. Physical activity and physical fitness in lymphoma patients before, during, and after chemotherapy: a prospective longitudinal study. *Annals of hematology* 2014; **93**(3): 411-24.

209. Persoon S, Kersten MJ, Buffart LM, et al. Health-related physical fitness in patients with multiple myeloma or lymphoma recently treated with autologous stem cell transplantation. *Journal of science and medicine in sport* 2017; **20**(2): 116-22.

210. Armand A, Rochette E, Grèze V, et al. Fitness and metabolic response to exercise in young adult survivors of childhood lymphoma. *Support Care Cancer* 2023; **31**(6): 358.

211. Atkinson M, Murnane A, Goddard T, Pendergrast C, Rogers P, Manudhane R, Osborn M. A randomized controlled trial of a structured exercise intervention after the completion of acute cancer treatment in adolescents and young adults. *Pediatr Blood Cancer* 2021; **68**(1): e28751.

212. Bertheussen GF, Oldervoll L, Kaasa S, Sandmael JA, Helbostad JL. Measurement of physical activity in cancer survivors--a comparison of the HUNT 1 Physical Activity Questionnaire (HUNT 1 PA-Q) with the International Physical Activity Questionnaire (IPAQ) and aerobic capacity. *Supportive care in cancer : official journal of the Multinational Association of Supportive Care in Cancer* 2013; **21**(2): 449-58.

213. Bjorke ACH, Raastad T, Berntsen S. Criteria for the determination of maximal oxygen uptake in patients newly diagnosed with cancer: Baseline data from the randomized controlled trial of physical training and cancer (Phys-Can). 2020; **15**(6): e0234507.

214. Bjørke ACH, Raastad T, Berntsen S. Criteria for the determination of maximal oxygen uptake in patients newly diagnosed with cancer: baseline data from the randomized controlled trial of physical training and cancer (Phys-Can). *PloS one* 2020; **15**(6): e0234507.

215. Carannante A, Attanasio U, Cuomo A, et al. Evaluation of exercise capacity by means of cardiopulmonary exercise testing (CPET) in older adult cancer patients undergoing antineoplastic treatments. *Journal of Gerontology and Geriatrics* 2023; **71**(4): 207-17.

216. Courneya KS, Jones LW, Peddle CJ, et al. Effects of aerobic exercise training in anemic cancer patients receiving darbepoetin alfa: a randomized controlled trial. *Oncologist* 2008; **13**(9): 1012-20.

217. Daneryd P, Svanberg E, Korner U, et al. Protection of metabolic and exercise capacity in unselected weight-losing cancer patients following treatment with recombinant erythropoietin: a randomized prospective study. *Cancer research* 1998; **58**(23): 5374-9.

218. De Backer IC, Schep G, Hoogeveen A, Vreugdenhil G, Kester AD, van Breda E. Exercise testing and training in a cancer rehabilitation program: the advantage of the steep ramp test. *Archives of physical medicine and rehabilitation* 2007; **88**(5): 610-6.

219. De Backer IC, Van Breda E, Vreugdenhil A, Nijziel MR, Kester AD, Schep G. High-intensity strength training improves quality of life in cancer survivors. *Acta oncologica (Stockholm, Sweden)* 2007; **46**(8): 1143-51.

220. De Backer IC, Vreugdenhil G, Nijziel MR, Kester AD, van Breda E, Schep G. Long-term follow-up after cancer rehabilitation using high-intensity resistance training: persistent improvement of physical performance and quality of life. *British journal of cancer* 2008; **99**(1): 30-6.

221. Demmelmaier I, Brooke HL, Henriksson A, et al. Does exercise intensity matter for fatigue during (neo-)adjuvant cancer treatment? The Phys-Can randomized clinical trial. *Scand J Med Sci Sports* 2021; **31**(5): 1144-59.

222. Goodenough CG, Wogksch MD, Kundu M, et al. Associations between exercise capacity, p16INK4a expression and inflammation among adult survivors of childhood cancer. *Frontiers in Oncology* 2022; **12**((Goodenough C.G.; Wogksch M.D.; Wang Z.; Armstrong G.T.; Hudson M.M.; Robison L.L.; Ness K.K., kiri.ness@stjude.org) Department of Epidemiology and Cancer Control, St. Jude Children’s Research Hospital, Memphis, TN, United States(Kundu M.) Department of C).

223. Griffith K, Wenzel J, Shang J, Thompson C, Stewart K, Mock V. Impact of a walking intervention on cardiorespiratory fitness, self-reported physical function, and pain in patients undergoing treatment for solid tumors. *Cancer* 2009; **115**(20): 4874-84.

224. Henriksson A, Strandberg E, Stenling A, et al. Does inflammation markers or treatment type moderate exercise intensity effects on changes in muscle strength in cancer survivors participating in a 6-month combined resistance- and endurance exercise program? Results from the Phys-Can trial. *BMC Sports Science, Medicine and Rehabilitation* 2023; **15**(1).

225. Huang GH, Ismail H, Murnane A, Kim P, Riedel B. Structured exercise program prior to major cancer surgery improves cardiopulmonary fitness: a retrospective cohort study. *Supportive care in cancer : official journal of the Multinational Association of Supportive Care in Cancer* 2016; **24**(5): 2277-85.

226. Jones LW, Douglas PS, Khouri MG, et al. Safety and efficacy of aerobic training in patients with cancer who have heart failure: an analysis of the HF-ACTION randomized trial. *Journal of clinical oncology : official journal of the American Society of Clinical Oncology* 2014; **32**(23): 2496-502.

227. Kampshoff CS, Chinapaw MJ, Brug J, et al. Randomized controlled trial of the effects of high intensity and low-to-moderate intensity exercise on physical fitness and fatigue in cancer survivors: results of the Resistance and Endurance exercise After ChemoTherapy (REACT) study. *BMC medicine* 2015; **13**: 275.

228. Kampshoff CS, van Dongen JM, van Mechelen W, et al. Long-term effectiveness and cost-effectiveness of high versus low-to-moderate intensity resistance and endurance exercise interventions among cancer survivors. *Journal of cancer survivorship : research and practice* 2018; **12**(3): 417-29.

229. Kenjale AA, Hornsby WE, Crowgey T, et al. Pre-exercise participation cardiovascular screening in a heterogeneous cohort of adult cancer patients. *The oncologist* 2014; **19**(9): 999-1005.

230. Kerrigan DJ, Reddy M, Walker EM, et al. Cardiac Rehabilitation Improves Fitness in Patients With Subclinical Markers of Cardiotoxicity While Receiving Chemotherapy: a RANDOMIZED CONTROLLED STUDY. 2023; **43**(2): 129‐34.

231. Klika RJ, Golik KS, Drum SN, Callahan KE, Thorland WG. Comparison of physiological response to cardiopulmonary exercise testing among cancer survivors and healthy controls. *European journal of applied physiology* 2011; **111**(6): 1167-76.

232. Li MHG, Bolshinsky V, Ismail H, Ho KM, Heriot A, Riedel B. Comparison of Duke Activity Status Index with cardiopulmonary exercise testing in cancer patients. *Journal of Anesthesia* 2018; **32**(4): 576-84.

233. Lundholm K, Daneryd P, Bosaeus I, Korner U, Lindholm E. Palliative nutritional intervention in addition to cyclooxygenase and erythropoietin treatment for patients with malignant disease: Effects on survival, metabolism, and function. *Cancer* 2004; **100**(9): 1967-77.

234. May AM, Van Weert E, Korstjens I, et al. Improved physical fitness of cancer survivors: a randomised controlled trial comparing physical training with physical and cognitive-behavioural training. *Acta oncologica (Stockholm, Sweden)* 2008; **47**(5): 825-34.

235. May AM, van Weert E, Korstjens I, et al. Monitoring training progress during exercise training in cancer survivors: a submaximal exercise test as an alternative for a maximal exercise test? *Archives of physical medicine and rehabilitation* 2010; **91**(3): 351-7.

236. Midtgaard J, Christensen JF, Tolver A, et al. Efficacy of multimodal exercise-based rehabilitation on physical activity, cardiorespiratory fitness, and patient-reported outcomes in cancer survivors: a randomized, controlled trial. 2013; **24**(9): 2267.

237. Nadruz W, West E, Sengeløv M, et al. Cardiovascular phenotype and prognosis of patients with heart failure induced by cancer therapy. *Heart* 2018.

238. Nawoor-Quinn Z, Oliver A, Raobaikady R, Mohammad K, Cone S, Kasivisvanathan R. The Marsden Morbidity Index: the derivation and validation of a simple risk index scoring system using cardiopulmonary exercise testing variables to predict morbidity in high-risk patients having major cancer surgery. *Perioperative Medicine* 2022; **11**(1).

239. Ness KK, Plana JC, Joshi VM, et al. Exercise Intolerance, Mortality, and Organ System Impairment in Adult Survivors of Childhood Cancer. *Journal of clinical oncology : official journal of the American Society of Clinical Oncology* 2020; **38**(1): 29-42.

240. O'Mahony M, Mohammed K, Kasivisvanathan R. Cardiopulmonary Exercise Testing Versus Frailty, Measured by the Clinical Frailty Score, in Predicting Morbidity in Patients Undergoing Major Abdominal Cancer Surgery. *World J Surg* 2021; **45**(1): 116-25.

241. Pahl A, Wehrle A, Kneis S, Gollhofer A, Bertz H. Whole body vibration training during allogeneic hematopoietic cell transplantation-the effects on patients' physical capacity. 2020; **99**(3): 635.

242. Pein F, Sakiroglu O, Dahan M, et al. Cardiac abnormalities 15 years and more after adriamycin therapy in 229 childhood survivors of a solid tumour at the Institut Gustave Roussy. 2004; **91**(1): 37.

243. Pillinger NL, Koh CE, Ansari N, Munoz PA, McNamara SG, Steffens D. Preoperative cardiopulmonary exercise testing improves risk assessment of morbidity and length of stay following cytoreductive surgery with hyperthermic intraperitoneal chemotherapy. *Anaesth Intensive Care* 2022; **50**(6): 447-56.

244. Prinsen H, Bleijenberg G, Heijmen L, et al. The role of physical activity and physical fitness in postcancer fatigue: a randomized controlled trial. *Supportive care in cancer : official journal of the Multinational Association of Supportive Care in Cancer* 2013; **21**(8): 2279-88.

245. Santa Mina D, Au D, Papadopoulos E, et al. Aerobic capacity attainment and reasons for cardiopulmonary exercise test termination in people with cancer: a descriptive, retrospective analysis from a single laboratory. *Support Care Cancer* 2020; **28**(9): 4285-94.

246. Schmidt K, Vogt L, Thiel C, Jager E, Banzer W. Validity of the six-minute walk test in cancer patients. *Int J Sports Med* 2013; **34**(7): 631-6.

247. Schmitt J, Lindner N, Reuss-Borst M, Holmberg HC, Sperlich B. A 3-week multimodal intervention involving high-intensity interval training in female cancer survivors: a randomized controlled trial. 2016; **4**(3).

248. Schneider C, Ryffel C, Stütz L, et al. Supervised exercise training in patients with cancer during anthracycline-based chemotherapy to mitigate cardiotoxicity: a randomized-controlled-trial. *Frontiers in Cardiovascular Medicine* 2023; **10**((Schneider C.; Stütz L.; Eser P.; Wilhelm M., matthias.wilhelm@insel.ch) Centre for Rehabilitation & Sports Medicine, Inselspital, Bern University Hospital, University of Bern, Bern, Switzerland(Schneider C.) Graduate School for Health Sciences, Universit).

249. Stuiver MM, Kampshoff CS, Persoon S, et al. Validation and Refinement of Prediction Models to Estimate Exercise Capacity in Cancer Survivors Using the Steep Ramp Test. *Archives of physical medicine and rehabilitation* 2017; **98**(11): 2167-73.

250. van der Schoot GGF, Ormel HL, Westerink NDL, et al. Optimal Timing of a Physical Exercise Intervention to Improve Cardiorespiratory Fitness: During or After Chemotherapy. *JACC: CardioOncology* 2022; **4**(4): 491-503.

251. Viamonte SG, Joaquim AV, Alves AJ, et al. Cardio-Oncology Rehabilitation for Cancer Survivors With High Cardiovascular Risk: A Randomized Clinical Trial. *JAMA Cardiol* 2023; **8**(12): 1119-28.

252. Waterland JL, Ismail H, Granger CL, et al. Prehabilitation in high-risk patients scheduled for major abdominal cancer surgery: a feasibility study. *Perioperative Medicine* 2022; **11**(1).

253. Weemaes ATR, Beelen M, Bongers BC, Weijenberg MP, Lenssen AF. Criterion Validity and Responsiveness of the Steep Ramp Test to Evaluate Aerobic Capacity in Survivors of Cancer Participating in a Supervised Exercise Rehabilitation Program. *Arch Phys Med Rehabil* 2021; **102**(11): 2150-6.

254. van Weert E, Hoekstra-Weebers JE, Grol BM, Otter R, Arendzen JH, Postema K, van der Schans CP. Physical functioning and quality of life after cancer rehabilitation. *International journal of rehabilitation research Internationale Zeitschrift fur Rehabilitationsforschung Revue internationale de recherches de readaptation* 2004; **27**(1): 27-35.

255. Wiestad TH, Raastad T, Nordin K, Igelström H, Henriksson A, Demmelmaier I, Berntsen S. The Phys-Can observational study: Adjuvant chemotherapy is associated with a reduction whereas physical activity level before start of treatment is associated with maintenance of maximal oxygen uptake in patients with cancer. *BMC Sports Science, Medicine and Rehabilitation* 2020; **12**(1).

256. Koutoukidis DA, Land J, Hackshaw A, et al. Fatigue, quality of life and physical fitness following an exercise intervention in multiple myeloma survivors (MASCOT): an exploratory randomised Phase 2 trial utilising a modified Zelen design. 2020.

257. van Dongen JM, Persoon S, Jongeneel G, et al. Long-term effectiveness and cost-effectiveness of an 18-week supervised exercise program in patients treated with autologous stem cell transplantation: results from the EXIST study. 2019; **13**(4): 558.

258. Lee J-K, Park S, Jee Y-S. Immunoprotecting Effects of Exercise Program against Ovarian Cancer: A Single-Blind, Randomized Controlled Trial. *Cancers* 2022; **14**(11): 2808-.

259. Pinelli C, Morotti M, Casarin J, et al. Interval Debulking Surgery for Advanced Ovarian Cancer in Elderly Patients (≥70 y): Does the Age Matter? *J Invest Surg* 2021; **34**(9): 1023-30.

260. Smits A, Agius CM, Blake D, et al. Is Cardiopulmonary Exercise Testing Predictive of Surgical Complications in Patients Undergoing Surgery for Ovarian Cancer? *Cancers* 2023; **15**(21).

261. Ausania F, Vallance AE, Manas DM, et al. Double bypass for inoperable pancreatic malignancy at laparotomy: postoperative complications and long-term outcome. *Annals of the Royal College of Surgeons of England* 2012; **94**(8): 563-8.

262. Chandrabalan VV, McMillan DC, Carter R, Kinsella J, McKay CJ, Carter CR, Dickson EJ. Pre-operative cardiopulmonary exercise testing predicts adverse post-operative events and non-progression to adjuvant therapy after major pancreatic surgery. *HPB : the official journal of the International Hepato Pancreato Biliary Association* 2013; **15**(11): 899-907.

263. Junejo MA, Mason JM, Sheen AJ, et al. Cardiopulmonary exercise testing for preoperative risk assessment before pancreaticoduodenectomy for cancer. *Annals of surgical oncology* 2014; **21**(6): 1929-36.

264. Alberga AS, Segal RJ, Reid RD, et al. Age and androgen-deprivation therapy on exercise outcomes in men with prostate cancer. *Supportive care in cancer : official journal of the Multinational Association of Supportive Care in Cancer* 2012; **20**(5): 971-81.

265. Bonsignore A, Field D, Speare R, Dolan L, Oh P, Santa Mina D. The Effect of Cardiac Rehabilitation in Men With and Without Prostate Cancer: A Retrospective, Comparative Cohort Study. *J Phys Act Health* 2018; **15**(10): 781-7.

266. Djurhuus SS, Simonsen C, Toft BG, et al. Exercise training to increase tumour natural killer-cell infiltration in men with localised prostate cancer: a randomised controlled trial. 2023; **131**(1): 116‐24.

267. Eriksen AK, Hansen RD, Borre M, et al. A lifestyle intervention among elderly men on active surveillance for non-aggressive prostate cancer: a randomised feasibility study with whole-grain rye and exercise. *Trials* 2017; **18**(1): 20.

268. Harrison MR, Davis PG, Khouri MG, et al. A randomized controlled trial comparing changes in fitness with or without supervised exercise in patients initiated on enzalutamide and androgen deprivation therapy for non-metastatic castration-sensitive prostate cancer (EXTEND). 2022; **25**(1): 58‐64.

269. Hojan K, Kwiatkowska-Borowczyk E, Leporowska E, Milecki P. Inflammation, cardiometabolic markers, and functional changes in men with prostate cancer: A randomized controlled trial of a 12-month exercise program. *Polish Archives of Internal Medicine* 2017; **127**(1): 25-35.

270. Houben LHP, Overkamp M, Van Kraaij P, et al. Resistance Exercise Training Increases Muscle Mass and Strength in Prostate Cancer Patients on Androgen Deprivation Therapy. *Medicine & Science in Sports & Exercise* 2023; **55**(4): 614-24.

271. Jones LW, Hornsby WE, Freedland SJ, et al. Effects of nonlinear aerobic training on erectile dysfunction and cardiovascular function following radical prostatectomy for clinically localized prostate cancer. *Eur Urol* 2014; **65**(5): 852-5.

272. Kang D-W, Fairey AS, Boulé NG, Field CJ, Wharton SA, Courneya KS. Effects of Exercise on Cardiorespiratory Fitness and Biochemical Progression in Men With Localized Prostate Cancer Under Active Surveillance: The ERASE Randomized Clinical Trial. *JAMA Oncology* 2021; **7**(10): 1487-95.

273. Ndjavera W, Orange ST, O'Doherty AF, Leicht AS, Rochester M, Mills R, Saxton JM. Exercise-induced attenuation of treatment side-effects in patients with newly diagnosed prostate cancer beginning androgen-deprivation therapy: a randomised controlled trial. 2020; **125**(1): 28.

274. Scott JM, Hornsby WE, Lane A, Kenjale AA, Eves ND, Jones LW. Reliability of maximal cardiopulmonary exercise testing in men with prostate cancer. *Medicine and science in sports and exercise* 2015; **47**(1): 27-32.

275. Segal RJ, Reid RD, Courneya KS, et al. Randomized controlled trial of resistance or aerobic exercise in men receiving radiation therapy for prostate cancer. *J Clin Oncol* 2009; **27**(3): 344-51.

276. Uth J, Hornstrup T, Schmidt JF, et al. Football training improves lean body mass in men with prostate cancer undergoing androgen deprivation therapy. *Scandinavian Journal of Medicine & Science in Sports* 2014; **24**(4): 105-12.

277. Uth J, Fristrup B, Haahr RD, et al. Football training over 5 years is associated with preserved femoral bone mineral density in men with prostate cancer. 2018; **28 Suppl 1**: 61.

278. Van Blarigan EL, Kenfield SA, Olshen A, et al. Effect of a Home-based Walking Intervention on Cardiopulmonary Fitness and Quality of Life Among Men with Prostate Cancer on Active Surveillance: the Active Surveillance Exercise Randomized Controlled Trial. 2023.

279. Adams SC, DeLorey DS, Davenport MH, et al. Effects of high-intensity aerobic interval training on cardiovascular disease risk in testicular cancer survivors: A phase 2 randomized controlled trial. 2017; **123**(20): 4057-65.

280. Hyltander A, Drott C, Unsgaard B, Tolli J, Korner U, Arfvidsson B, Lundholm K. The effect on body composition and exercise performance of home parenteral nutrition when given as adjunct to chemotherapy of testicular carcinoma. *European journal of clinical investigation* 1991; **21**(4): 413-20.

281. Minnella EM, Awasthi R, Bousquet-Dion G, et al. Multimodal Prehabilitation to Enhance Functional Capacity Following Radical Cystectomy: a Randomized Controlled Trial. 2019.

282. Pieczyńska A, Pilarska A, Adamska K, Zasadzka E, Hojan K. Psychological Characteristics Associated with Post-Treatment Physical Status and Quality of Life in Patients with Brain Tumor Undergoing Radiotherapy. *Journal of Personalized Medicine* 2022; **12**(11).

283. Pieczyńska A, Zasadzka E, Pilarska A, Procyk D, Adamska K, Hojan K. Rehabilitation Exercises Supported by Monitor-Augmented Reality for Patients with High-Grade Glioma Undergoing Radiotherapy: Results of a Randomized Clinical Trial. *Journal of Clinical Medicine* 2023; **12**(21).

284. Ruden E, Reardon DA, Coan AD, et al. Exercise behavior, functional capacity, and survival in adults with malignant recurrent glioma. *Journal of clinical oncology : official journal of the American Society of Clinical Oncology* 2011; **29**(21): 2918-23.

285. Ariza-Garcia A, Lozano-Lozano M, Galiano-Castillo N, Postigo-Martin P, Arroyo-Morales M, Cantarero-Villanueva I. A Web-Based Exercise System (e-CuidateChemo) to Counter the Side Effects of Chemotherapy in Patients With Breast Cancer: Randomized Controlled Trial. 2019; **21**(7): e14418.

286. Bellissimo MP, Canada JM, Jordan JH, et al. Physical Activity During Breast Cancer Therapy Associates With Preserved Exercise Capacity and Cardiac Function (WF97415). *JACC: CardioOncology* 2023; **5**(5): 641-52.

287. Brahmbhatt P, Sabiston CM, Lopez C, et al. Feasibility of Prehabilitation Prior to Breast Cancer Surgery: A Mixed-Methods Study. *Frontiers in Oncology* 2020; **10**.

288. Buchan J, Janda M, Box R, Schmitz K, Hayes S. A Randomized Trial on the Effect of Exercise Mode on Breast Cancer-Related Lymphedema. *Medicine and science in sports and exercise* 2016; **48**(10): 1866-74.

289. CeŠEiko R, Thomsen SN, Tomsone S, et al. Heavy Resistance Training in Breast Cancer Patients Undergoing Adjuvant Therapy. 2020; **52**(6): 1239.

290. Hojan K, Procyk D, Horynska-Kestowicz D, Leporowska E, Litwiniuk M. The preventive role of regular physical training in ventricular remodeling, serum cardiac markers, and exercise performance changes in breast cancer in women undergoing trastuzumab therapy—an reh-her study. 2020; **9**(5).

291. Kaltsatou A, Mameletzi D, Douka S. Physical and psychological benefits of a 24-week traditional dance program in breast cancer survivors. *J Bodyw Mov Ther* 2011; **15**(2): 162-7.

292. Kim DY, Kim JH, Park SW. Aerobic capacity correlates with health-related quality of life after breast cancer surgery. *European journal of cancer care* 2019; **28**(4): e13050.

293. Kokkonen K, Saarto T, Makinen T, Pohjola L, Kautio H, Jarvenpaa S, Puustjarvi-Sunabacka K. The functional capacity and quality of life of women with advanced breast cancer. *Breast cancer (Tokyo, Japan)* 2017; **24**(1): 128-36.

294. Kulkarni N, Mahajan AA, Khatri SM. A randomized controlled trial of the effectiveness of aerobic training for patients with breast cancer undergoing radiotherapy. *Journal of the Association of Chartered Physiotherapists in Women's Health* 2013; (113): 42-50.

295. Mustian KM, Palesh OG, Flecksteiner SA. Tai Chi Chuan for breast cancer survivors. *Med Sport Sci* 2008; **52**: 209-17.

296. Ortiz A, Tirado M, Hughes DC, Gonzalez V, Song J, Mama SK, Basen-Engquist K. Relationship between physical activity, disability, and physical fitness profile in sedentary Latina breast cancer survivors. *Physiotherapy Theory & Practice* 2018; **34**(10): 783-94.

297. Ortiz A, Hughes DC, Mama SK, et al. Effectiveness of a Home-Based Exercise Intervention in the Fitness Profile of Hispanic Survivors of Breast Cancer. *Rehabilitation Oncology* 2021; **39**(4): 175-83.

298. Prieto-Gómez V, Yuste-Sánchez MJ, Bailón-Cerezo J, Romay-Barrero H, de la Rosa-Díaz I, Lirio-Romero C, Torres-Lacomba M. Effectiveness of Therapeutic Exercise and Patient Education on Cancer-Related Fatigue in Breast Cancer Survivors: A Randomised, Single-Blind, Controlled Trial with a 6-Month Follow-Up. *Journal of Clinical Medicine* 2022; **11**(1).

299. Reis D, Walsh ME, Young-McCaughan S, Jones T. Effects of Nia Exercise in Women Receiving Radiation Therapy for Breast Cancer. *Clinical Journal of Oncology Nursing* 2013; **17**: E374-82.

300. Santagnello SB, Martins FM, e Oliveira Junior GN, e Freitas Rodrigues de Sousa J, Nomelini RS, Murta EFC, Orsatti FL. Improvements in muscle strength, power, and size and self-reported fatigue as mediators of the effect of resistance exercise on physical performance breast cancer survivor women: a randomized controlled trial. 2020.

301. Vardar-Yagli N, Sener G, Saglam M, et al. Associations among physical activity, comorbidity, functional capacity, peripheral muscle strength and depression in breast cancer survivors. *Asian Pacific journal of cancer prevention : APJCP* 2015; **16**(2): 585-9.

302. Wang YJ, Boehmke M, Wu YW, Dickerson SS, Fisher N. Effects of a 6-week walking program on Taiwanese women newly diagnosed with early-stage breast cancer. *Cancer nursing* 2011; **34**(2): E1-13.

303. Winters-Stone KM, Torgrimson-Ojerio B, Dieckmann NF, Stoyles S, Mitri Z, Luoh SW. A randomized-controlled trial comparing supervised aerobic training to resistance training followed by unsupervised exercise on physical functioning in older breast cancer survivors. *J Geriatr Oncol* 2022; **13**(2): 152-60.

304. Bousquet-Dion G, Awasthi R, Loiselle SÈ, et al. Evaluation of supervised multimodal prehabilitation programme in cancer patients undergoing colorectal resection: a randomized control trial. *Acta Oncologica* 2018; **57**(6): 849-59.

305. Chen BP, Awasthi R, Sweet SN, et al. Four-week prehabilitation program is sufficient to modify exercise behaviors and improve preoperative functional walking capacity in patients with colorectal cancer. *Support Care Cancer* 2017; **25**(1): 33-40.

306. Chen SC, Huang HP, Huang WS, Lin YC, Chu TP, Beaton RD, Jane SW. Non-randomized preliminary study of an education and elastic-band resistance exercise program on severity of neuropathy, physical function, muscle strength and endurance & quality of life in colorectal cancer patients experiencing oxaliplatin-induced peri. *Eur J Oncol Nurs* 2020; **49**: 101834.

307. Courneya KS, Vardy JL, O'Callaghan CJ, et al. Effects of a Structured Exercise Program on Physical Activity and Fitness in Colon Cancer Survivors: One Year Feasibility Results from the CHALLENGE Trial. *Cancer epidemiology, biomarkers & prevention : a publication of the American Association for Cancer Research, cosponsored by the American Society of Preventive Oncology* 2016; **25**(6): 969-77.

308. Gillis C, Li C, Lee L, et al. Prehabilitation versus rehabilitation: a randomized control trial in patients undergoing colorectal resection for cancer. *Anesthesiology* 2014; **121**(5): 937-47.

309. Northgraves MJ, Arunachalam L, Madden LA, Marshall P, Hartley JE, MacFie J, Vince RV. Feasibility of a novel exercise prehabilitation programme in patients scheduled for elective colorectal surgery: a feasibility randomised controlled trial. 2020; **28**(7): 3197.

310. Pecorelli N, Fiore JF, Jr., Gillis C, et al. The six-minute walk test as a measure of postoperative recovery after colorectal resection: further examination of its measurement properties. *Surg Endosc* 2016; **30**(6): 2199-206.

311. Soares-Miranda L, Lucia A, Silva M, et al. Physical Fitness and Health-related Quality of Life in Patients with Colorectal Cancer. *Int J Sports Med* 2021; **42**(10): 924-9.

312. Wang X, Chen R, Ge L, et al. Effect of short-term prehabilitation of older patients with colorectal cancer: A propensity score-matched analysis. *Frontiers in Oncology* 2023; **13**((Wang X.; Gu Y.; Zhang L.; Wang L.; Zhuang C.; Wu Q., qian_wu2010@163.com) Department of Gastrointestinal Surgery, Shanghai Tenth People’s Hospital Affiliated to Tongji University, Shanghai, China(Wang X.; Chen R.; Ge L.) College of Medicine, Tongji Unive).

313. Zimmer P, Trebing S, Timmers-Trebing U, et al. Eight-week, multimodal exercise counteracts a progress of chemotherapy-induced peripheral neuropathy and improves balance and strength in metastasized colorectal cancer patients: a randomized controlled trial. 2018; **26**(2): 615‐24.

314. Li Q, Wang Y, Jin T, Lu L, Tong Y. Effect of trimodal pre-rehabilitation on the rehabilitation of patients with gastrointestinal tumors in the perioperative period. *American Journal of Translational Research* 2022; **14**(2): 967-78.

315. Eriksson F, Trölle U, Becovic S, Hermansson M, Olsén MF. The relationship between measures of physical performance in preoperative oesophageal cancer patients. *European Journal of Physiotherapy* 2021; **23**(3): 144-8.

316. Guinan EM, Doyle SL, Bennett AE, et al. Sarcopenia during neoadjuvant therapy for oesophageal cancer: characterising the impact on muscle strength and physical performance. *Supportive Care in Cancer* 2018; **26**(5): 1569-76.

317. Guinan EM, Bennett AE, Doyle SL, et al. Measuring the impact of oesophagectomy on physical functioning and physical activity participation: a prospective study. 2019; **19**(1): 682.

318. Inoue T, Ito S, Ando M, et al. Changes in exercise capacity, muscle strength, and health-related quality of life in esophageal cancer patients undergoing esophagectomy. *BMC Sports Sci Med Rehabil* 2016; **8**: 34.

319. Minnella EM, Awasthi R, Loiselle SE, Agnihotram RV, Ferri LE, Carli F. Effect of Exercise and Nutrition Prehabilitation on Functional Capacity in Esophagogastric Cancer Surgery: A Randomized Clinical Trial. *JAMA Surg* 2018; **153**(12): 1081-9.

320. Otani T, Ichikawa H, Hanyu T, et al. Long-Term Trends in Respiratory Function After Esophagectomy for Esophageal Cancer. 2020; **245**: 168.

321. Sugimura K, Miyata H, Kanemura T, et al. Impact of preoperative skeletal muscle mass and physical performance on short-term and long-term postoperative outcomes in patients with esophageal cancer after esophagectomy. *Annals of Gastroenterological Surgery* 2022; **6**(5): 623-32.

322. Tan QY, Wang RW, Jiang YG, et al. Lung volume reduction surgery allows esophageal tumor resection in selected esophageal carcinoma with severe emphysema. *The Annals of thoracic surgery* 2006; **82**(5): 1849-56.

323. Tang A, Ahmad U, Raja S, et al. Looking beyond the eyeball test: A novel vitality index to predict recovery after esophagectomy. *J Thorac Cardiovasc Surg* 2021; **161**(3): 822-32.e6.

324. Eden MM, Tompkins J, Verheijde JL. Reliability and a correlational analysis of the 6MWT, ten-meter walk test, thirty second sit to stand, and the linear analog scale of function in patients with head and neck cancer. *Physiotherapy theory and practice* 2018; **34**(3): 202-11.

325. Samuel SR, Maiya GA, Babu AS, Vidyasagar MS. Effect of exercise training on functional capacity & quality of life in head & neck cancer patients receiving chemoradiotherapy. *Indian J Med Res* 2013; **137**(3): 515-20.

326. Samuel SR, Maiya AG, Fernandes DJ, et al. Effectiveness of exercise-based rehabilitation on functional capacity and quality of life in head and neck cancer patients receiving chemo-radiotherapy. 2019; **27**(10): 3913.

327. Su TL, Chen AN, Leong CP, Huang YC, Chiang CW, Chen IH, Lee YY. The effect of home-based program and outpatient physical therapy in patients with head and neck cancer: a randomized, controlled trial. *Oral oncology* 2017; **74**: 130‐4.

328. Yen CJ, Hung CH, Kao CL, et al. Multimodal exercise ameliorates exercise responses and body composition in head and neck cancer patients receiving chemotherapy. 2019; **27**(12): 4687.

329. Hayashi K, Yokoyama Y, Nakajima H, et al. Preoperative 6-minute walk distance accurately predicts postoperative complications after operations for hepato-pancreato-biliary cancer. *Surgery* 2017; **161**(2): 525-32.

330. Keerthana AK, Kumaresan A, Suganthirababu P, Vishnuram S, Srinivasan V, kumar P, Alagesan J. Effect of Task Oriented Approach on Cancer Related Fatigue and Functional Capacity among Subjects with Blood Leukaemia. *Indian Journal of Physiotherapy & Occupational Therapy*; **18**: 340-5.

331. Labonté J, Caru M, Lemay V, et al. Developing and validating equations to predict V˙O2 peak from the 6MWT in Childhood ALL Survivors. *Disability & Rehabilitation* 2021; **43**(20): 2937-44.

332. Morris GS, Bruedly KE, Scheetz JS. Physical Performance Outcomes of Stem Cell Transplant Recipients Do Not Reflect Their Exposure to Glucocorticoids. *Rehabilitation Oncology* 2011; **29**(3): 9-13.

333. Ness KK, Hudson MM, Pui CH, et al. Neuromuscular impairments in adult survivors of childhood acute lymphoblastic leukemia: associations with physical performance and chemotherapy doses. *Cancer* 2012; **118**(3): 828-38.

334. Timilshina N, Breunis H, Tomlinson GA, Brandwein JM, Buckstein R, Durbano S, Alibhai SMH. Long-term recovery of quality of life and physical function over three years in adult survivors of acute myeloid leukemia after intensive chemotherapy. 2019; **33**(1): 15.

335. Wogksch MD, Finch ER, Nolan VG, et al. Energy cost of walking in obese survivors of acute lymphoblastic leukemia: A report from the St. Jude Lifetime Cohort. *Frontiers in Pediatrics* 2022; **10**((Wogksch M.D.; Finch E.R.; Goodenough C.G.; Mulrooney D.A.; Brinkman T.M.; Lanctot J.Q.; Armstrong G.T.; Robison L.L.; Hudson M.M.; Ness K.K., Kiri.Ness@stjude.org) Department of Epidemiology and Cancer Control, St. Jude Children’s Research Hospital, Memp).

336. Bewarder M, Klostermann A, Ahlgrimm M, Bittenbring JT, Pfreundschuh M, Wagenpfeil S, Kaddu-Mulindwa D. Safety and feasibility of electrical muscle stimulation in patients undergoing autologous and allogeneic stem cell transplantation or intensive chemotherapy. 2019; **27**(3): 1013.

337. DeFeo BM, Kaste SC, Li Z, et al. Long-Term Functional Outcomes Among Childhood Survivors of Cancer Who Have a History of Osteonecrosis. 2020; **100**(3): 509.

338. Jones LW, Devlin SM, Maloy MA, et al. Prognostic Importance of Pretransplant Functional Capacity After Allogeneic Hematopoietic Cell Transplantation. *The oncologist* 2015; **20**(11): 1290-7.

339. Morais NI, Palhares LC, Miranda EC, Lima CS, De Souza CA, Vigorito AC. Effects of a physiotherapeutic protocol in cardiorespiratory, muscle strength, aerobic capacity and quality of life after hematopoietic stem cell transplantation. *Hematology, Transfusion and Cell Therapy* 2023; **45**(2): 154-8.

340. Morishita S, Kaida K, Yamauchi S, et al. Relationship between corticosteroid dose and declines in physical function among allogeneic hematopoietic stem cell transplantation patients. *Supportive care in cancer : official journal of the Multinational Association of Supportive Care in Cancer* 2013; **21**(8): 2161-9.

341. Morishita S, Kaida K, Aoki O, et al. Balance function in patients who had undergone allogeneic hematopoietic stem cell transplantation. *Gait & posture* 2015; **42**(3): 406-8.

342. Morishita S, Kaida K, Yamauchi S, Wakasugi T, Ikegame K, Ogawa H, Domen K. Relationship of physical activity with physical function and health-related quality of life in patients having undergone allogeneic haematopoietic stem-cell transplantation. *European journal of cancer care* 2017; **26**(4).

343. Kawaguchi T, Yoshio S, Sakamoto Y, et al. Impact of decorin on the physical function and prognosis of patients with hepatocellular carcinoma. 2020; **9**(4).

344. Ambrogi V, Baldi A, Schillaci O, Mineo TC. Clinical impact of extrapleural pneumonectomy for malignant pleural mesothelioma. *Annals of surgical oncology* 2012; **19**(5): 1692-9.

345. An N, Dong W, Pang G, Zhang Y, Liu C. TPVB and general anesthesia affects postoperative functional recovery in elderly patients with thoracoscopic pulmonary resections based on ERAS pathway. *Translational Neuroscience* 2023; **14**(1).

346. Andreetti C, Menna C, Ibrahim M, Ciccone AM, D'Andrilli A, Venuta F, Rendina EA. Postoperative pain control: videothoracoscopic versus conservative mini-thoracotomic approach. *European journal of cardio-thoracic surgery : official journal of the European Association for Cardio-thoracic Surgery* 2014; **46**(5): 907-12.

347. Araujo AS, Nogueira IC, Gomes Neto A, et al. The impact of lung cancer resection surgery on fibrinogen and C-reactive protein and their relationship with patients outcomes: A prospective follow up study. *Cancer biomarkers : section A of Disease markers* 2016; **16**(1): 47-53.

348. Ayub T, Jehangir M, Ara F, Wani WA, Umbreen U. 6 Minute Walk Test Performance to Predict Postoperative Pulmonary Complications in Major Oncosurgeries. *International Journal of Toxicological and Pharmacological Research* 2023; **13**(6): 158-67.

349. Bradley A, Marshall A, Stonehewer L, et al. Pulmonary rehabilitation programme for patients undergoing curative lung cancer surgery. *European journal of cardio-thoracic surgery : official journal of the European Association for Cardio-thoracic Surgery* 2013; **44**(4): e266-71.

350. Brocki BC, Westerdahl E, Andreasen JJ, Souza DSR. Improvements in physical performance and health-related quality of life one year after radical operation for lung cancer. *Cancer treatment communications* 2015; **4**: 65‐74.

351. Brocki BC, Andreasen JJ, Langer D, Souza DS, Westerdahl E. Postoperative inspiratory muscle training in addition to breathing exercises and early mobilization improves oxygenation in high-risk patients after lung cancer surgery: a randomized controlled trial. *European journal of cardio-thoracic surgery : official journal of the European Association for Cardio-thoracic Surgery* 2016; **49**(5): 1483-91.

352. Bruera E, Sweeney C, Willey J, Palmer JL, Strasser F, Morice RC, Pisters K. A randomized controlled trial of supplemental oxygen versus air in cancer patients with dyspnea. *Palliative medicine* 2003; **17**(8): 659-63.

353. Chang NW, Lin KC, Lee SC, Chan JY, Lee YH, Wang KY. Effects of an early postoperative walking exercise programme on health status in lung cancer patients recovering from lung lobectomy. *Journal of clinical nursing* 2014; **23**(23-24): 3391-402.

354. Stewart Coats AJ, Ho GF, Prabhash K, et al. Espindolol for the treatment and prevention of cachexia in patients with stage III/IV non-small cell lung cancer or colorectal cancer: a randomized, double-blind, placebo-controlled, international multicentre phase II study (the ACT-ONE trial). *J Cachexia Sarcopenia Muscle* 2016; **7**(3): 355-65.

355. D'Andrilli A, Maurizi G, Andreetti C, et al. Sleeve Lobectomy Versus Standard Lobectomy for Lung Cancer: Functional and Oncologic Evaluation. *The Annals of thoracic surgery* 2016; **101**(5): 1936-42.

356. Denehy L, Hornsby WE, Herndon JE, et al. Prognostic validation of the body mass index, airflow obstruction, dyspnea, and exercise capacity (BODE) index in inoperable non-small-cell lung cancer. *Journal of thoracic oncology : official publication of the International Association for the Study of Lung Cancer* 2013; **8**(12): 1545-50.

357. Deslauriers J, Ugalde P, Miro S, et al. Long-term physiological consequences of pneumonectomy. *Seminars in thoracic and cardiovascular surgery* 2011; **23**(3): 196-202.

358. Dhillon HM, Bell ML, van der Ploeg HP, et al. Impact of physical activity on fatigue and quality of life in people with advanced lung cancer: a randomized controlled trial. *Annals of oncology : official journal of the European Society for Medical Oncology* 2017; **28**(8): 1889-97.

359. Doğan N, Taşcı S. The Effects of Acupressure on Quality of Life and Dyspnea in Lung Cancer: a Randomized, Controlled Trial. 2020; **26**(1): 49‐56.

360. Edbrooke L, Aranda S, Granger CL, et al. Multidisciplinary home-based rehabilitation in inoperable lung cancer: a randomised controlled trial. 2019; **74**(8): 787.

361. Edbrooke L, Granger CL, Clark RA, Denehy L. Physical activity levels are low in inoperable lung cancer: Exploratory analyses from a randomised controlled trial. 2019; **8**(9).

362. Erdogan Y, Gunay E, Ergun P, Kaymaz D, Temiz G, Karaoglanoglu N. Can exercise capacity assessed by the shuttle walk test predict the development of post-operative complications in patients with lung cancer? *Tuberk Toraks* 2013; **61**(1): 28-32.

363. Gao K, Yu PM, Su JH, et al. Cardiopulmonary exercise testing screening and pre-operative pulmonary rehabilitation reduce postoperative complications and improve fast-track recovery after lung cancer surgery: A study for 342 cases. *Thorac Cancer* 2015; **6**(4): 443-9.

364. Glattki GP, Manika K, Sichletidis L, Alexe G, Brenke R, Spyratos D. Pulmonary rehabilitation in non-small cell lung cancer patients after completion of treatment. *American journal of clinical oncology* 2012; **35**(2): 120-5.

365. Goldsmith I, Chesterfield-Thomas G, Toghill H. Pre-treatment optimization with pulmonary rehabilitation in lung cancer: Making the inoperable patients operable. *EClinicalMedicine* 2021; **31**.

366. Granger CL, McDonald CF, Irving L, et al. Low physical activity levels and functional decline in individuals with lung cancer. *Lung Cancer* 2014; **83**(2): 292-9.

367. Granger CL, Holland AE, Gordon IR, Denehy L. Minimal important difference of the 6-minute walk distance in lung cancer. *Chronic respiratory disease* 2015; **12**(2): 146-54.

368. Granger CL, Parry SM, Denehy L. The self-reported Physical Activity Scale for the Elderly (PASE) is a valid and clinically applicable measure in lung cancer. *Supportive care in cancer : official journal of the Multinational Association of Supportive Care in Cancer* 2015; **23**(11): 3211-8.

369. Granger CL, Parry SM, Edbrooke L, Denehy L. Deterioration in physical activity and function differs according to treatment type in non-small cell lung cancer - future directions for physiotherapy management. *Physiotherapy* 2016; **102**(3): 256-63.

370. Sebio Garcia R, Yanez-Brage MI, Gimenez Moolhuyzen E, Salorio Riobo M, Lista Paz A, Borro Mate JM. Preoperative exercise training prevents functional decline after lung resection surgery: a randomized, single-blind controlled trial. *Clin Rehabil* 2017; **31**(8): 1057-67.

371. Ha D, Choi H, Zell K, et al. Association of impaired heart rate recovery with cardiopulmonary complications after lung cancer resection surgery. *The Journal of thoracic and cardiovascular surgery* 2015; **149**(4): 1168-73.e3.

372. Ha D, Ries AL, Lippman SM, Fuster MM. Effects of curative-intent lung cancer therapy on functional exercise capacity and patient-reported outcomes. *Support Care Cancer* 2020; **28**(10): 4707-20.

373. Hamada K, Irie M, Fujino Y, Hyodo M, Hanagiri T. Prognostic value of preoperative exercise capacity in patients undergoing thoracoscopic lobectomy for non-small cell lung cancer. *Lung Cancer* 2019; **128**: 47-52.

374. Hattori K, Matsuda T, Takagi Y, et al. Preoperative six-minute walk distance is associated with pneumonia after lung resection. *Interactive cardiovascular and thoracic surgery* 2018; **26**(2): 277-83.

375. Henke CC, Cabri J, Fricke L, Pankow W, Kandilakis G, Feyer PC, de Wit M. Strength and endurance training in the treatment of lung cancer patients in stages IIIA/IIIB/IV. *Supportive care in cancer : official journal of the Multinational Association of Supportive Care in Cancer* 2014; **22**(1): 95-101.

376. Huang J, Lai Y, Zhou X, Li S, Su J, Yang M, Che G. Short-term high-intensity rehabilitation in radically treated lung cancer: a three-armed randomized controlled trial. *Journal of thoracic disease* 2017; **9**(7): 1919‐29.

377. Illini O, Valipour A, Gattinger D, Petrovic M, Fabikan H, Hochmair MJ, Zwick RH. Effectiveness of Outpatient Pulmonary Rehabilitation in Patients with Surgically Resected Lung Cancer: A Retrospective Real-World Analysis. *Cancers* 2022; **14**(14).

378. Irie M, Nakanishi R, Yasuda M, Fujino Y, Hamada K, Hyodo M. Risk factors for short-term outcomes after thoracoscopic lobectomy for lung cancer. *The European respiratory journal* 2016; **48**(2): 495-503.

379. Jastrzebski D, Maksymiak M, Kostorz S, et al. Pulmonary rehabilitation in advanced lung cancer patients during chemotherapy. *Advances in experimental medicine and biology* 2015; **861**: 57‐64.

380. Ji W, Kwon H, Lee S, et al. Mobile Health Management Platform-Based Pulmonary Rehabilitation for Patients With Non-Small Cell Lung Cancer: Prospective Clinical Trial. 2019; **7**(6): e12645.

381. Jones LW, Hornsby WE, Goetzinger A, et al. Prognostic significance of functional capacity and exercise behavior in patients with metastatic non-small cell lung cancer. *Lung Cancer* 2012; **76**(2): 248-52.

382. Jonsson M, Hurtig-Wennlof A, Ahlsson A, Vidlund M, Cao Y, Westerdahl E. In-hospital physiotherapy improves physical activity level after lung cancer surgery: a randomized controlled trial. 2019; **105**(4): 434.

383. Kasymjanova G, Correa JA, Kreisman H, et al. Prognostic value of the six-minute walk in advanced non-small cell lung cancer. *Journal of thoracic oncology : official publication of the International Association for the Study of Lung Cancer* 2009; **4**(5): 602-7.

384. Katakami N, Uchino J, Yokoyama T, et al. Anamorelin (ONO-7643) for the treatment of patients with non-small cell lung cancer and cachexia: Results from a randomized, double-blind, placebo-controlled, multicenter study of Japanese patients (ONO-7643-04). *Cancer* 2018; **124**(3): 606-16.

385. Kong S, Shin S, Lee JK, et al. Association between sarcopenia and physical function among preoperative lung cancer patients. *Journal of Personalized Medicine* 2020; **10**(4): 1-11.

386. Lai Y, Su J, Qiu P, Wang M, Zhou K, Tang Y, Che G. Systematic short-term pulmonary rehabilitation before lung cancer lobectomy: a randomized trial. *Interactive cardiovascular and thoracic surgery* 2017; **25**(3): 476‐83.

387. Lai Y, Wang X, Zhou K, Su J, Che G. Impact of one-week preoperative physical training on clinical outcomes of surgical lung cancer patients with limited lung function: A randomized trial. 2019; **7**(20).

388. Li J, Davies M, Ye M, Li Y, Huang L, Li L. Impact of an Animation Education Program on Promoting Compliance With Active Respiratory Rehabilitation in Postsurgical Lung Cancer Patients: a Randomized Clinical Trial. 2019.

389. Li J, Zheng J. Effect of lung rehabilitation training combined with nutritional intervention on patients after thoracoscopic resection of lung cancer. *Oncology Letters* 2024; **27**(3).

390. Liu Z, Qiu T, Pei L, et al. Two-Week Multimodal Prehabilitation Program Improves Perioperative Functional Capability in Patients Undergoing Thoracoscopic Lobectomy for Lung Cancer: a Randomized Controlled Trial. 2019.

391. Marjanski T, Wnuk D, Bosakowski D, Szmuda T, Sawicka W, Rzyman W. Patients who do not reach a distance of 500 m during the 6-min walk test have an increased risk of postoperative complications and prolonged hospital stay after lobectomy. *European journal of cardio-thoracic surgery : official journal of the European Association for Cardio-thoracic Surgery* 2015; **47**(5): e213-9.

392. Maruyama R, Tanaka J, Kitagawa D, et al. Physical assessment immediately after lobectomy via miniposterolateral thoracotomy assisted by videothoracoscopy for non-small cell lung cancer. *Surgery today* 2011; **41**(7): 908-13.

393. Menna C, Poggi C, Andreetti C, et al. Does the length of uniportal video-assisted thoracoscopic lobectomy affect postoperative pain? Results of a randomized controlled trial. 2020.

394. Miller KL, Kocak Z, Kahn D, et al. Preliminary report of the 6-minute walk test as a predictor of radiation-induced pulmonary toxicity. *International journal of radiation oncology, biology, physics* 2005; **62**(4): 1009-13.

395. Mohan A, Singh P, Kumar S, Mohan C, Pathak AK, Pandey RM, Guleria R. Effect of change in symptoms, respiratory status, nutritional profile and quality of life on response to treatment for advanced non-small cell lung cancer. *Asian Pacific journal of cancer prevention : APJCP* 2008; **9**(4): 557-62.

396. Mohan A, Poulose R, Gupta T, et al. Impact of chemotherapy on symptom profile, oxidant-antioxidant balance and nutritional status in non-small cell Lung Cancer. *Lung India* 2017; **34**(4): 336-40.

397. Morano MT, Mesquita R, Da Silva GP, et al. Comparison of the effects of pulmonary rehabilitation with chest physical therapy on the levels of fibrinogen and albumin in patients with lung cancer awaiting lung resection: a randomized clinical trial. *BMC pulmonary medicine* 2014; **14**: 121.

398. Mujovic N, Mujovic N, Subotic D, et al. Preoperative pulmonary rehabilitation in patients with non-small cell lung cancer and chronic obstructive pulmonary disease. *Archives of Medical Science* 2014; **10**(1): 68-75.

399. Mujovic N, Mujovic N, Subotic D, et al. Influence of pulmonary rehabilitation on lung function changes after the lung resection for primary lung cancer in patients with chronic obstructive pulmonary disease. *Aging and Disease* 2015; **6**(6): 466-77.

400. Nakagawa T, Tomioka Y, Toyazaki T, Gotoh M. Association between values of preoperative 6-min walk test and surgical outcomes in lung cancer patients with decreased predicted postoperative pulmonary function. *Scand J Med Sci Sports* 2018; **66**(4): 220-4.

401. Nomori H, Horio H, Naruke T, Suemasu K. What is the advantage of a thoracoscopic lobectomy over a limited thoracotomy procedure for lung cancer surgery? *The Annals of thoracic surgery* 2001; **72**(3): 879-84.

402. Nomori H, Ohtsuka T, Horio H, Naruke T, Suemasu K. Difference in the impairment of vital capacity and 6-minute walking after a lobectomy performed by thoracoscopic surgery, an anterior limited thoracotomy, an anteroaxillary thoracotomy, and a posterolateral thoracotomy. *Surgery today* 2003; **33**(1): 7-12.

403. Parsons JA, Johnston MR, Slutsky AS. Predicting length of stay out of hospital following lung resection using preoperative health status measures. *Qual Life Res* 2003; **12**(6): 645-54.

404. Quist M, Adamsen L, Rorth M, Laursen JH, Christensen KB, Langer SW. The Impact of a Multidimensional Exercise Intervention on Physical and Functional Capacity, Anxiety, and Depression in Patients With Advanced-Stage Lung Cancer Undergoing Chemotherapy. *Integrative cancer therapies* 2015; **14**(4): 341-9.

405. Rehman M, Ahmad U, Waseem M, Ali B, Tariq MI. Effects of Exercise Training in Patients with Lung Cancer during Chemotherapy Treatment. *Malaysian Journal of Medical Sciences* 2023; **30**(2): 141-52.

406. Rick O, Metz T, Eberlein M, Schirren J, Bölükbas S. The Six-Minute-Walk Test in assessing respiratory function after tumor surgery of the lung: A cohort study. *Journal of Thoracic Disease* 2014; **6**(5): 421-8.

407. Riesenberg H, Lubbe AS. In-patient rehabilitation of lung cancer patients--a prospective study. *Supportive care in cancer : official journal of the Multinational Association of Supportive Care in Cancer* 2010; **18**(7): 877-82.

408. Rosero ID, Ramírez-Vélez R, Martínez-Velilla N, Cedeño-Veloz BA, Morilla I, Izquierdo M. Effects of a multicomponent exercise program in older adults with non-small-cell lung cancer during adjuvant/palliative treatment: An intervention study. 2020; **9**(3).

409. Rutkowska A, Jastrzebski D, Rutkowski S, et al. Exercise Training in Patients With Non-Small Cell Lung Cancer During In-Hospital Chemotherapy Treatment: A RANDOMIZED CONTROLLED TRIAL. 2019; **39**(2): 127.

410. Saad IA, Botega NJ, Toro IF. Evaluation of quality of life of patients submitted to pulmonary resection due to neoplasia. *Jornal brasileiro de pneumologia : publicacao oficial da Sociedade Brasileira de Pneumologia e Tisilogia* 2006; **32**(1): 10-5.

411. Saad IA, Botega NJ, Toro IF. Predictors of quality-of-life improvement following pulmonary resection due to lung cancer. *Sao Paulo medical journal = Revista paulista de medicina* 2007; **125**(1): 46-9.

412. Saito H, Shiraishi A, Nomori H, et al. Impact of age on the recovery of six-minute walking distance after lung cancer surgery: a retrospective cohort study. 2020; **68**(2): 150.

413. Shallwani SM, Simmonds MJ, Kasymjanova G, Spahija J. Quality of life, symptom status and physical performance in patients with advanced non-small cell lung cancer undergoing chemotherapy: an exploratory analysis of secondary data. *Lung Cancer* 2016; **99**: 69-75.

414. Sterzi S, Cesario A, Cusumano G, et al. Post-operative rehabilitation for surgically resected non-small cell lung cancer patients: serial pulmonary functional analysis. *Journal of rehabilitation medicine* 2013; **45**(9): 911-5.

415. Stigt JA, Uil SM, van Riesen SJ, Simons FJ, Denekamp M, Shahin GM, Groen HJ. A randomized controlled trial of postthoracotomy pulmonary rehabilitation in patients with resectable lung cancer. *J Thorac Oncol* 2013; **8**(2): 214-21.

416. Suzuki H, Sekine Y, Yoshida S, et al. Efficacy of perioperative administration of long-acting bronchodilator on postoperative pulmonary function and quality of life in lung cancer patients with chronic obstructive pulmonary disease. Preliminary results of a randomized control study. *Surgery today* 2010; **40**(10): 923-30.

417. Takemura N, Cheung DST, Fong DYT, et al. Relationship of subjective and objective sleep measures with physical performance in advanced-stage lung cancer patients. *Sci Rep* 2021; **11**(1): 17208.

418. Tao W, Huang J, Jin Y, Peng K, Zhou J. Effect of Pulmonary Rehabilitation Exercise on Lung Volume and Respiratory Muscle Recovery in Lung Cancer Patients Undergoing Lobectomy. 2023.

419. Timmerman JGJ, Dekker-van Weering MGHM, Wouters MWJMM, Stuiver MMM, e Kanter WW, Vollenbroek-Hutten MMRM. Physical behavior and associations with health outcomes in operable NSCLC patients: A prospective study. *Lung Cancer* 2018; **119**: 91-8.

420. Titz C, Hummler S, Schmidt ME, Thomas M, Steins M, Wiskemann J. Exercise behavior and physical fitness in patients with advanced lung cancer. *Supportive care in cancer* 2018; **26**(8): 2725‐36.

421. Torre-Bouscoulet L, Muñoz-Montaño WR, Martínez-Briseño D, et al. Abnormal pulmonary function tests predict the development of radiation-induced pneumonitis in advanced non-small cell lung Cancer. *Respiratory Research* 2018; **19**(1).

422. Torre-Bouscoulet L, Arroyo-Hernández M, Martínez-Briseño D, et al. Longitudinal Evaluation of Lung Function in Patients With Advanced Non-Small Cell Lung Cancer Treated With Concurrent Chemoradiation Therapy. *International Journal of Radiation Oncology Biology Physics* 2018; **101**(4): 910-8.

423. Ueda K, Haruki T, Murakami J, Tanaka T, Hayashi M, Hamano K. No Drain After Thoracoscopic Major Lung Resection for Cancer Helps Preserve the Physical Function. 2019; **108**(2): 399.

424. Ugalde P, Miro S, Provencher S, et al. Ipsilateral diaphragmatic motion and lung function in long-term pneumonectomy patients. *The Annals of thoracic surgery* 2008; **86**(6): 1745-51; discussion 51-2.

425. Vagvolgyi A, Rozgonyi Z, Kerti M, Agathou G, Vadasz P, Varga J. Effectiveness of pulmonary rehabilitation and correlations in between functional parameters, extent of thoracic surgery and severity of post-operative complications: Randomized clinical trial. *Journal of Thoracic Disease* 2018; **10**(6): 3519-31.

426. van der Weijst L, Bultijnck R, Van Damme A, Huybrechts V, van Eijkeren M, Lievens Y. Patient-reported outcomes and functional exercise capacity in a real-life setting in non-small cell lung cancer patients undergoing stereotactic body radiotherapy: the Lung PLUS study. *Frontiers in Oncology* 2023; **13**((van der Weijst L.; Bultijnck R., renee.bultijnck@ugent.be; Huybrechts V.; van Eijkeren M.; Lievens Y.) Department of Human Structure and Repair, Ghent University, Ghent, Belgium(van der Weijst L.; Bultijnck R., renee.bultijnck@ugent.be; Van Damme A.; Huy).

427. Wang LY, Wu HD, Chen KY, Hsieh CH, Lai CC. Impact of age on functional exercise correlates in patients with advanced lung cancer. *OncoTargets and Therapy* 2013; **6**: 1277-83.

428. Tang YJ, Wang CY, Wang CD, Dong YZ. Clinical study of simultaneous lung volume reduction surgery during resection of pulmonary or esophageal neoplasms. *Chinese medical journal* 2009; **122**(24): 2973-6.

429. Yang M, Zhong JD, Zhang JE, Huang XX, Li CZ, Hong ZX, Zhang SW. Effect of the self-efficacy-enhancing active cycle of breathing technique on lung cancer patients with lung resection: A quasi-experimental trial. *European journal of oncology nursing : the official journal of European Oncology Nursing Society* 2018; **34**: 1-7.

430. Zarogoulidis P, Kerenidi T, Huang H, et al. Six minute walking test and carbon monoxide diffusing capacity for non-small cell lung cancer: Easy performed tests in every day practice. *Journal of Thoracic Disease* 2012; **4**(6): 569-76.

431. Zhang W, Cong X, Zhang L, et al. Effects of thoracic nerve block on perioperative lung injury, immune function, and recovery after thoracic surgery. 2020; **10**(3).

432. Armstrong GT, Joshi VM, Zhu L, et al. Increased tricuspid regurgitant jet velocity by Doppler echocardiography in adult survivors of childhood cancer: a report from the St Jude Lifetime Cohort Study. *Journal of clinical oncology : official journal of the American Society of Clinical Oncology* 2013; **31**(6): 774-81.

433. Bolli R, Perin EC, Willerson JT, et al. Allogeneic Mesenchymal Cell Therapy in Anthracycline-Induced Cardiomyopathy Heart Failure Patients: The CCTRN SENECA Trial. *JACC: CardioOncology* 2020; **2**(4): 581-95.

434. Brown JC, Yung RL, Gobbie-Hurder A, et al. Randomized trial of a clinic-based weight loss intervention in cancer survivors. 2018; **12**(2): 186‐95.

435. Chemaitilly W, Li Z, Huang S, et al. Anterior hypopituitarism in adult survivors of childhood cancers treated with cranial radiotherapy: a report from the St Jude Lifetime Cohort study. 2015; **33**(5): 492.

436. Devenney K, Murphy N, Ryan R, et al. Implementing a physiotherapy led cancer exercise programme in a National Cancer Centre: the FIXCAS study. *Physiotherapy* 2023; **120**: 27-35.

437. Gagnon B, Murphy J, Eades M, et al. A prospective evaluation of an interdisciplinary nutrition-rehabilitation program for patients with advanced cancer. *Current Oncology* 2013; **20**(6): 310-8.

438. Glare P, Jongs W, Zafiropoulos B. Establishing a cancer nutrition rehabilitation program (CNRP) for ambulatory patients attending an Australian cancer center. *Supportive care in cancer : official journal of the Multinational Association of Supportive Care in Cancer* 2011; **19**(4): 445-54.

439. Green DM, Zhu L, Wang M, et al. Pulmonary Function after Treatment for Childhood Cancer. A Report from the St. Jude Lifetime Cohort Study (SJLIFE). *Annals of the American Thoracic Society* 2016; **13**(9): 1575-85.

440. Hadzibegovic S, Porthun J, Lena A, et al. Hand grip strength in patients with advanced cancer: A prospective study. *Journal of Cachexia, Sarcopenia and Muscle* 2023; **14**(4): 1682-94.

441. Hayakawa J, Miyamura D, Kimura SI, et al. Negative impact of chronic graft-versus-host disease and glucocorticoid on the recovery of physical function after allogeneic hematopoietic stem cell transplantation. 2019; **54**(7): 994.

442. Hui D, Xu A, Frisbee-Hume S, Chisholm G, Morgado M, Reddy S, Bruera E. Effects of prophylactic subcutaneous fentanyl on exercise-induced breakthrough dyspnea in cancer patients: a preliminary double-blind, randomized, controlled trial. *Journal of pain and symptom management* 2014; **47**(2): 209-17.

443. Hutchison NA, Deval N, Rabusch S, Rich H, Kelley T, Flinn N, Banerji N. Physical Therapy-Based Exercise Protocol for Cancer Patients: Evaluating Outcomes for Cardiopulmonary Performance and Cancer-Related Fatigue. 2019; **11**(11): 1178.

444. Irwin ML, Cartmel B, Harrigan M, et al. Effect of the LIVESTRONG at the YMCA exercise program on physical activity, fitness, quality of life, and fatigue in cancer survivors. *Cancer* 2017; **123**(7): 1249-58.

445. Kimberg CI, Klosky JL, Zhang N, et al. Predictors of health care utilization in adult survivors of childhood cancer exposed to central nervous system-directed therapy. *Cancer* 2015; **121**(5): 774-82.

446. Kirkham AA, Klika RJ, Ballard T, Downey P, Campbell KL. Effective Translation of Research to Practice: Hospital-Based Rehabilitation Program Improves Health-Related Physical Fitness and Quality of Life of Cancer Survivors. *J Natl Compr Canc Netw* 2016; **14**(12): 1555-62.

447. Leach HJ, Covington KR, Pergolotti M, Sharp J, Maynard B, Eagan J, Beasley J. Translating Research to Practice Using a Team-Based Approach to Cancer Rehabilitation: A Physical Therapy and Exercise-Based Cancer Rehabilitation Program Reduces Fatigue and Improves Aerobic Capacity. 2018; **36**(4): 206.

448. St Lezin E, Karafin MS, Bruhn R, et al. Therapeutic impact of red blood cell transfusion on anemic outpatients: the RETRO study. 2019; **59**(6): 1934.

449. Al-Mozaini MA, Islam M, Noman ASM, Karim ATMR, Farhat WA, Yeger H, Islam SS. Decline in Respiratory Functions in Hospitalized SARS-CoV-2 Infected Cancer Patients Following Cytotoxic Chemotherapy—An Additional Risk for Post-chemotherapy Complications. *Frontiers in Medicine* 2022; **9**((Al-Mozaini M.A.) Department of Infectious Disease and Immunity, King Faisal Specialist Hospital and Research Centre, Riyadh, Saudi Arabia(Islam M.; Karim A.T.M.R.) Department of Medicine, Parkview Hospital, Chittagong, Bangladesh(Noman A.S.M.) Department).

450. Madeddu C, Dessi M, Panzone F, et al. Randomized phase III clinical trial of a combined treatment with carnitine + celecoxib +/- megestrol acetate for patients with cancer-related anorexia/cachexia syndrome. *Clinical nutrition (Edinburgh, Scotland)* 2012; **31**(2): 176-82.

451. Mangia AS, Coqueiro NLO, Azevedo FC, et al. What clinical, functional, and psychological factors before treatment are predictors of poor quality of life in cancer patients at the end of chemotherapy? *Revista da Associacao Medica Brasileira (1992)* 2017; **63**(11): 978-87.

452. Marker RJ, Wechsler S, Leach HJ. Cancer-Related Fatigue Is Associated with Objective Measures of Physical Function before and after a Clinical Exercise Program: A Retrospective Analysis. *Rehabilitation Oncology* 2024; **42**(1): 31-8.

453. McCrary JM, Goldstein D, Wyld D, Henderson R, Lewis CR, Park SB. Mobility in survivors with chemotherapy-induced peripheral neuropathy and utility of the 6-min walk test. 2019; **13**(4): 495.

454. Mikkelsen MK, Lund CM, Vinther A, et al. Effects of a 12-Week Multimodal Exercise Intervention Among Older Patients with Advanced Cancer: Results from a Randomized Controlled Trial. *Oncologist* 2022; **27**(1): 67-78.

455. Morishita S, Kaida K, Setogawa K, et al. Safety and feasibility of physical therapy in cytopenic patients during allogeneic haematopoietic stem cell transplantation. *European journal of cancer care* 2013; **22**(3): 289-99.

456. Morris GS, Gallagher GH, Baxter MF, Brueilly KE, Scheetz JS, Ahmed MM, Shannon VR. Pulmonary rehabilitation improves functional status in oncology patients. *Archives of physical medicine and rehabilitation* 2009; **90**(5): 837-41.

457. Mulrooney DA, Armstrong GT, Huang S, et al. Cardiac Outcomes in Adult Survivors of Childhood Cancer Exposed to Cardiotoxic Therapy: A Cross-sectional Study. *Annals of internal medicine* 2016; **164**(2): 93-101.

458. Ngo-Huang A, Yadav R, Bansal S, et al. An Exploratory Study on Physical Function in Stem Cell Transplant Patients Undergoing Corticosteroid Treatment for Acute Graft-Versus-Host-Disease. *Am J Phys Med Rehabil* 2021; **100**(4): 402-6.

459. Noh S, Bertini C, Mira-Avendano I, et al. Interstitial lung abnormalities after hospitalization for COVID-19 in patients with cancer: A prospective cohort study. *Cancer Medicine* 2023; **12**(17): 17753-65.

460. Oviatt PL, Stather DR, Michaud G, Maceachern P, Tremblay A. Exercise capacity, lung function, and quality of life after interventional bronchoscopy. *Journal of thoracic oncology : official publication of the International Association for the Study of Lung Cancer* 2011; **6**(1): 38-42.

461. Parmar MP, Vanderbyl BL, Kanbalian M, Windholz TY, Tran AT, Jagoe RT. A multidisciplinary rehabilitation programme for cancer cachexia improves quality of life. 2017; **7**(4): 441-9.

462. Potiaumpai M, Cutrono S, Medina T, et al. Multidirectional Walking in Hematopoietic Stem Cell Transplant Patients. *Med Sci Sports Exerc* 2021; **53**(2): 258-66.

463. Rossi A, Garber CE, Ortiz M, Shankar V, Goldberg GL, Nevadunsky NS. Feasibility of a physical activity intervention for obese, socioculturally diverse endometrial cancer survivors. *Gynecologic oncology* 2016; **142**(2): 304-10.

464. Santa Mina D, Au D, Brunet J, et al. Effects of the community-based Wellspring Cancer Exercise Program on functional and psychosocial outcomes in cancer survivors. *Curr Oncol* 2017; **24**(5): 284-94.

465. Schuler MK, Hentschel L, Kisel W, et al. Impact of Different Exercise Programs on Severe Fatigue in Patients Undergoing Anticancer Treatment-A Randomized Controlled Trial. *Journal of pain and symptom management* 2017; **53**(1): 57-66.

466. Schumacher AN, Shackelford DYK, Brown JM, Hayward R. Validation of the 6-min Walk Test for Predicting Peak V˙O2 in Cancer Survivors. 2019; **51**(2): 271.

467. Slater ME, Steinberger J, Ross JA, et al. Physical Activity, Fitness, and Cardiometabolic Risk Factors in Adult Survivors of Childhood Cancer with a History of Hematopoietic Cell Transplantation. *Biology of blood and marrow transplantation : journal of the American Society for Blood and Marrow Transplantation* 2015; **21**(7): 1278-83.

468. Smith WA, Li Z, Loftin M, Carlyle BE, Hudson MM, Robison LL, Ness KK. Measured versus self-reported physical function in adult survivors of childhood cancer. *Medicine and science in sports and exercise* 2014; **46**(2): 211-8.

469. Sturm I, Baak J, Storek B, Traore A, Thuss-Patience P. Effect of dance on cancer-related fatigue and quality of life. *Supportive care in cancer : official journal of the Multinational Association of Supportive Care in Cancer* 2014; **22**(8): 2241-9.

470. Swenson KK, Nissen MJ, Knippenberg K, et al. Cancer rehabilitation: outcome evaluation of a strengthening and conditioning program. *Cancer nursing* 2014; **37**(3): 162-9.

471. Tran H, Lin C, Yu F, Frederick A, Mieras M, Baccaglini L. A multicenter study on the relative effectiveness of a 12-week physical training program for adults with an oncologic diagnosis. *Supportive care in cancer : official journal of the Multinational Association of Supportive Care in Cancer* 2016; **24**(9): 3705-13.

472. Uster A, Ruehlin M, Mey S, et al. Effects of nutrition and physical exercise intervention in palliative cancer patients: A randomized controlled trial. *Clinical Nutrition* 2018; **37**(4): 1202-9.

473. Coleman EA, Coon SK, Kennedy RL, Lockhart KD, Stewart CB, Anaissie EJ, Barlogie B. Effects of exercise in combination with epoetin alfa during high-dose chemotherapy and autologous peripheral blood stem cell transplantation for multiple myeloma. *Oncology nursing forum* 2008; **35**(3): E53-61.

474. Larsen RF, Jarden M, Minet LR, Frølund UC, Möller S, Abildgaard N. Physical function in patients newly diagnosed with multiple myeloma; a Danish cohort study. *BMC cancer* 2020; **20**(1): 169.

475. An N-H, Parker NH, Bruera E, et al. Home-Based Exercise Prehabilitation During Preoperative Treatment for Pancreatic Cancer Is Associated With Improvement in Physical Function and Quality of Life. 2019; **18**: 1.

476. Birgitte Stene G, Balstad TR, Leer ASM, et al. Deterioration in muscle mass and physical function differs according to weight loss history in cancer cachexia. 2019; **11**(12).

477. Wiedenmann B, Malfertheiner P, Friess H, et al. A multicenter, phase II study of infliximab plus gemcitabine in pancreatic cancer cachexia. *The journal of supportive oncology* 2008; **6**(1): 18-25.

478. Alibhai SM, Breunis H, Timilshina N, et al. Impact of androgen-deprivation therapy on physical function and quality of life in men with nonmetastatic prostate cancer. *Journal of clinical oncology : official journal of the American Society of Clinical Oncology* 2010; **28**(34): 5038-45.

479. Alibhai SM, Breunis H, Timilshina N, et al. Long-term impact of androgen-deprivation therapy on physical function and quality of life. *Cancer* 2015; **121**(14): 2350-7.

480. Au D, Matthew AG, Lopez P, et al. Prehabilitation and acute postoperative physical activity in patients undergoing radical prostatectomy: a secondary analysis from an RCT. 2019; **5**(1).

481. Mareschal J, Weber K, Rigoli P, et al. The ADAPP trial: a two-year longitudinal multidisciplinary intervention study for prostate cancer frail patients on androgen deprivation associated to curative radiotherapy. *Acta oncologica (Stockholm, Sweden)* 2017; **56**(4): 569-74.

482. O'Neill RF, Haseen F, Murray LJ, O'Sullivan JM, Cantwell MM. A randomised controlled trial to evaluate the efficacy of a 6-month dietary and physical activity intervention for patients receiving androgen deprivation therapy for prostate cancer. *Journal of cancer survivorship : research and practice* 2015; **9**(3): 431-40.

483. Santa Mina D, Hilton WJ, Matthew AG, et al. Prehabilitation for radical prostatectomy: A multicentre randomized controlled trial. *Surgical Oncology* 2018; **27**(2): 289-98.

484. Villumsen BR, Jorgensen MG, Frystyk J, Hordam B, Borre M. Home-based ‘exergaming’ was safe and significantly improved 6-min walking distance in patients with prostate cancer: a single-blinded randomised controlled trial. 2019.

485. Bishop MW, Ness KK, Chenghong L, et al. Cumulative Burden of Chronic Health Conditions in Adult Survivors of Osteosarcoma and Ewing Sarcoma: A Report from the St. Jude Lifetime Cohort Study. 2020; **29**(8): 1627.

486. Morri M, Forni C, Ruisi R, Giamboi T, Giacomella F, Donati DM, Benedetti MG. Postoperative function recovery in patients with endoprosthetic knee replacement for bone tumour: an observational study. 2018; **19**(1): 353.

487. van Egmond-van Dam JC, Bekkering WP, Bramer JAM, Beishuizen A, Fiocco M, Dijkstra PDS. Functional outcome after surgery in patients with bone sarcoma around the knee; results from a long-term prospective study. *Journal of surgical oncology* 2017; **115**(8): 1028-32.

488. Blackwell JEM, Doleman B, Boereboom CL, et al. High-intensity interval training produces a significant improvement in fitness in less than 31 days before surgery for urological cancer: a randomised control trial. 2020.

489. Ruden E, Reardon DA, Coan AD, et al. Exercise behavior, functional capacity, and survival in adults with malignant recurrent glioma. *Journal of clinical oncology : official journal of the American Society of Clinical Oncology* 2011; **29**(21): 2918-23.

490. Alizadeh AM, Isanejad A, Sadighi S, Mardani M, Kalaghchi B, Hassan ZM. High-intensity interval training can modulate the systemic inflammation and HSP70 in the breast cancer: a randomized control trial. 2019; **145**(10): 2583‐93.

491. Antunes P, Joaquim A, Sampaio F, et al. Effects of exercise training on cardiac toxicity markers in women with breast cancer undergoing chemotherapy with anthracyclines: a randomized controlled trial. 2023; **30**(9): 844‐55.

492. Brown NI, Pekmezi DW, Oster RA, et al. Relationships between Obesity, Exercise Preferences, and Related Social Cognitive Theory Variables among Breast Cancer Survivors. *Nutrients* 2023; **15**(5).

493. Casla S, Lopez-Tarruella S, Jerez Y, et al. Supervised physical exercise improves VO2max, quality of life, and health in early stage breast cancer patients: a randomized controlled trial. 2015; **153**(2): 371‐82.

494. Chap L, Shpiner R, Levine M, Norton L, Lill M, Glaspy J. Pulmonary toxicity of high-dose chemotherapy for breast cancer: a non-invasive approach to diagnosis and treatment. *Bone marrow transplantation* 1997; **20**(12): 1063-7.

495. Chung WP, Yang HL, Hsu YT, et al. Real-time exercise reduces impaired cardiac function in breast cancer patients undergoing chemotherapy: a randomized controlled trial. *Annals of physical and rehabilitation medicine* 2021: 101485‐.

496. Delrieu L, Pialoux V, Perol O, et al. Feasibility and Health Benefits of an Individualized Physical Activity Intervention in Women With Metastatic Breast Cancer: intervention Study. 2020; **8**(1): e12306‐.

497. Dieli-Conwright CM, Courneya KS, Demark-Wahnefried W, et al. Aerobic and resistance exercise improves physical fitness, bone health, and quality of life in overweight and obese breast cancer survivors: a randomized controlled trial. 2018; **20**(1): 124.

498. Do J, Cho Y, Jeon J. Effects of a 4-week multimodal rehabilitation program on quality of life, cardiopulmonary function, and fatigue in breast cancer patients. 2015; **18**(1): 87.

499. Elme A, Utriainen M, Kellokumpu-Lehtinen P, et al. Obesity and physical inactivity are related to impaired physical health of breast cancer survivors. *Anticancer Res* 2013; **33**(4): 1595-602.

500. Farajivafa V, Khosravi N, Rezaee N, Koosha M, Haghighat S. Effectiveness of home-based exercise in breast cancer survivors: a randomized clinical trial. *BMC Sports Science, Medicine and Rehabilitation* 2023; **15**(1).

501. França-Lara ÉG, Weber SH, Pinho RA, Casali-da-Rocha JC, Elifio-Esposito S. A remote, fully oriented personalized program of physical exercise for women in follow-up after breast cancer treatment improves body composition and physical fitness. *Sports Medicine and Health Science* 2023; **5**(2): 128-36.

502. Giallauria F, Maresca L, Vitelli A, et al. Exercise training improves heart rate recovery in women with breast cancer. *Springerplus* 2015; **4**: 388.

503. Groarke JD, Mahmood SS, Payne D, et al. Case-control study of heart rate abnormalities across the breast cancer survivorship continuum. 2019; **8**(1): 447.

504. Gustavsson A, Bendahl PO, Cwikiel M, Eskilsson J, Thapper KL, Pahlm O. No serious late cardiac effects after adjuvant radiotherapy following mastectomy in premenopausal women with early breast cancer. *International journal of radiation oncology, biology, physics* 1999; **43**(4): 745-54.

505. Gyenes G, Fornander T, Carlens P, Rutqvist LE. Morbidity of ischemic heart disease in early breast cancer 15-20 years after adjuvant radiotherapy. *International journal of radiation oncology, biology, physics* 1994; **28**(5): 1235-41.

506. Heim ME, v d Malsburg ML, Niklas A. Randomized controlled trial of a structured training program in breast cancer patients with tumor-related chronic fatigue. 2007; **30**(8-9): 429.

507. Joaquim A, Amarelo A, Antunes P, et al. Effects of a Physical Exercise Program on Quality of Life and Physical Fitness of Breast Cancer Survivors: the MAMA_MOVE Gaia After Treatment Trial. 2023: 1‐24.

508. Jones LM, Stoner L, Baldi JC, McLaren B. Circuit resistance training and cardiovascular health in breast cancer survivors. *European journal of cancer care* 2020; **29**(4): e13231.

509. Lakoski SG, Barlow CE, Koelwyn GJ, et al. The influence of adjuvant therapy on cardiorespiratory fitness in early-stage breast cancer seven years after diagnosis: the Cooper Center Longitudinal Study. *Breast cancer research and treatment* 2013; **138**(3): 909-16.

510. Ligibel JA, Giobbie-Hurder A, Shockro L, et al. Randomized trial of a physical activity intervention in women with metastatic breast cancer. *Cancer* 2016; **122**(8): 1169-77.

511. Mijwel S, Backman M, Bolam KA, et al. Highly favorable physiological responses to concurrent resistance and high-intensity interval training during chemotherapy: the OptiTrain breast cancer trial. *Breast cancer research and treatment* 2018; **169**(1): 93‐103.

512. Mijwel S, Jervaeus A, Bolam KA, Norrbom J, Bergh J, Rundqvist H, Wengstrom Y. High-intensity exercise during chemotherapy induces beneficial effects 12 months into breast cancer survivorship. 2019; **13**(2): 244.

513. Murtezani A, Ibraimi Z, Bakalli A, Krasniqi SH, Devolli Disha E, Kurtishi I. The effect of aerobic exercise on quality of life among breast cancer survivors: a randomized controlled trial. *Pain practice* 2014; **14**: 28.

514. Musanti R. A study of exercise modality and physical self-esteem in breast cancer survivors. *Medicine and science in sports and exercise* 2012; **44**(2): 352-61.

515. Mutrie N, Campbell AM, Whyte F, et al. Benefits of supervised group exercise programme for women being treated for early stage breast cancer: pragmatic randomised controlled trial. *BMJ* 2007; **334**(7592): 517.

516. Natalucci V, Marini CF, Flori M, et al. Effects of a home-based lifestyle intervention program on cardiometabolic health in breast cancer survivors during the covid-19 lockdown. *Journal of Clinical Medicine* 2021; **10**(12).

517. Navarro-Sanz A, Espejo-Reina A, Cerezo-Guzman MV, Fernandez-Ortega JF, Meza-Leiva H, Conejo-Tirado I, Santiago-Sanchez C. Synchronized Pedaling with Martial Arts Improves Quality of Life of Women with Breast Cancer. 2018; **39**(13): 978.

518. Okumatsu K, Tsujimoto T, Wakaba K, et al. Effects of a combined exercise plus diet program on cardiorespiratory fitness of breast cancer patients. 2019; **26**(1): 65‐71.

519. Penttinen HM, Saarto T, Kellokumpu-Lehtinen P, et al. Quality of life and physical performance and activity of breast cancer patients after adjuvant treatments. *Psycho-oncology* 2011; **20**(11): 1211-20.

520. Reis AD, Lima LS, Barros E, et al. Impact of home-based aerobic training combined with food orientation on food consumption, daily physical activity and cardiorespiratory fitness among breast cancer survivors: six-month clinical trial. 2021; **139**(3): 259‐68.

521. Reis AD, Pereira P, Filha J, et al. Effect of Combined Training on Body Image, Body Composition and Functional Capacity in Patients with Breast Cancer: controlled Clinical Trial. 2023; **45**(5): 242‐52.

522. Rogers LQ, Courneya KS, Anton PM, et al. Effects of the BEAT Cancer physical activity behavior change intervention on physical activity, aerobic fitness, and quality of life in breast cancer survivors: a multicenter randomized controlled trial. *Breast cancer research and treatment* 2015; **149**(1): 109-19.

523. Romero-Elías M, Álvarez-Bustos A, Cantos B, et al. C-Reactive Protein Is Associated with Physical Fitness in Breast Cancer Survivors. *Journal of Clinical Medicine* 2023; **12**(1).

524. Saarto T, Penttinen HM, Sievanen H, et al. Effectiveness of a 12-month exercise program on physical performance and quality of life of breast cancer survivors. *Anticancer research* 2012; **32**(9): 3875-84.

525. Samhan AF, Ahmed AS, Mahmoud WS, Abdelhalim NM. Effects of High-Intensity Interval Training on Cardiorespiratory Fitness, Body Composition, and Quality of Life in Overweight and Obese Survivors of Breast Cancer. *Rehabilitation Oncology* 2021; **39**(4): 168-74.

526. Schmidt T, Weisser B, Durkop J, Jonat W, Van Mackelenbergh M, Rocken C, Mundhenke C. Comparing Endurance and Resistance Training with Standard Care during Chemotherapy for Patients with Primary Breast Cancer. *Anticancer research* 2015; **35**(10): 5623-9.

527. Schwartz AL. Daily fatigue patterns and effect of exercise in women with breast cancer. *Cancer practice* 2000; **8**(1): 16-24.

528. Scott E, Daley AJ, Doll H, et al. Effects of an exercise and hypocaloric healthy eating program on biomarkers associated with long-term prognosis after early-stage breast cancer: a randomized controlled trial. 2013; **24**(1): 181.

529. Segal R, Evans W, Johnson D, et al. Structured exercise improves physical functioning in women with stages I and II breast cancer: results of a randomized controlled trial. 2001; **19**(3): 657.

530. Soriano-Maldonado A, Diez-Fernandez DM, Esteban-Simon A, et al. Effects of a 12-week supervised resistance training program, combined with home-based physical activity, on physical fitness and quality of life in female breast cancer survivors: the EFICAN randomized controlled trial. 2023; **17**(5): 1371‐85.

531. Stalsberg R, Bertheussen GF, Børset H, et al. Do Breast Cancer Patients Manage to Participate in an Outdoor, Tailored, Physical Activity Program during Adjuvant Breast Cancer Treatment, Independent of Health and Socio-Demographic Characteristics? *Journal of Clinical Medicine* 2022; **11**(3).

532. van Waart H, Stuiver MM, van Harten WH, et al. Effect of Low-Intensity Physical Activity and Moderate- to High-Intensity Physical Exercise During Adjuvant Chemotherapy on Physical Fitness, Fatigue, and Chemotherapy Completion Rates: results of the PACES Randomized Clinical Trial. 2015; **33**(17): 1918‐27.

533. Vardar Yagli N, Sener G, Arikan H, et al. Do yoga and aerobic exercise training have impact on functional capacity, fatigue, peripheral muscle strength, and quality of life in breast cancer survivors? *Integrative cancer therapies* 2015; **14**(2): 125-32.

534. Schwartz AL, Winters-Stone K. Effects of a 12-month randomized controlled trial of aerobic or resistance exercise during and following cancer treatment in women. 2009; **37**(3): 62.

535. Schneider J, Schlüter K, Sprave T, Wiskemann J, Rosenberger F. Exercise intensity prescription in cancer survivors: ventilatory and lactate thresholds are useful submaximal alternatives to VO(2peak). *Support Care Cancer* 2020; **28**(11): 5521-8.

536. Alexander D, Allardice GM, Moug SJ, Morrison DS. A retrospective cohort study of the influence of lifestyle factors on the survival of patients undergoing surgery for colorectal cancer. *Colorectal disease : the official journal of the Association of Coloproctology of Great Britain and Ireland The Indian journal of chest diseases & allied sciences* 2017; **19**(6): 544-50.

537. Cuijpers ACM, Heldens A, Bours MJL, van Meeteren NLU, Stassen LPS, Lubbers T, Bongers BC. Relation between preoperative aerobic fitness estimated by steep ramp test performance and postoperative morbidity in colorectal cancer surgery: prospective observational study. *Br J Surg* 2022; **109**(2): 155-9.

538. Heldens A, Bongers BC, Lenssen AF, Stassen LPS, Buhre WF, van Meeteren NLU. The association between performance parameters of physical fitness and postoperative outcomes in patients undergoing colorectal surgery: An evaluation of care data. *European journal of surgical oncology : the journal of the European Society of Surgical Oncology and the British Association of Surgical Oncology* 2017; **43**(11): 2084-92.

539. Heldens AFJM, Bongers BC, De Vos-Geelen J, Minis-Rutten IJ, Stassen LP, Buhre WF, Van Meeteren NL. Physical Fitness and Skeletal Muscle Mass during Neoadjuvant Chemoradiotherapy in Patients with Locally Advanced Rectal Cancer: An Observational Study. *Rehabilitation Oncology* 2021; **39**(4): E73-E82.

540. Kindred MM, Pinto BM, Dunsiger SI. Predictors of sedentary behavior among colorectal survivors. *Supportive care in cancer : official journal of the Multinational Association of Supportive Care in Cancer* 2019; **27**(6): 2049-56.

541. Lee L, Elfassy N, Li C, et al. Valuing postoperative recovery: validation of the SF-6D health-state utility. *The Journal of surgical research* 2013; **184**(1): 108-14.

542. Liedman BL, Bennegard K, Olbe LC, Lundell LR. Predictors of postoperative morbidity and mortality after surgery for gastro-oesophageal carcinomas. *The European journal of surgery = Acta chirurgica* 1995; **161**(3): 173-80.

543. Nakamura T, Ishikawa H, Sakai T, Ayabe M, Wakabayashi K, Mutoh M, Matsuura N. Effect of physical fitness on colorectal tumor development in patients with familial adenomatous polyposis. 2019; **98**(38): e17076.

544. Norager CB, Jensen MB, Madsen MR, Qvist N, Laurberg S. Effect of darbepoetin alfa on physical function in patients undergoing surgery for colorectal cancer. A randomized, double-blind, placebo-controlled study. *Oncology* 2006; **71**(3-4): 212-20.

545. Nuri R, Moghaddasi M, Darvishi H, Izadpanah A. Effect of aerobic exercise on leptin and ghrelin in patients with colorectal cancer. *Journal of cancer research and therapeutics* 2016; **12**(1): 169-74.

546. Soumya CN, Thomas M, Ravindran S, Krishna KMJ. Preoperative incremental shuttle walk test for morbidity and mortality prediction in elective major colorectal surgery. *Indian Journal of Anaesthesia* 2022; **66**(17): S250-S6.

547. Chang YL, Tsai YF, Hsu CL, Chao YK, Hsu CC, Lin KC. The effectiveness of a nurse-led exercise and health education informatics program on exercise capacity and quality of life among cancer survivors after esophagectomy: a randomized controlled trial. *International journal of nursing studies* 2020; **101**: 103418.

548. Doganay E, Wynter-Blyth V, Halliday L, Mackinnon T, Osborn H, Moorthy K. Study of Long-Term Follow-up of Exercise Levels following Participation in a Prehabilitation Program in Esophagogastric Cancer. 2020; **38**(3): 110.

549. Gannon JA, Guinan EM, Doyle SL, Beddy P, Reynolds JV, Hussey J. Reduced fitness and physical functioning are long-term sequelae after curative treatment for esophageal cancer: a matched control study. *Diseases of the esophagus : official journal of the International Society for Diseases of the Esophagus* 2017; **30**(8): 1-7.

550. Liedman B, Johnsson E, Merke C, Ruth M, Lundell L. Preoperative adjuvant radiochemotherapy may increase the risk in patients undergoing thoracoabdominal esophageal resections. *Digestive surgery* 2001; **18**(3): 169-75.

551. von Dobeln GA, Nilsson M, Adell G, et al. Pulmonary function and cardiac stress test after multimodality treatment of esophageal cancer. *Practical radiation oncology* 2016; **6**(3): e53‐e9.

552. Cho H, Yoshikawa T, Oba MS, et al. Matched pair analysis to examine the effects of a planned preoperative exercise program in early gastric cancer patients with metabolic syndrome to reduce operative risk: the Adjuvant Exercise for General Elective Surgery (AEGES) study group. *Annals of surgical oncology* 2014; **21**(6): 2044‐50.

553. Nowak AK, Newton RU, Cruickshank T, Cormie P, Halkett GKB, Tsoi D, Galvão DA. A feasibility, safety, and efficacy evaluation of supervised aerobic and resistance exercise for patients with glioblastoma undertaking adjuvant chemoradiotherapy. *Neuro-Oncology Practice* 2023; **10**(3): 261-70.

554. Peel AB, Barlow CE, Leonard D, DeFina LF, Jones LW, Lakoski SG. Cardiorespiratory fitness in survivors of cervical, endometrial, and ovarian cancers: The Cooper Center Longitudinal Study. *Gynecologic oncology* 2015; **138**(2): 394-7.

555. Alibhai SM, Breunis H, Timilshina N, et al. Quality of life and physical function in adults treated with intensive chemotherapy for acute myeloid leukemia improve over time independent of age. *J Geriatr Oncol* 2015; **6**(4): 262-71.

556. Fateen MA, El Demerdash DM, Zayed RA, Mattar MM. Role of physical function in predicting short-term treatment outcome in Egyptian acute myeloid leukemia patients: a single center experience. 2019; **41**(1): 17.

557. Jarfelt M, Kujacic V, Holmgren D, Bjarnason R, Lannering B. Exercise echocardiography reveals subclinical cardiac dysfunction in young adult survivors of childhood acute lymphoblastic leukemia. *Pediatric blood & cancer* 2007; **49**(6): 835-40.

558. Ness KK, Baker KS, Dengel DR, Youngren N, Sibley S, Mertens AC, Gurney JG. Body composition, muscle strength deficits and mobility limitations in adult survivors of childhood acute lymphoblastic leukemia. *Pediatric blood & cancer* 2007; **49**(7): 975-81.

559. Suominen A, Haavisto A, Mathiesen S, et al. Physical Fitness and Frailty in Males after Allogeneic Hematopoietic Stem Cell Transplantation in Childhood: A Long-Term Follow-Up Study. *Cancers* 2022; **14**(14).

560. Timilshina N, Breunis H, Brandwein JM, et al. Do quality of life or physical function at diagnosis predict short-term outcomes during intensive chemotherapy in AML? *Annals of oncology : official journal of the European Society for Medical Oncology* 2014; **25**(4): 883-8.

561. de Almeida LB, Mira PAC, Fioritto AP, et al. Functional Capacity Change Impacts the Quality of Life of Hospitalized Patients Undergoing Hematopoietic Stem Cell Transplantation. *American journal of physical medicine & rehabilitation* 2019; **98**(6): 450-5.

562. Großek A, Elter T, Oberste M, et al. Feasibility and suitability of a graded exercise test in patients with aggressive hemato-oncological disease. *Support Care Cancer* 2021; **29**(8): 4859-66.

563. Kramer M, Heussner P, Herzberg PY, et al. Validation of the grip test and human activity profile for evaluation of physical performance during the intermediate phase after allogeneic hematopoietic stem cell transplantation. *Supportive care in cancer : official journal of the Multinational Association of Supportive Care in Cancer* 2013; **21**(4): 1121-9.

564. Morishita S, Wakasugi T, Tanaka T, et al. Changes in Borg scale for resistance training and test of exercise tolerance in patients undergoing allogeneic hematopoietic stem cell transplantation. *Supportive care in cancer* 2018: 1‐7.

565. Andersen AH, Vinther A, Poulsen LL, Mellemgaard A. A modified exercise protocol may promote continuance of exercise after the intervention in lung cancer patients--a pragmatic uncontrolled trial. 2013; **21**(8): 2247.

566. Arbane G, Douiri A, Hart N, et al. Effect of postoperative physical training on activity after curative surgery for non-small cell lung cancer: a multicentre randomised controlled trial. *Physiotherapy* 2014; **100**(2): 100-7.

567. Bagg LR. The 12-min walking distance; its use in the pre-operative assessment of patients with bronchial carcinoma before lung resection. *Respiration* 1984; **46**(4): 342-5.

568. Benattia A, Debeaumont D, Guyader V, Tardif C, Peillon C, Cuvelier A, Baste JM. Physiologic assessment before video thoracoscopic resection for lung cancer in patients with abnormal pulmonary function. *J Thorac Dis* 2016; **8**(6): 1170-8.

569. Boujibar F, Gillibert A, Bonnevie T, et al. The 6-minute stepper test and the sit-to-stand test predict complications after major pulmonary resection via minimally invasive surgery: a prospective inception cohort study. *J Physiother* 2022; **68**(2): 130-5.

570. Brunelli A, Al RM, Monteverde M, Borri A, Salati M, Fianchini A. Stair climbing test predicts cardiopulmonary complications after lung resection. *Chest* 2002; **121**(4): 1106-10.

571. Brunelli A, Monteverde M, Borri A, Salati M, Al Refai M, Fianchini A. Predicted versus observed maximum oxygen consumption early after lung resection. *The Annals of thoracic surgery* 2003; **76**(2): 376-80.

572. Brunelli A, Al Refai M, Monteverde M, Borri A, Salati M, Fianchini A. Predictors of exercise oxygen desaturation following major lung resection. *European journal of cardio-thoracic surgery : official journal of the European Association for Cardio-thoracic Surgery* 2003; **24**(1): 145-8.

573. Brunelli A, Monteverde M, Al Refai M, Fianchini A. Stair climbing test as a predictor of cardiopulmonary complications after pulmonary lobectomy in the elderly. *The Annals of thoracic surgery* 2004; **77**(1): 266-70.

574. Brunelli A, Xiume F, Refai M, Salati M, Marasco R, Sciarra V, Sabbatini A. Evaluation of expiratory volume, diffusion capacity, and exercise tolerance following major lung resection: a prospective follow-up analysis. *Chest* 2007; **131**(1): 141-7.

575. Brunelli A, Socci L, Refai M, Salati M, Xiume F, Sabbatini A. Quality of life before and after major lung resection for lung cancer: a prospective follow-up analysis. *Ann Thorac Surg* 2007; **84**(2): 410-6.

576. Brunelli A, Refai M, Xiume F, Salati M, Sciarra V, Socci L, Sabbatini A. Performance at symptom-limited stair-climbing test is associated with increased cardiopulmonary complications, mortality, and costs after major lung resection. *The Annals of thoracic surgery* 2008; **86**(1): 240-7; discussion 7-8.

577. Brunelli A, Pompili C, Berardi R, et al. Performance at preoperative stair-climbing test is associated with prognosis after pulmonary resection in stage I non-small cell lung cancer. *The Annals of thoracic surgery* 2012; **93**(6): 1796-800.

578. Dong J, Mao Y, Li J, He J. Stair-Climbing Test Predicts Postoperative Cardiopulmonary Complications and Hospital Stay in Patients with Non-Small Cell Lung Cancer. *Medical science monitor : international medical journal of experimental and clinical research* 2017; **23**: 1436-41.

579. Edvardsen E, Skjonsberg OH, Holme I, Nordsletten L, Borchsenius F, Anderssen SA. High-intensity training following lung cancer surgery: a randomised controlled trial. *Thorax* 2015; **70**(3): 244-50.

580. Fennelly J, Potter L, Pompili C, Brunelli A. Performance in the shuttle walk test is associated with cardiopulmonary complications after lung resections. *J Thorac Dis* 2017; **9**(3): 789-95.

581. Granger CL, Parry SM, Denehy L. The self-reported Physical Activity Scale for the Elderly (PASE) is a valid and clinically applicable measure in lung cancer. 2015; **23**(11): 3211.

582. Helminen O, Valo J, Andersen H, Söderström J, Sihvo E. Association of performance in a stair-climbing test with complications and survival after lung cancer resection in the video-assisted thoracoscopic surgery era: Population-based outcomes. *ERJ Open Research* 2021; **7**(3).

583. Ito H, Nakayama H, Yamada K, Yokose T, Masuda M. Outcomes of lobectomy in 'active' octogenarians with clinical stage I non-small-cell lung cancer. *Annals of thoracic and cardiovascular surgery : official journal of the Association of Thoracic and Cardiovascular Surgeons of Asia* 2015; **21**(1): 24-30.

584. Kadiri SB, Kerr AP, Oswald NK, et al. Fit 4 surgery, a bespoke app with biofeedback delivers rehabilitation at home before and after elective lung resection. 2019; **14**(1): 132.

585. Keenan RJ, Landreneau RJ, Maley RH, Jr S, D M, R B, S S. Segmental resection spares pulmonary function in patients with stage I lung cancer. *The Annals of thoracic surgery* 2004; **78**(1): 228-33; discussion -33.

586. Kushibe K, Kawaguchi T, Kimura M, Takahama M, Tojo T, Taniguchi S. Changes in ventilatory capacity, exercise capacity, and pulmonary blood flow after lobectomy in patients with lung cancer--which lobectomy has the most loss in exercise capacity? *Interact Cardiovasc Thorac Surg* 2008; **7**(6): 1011-4.

587. Liu Z, Qiu T, Pei L, et al. Two-Week Multimodal Prehabilitation Program Improves Perioperative Functional Capability in Patients Undergoing Thoracoscopic Lobectomy for Lung Cancer: A Randomized Controlled Trial. *Anesth Analg* 2020; **131**(3): 840-9.

588. Nakagawa T, Chiba N, Saito M, Sakaguchi Y, Ishikawa S. Clinical relevance of decreased oxygen saturation during 6-min walk test in preoperative physiologic assessment for lung cancer surgery. *General thoracic and cardiovascular surgery* 2014; **62**(10): 620-6.

589. Nikolic I, Majeric-Kogler V, Plavec D, Maloca I, Slobodnjak Z. Stairs climbing test with pulse oximetry as predictor of early postoperative complications in functionally impaired patients with lung cancer and elective lung surgery: prospective trial of consecutive series of patients. *Croatian medical journal* 2008; **49**(1): 50-7.

590. Ninan M, Sommers KE, Landreneau RJ, et al. Standardized exercise oximetry predicts postpneumonectomy outcome. *The Annals of thoracic surgery* 1997; **64**(2): 328-32; discussion 32-3.

591. Refai M, Pompili C, Salati M, Xiume F, Sabbatini A, Brunelli A. Can maximal inspiratory and expiratory pressures during exercise predict complications in patients submitted to major lung resections? A prospective cohort study. *European journal of cardio-thoracic surgery : official journal of the European Association for Cardio-thoracic Surgery* 2014; **45**(4): 665-69; discussion 9-70.

592. Varela G, Cordovilla R, Jimenez MF, Novoa N. Utility of standardized exercise oximetry to predict cardiopulmonary morbidity after lung resection. *European journal of cardio-thoracic surgery : official journal of the European Association for Cardio-thoracic Surgery* 2001; **19**(3): 351-4.

593. Weinstein H, Bates AT, Spaltro BE, Thaler HT, Steingart RM. Influence of preoperative exercise capacity on length of stay after thoracic cancer surgery. *The Annals of thoracic surgery* 2007; **84**(1): 197-202.

594. Win T, Jackson A, Groves AM, Wells FC, Ritchie AJ, Munday H, Laroche CM. Relationship of shuttle walk test and lung cancer surgical outcome. *European journal of cardio-thoracic surgery : official journal of the European Association for Cardio-thoracic Surgery* 2004; **26**(6): 1216-9.

595. Win T, Jackson A, Sharples L, Groves AM, Wells FC, Ritchie AJ, Laroche CM. Cardiopulmonary exercise tests and lung cancer surgical outcome. *Chest* 2005; **127**(4): 1159-65.

596. Win T, Groves AM, Ritchie AJ, Wells FC, Cafferty F, Laroche CM. The effect of lung resection on pulmonary function and exercise capacity in lung cancer patients. *Respiratory care* 2007; **52**(6): 720-6.

597. Adams MJ, Ng AK, Mauch P, Lipsitz SR, Winters P, Lipshultz SE. Peak oxygen consumption in Hodgkin's lymphoma survivors treated with mediastinal radiotherapy as a predictor of quality of life 5 years later. 2015; **39**(2): 93.

598. Barği G, Boşnak Güçlü M, Türköz Sucak G. How does myeloid or lymphoid origin of hematologic malignancy affect pulmonary function, muscle strength, exercise capacity, and quality of life? *Turkish Journal of Physiotherapy and Rehabilitation* 2020; **31**(2): 115-22.

599. Cox MC, Nusca SM, Di Landro F, et al. Exercise training (ET) in adult and elderly patients receiving anti-lymphoma treatments is feasible and may improve the provision of care. *Leuk Lymphoma* 2021; **62**(3): 560-70.

600. Groarke JD, Tanguturi VK, Hainer J, et al. Abnormal exercise response in long-term survivors of hodgkin lymphoma treated with thoracic irradiation: evidence of cardiac autonomic dysfunction and impact on outcomes. *Journal of the American College of Cardiology* 2015; **65**(6): 573-83.

601. Gustavsson A, Eskilsson J, Landberg T, Svahn-Tapper G, White T, Wollmer P, Akerman M. Late cardiac effects after mantle radiotherapy in patients with Hodgkin's disease. *Annals of oncology : official journal of the European Society for Medical Oncology* 1990; **1**(5): 355-63.

602. Lund MB, Kongerud J, Boe J, Nome O, Abrahamsen AF, Ihlen H, Forfang K. Cardiopulmonary sequelae after treatment for Hodgkin's disease: increased risk in females? 1996; **7**(3): 257.

603. Pohjola-Sintonen S, Totterman KJ, Salmo M, Siltanen P. Late cardiac effects of mediastinal radiotherapy in patients with Hodgkin's disease. *Cancer* 1987; **60**(1): 31-7.

604. Persoon S, Chin AMJM, Buffart LM, et al. Randomized controlled trial on the effects of a supervised high intensity exercise program in patients with a hematologic malignancy treated with autologous stem cell transplantation: Results from the EXIST study. *PloS one* 2017; **12**(7): e0181313.

605. Adamsen L, Quist M, Midtgaard J, et al. The effect of a multidimensional exercise intervention on physical capacity, well-being and quality of life in cancer patients undergoing chemotherapy. *Support Care Cancer* 2006; **14**(2): 116-27.

606. Adamsen L, Quist M, Andersen C, et al. Effect of a multimodal high intensity exercise intervention in cancer patients undergoing chemotherapy: randomised controlled trial. *BMJ (Clinical research ed)* 2009; **339**: b3410.

607. Baumann FT, Zopf EM, Nykamp E, et al. Physical activity for patients undergoing an allogeneic hematopoietic stem cell transplantation: benefits of a moderate exercise intervention. *Eur J Haematol* 2011; **87**(2): 148-56.

608. Bird L, Arthur A, Niblock T, Stone R, Watson L, Cox K. Rehabilitation programme after stem cell transplantation: randomized controlled trial. *Journal of advanced nursing* 2010; **66**(3): 607-15.

609. Broderick JM, Guinan E, Kennedy MJ, et al. Feasibility and efficacy of a supervised exercise intervention in de-conditioned cancer survivors during the early survivorship phase: the PEACH trial. 2013; **7**(4): 551.

610. Cantwell M, Moyna N, McCaffrey N, et al. A two-arm non-randomised trial of MedEx IMPACT: a community-based, physical activity behaviour change intervention for survivors of cancer. *Support Care Cancer* 2024; **32**(2): 95.

611. Cella D, Viswanathan HN, Hays RD, et al. Development of a fatigue and functional impact scale in anemic cancer patients receiving chemotherapy. 2008; **113**(6): 1480.

612. Coletta AM, Rose NB, Johnson AF, et al. The impact of a hospital-based exercise oncology program on cancer treatment-related side effects among rural cancer survivors. *Support Care Cancer* 2021; **29**(8): 4663-72.

613. Coletta AM, Playdon MC, Baron KG, et al. The association between time-of-day of habitual exercise training and changes in relevant cancer health outcomes among cancer survivors. *PLoS One* 2021; **16**(10): e0258135.

614. De Backer IC, Van BE, Vreugdenhil A, Nijziel MR, Kester AD, Schep G. High-intensity strength training improves quality of life in cancer survivors. *Acta oncologica (Stockholm, Sweden)* 2007; **46**(8): 1143-51.

615. Dimeo F, Fetscher S, Lange W, Mertelsmann R, Keul J. Effects of aerobic exercise on the physical performance and incidence of treatment-related complications after high-dose chemotherapy. *Blood* 1997; **90**(9): 3390-4.

616. Dimeo FC, Thomas F, Raabe-Menssen C, Propper F, Mathias M. Effect of aerobic exercise and relaxation training on fatigue and physical performance of cancer patients after surgery. A randomised controlled trial. *Supportive care in cancer : official journal of the Multinational Association of Supportive Care in Cancer* 2004; **12**(11): 774-9.

617. Drake D, Falzer P, Xistris D, Robinson G, Roberge M. Physical fitness training: outcomes for adult oncology patients. *Clinical nursing research* 2004; **13**(3): 245-64.

618. Fioritto AP, Oliveira CC, Albuquerque VS, Almeida LB, Granger CL, Denehy L, Malaguti C. Individualized in-hospital exercise training program for people undergoing hematopoietic stem cell transplantation: a feasibility study. *Disability & Rehabilitation* 2021; **43**(3): 386-92.

619. George SM, Alfano CM, Groves J, Karabulut Z, Haman KL, Murphy BA, Matthews CE. Objectively measured sedentary time is related to quality of life among cancer survivors. *PloS one* 2014; **9**(2): e87937.

620. Gresham G, Dy SM, Zipunnikov V, et al. Fatigability and endurance performance in cancer survivors: Analyses from the Baltimore Longitudinal Study of Aging. 2018; **124**(6): 1279.

621. Groarke JD, Payne DL, Claggett B, et al. Association of post-diagnosis cardiorespiratory fitness with cause-specific mortality in cancer. *Eur Heart J Qual Care Clin Outcomes* 2020; **6**(4): 315-22.

622. Knobf MT, Jeon S, Smith B, et al. The Yale Fitness Intervention Trial in female cancer survivors: Cardiovascular and physiological outcomes. *Heart & Lung* 2017; **46**(5): 375-81.

623. Knutsen L, Quist M, Midtgaard J, Rorth M, Adamsen L. Maximum physical capacity testing in cancer patients undergoing chemotherapy: qualitative findings from an exercise program. *Scand J Med Sci Sports* 2006; **16**(6): 403-11.

624. Lestuzzi C, Stolfo D, De Paoli A, et al. Cardiotoxicity from Capecitabine Chemotherapy: Prospective Study of Incidence at Rest and During Physical Exercise. *Oncologist* 2022; **27**(2): e158-e67.

625. Makinen L, Makipernaa A, Rautonen J, Heino M, Pyrhonen S, Laitinen LA, Siimes MA. Long-term cardiac sequelae after treatment of malignant tumors with radiotherapy or cytostatics in childhood. 1990; **65**(9): 1913.

626. Midtgaard J, Rorth M, Stelter R, et al. The impact of a multidimensional exercise program on self-reported anxiety and depression in cancer patients undergoing chemotherapy: a phase II study. *Palliat Support Care* 2005; **3**(3): 197-208.

627. Morishita S, Kaida K, Yamauchi S, et al. Early-phase differences in health-related quality of life, psychological status, and physical function between human leucocyte antigen-haploidentical and other allogeneic haematopoietic stem cell transplantation recipients. *European journal of oncology nursing : the official journal of European Oncology Nursing Society* 2015; **19**(5): 443-50.

628. Oldervoll LM, Loge JH, Lydersen S, et al. Physical exercise for cancer patients with advanced disease: a randomized controlled trial. *The oncologist* 2011; **16**(11): 1649-57.

629. Quinn SE, Crandell CE, Blake ME, et al. The Correlative Strength of Objective Physical Assessment Against the ECOG Performance Status Assessment in Individuals Diagnosed With Cancer. 2020; **100**(3): 416.

630. Rabin C, Pinto B, Fava J. Randomized Trial of a Physical Activity and Meditation Intervention for Young Adult Cancer Survivors. *Journal of adolescent and young adult oncology* 2016; **5**(1): 41-7.

631. Repka CP, Peterson BM, Brown JM, Lalonde TL, Schneider CM, Hayward R. Cancer type does not affect exercise-mediated improvements in cardiorespiratory function and fatigue. 2014; **13**(6): 473.

632. Ruiz-Casado A, Verdugo AS, Solano MJ, et al. Objectively assessed physical activity levels in Spanish cancer survivors. 2014; **41**(1): E12.

633. Thorsen L, Skovlund E, Stromme SB, Hornslien K, Dahl AA, Fossa SD. Effectiveness of physical activity on cardiorespiratory fitness and health-related quality of life in young and middle-aged cancer patients shortly after chemotherapy. *J Clin Oncol* 2005; **23**(10): 2378-88.

634. Thorsen L, Nystad W, Stigum H, et al. Cardiorespiratory fitness in relation to self-reported physical function in cancer patients after chemotherapy. *J Sports Med Phys Fitness* 2006; **46**(1): 122-7.

635. van Haren I, Staal JB, Potting CM, Atsma F, Hoogeboom TJ, Blijlevens NMA, Nijhuis-van der Sanden MWG. Physical exercise prior to hematopoietic stem cell transplantation: A feasibility study. 2018; **34**(10): 747.

636. Velensek V, Mazic U, Krzisnik C, Demsar D, Jazbec J, Jereb B. Cardiac damage after treatment of childhood cancer: a long-term follow-up. *BMC cancer* 2008; **8**: 141.

637. Beydoun N, Bucci JA, Chin YS, Spry N, Newton R, Galvao DA. Prospective study of exercise intervention in prostate cancer patients on androgen deprivation therapy. 2014; **58**(3): 369.

638. Capela A, Antunes P, Coelho CA, et al. Effects of walking football on adherence, safety, quality of life and physical fitness in patients with prostate cancer: Findings from the PROSTATA_MOVE randomized controlled trial. *Frontiers in Oncology* 2023; **13**((Capela A.; Antunes P.; Coelho C.A.; Garcia C.L.; Custódio S.; Costa T.; Amarelo A.; Silva J.; Joaquim A.; Viamonte S.; Alves A.J., ajalves@umaia.pt) ONCOMOVE® – Associação de Investigação de Cuidados de Suporte em Oncologia (AICSO), Vila Nova de Gaia, Po).

639. Cormie P, Galvao DA, Spry N, et al. Can supervised exercise prevent treatment toxicity in patients with prostate cancer initiating androgen-deprivation therapy: a randomised controlled trial. *BJU Int* 2015; **115**(2): 256-66.

640. Galvao DA, Taaffe DR, Spry N, Joseph D, Newton RU. Combined resistance and aerobic exercise program reverses muscle loss in men undergoing androgen suppression therapy for prostate cancer without bone metastases: a randomized controlled trial. *J Clin Oncol* 2010; **28**(2): 340-7.

641. Galvao DA, Spry N, Denham J, et al. A Multicentre Year-long Randomised Controlled Trial of Exercise Training Targeting Physical Functioning in Men with Prostate Cancer Previously Treated with Androgen Suppression and Radiation from TROG 03.04 RADAR. *Eur Urol* 2014; **65**(5): 856-64.

642. Gong J, Payne D, Caron J, et al. Reduced Cardiorespiratory Fitness and Increased Cardiovascular Mortality After Prolonged Androgen Deprivation Therapy for Prostate Cancer. *JACC: CardioOncology* 2020; **2**(4): 553-63.

643. Henriksson P, Linde B, Edhag O. Deleterious effects of low-dose oestrogen therapy on coronary status in patients with prostatic cancer. *European heart journal* 1987; **8**(7): 779-84.

644. Henriksson P, Edhag O, Eriksson A, Johansson SE. Patients at high risk of cardiovascular complications in oestrogen treatment of prostatic cancer. *British journal of urology* 1989; **63**(2): 186‐90.

645. Lam T, Cheema B, Hayden A, et al. Androgen deprivation in prostate cancer: benefits of home-based resistance training. *Sports Medicine - Open* 2020; **6**(1).

646. Lauwick S, Kim DJ, Mistraletti G, Carli F. Functional walking capacity as an outcome measure of laparoscopic prostatectomy: the effect of lidocaine infusion. *British journal of anaesthesia* 2009; **103**(2): 213-9.

647. Nilsen TS, Raastad T, Skovlund E, et al. Effects of strength training on body composition, physical functioning, and quality of life in prostate cancer patients during androgen deprivation therapy. *Acta oncologica (Stockholm, Sweden)* 2015; **54**(10): 1805-13.

648. O'Neill RF, Haseen F, Murray LJ, O'Sullivan JM, Cantwell MM. A randomised controlled trial to evaluate the efficacy of a 6-month dietary and physical activity intervention for patients receiving androgen deprivation therapy for prostate cancer. 2015; **9**(3): 431.

649. Park YH, Lee JI, Lee JY, et al. Internet of things-based lifestyle intervention for prostate cancer patients on androgen deprivation therapy: A prospective, multicenter, randomized trial. *American Journal of Cancer Research* 2021; **11**(11): 5496-507.

650. Windsor PM, Nicol KF, Potter J. A randomized, controlled trial of aerobic exercise for treatment-related fatigue in men receiving radical external beam radiotherapy for localized prostate carcinoma. *Cancer* 2004; **101**(3): 550-7.

651. Jones LW, Courneya KS, Mackey JR, et al. Cardiopulmonary function and age-related decline across the breast cancer survivorship continuum. *Journal of clinical oncology : official journal of the American Society of Clinical Oncology* 2012; **30**(20): 2530-7.

652. Jones LW, Watson D, Herndon JE, 2nd, Eves ND, Haithcock BE, Loewen G, Kohman L. Peak oxygen consumption and long-term all-cause mortality in nonsmall cell lung cancer. *Cancer* 2010; **116**(20): 4825-32.
